# 5-((3-Amidobenzyl)oxy)nicotinamides as SIRT2 Inhibitors: A Study of Constrained Analogs

**DOI:** 10.3390/molecules28227655

**Published:** 2023-11-18

**Authors:** Teng Ai, Daniel J. Wilson, Liqiang Chen

**Affiliations:** Center for Drug Design, College of Pharmacy, University of Minnesota, Minneapolis, MN 55455, USA

**Keywords:** sirtuins, sirtuin inhibitors, SIRT1, SIRT2, SIRT3, constrained inhibitors

## Abstract

SIRT2 is a member of NAD^+^-dependent sirtuins and its inhibition has been proposed as a promising therapeutic approach for treating human diseases, including neurodegenerative diseases, cancer, and infections. Expanding SIRT2 inhibitors based on the 3-aminobenzyloxy nicotinamide core structure, we have synthesized and evaluated constrained analogs and selected stereoisomers. Our structure-activity relationship (SAR) study has revealed that 2,3-constrained (*S*)-isomers possess enhanced in vitro enzymatic inhibitory activity against SIRT2 and retain excellent selectivity over SIRT1 and SIRT3, provided that a suitable ring A is used. This current study further explores SIRT2 inhibitors based on the 3-aminobenzyloxy nicotinamide scaffold and contributes to the discovery of potent, selective SIRT2 inhibitors that have been actively pursued for their potential therapeutic applications.

## 1. Introduction

Sirtuins are a group of nicotinamide adenine dinucleotide (NAD^+^)-dependent protein deacetylase that use NAD^+^ as a cofactor to remove an acetyl or other acyl group from the lysine residue of various substrates [1,2]. Among the seven human sirtuins (SIRT1-SIRT7), SIRT2 is the only one that mainly resides in the cytoplasm; however, it can be localized to the nucleus [3] and can also be detected in the mitochondria [4,5]. SIRT2 is implicated in different human diseases, including neurodegenerative diseases, cancer, and infections [6]. Therefore, as the most abundant sirtuin homolog in the brain [7,8], selective inhibition of SIRT2 has been proposed as a promising therapeutic approach for Parkinson’s disease [9] and Alzheimer’s disease [10]. While the roles of SIRT2 are complex and might vary in different cancers, pharmaceutical inhibition of SIRT2 holds promise as a potential treatment for certain types of cancer [11]. More recently, SIRT2 as a proviral host factor has been investigated, suggesting that SIRT2 is a viable antiviral target [12,13,14,15].

**Figure 1:**
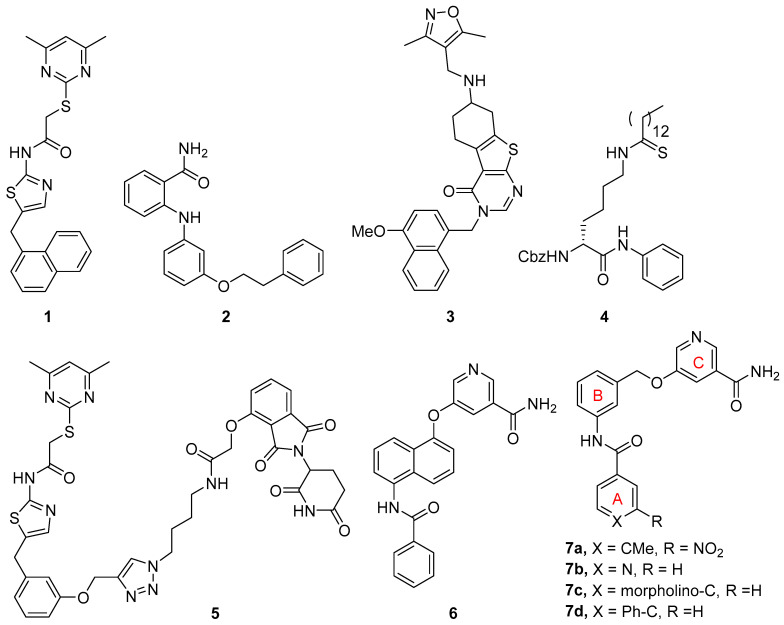
Selected SIRT2 inhibitors.

Interest in discovering potent and selective SIRT2 inhibitors and exploring their potential therapeutic applications in various diseases has resulted in a number of SIRT2 inhibitors, which have been updated and reviewed [11,16,17,18]. Remarkable among these SIRT2 inhibitors are those that bind into the ligand-induced, hydrophobic extended C-site and selectivity pocket, including SirReal2 (**1**, Figure 1) and its analogs [19,20], 2-anilinobenzamide **2** [21], and thienopyrimidinone **3** [22], which all possess excellent selectivity over SIRT1 and SIRT3. Mechanism-based SIRT2 inhibitors, such as thioamide **4**, have also been developed [23,24,25]. Another significant development is the discovery of SIRT2 degraders based on the proteolysis-targeting chimera (PROTAC) strategy by introducing an E3 ligase-engaging moiety [26]. This new class of SIRT2 inhibitors is exemplified by compound **5** [27], which is based on the structure of SirReal inhibitors, and one derived from a mechanism-based inhibitor [28].

Through a fragment-based approach, we have discovered potent and selective SIRT2 inhibitors based on a 5-aminonaphthalen-1-yloxy nicotinamide core structure as exemplified by compound **6** (Figure 1) [29]. Further simplification of the central naphthalene ring has resulted in SIRT2 inhibitors that feature a 3-aminobenzyloxy nicotinamide scaffold as represented by compound **7** [30]. An extensive structure-activity relationship (SAR) study on ring A has led to excellent SIRT2 inhibitors including compounds **7a**–**d**. In our continued efforts to discover novel SIRT2 inhibitors, here we report constrained analogs based on the 3-aminobenzyloxy nicotinamide core structure. In these constrained compounds, we chose to rigidify the benzyloxy linker between rings B and C (Figure 1) by incorporating a five- or six-membered ring as part of the linker. We aimed to discover constrained inhibitors that allow for optimal projection of the terminal nicotinamide moiety into the C-pocket, which is occupied by the nicotinamide portion of NAD during the deacylation reaction.

## 2. Results and Discussion

**Figure 2:**
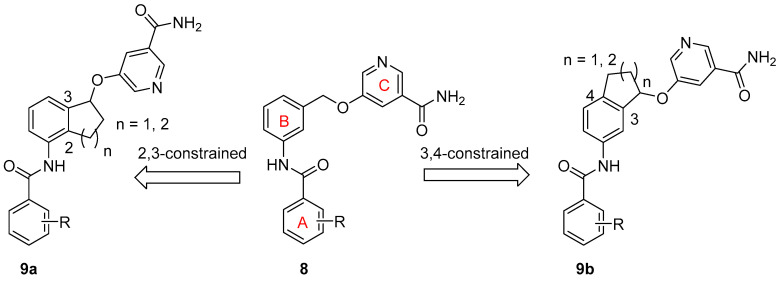
Design of constrained SIRT2 inhibitors.

**SAR Study.** There are two approaches to designing constrained analogs of compound **8** by introducing a five-member ring through linking either positions 2 and 3 (**9a**, Figure 2) or positions 3 and 4 (**9b**). Besides a five-member ring, a six-member ring can also be explored (Figure 2). Accordingly, we first prepared compounds **15a**–**b** and **16a**–**b**, which featured a 2,3-five-member ring and a 3,4-five-member ring, respectively. Furthermore, six-member ring analogs **17** and **18** were also obtained. For these initial compounds, 4-methyl-3-nitrophenyl or 4-pyridyl was used as ring A because they were identified as good moieties in our previous SAR study (Table 1) [30].

These racemic compounds were subsequently tested against human SIRT1-3 (Table 1) with their non-constrained counterparts **7a**–**b** as reference compounds. The enzymatic results showed that constrained analogs retained selectivity against SIRT2 over SIRT1 and SIRT3. For 2,3-constrained five-member ring inhibitors, compound **15a** was about 3-fold less active than its non-constrained counterpart **7a**. However, when 4-pyridyl was used as ring A, the resulting **15b** exhibited anti-SIRT2 activity comparable to that of **7b**, suggesting that the nature of ring A could be an important factor. For 3,4-constrained isomers, a 5- and 3-fold reduction in SIRT2 inhibition was observed in **16a** and **16b** when compared with non-constrained **7a** and **7b**, respectively. On the other hand, incorporation of a six-member ring in **17** or **18** led to an approximately 30-fold reduction in SIRT2 inhibition. These results indicated that a five-member ring was tolerated while a six-member one was clearly detrimental to anti-SIRT2 activity.

Having established the viability of five-member ring constrained analogs, we proceeded to assess the effect of stereochemistry. To that end, we prepared compounds **19**–**22** (Table 2). For ring A, we chose to evaluate three variations (4-pyridyl, 4-morpholinophenyl, and 1,1′-biphenyl), which all showed high inhibitory activity against SIRT2 and good selectivity in the previous study [30]. These new compounds and their non-constrained counterparts **7b**–**d** were then evaluated in our in-house SIRT1-3 assays. In these assays, NAD and the peptide substrate were used at the *K*_M_ values determined for each enzyme, allowing for accurate assessment of an inhibitor’s selectivity against SIRT1-3. We first focused on 2,3-constrained stereoisomers. Among the (*S*)-isomers, **19a** (with 4-pyridyl as its ring A) possessed anti-SIRT2 activity nearly identical to that of non-constrained **7b**, a trend that was also observed in its racemic counterpart **15b** (Table 1); however, **19a** displayed improved selectivity over SIRT1 and SIRT3 when compared with **7b**. In comparison, (*S*)-isomer **19b**’s anti-SIRT2 capacity was slightly weaker than its non-constrained counterpart **7c** but retained a similar selectivity profile. When 1,1′-biphenyl was used as ring A, the resulting non-constrained **7d** exhibited excellent selectivity over SIRT1 and SIRT3. Remarkably, **7d**-derived (*S*)-isomer **19c** showed more than 3-fold improvement in anti-SIRT2 activity (64 nM), leading to >1500-fold selectivity against SIRT2 over SIRT1 and SIRT3. With its low nanomolar inhibitory activity and excellent selectivity profile, compound **19c** is among the most potent and selective SIRT2 inhibitors reported [11,16,17,18]. Also noticeably, ring A appeared to have different effects on constrained (*S*)-isomers’ ability to inhibit SIRT2 in comparison with their corresponding non-constrained ones. While 4-pyridyl had essentially no effect, 4-morpholinophenyl and 1,1′-biphenyl reduced and enhanced anti-SIRT2 inhibition, respectively. There is no definitive explanation for this phenomenon, but it might be because ring A binds into the relatively hydrophobic lysine substrate channel (see Computational Modeling below). Therefore, a lipophilic substitution (at position 4 of ring A) like a phenyl ring in 1,1′-biphenyl is preferred while a relatively polar morpholine ring in 4-morpholinophenyl is not well tolerated and 4-pyridyl has a minimal effect. We next examined (*R*)-isomers **20a**–**c**, which exhibited approximately a 5-, 12-, and 2-fold reduction in anti-SIRT2 activity, respectively; however, these compounds remained relatively selective against SIRT2. These results showed that a lipophilic ring A was still preferred in (*R*)-isomers. It was also clear that the anti-SIRT2 activity and selectivity of (*S*)-isomers were significantly higher than the corresponding (*R*)-isomers (Table 2). Taken together, for 2,3-constrained inhibitors, (*S*)-isomers were superior to (*R*)-isomers especially when a lipophilic ring A like 1,1′-biphenyl was used.

We continued to examine 3,4-constrained stereoisomers **21** and **22**. While (*R*)-isomers **21a**–**c** and (*S*)-isomers **22a**–**c** exhibited very similar anti-SIRT2 activity and selectivity profiles, their inhibitory capacity against SIRT2 was significantly diminished when compared with their non-constrained counterparts **7b**–**d** as well as 2,3-constrained (*S*)-isomers **19a**–**c**, respectively. These results clearly demonstrated that a 3,4-five-member ring was not a favorable structural feature. Taken together, 2,3-constrained (*S*)-isomers were the preferred isomers among those studied. Equipped with a suitable ring A like 1,1′-biphenyl, these stereoisomers could possess superior inhibitory activity and selectivity against SIRT2.

**Figure 3:**
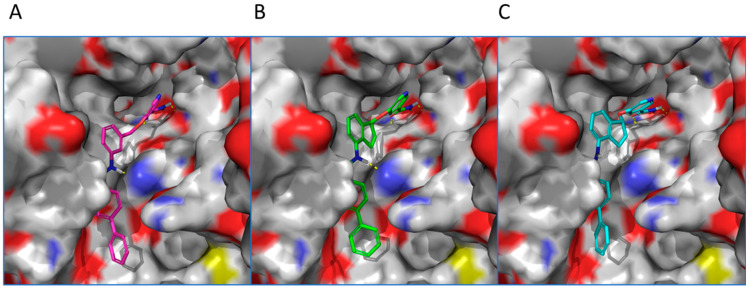
Proposed binding modes of selected SIRT2 inhibitors docked into the active site of human SIRT2 (PDB: 1j8f) (surface representation). (**A**) Compound **7d** (magenta). (**B**) Compound **19c** (green). (**C**) Compound **20c** (cyan).

**Computational Modeling.** To understand the binding modes of constrained analogs versus non-constrained ones, compounds **19c** and **20c** were selected for computational docking. Reference compound **7d** was first docked into human SIRT2 (PDB: 1j8f) [31]. A top pose showed that compound **7d**’s nicotinamide moiety (ring C) was projected into the C-site of the NAD^+^ binding pocket and the remaining of the molecule fitted in SIRT2′s substrate binding channel (Figure 3). The primary amide of nicotinamide formed hydrogen bonds with the backbone NH of Ile 169 and the side chain carboxylate of Asp170. The aromatic rings were engaged in multiple π-π interactions: nicotinamide with Phe 96, the central ring B with Phe 119 and His 187, and ring A with Phe 235. The docking of constrained (*S*)-isomer **19c** revealed a binding mode very similar to that of **7d** (Figure 3). The primary amide participated in hydrogen bonds with Ile 169 and Asp170. Rings A, B, and C formed π-π interactions with Phe 96, Phe 119, and Phe 235, respectively. Furthermore, like **7d**, the NH of compound **19c**’s secondary amide is positioned close to His 187; therefore, a hydrogen bond is possible. When (*R*)-isomer **20c** was docked into human SIRT2, its top pose generally mirrored the binding orientation of compounds **7d** and **19c**. However, **20c**’s binding mode lacked an π-π interaction with Phe 96. In addition, the NH of compound secondary amide is oriented away from His 187 to preclude a potential hydrogen bond interaction. These observations could explain compound **20c**’s inferior anti-SIRT2 activity in comparison with **19c**. Taken together, while incorporation of a 2,3-five-member ring did not drastically alter the binding modes, an (*S*)-isomer was desired due to its augmented protein-ligand interactions, which could account for (*S*)-isomer’s enhanced inhibitory activity.

**Scheme 1:**
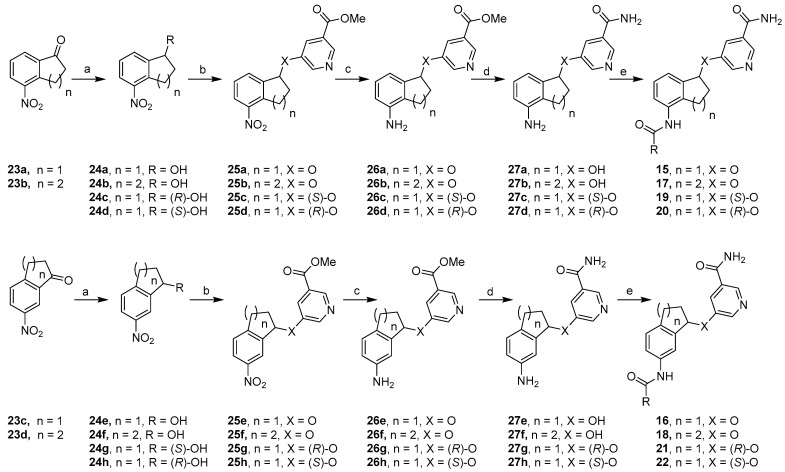
Reagents and conditions: (a) for **24a**–**b** and **24 e**–**f**, NaBH_4_, MeOH, 92–95%; for **24c** and **24h**, BH_3_·SMe_2_, (*S*)-CBS, CH_2_Cl_2_, −20 °C, 84% and 90%, respectively; for **24d** and **24g**, BH_3_·SMe_2_, (*R*)-CBS, CH_2_Cl_2_, −20 °C, both 89%; (b) methyl 5-hydroxynicotinate, DIAD, Ph_3_P, THF, 64–76%; (c) NaBH_4_, NiCl_2_·6H_2_O, MeOH, 59–94%; (d) 7 N methanolic NH_3_, 70 °C, 79–98%; (e) RCOCl, Et_3_N; or RCOOH, coupling agents, 4–42%.

**Chemical Synthesis.** Scheme 1 depicted a general synthetic sequence that was used to prepare constrained analogs. The starting ketones **23a** and **23c** were prepared through nitration of 1-indanone according to reported procedures [32]. Nitration of tetralone under similar conditions gave ketones **23b** and **23d** in good yields. The synthesis of 2,3-constrained five-member ring analogs **15a**–**b** started with ketone **23a**, which was reduced to secondary alcohol **24a** in excellent yields. Through Mitsunobu reaction, a methyl nicotinate moiety was introduced in good yields to give methyl ester **25a**, whose nitro group was further reduced in moderate yields to form aniline **26a**. The methyl ester functionality was subsequently converted into the corresponding primary amide in excellent yields, resulting in aniline **27a**. Upon amide formation, final compounds **15a**–**b** were obtained in low yields. This general synthetic sequence was also adopted to prepare 3,4-constrained five-member ring compounds **16a**–**b** as well as six-member ring analogs **17** and **18** in similar yields. To prepare (*S*)-isomers **19a**–**c**, enantioselective Corey-Bakshi-Shibata reduction was used [33]. (*S*)-CBS catalyst provided an (*R*)-hydroxyl group as shown in intermediate **24c** in excellent yields, which was then subjected to the same general synthetic sequence. Among the ensuing chemical transformations was Mitsunobu reaction, which inverted the stereochemistry of the chiral center, eventually leading to (*S*)-isomers **19a**–**c** in low overall yields. Analogously, **24d** was obtained by using (*R*)-CBS catalyst, and this intermediate was transformed into (*R*)-isomers **20a**–**c**. Furthermore, 3,4-constrained five-member ring analogs **21a**–**c** and **22a**–**c** were prepared in similar yields via the same synthetic sequence (Scheme 1).

## 3. Conclusions

Expanding our research on SIRT2 inhibitors based on the 3-aminobenzyloxy nicotinamide core structure, we have synthesized and evaluated constrained analogs by incorporating a five- or six-membered ring via different connection modes (either positions 2 and 3 or positions 3 and 4). While six-member rings are detrimental to anti-SIRT2 activity, introducing five-member rings is a viable structural modification. Examination of the stereoisomers derived from five-member rings reveals that 2,3-constrained isomers are preferred over 3,4-constrained ones. Furthermore, 2,3-constrained (*S*)-isomers are desired. When combined with a suitable ring A such as 1,1′-biphenyl, the resulting stereoisomer **19c** possesses enhanced inhibitory activity against SIRT2 and retains excellent selectivity over SIRT1 and SIRT3. These results suggest that it is beneficial to increase the rigidity of the linker between rings B and C. Besides 2,3-constrained five-member rings examined in this study, linkers containing a rigid double or triple bond could also be envisioned. Our study has demonstrated that ring A is a key determinant of anti-SIRT2 activity and selectivity; therefore, it is important to explore the structural diversity of ring A to properly match modifications performed on other elements of SIRT2 inhibitors. Taken together, we have discovered potent and selective constrained SIRT2 inhibitors based on 3-aminobenzyloxy nicotinamide, representing a significant addition to the growing list of novel SIRT2 inhibitors. The important SAR information gathered in the current study has also shed light on key structural factors for SIRT2 inhibition and will certainly facilitate the discovery of new chemotypes as SIRT2 inhibitors.

## 4. Experimental

**Chemical Synthesis.** All commercial reagents were used as provided unless otherwise indicated. An anhydrous solvent dispensing system (J. C. Meyer) using 2 packed columns of neutral alumina was used for drying THF, Et_2_O, and CH_2_Cl_2_, whereas 2 packed columns of molecular sieves were used to dry DMF. Solvents were dispensed under argon. Flash chromatography was performed with Ultra Pure silica gel (Silicycle, Quebec City, QC, Canada) or with RediSep R_f_ silica gel columns on a Teledyne ISCO CombiFlash^®^ R_f_ system using the solvents as indicated. Nuclear magnetic resonance spectra were recorded on a Varian 600 MHz with Me_4_Si or signals from residual solvent as the internal standard for ^1^H. Chemical shifts are reported in ppm, and signals are described as s (singlet), d (doublet), t (triplet), q (quartet), m (multiplet), bs (broad singlet), and dd (double doublet). Values given for coupling constants are first order. High resolution mass spectra were recorded on an Agilent TOF II TOF/MS instrument (Santa Clara, CA, USA) equipped with either an ESI or APCI interface. Analysis of sample purity was performed on an Agilent 1200 Infinity series HPLC system with a Phenomenex Gemini C18 column (5 μ, 4.6 × 250 mm). HPLC conditions were as follows: solvent A = water, solvent B = MeCN or MeOH, and flow rate = 2.0 mL/min. Compounds were eluted with a gradient of from 30% to 100% MeCN/water or from 40% to 100% MeOH/water in 15 min. Purity was determined by the absorbance at 254 nm. All tested compounds have a purity of ≥95% except for compound **20a** with a purity of 94% using the MeOH/water method and compound **22b** with a purity of 92% using the MeCN/water method.

*5-((4-(4-Methyl-3-nitrobenzamido)-2,3-dihydro-1H-inden-1-yl)oxy)nicotinamide* (**15a**). To a solution of **27a** (57 mg, 0.21 mmol) and Et_3_N (33 mg, 0.32 mmol) in anhydrous DMF (2 mL) at rt was added 4-methyl-3-nitrobenzoyl chloride (84 mg, 0.42 mmol) dropwise. After the mixture was allowed to stir at rt for 12 h, the reaction was quenched with saturated NH_4_Cl (10 mL). The resulting mixture was extracted with EtOAc, and the organic phase was washed with water and brine, dried over anhydrous Na_2_SO_4_, and concentrated in vacuo. The residue was purified by flash column chromatography (MeOH/CH_2_Cl_2_) to afford compound **15a** as a light yellow solid (37 mg, 42%). ^1^H NMR (DMSO-*d*_6_, 600 MHz) δ 10.32 (s, 1H), 8.67 (s, 1H), 8.57 (s, 1H), 8.49 (d, *J* = 2.4 Hz, 1H), 8.22 (d, *J* = 8.4 Hz, 1H), 8.16 (s, 1H), 7.92 (s, 1H), 7.69 (d, *J* = 7.8 Hz, 1H), 7.63 (s, 1H), 7.47–7.43 (m, 1H), 7.34–7.29 (m, 2H), 6.06 (dd, *J* = 3.9, 5.7 Hz, 1H), 3.07–3.01 (m, 1H), 2.92–2.85 (m, 1H), 2.64–2.57 (m, 4H), 2.09–2.03 (m, 1H). HRMS (ESI^+^) calcd for C_23_H_21_N_4_O_5_ (M + H)^+^ 433.1506 found 433.1509.

The following compounds were prepared through an amide formation reaction in a manner similar to that described for the preparation of compound **15a**.

*5-((4-(Isonicotinamido)-2,3-dihydro-1H-inden-1-yl)oxy)nicotinamide* (**15b**). White solid, 8%. ^1^H NMR (DMSO-*d*_6_, 600 MHz) δ 10.32 (s, 1H), 8.80 (d, *J* = 5.4 Hz, 2H), 8.67 (s, 1H), 8.49 (d, *J* = 3.0 Hz, 1H), 8.16 (s, 1H), 7.91 (s, 1H), 7.88 (d, *J* = 4.8 Hz, 2H), 7.62 (s, 1H), 7.50–7.47 (m, 1H), 7.34–7.30 (m, 2H), 6.08–6.04 (m, 1H), 3.08–3.02 (m, 1H), 2.92–2.86 (m, 1H), 2.64–2.57 (m, 1H), 2.10–2.03 (m, 1H). HRMS (ESI^+^) calcd for C_21_H_19_N_4_O_3_ (M + H)^+^ 375.1452 found 375.1463.
*5-((6-(4-Methyl-3-nitrobenzamido)-2,3-dihydro-1H-inden-1-yl)oxy)nicotinamide* (**16a**). Light yellow solid, 19%. ^1^H NMR (DMSO-*d*_6_, 600 MHz) δ 10.48 (s, 1H), 8.67 (s, 1H), 8.56 (d, *J* = 1.2 Hz, 1H), 8.50 (d, *J* = 2.4 Hz, 1H), 8.20 (d, *J* = 6.6 Hz, 1H), 8.16 (s, 1H), 7.90 (s, 1H), 7.85 (s, 1H), 7.74 (d, *J* = 7.8 Hz, 1H), 7.68 (d, *J* = 8.4 Hz, 1H), 7.63 (s, 1H), 7.35 (d, *J* = 9.0 Hz, 1H), 6.05 (dd, *J* = 3.6, 3.6 Hz, 1H), 3.07–3.01 (m, 1H), 2.92–2.86 (m, 1H), 2.69–2.63 (m, 1H), 2.59 (s, 3H), 2.09–2.04 (m, 1H). HRMS (ESI^+^) calcd for C_23_H_21_N_4_O_5_ (M + H)^+^ 433.1506 found 433.1509.
*5-((6-(Isonicotinamido)-2,3-dihydro-1H-inden-1-yl)oxy)nicotinamide* (**16b**). White solid, 17%. ^1^H NMR (DMSO-*d*_6_, 600 MHz) δ 10.51 (s, 1H), 8.77 (d, *J* = 6.0 Hz, 2H), 8.67 (s, 1H), 8.49 (d, *J* = 2.4 Hz, 1H), 8.15 (s, 1H), 7.90 (s, 1H), 7.88–7.83 (m, 3H), 7.73 (d, *J* = 7.8 Hz, 1H), 7.62 (s, 1H), 7.35 (d, *J* = 7.8 Hz, 1H), 6.06–6.02 (m, 1H), 3.08–3.02 (m, 1H), 2.94–2.86 (m, 1H), 2.68–2.62 (m, 1H), 2.10–2.03 (m, 1H). HRMS (ESI^+^) calcd for C_21_H_19_N_4_O_3_ (M + H)^+^ 375.1452 found 375.1458.
*5-((5-(4-Methyl-3-nitrobenzamido)-1,2,3,4-tetrahydronaphthalen-1-yl)oxy)nicotinamide* (**17**). White solid, 16%. ^1^H NMR (DMSO-*d*_6_, 600 MHz) δ 10.14 (s, 1H), 8.66 (s, 1H), 8.58 (s, 1H), 8.50 (s, 1H), 8.22 (d, *J* = 7.8 Hz, 1H), 8.16 (s, 1H), 7.95 (s, 1H), 7.69 (d, *J* = 7.8 Hz, 1H), 7.63 (s, 1H), 7.36–7.30 (m, 2H), 7.29–7.25 (m, 1H), 5.73–5.69 (m, 1H), 2.84–2.76 (m, 1H), 2.67–2.59 (m, 4H), 2.06–1.93 (m, 2H), 1.90–1.81 (m, 1H), 1.80–1.73 (m, 1H). HRMS (ESI^+^) calcd for C_24_H_23_N_4_O_5_ (M + H)^+^ 447.1663 found 447.1667.
*5-((7-(4-Methyl-3-nitrobenzamido)-1,2,3,4-tetrahydronaphthalen-1-yl)oxy)nicotinamide* (**18**). White solid, 15%. ^1^H NMR (DMSO-*d*_6_, 600 MHz) δ 10.41 (s, 1H), 8.66 (s, 1H), 8.55 (s, 1H), 8.51 (s, 1H), 8.18 (d, *J* = 8.4 Hz, 1H), 8.15 (s, 1H), 7.94 (s, 1H), 7.75–7.00 (m, 2H), 7.66 (d, *J* = 7.2 Hz, 1H), 7.63 (s, 1H), 7.19 (d, *J* = 8.4 Hz, 1H), 5.68–5.64 (m, 1H), 2.86–2.79 (m, 1H), 2.76–2.68 (m, 1H), 2.58 (s, 3H), 2.07–1.95 (m, 2H), 1.92–1.83 (m, 1H), 1.82–1.74 (m, 1H). HRMS (ESI^+^) calcd for C_23_H_21_ClN_3_O_3_ (M + H)^+^ 447.1663 found 447.1671.
*(S)-5-((4-(Isonicotinamido)-2,3-dihydro-1H-inden-1-yl)oxy)nicotinamide* (**19a**). White solid, 13%. ^1^H NMR (DMSO-*d*_6_, 600 MHz) δ 10.32 (s, 1H), 8.80 (d, *J* = 5.4 Hz, 2H), 8.67 (s, 1H), 8.49 (d, *J* = 2.4 Hz, 1H), 8.16 (s, 1H), 7.91 (s, 1H), 7.88 (d, *J* = 4.8 Hz, 2H), 7.63 (s, 1H), 7.51–7.47 (m, 1H), 7.35–7.30 (m, 2H), 6.06 (dd, *J* = 3.6, 6.0 Hz, 1H), 3.08–3.01 (m, 1H), 2.93–2.85 (m, 1H), 2.65–2.56 (m, 1H), 2.11–2.03 (m, 1H). HRMS (ESI^+^) calcd for C_21_H_19_N_4_O_3_ (M + H)^+^ 375.1452 found 375.1460.
*(S)-5-((4-(4-Morpholinobenzamido)-2,3-dihydro-1H-inden-1-yl)oxy)nicotinamide* (**19b**). White solid, 5%. ^1^H NMR (CD_3_OD, 600 MHz) δ 8.65 (s, 1H), 8.45 (s, 1H), 7.97 (s, 1H), 7.89 (d, *J* = 8.4 Hz, 2H), 7.44 (d, *J* = 7.2 Hz,1H), 7.34–7.26 (m, 2H), 7.03 (d, *J* = 8.4 Hz, 2H), 6.04–5.99 (m, 1H), 3.88–3.78 (m, 4H), 3.35–3.25 (m, 4H), 3.16–3.08 (m, 1H), 3.00–2.92 (m, 1H), 2.72–2.62 (m, 1H), 2.24–2.16 (m, 1H). HRMS (ESI^+^) calcd for C_26_H_27_N_4_O_4_ (M + H)^+^ 459.2027 found 459.2031.
*(S)-5-((4-([1,1′-Biphenyl]-4-ylcarboxamido)-2,3-dihydro-1H-inden-1-yl)oxy)nicotinamide* (**19c**). White solid, 28%. ^1^H NMR (DMSO-*d*_6_, 600 MHz) δ 10.10 (s, 1H), 8.67 (s, 1H), 8.50 (d, *J* = 1.8 Hz, 1H), 8.17 (s, 1H), 8.08 (d, *J* = 8.4 Hz, 2H), 7.92 (s, 1H), 7.84 (d, *J* = 7.8 Hz, 2H), 7.76 (d, *J* = 7.8 Hz, 2H), 7.63 (s, 1H), 7.55–7.47 (m, 3H), 7.43 (dd, *J* = 7.2, 7.2 Hz, 1H), 7.34–7.28 (m, 2H), 6.08–6.04 (m, 1H), 3.10–3.02 (m, 1H), 2.95–2.88 (m, 1H), 2.65–2.56 (m, 1H), 2.11–2.03 (m, 1H). HRMS (ESI^+^) calcd for C_28_H_24_N_3_O_3_ (M + H)^+^ 450.1812 found 450.1812.
*(R)-5-((4-(Isonicotinamido)-2,3-dihydro-1H-inden-1-yl)oxy)nicotinamide* (**20a**). White solid, 10%. ^1^H NMR (DMSO-*d*_6_, 600 MHz) δ 10.33 (s, 1H), 8.80 (d, *J* = 4.2 Hz, 2H), 8.67 (s, 1H), 8.49 (s, 1H), 8.16 (s, 1H), 7.91 (s, 1H), 7.88 (d, *J* = 4.2 Hz, 2H), 7.63 (s, 1H), 7.49 (d, *J* = 4.8 Hz, 1H), 7.35–7.30 (m, 2H), 6.08–6.04 (m, 1H), 3.08–3.01 (m, 1H), 2.93–2.85 (m, 1H), 2.65–2.56 (m, 1H), 2.11–2.03 (m, 1H). HRMS (ESI^+^) calcd for C_21_H_19_N_4_O_3_ (M + H)^+^ 375.1452 found 375.1465.
*(R)-5-((4-(4-Morpholinobenzamido)-2,3-dihydro-1H-inden-1-yl)oxy)nicotinamide* (**20b**). White solid, 9%. ^1^H NMR (DMSO-*d*_6_, 600 MHz) δ 9.75 (s, 1H), 8.67 (s, 1H), 8.49 (s, 1H), 8.16 (s, 1H), 7.93–7.85 (m, 3H), 7.63 (s, 1H), 7.46 (d, *J* = 7.2 Hz, 1H), 7.29–7.23 (m, 2H), 7.03 (d, *J* = 9.0 Hz, 2H), 6.06–6.02 (m, 1H), 3.79–3.72 (m, 4H), 3.28–3.22 (m, 4H), 3.08–3.00 (m, 1H), 2.91–2.84 (m, 1H), 2.63–2.55 (m, 1H), 2.09–2.02 (m, 1H). HRMS (ESI^+^) calcd for C_26_H_27_N_4_O_4_ (M + H)^+^ 459.2027e found 459.2039.
*(R)-5-((4-([1,1′-Biphenyl]-4-carboxamido)-2,3-dihydro-1H-inden-1-yl)oxy)nicotinamide* (**20c**). White solid, 5%. ^1^H NMR (CD_3_OD, 600 MHz) δ 8.65 (s, 1H), 8.46 (s, 1H), 8.06 (d, *J* = 7.8 Hz, 2H), 7.99 (s, 1H), 7.79 (d, *J* = 8.4 Hz, 2H), 7.70 (d, *J* = 8.4 Hz, 2H), 7.52–7.46 (m, 3H), 7.42–7.39 (m, 1H), 7.38–7.32 (m, 2H), 6.06–6.02 (m, 1H), 3.20–3.13 (m, 1H), 3.03–2.96 (m, 1H), 2.74–2.66 (m, 1H), 2.26–2.19 (m, 1H). HRMS (ESI^+^) calcd for C_28_H_24_N_3_O_3_ (M + H)^+^ 450.1812 found 450.1819.
*(R)-5-((6-(Isonicotinamido)-2,3-dihydro-1H-inden-1-yl)oxy)nicotinamide* (**21a**). White solid, 4%. ^1^H NMR (DMSO-*d*_6_, 600 MHz) δ 10.51 (s, 1H), 8.77 (d, *J* = 6.0 Hz, 2H), 8.67 (s, 1H), 8.50 (s, 1H), 8.16 (s, 1H), 7.90 (s, 1H), 7.88–7.82 (m, 3H), 7.73 (d, *J* = 8.4 Hz, 1H), 7.63 (s, 1H), 7.35 (d, *J* = 8.4 Hz, 1H), 6.06–6.01 (m, 1H), 3.08–3.01 (m, 1H), 2.94–2.86 (m, 1H), 2.68–2.62 (m, 1H), 2.10–2.03 (m, 1H). HRMS (ESI^+^) calcd for C_21_H_19_N_4_O_3_ (M + H)^+^ 375.1452 found 375.1461.
*(R)-5-((6-(4-Morpholinobenzamido)-2,3-dihydro-1H-inden-1-yl)oxy)nicotinamide* (**21b**). White solid, 27%. ^1^H NMR (DMSO-*d*_6_, 600 MHz) δ 9.98 (s, 1H), 8.67 (s, 1H), 8.49 (s, 1H), 8.16 (s, 1H), 7.93–7.83 (m, 4H), 7.72 (d, *J* = 8.4 Hz, 1H), 7.63 (s, 1H), 7.29 (d, *J* = 7.8 Hz, 1H), 7.01 (d, *J* = 7.8 Hz, 2H), 6.03–5.98 (m, 1H), 3.78–3.71 (m, 4H), 3.27–3.20 (m, 4H), 3.06–2.98 (m, 1H), 2.92–2.83 (m, 1H), 2.68–2.60 (m, 1H), 2.10–2.02 (m, 1H). HRMS (ESI^+^) calcd for C_26_H_27_N_4_O_4_ (M + H)^+^ 459.2027 found 459.2034.
*(R)-5-((6-([1,1′-Biphenyl]-4-ylcarboxamido)-2,3-dihydro-1H-inden-1-yl)oxy)nicotinamide* (**21c**). White solid, 30%. ^1^H NMR (DMSO-*d*_6_, 600 MHz) δ 10.32 (s, 1H), 8.68 (s, 1H), 8.51 (s, 1H), 8.16 (s, 1H), 8.05 (d, *J* = 7.8 Hz, 2H), 7.91 (s, 2H), 7.83 (d, *J* = 8.4 Hz, 2H), 7.78–7.73 (m, 3H), 7.63 (s, 1H), 7.51 (dd, *J* = 7.2, 7.2 Hz, 2H), 7.45–7.40 (m, 1H), 7.34 (d, *J* = 7.8 Hz, 1H), 6.06–6.02 (m, 1H), 3.08–3.01 (m, 1H), 2.93–2.85 (m, 1H), 2.70–2.62 (m, 1H), 2.11–2.03 (m, 1H). HRMS (ESI^+^) calcd for C_28_H_24_N_3_O_3_ (M + H)^+^ 450.1812 found 450.1820.
*(S)-5-((6-(Isonicotinamido)-2,3-dihydro-1H-inden-1-yl)oxy)nicotinamide* (**22a**). White solid, 9%. ^1^H NMR (CD_3_OD, 600 MHz) δ 8.72 (d, *J* = 5.4 Hz, 2H), 8.65 (s, 1H), 8.45 (s, 1H), 7.99–7.96 (m, 1H), 7.87 (d, *J* = 5.4 Hz, 2H), 7.83 (s, 1H), 7.68–7.64 (m, 1H), 7.35 (d, *J* = 8.4 Hz, 1H), 6.01–5.97 (m, 1H), 3.17–3.10 (m, 1H), 3.00–2.93 (m, 1H), 2.74–2.67 (m, 1H), 2.26–2.19 (m, 1H). HRMS (ESI^+^) calcd for C_21_H_19_N_4_O_3_ (M + H)^+^ 375.1452 found 375.1465.
*(S)-5-((6-(4-Morpholinobenzamido)-2,3-dihydro-1H-inden-1-yl)oxy)nicotinamide* (**22b**). White solid, 7%. ^1^H NMR (DMSO-*d*_6_, 600 MHz) δ 9.98 (s, 1H), 8.67 (s, 1H), 8.49 (s, 1H), 8.16 (s, 1H), 7.93–7.83 (m, 4H), 7.72 (d, *J* = 8.4 Hz, 1H), 7.63 (s, 1H), 7.29 (d, *J* = 7.8 Hz, 1H), 7.01 (d, *J* = 8.4 Hz, 2H), 6.03–5.98 (m, 1H), 3.78–3.70 (m, 4H), 3.28–3.21 (m, 4H), 3.06–2.98 (m, 1H), 2.92–2.83 (m, 1H), 2.68–2.60 (m, 1H), 2.10–2.02 (m, 1H). HRMS (ESI^+^) calcd for C_26_H_27_N_4_O_4_ (M + H)^+^ 459.2027 found 459.2037.
*(S)-5-((6-([1,1′-Biphenyl]-4-ylcarboxamido)-2,3-dihydro-1H-inden-1-yl)oxy)nicotinamide* (**22c**). White solid, 25%. ^1^H NMR (DMSO-*d*_6_, 600 MHz) δ 10.32 (s, 1H), 8.67 (s, 1H), 8.51 (s, 1H), 8.17 (s, 1H), 8.05 (d, *J* = 8.4 Hz, 2H), 7.91 (s, 2H), 7.83 (d, *J* = 8.4 Hz, 2H), 7.78–7.73 (m, 3H), 7.63 (s, 1H), 7.51 (dd, *J* = 7.5, 7.5 Hz, 2H), 7.45–7.41 (m, 1H), 7.34 (d, *J* = 7.2 Hz, 1H), 6.06–6.02 (m, 1H), 3.08–3.01 (m, 1H), 2.93–2.85 (m, 1H), 2.70–2.62 (m, 1H), 2.11–2.03 (m, 1H). HRMS (ESI^+^) calcd for C_28_H_24_N_3_O_3_ (M + H)^+^ 450.1812 found 450.1820.
*4-Nitro-2,3-dihydro-1H-inden-1-one (23a) and 6-Nitro-2,3-dihydro-1H-inden-1-one* (**23c**) [32]. To a solution of 1-indanone (20 g, 151 mmol) in concentrated H_2_SO_4_ (210 mL) at 0 °C was added KNO_3_ (15.3 g, 151 mmol) in several portions. The reaction mixture was allowed to stir for 1 h and poured over ice (1 L). The mixture was extracted with EtOAc, and the organic phase was washed with brine and dried over Na_2_SO_4_. After filtration and removal of the solvent, the residue was purified by flash column chromatography (EtOAc/hexanes) to afford **23a** as a yellow solid (3.00 g, 23%) and **23c** as a yellow solid (4.00 g, 30%). ^1^H NMR data were consistent with those reported [32].
*5-Nitro-3,4-dihydronaphthalen-1(2H)-one (23b) and 7-Nitro-3,4-dihydronaphthalen-1(2H)-one* (**23d**). In a manner similar to that described for the preparation of compounds **23a** and **23c**, tetralone (5.53g, 37.8 mmol) was subjected to a nitration reaction to afford **23b** as a yellow solid (1.48 g, 20%). ^1^H NMR (DMSO-*d*_6_, 600 MHz) δ 8.22 (d, *J* = 7.8 Hz, 1H), 8.19 (d, *J* = 8.4 Hz, 1H), 7.61 (dd, *J* = 7.5, 7.5 Hz, 1H), 3.09 (t, *J* = 6.3 Hz, 2H), 2.68 (t, *J* = 6.9 Hz, 2H), 2.06 (p, *J* = 6.3 Hz, 2H). HRMS (ESI^−^) calcd for C_10_H_8_NO_3_ (M − H)^-^ 190.0510, found 190.0505 and **23d** as a yellow solid (4.00 g, 55%). ^1^H NMR (DMSO-*d*_6_, 600 MHz) δ 8.54 (s, 1H), 8.36 (d, *J* = 8.4 Hz, 1H), 7.67 (d, *J* = 8.4 Hz, 1H), 3.08 (t, *J* = 5.7 Hz, 2H), 2.70 (t, *J* = 6.6 Hz, 2H), 2.09 (p, *J* = 6.3 Hz, 2H). HRMS (ESI^−^) calcd for C_10_H_8_NO_3_ (M − H)^−^ 190.0510 found 190.0511.
*4-Nitro-2,3-dihydro-1H-inden-1-ol* (**24a**). To a solution of **23a** (1.50 g, 8.47 mmol) in MeOH was added NaBH_4_ (641 mg, 16.9 mmol) in several portions. The reaction mixture was allowed to stir at rt for 2 h. After the reaction was quenched with 1 N HCl, the aqueous solution was extracted with EtOAc and the organic phase washed with brine and dried over Na_2_SO_4_. After filtration and removal of the solvent, the residue was purified by flash column chromatography (EtOAc/hexanes) to afford **24a** as a light yellow solid (1.40 g, 92%). ^1^H NMR (CDCl_3_, 600 MHz) δ 8.13 (d, *J* = 8.4 Hz, 1H), 7.73 (d, *J* = 7.2 Hz, 1H), 7.44 (dd, *J* = 7.5, 7.5 Hz, 1H), 5.33 (d, *J* = 6.6 Hz, 1H), 3.60–3.54 (m, 1H), 3.32–3.26 (m, 1H), 2.63–2.56 (m, 1H), 2.06–2.00 (m, 1H). HRMS (ESI^−^) calcd for C_9_H_8_NO_3_ (M − H)^−^ 178.0510 found 178.0513.
*5-Nitro-1,2,3,4-tetrahydronaphthalen-1-ol* (**24b**). In a manner similar to that described for the preparation of compound **24a**, **23b** (1.35 g, 7.06 mmol) was reduced with NaBH_4_ to afford **24b** as a light yellow solid (1.25 g, 92%). ^1^H NMR (DMSO-*d*_6_, 600 MHz) δ 8.26 (s, 1H), 7.99 (d, *J* = 8.4 Hz, 1H), 7.35 (d, *J* = 8.4 Hz, 1H), 5.55 (d, *J* = 6.0 Hz, 1H), 4.66–4.61 (m, 1H), 2.88–2.75 (m, 2H), 2.01–1.94 (m, 1H), 1.93–1.85 (m, 1H), 1.76–1.62 (m, 2H). HRMS (ESI^−^) calcd for C_10_H_10_NO_3_ (M − H)^−^ 192.0666 found 192.0659.
*(R)-4-Nitro-2,3-dihydro-1H-inden-1-ol* (**24c**). To a solution of compound **23a** (1.77 g, 10.0 mmol) and (*S*)-CBS (139 mg, 0.50 mmol) in CH_2_Cl_2_ (30 mL) at –20 °C was slowly added BH_3_^.^SMe_2_ (1.00 mL, 10.0 mmol), and the mixture was stirred at this temperature for 1 h. After the reaction was quenched with saturated NH_4_Cl (20 mL) and stirred at rt for 3 h, the mixture was extracted with EtOAc. The organic phase was washed with water and brine, dried over anhydrous Na_2_SO_4,_ and concentrated in vacuo. The residue was purified by flash column chromatography (EtOAc/hexanes) to afford compound **24c** as a white solid (1.61 g, 90%). [α]_D_^20^ = +51.4 (c = 0.43, MeOH). ^1^H NMR (CDCl_3_, 600 MHz) δ 8.12 (d, *J* = 8.4 Hz, 1H), 7.72 (d, *J* = 7.2 Hz, 1H), 7.43 (dd, *J* = 7.5, 7.5 Hz, 1H), 5.32 (dd, *J* = 6.6, 6.6 Hz, 1H), 3.60–3.52 (m, 1H), 3.32–3.24 (m, 1H), 2.63–2.56 (m, 1H), 2.06–2.00 (m, 1H). HRMS (ESI^−^) calcd for C_9_H_8_NO_3_ (M − H)^−^ 178.0510 found 178.0512.
*(S)-4-Nitro-2,3-dihydro-1H-inden-1-ol* (**24d**). In a manner similar to that described for the preparation of compound **24c**, **23a** (1.00 g, 5.65 mmol) was reduced with BH_3_^.^SMe_2_ (0.57 mL, 5.65 mmol) in the presence of (*R*)-CBS (78 mg, 0.28 mmol) to give **24d** as a light yellow solid (900 mg, 89%). [α]_D_^20^ = –47.3 (c = 0.75, MeOH). ^1^H NMR (CDCl_3_, 600 MHz) δ 8.13 (d, *J* = 8.4 Hz, 1H), 7.73(d, *J* = 7.2 Hz, 1H), 7.44 (dd, *J* = 7.5, 7.5 Hz, 1H), 5.33 (dd, *J* = 6.6, 6.6 Hz, 1H), 3.60–3.52 (m, 1H), 3.32–3.24 (m, 1H), 2.63–2.56 (m, 1H), 2.06–2.00 (m, 1H). HRMS (ESI^−^) calcd for C_9_H_8_NO_3_ (M − H)^−^ 178.0510 found 178.0513.
*6-Nitro-2,3-dihydro-1H-inden-1-ol* (**24e**). In a manner similar to that described for the preparation of compound **24a**, **23c** (2.50 g, 14.1 mmol) was reduced with NaBH_4_ (1.07 g, 28.2 mmol) in MeOH to give **24e** as a light yellow solid (2.40 g, 95%). ^1^H NMR (CDCl_3_, 600 MHz) δ 8.26–8.22 (m, 1H), 8.14–8.10 (m, 1H), 7.39–7.35 (m, 1H), 5.34–5.28 (m, 1H), 3.16–3.09 (m, 1H), 2.94–2.86 (m, 1H), 2.63–2.56 (m, 1H), 2.09–2.01 (m, 1H). HRMS (ESI^−^) calcd for C_9_H_8_NO_3_ (M − H)^−^ 178.0510 found 178.0512.
*7-Nitro-1,2,3,4-tetrahydronaphthalen-1-ol* (**24f**). In a manner similar to that described for the preparation of compound **24a**, **23d** (1.50 g, 7.85 mmol) was reduced with NaBH_4_ to give **24f** as a white solid (1.40 g, 92%). ^1^H NMR (DMSO-*d*_6_, 600 MHz) δ 7.76 (d, *J* = 7.8 Hz, 2H), 7.42 (dd, *J* = 7.8, 7.8 Hz, 1H), 5.43 (d, *J* = 5.4 Hz, 1H), 4.65–4.60 (m, 1H), 2.89–2.82 (m, 1H), 2.80–2.73 (m, 1H), 1.96–1.84 (m, 2H), 1.74–1.64 (m, 2H). HRMS (ESI^−^) calcd for C_10_H_10_NO_3_ (M − H)^−^ 192.0666 found 192.0669.
*(S)-6-Nitro-2,3-dihydro-1H-inden-1-ol* (**24g**). In a manner similar to that described for the preparation of compound **24c**, **23c** (1.00 g, 5.65 mmol) was reduced with BH_3_^.^SMe_2_ (0.57 mL, 5.65 mmol) in the presence of (*R*)-CBS (78 mg, 0.28 mmol) to give **24g** as a light yellow solid (900 mg, 89%). [α]_D_^20^ = +46.1 (c = 0.42, MeOH). ^1^H NMR (CDCl_3_, 600 MHz) δ 8.26–8.22 (m, 1H), 8.14 (dd, *J* = 8.4, 2.4 Hz, 1H), 7.37 (d, *J* = 7.8 Hz, 1H), 5.32 (dd, *J* = 6.6, 6.6 Hz, 1H), 3.16–3.09 (m, 1H), 2.94–2.86 (m, 1H), 2.64–2.58 (m, 1H), 2.09–2.01 (m, 1H). HRMS (ESI^−^) calcd for C_9_H_8_NO_3_ (M − H)^−^ 178.0510 found 178.0513.
*(R)-6-Nitro-2,3-dihydro-1H-inden-1-ol* (**24h**): In a manner similar to that described for the preparation of compound **24c**, **23c** (1.00 g, 5.65 mmol) was reduced with BH_3_^.^SMe_2_ (0.57 mL, 5.65 mmol) in the presence of (*S*)-CBS (78 mg, 0.28 mmol) in CH_2_Cl_2_ to give **24h** (850 mg, 84%) as a light yellow solid. [α]_D_^20^ = –46.6 (c = 0.70, MeOH). ^1^H NMR (CDCl_3_, 600 MHz) δ 8.26–8.22 (m, 1H), 8.14 (dd, *J* = 8.4, 2.4 Hz, 1H), 7.37 (d, *J* = 7.8 Hz, 1H), 5.32 (dd, *J* = 6.6, 6.6 Hz, 1H), 3.16–3.09 (m, 1H), 2.94–2.86 (m, 1H), 2.64–2.58 (m, 1H), 2.09–2.01 (m, 1H). HRMS (ESI^−^) calcd for C_9_H_8_NO_3_ (M − H)^−^ 178.0510 found 178.0514.
*Methyl 5-((4-Nitro-2,3-dihydro-1H-inden-1-yl)oxy)nicotinate* (**25a**). To a solution of **24a** (1.28 g, 7.16 mmol), methyl 5-hydroxynicotinate (1.32 g, 8.59 mmol), and Ph_3_P (2.82 g, 10.7 mmol) in anhydrous THF (45 mL) at rt was added DIAD (2.17 g, 10.7 mmol) dropwise. After the mixture was stirred for 12 h and the solvent removed, the residue was purified by flash column chromatography (EtOAc/hexanes) to afford **25a** as a yellow solid (1.70 g, 76%). ^1^H NMR (DMSO-*d*_6_, 600 MHz) δ 8.74 (s, 1H), 8.61 (d, *J* = 3.0 Hz, 1H), 8.20 (d, *J* = 7.8 Hz, 1H), 7.93 (s, 1H), 7.88 (d, *J* = 6.6 Hz, 1H), 7.59 (dd, *J* = 7.8, 7.8 Hz, 1H), 6.17 (dd, *J* = 6.6, 4.2 Hz, 1H), 3.91 (s, 3H), 3.52–3.45 (m, 1H), 3.40–3.34 (m, 1H), 2.71–2.64 (m, 1H), 2.17–2.10 (m, 1H). HRMS (ESI^+^) calcd for C_16_H_15_N_2_O_5_ (M + H)^+^ 315.0975 found 315.0983.
*Methyl 5-((5-Nitro-1,2,3,4-tetrahydronaphthalen-1-yl)oxy)nicotinate* (**25b**). In a manner similar to that described for the preparation of compound **25a**, **24b** (468 mg, 2.42 mmol) and methyl 5-hydroxynicotinate (371 mg, 2.42 mmol) were treated with DIAD and Ph_3_P to afford **25b** as a yellow solid (600 mg, 75%). ^1^H NMR (CDCl_3_, 600 MHz) δ 8.88 (s, 1H), 8.53 (d, *J* = 3.0 Hz, 1H), 7.91 (s, 1H), 7.88 (d, *J* = 8.4 Hz, 1H), 7.61 (d, *J* = 7.8 Hz, 1H), 7.38 (dd, *J* = 8.1, 8.1 Hz, 1H), 5.50 (dd, *J* = 4.8, 4.8 Hz, 1H), 3.98 (s, 3H), 3.17–3.11 (m, 1H), 3.04–2.96 (m, 1H), 2.22–2.15 (m, 1H), 2.12–1.98 (m, 2H), 1.93–1.86 (m, 1H). HRMS (ESI^+^) calcd for C_17_H_17_N_2_O_5_ (M + H)^+^ 329.1132 found 329.1139.
*(S)-Methyl 5-((4-Nitro-2,3-dihydro-1H-inden-1-yl)oxy)nicotinate* (**25c**). In a manner similar to that described for the preparation of compound **25a**, **24c** (500 mg, 2.79 mmol) and methyl 5-hydroxynicotinate (513 g, 3.35 mmol) were treated with DIAD and Ph_3_P to afford **25c** as a yellow solid (580 g, 66%). ^1^H NMR (CDCl_3_, 600 MHz) δ 8.84 (s, 1H), 8.52 (d, *J* = 2.4 Hz, 1H), 7.90 (s, 1H), 7.11 (dd, *J* = 7.8, 7.8 Hz, 1H), 6.85 (d, *J* = 7.8 Hz, 1H), 6.68 (d, *J* = 7.2 Hz, 1H), 5.82 (dd, *J* = 6.6, 3.6 Hz, 1H), 3.96 (s, 3H), 3.00–2.92 (m, 1H), 2.80–2.73 (m, 1H), 2.66–2.60 (m, 1H), 2.28–2.22 (m, 1H). HRMS (ESI^+^) calcd for C_16_H_15_N_2_O_5_ (M + H)^+^ 315.0975 found 315.0984.
*(R)-Methyl 5-((4-Nitro-2,3-dihydro-1H-inden-1-yl)oxy)nicotinate* (**25d**). In a manner similar to that described for the preparation of compound **25a**, **24d** (500 mg, 2.79 mmol) and methyl 5-hydroxynicotinate (513 g, 3.35 mmol) were treated with DIAD and Ph_3_P to afford **25d** as a yellow solid (560 g, 64%). ^1^H NMR (CDCl_3_, 600 MHz) δ 8.84 (s, 1H), 8.52 (s, 1H), 7.90 (s, 1H), 7.11 (dd, *J* = 7.8, 7.8 Hz, 1H), 6.85 (d, *J* = 7.8 Hz, 1H), 6.68 (d, *J* = 7.2 Hz, 1H), 5.82 (dd, *J* = 6.6, 3.6 Hz, 1H), 3.96 (s, 3H), 3.00–2.92 (m, 1H), 2.80–2.73 (m, 1H), 2.66–2.60 (m, 1H), 2.28–2.22 (m, 1H). HRMS (ESI^+^) calcd for C_16_H_15_N_2_O_5_ (M + H)^+^ 315.0975 found 315.0985.
*Methyl 5-((6-Nitro-2,3-dihydro-1H-inden-1-yl)oxy)nicotinate* (**25e**). In a manner similar to that described for the preparation of compound **25a**, **25e** was prepared from **24e** (1.14 g, 6.34 mmol) as a yellow solid (1.30 g, 65%). ^1^H NMR (CDCl_3_, 600 MHz) δ 8.89 (s, 1H), 8.54 (d, *J* = 3.0 Hz, 1H), 8.28 (s, 1H), 8.23 (dd, *J* = 7.8, 1.8 Hz, 1H), 7.91 (s, 1H), 7.48 (s, 1H), 5.91–5.88 (m, 1H), 3.98 (s, 3H), 3.29–3.23 (m, 1H), 3.11–3.05 (m, 1H), 2.79–2.73 (m, 1H), 2.34–2.28 (m, 1H). HRMS (ESI^+^) calcd for C_16_H_15_N_2_O_5_ (M + H)^+^ 315.0975 found 315.0988.
*Methyl 5-((7-Nitro-1,2,3,4-tetrahydronaphthalen-1-yl)oxy)nicotinate* (**25f**). In a manner similar to that described for the preparation of compound **25a**, **24f** (505 mg, 3.30 mmol) and methyl 5-hydroxynicotinate (638 mg, 3.30 mmol) were treated with DIAD and Ph_3_P to afford **25f** as a yellow solid (810 mg, 75%). ^1^H NMR (CDCl_3_, 600 MHz) δ 8.89 (s, 1H), 8.55 (d, *J* = 2.4 Hz, 1H), 8.24 (s, 1H), 8.12 (d, *J* = 9.0 Hz, 1H), 7.92 (s, 1H), 7.35 (d, *J* = 8.4, 1H), 5.51 (dd, *J* = 4.8, 4.8 Hz, 1H), 3.98 (s, 3H), 3.05–2.99 (m, 1H), 2.91–2.85 (m, 1H), 2.17–2.10 (m, 2H), 2.08–2.02 (m, 1H), 1.93–1.87 (m, 1H). HRMS (ESI^+^) calcd for C_17_H_17_N_2_O_5_ (M + H)^+^ 329.1132 found 329.1134.
*(R)-Methyl 5-((6-Nitro-2,3-dihydro-1H-inden-1-yl)oxy)nicotinate* (**25g**). In a manner similar to that described for the preparation of compound **25a**, **24g** (550 mg, 3.07 mmol) and methyl 5-hydroxynicotinate (554 g, 3.68 mmol) were treated with DIAD and Ph_3_P to afford **25g** as a yellow solid (629 g, 64%). ^1^H NMR (CDCl_3_, 600 MHz) δ 8.84 (s, 1H), 8.52 (d, *J* = 3.0 Hz, 1H), 7.89 (dd, *J* = 2.4, 2.4 Hz, 1H), 7.10 (d, *J* = 7.8 Hz, 1H), 6.74 (s, 1H), 6.69 (dd, *J* = 8.1, 1.8 Hz, 1H), 5.76 (dd, *J* = 6.0, 4.2 Hz, 1H), 3.97 (s, 3H), 3.08–3.02 (m, 1H), 2.88–2.82 (m, 1H), 2.63–2.56 (m, 1H), 2.22–2.14 (m, 1H). HRMS (ESI^+^) calcd for C_16_H_15_N_2_O_5_ (M + H)^+^ 315.0975 found 315.0981.
*(S)-Methyl 5-((6-Nitro-2,3-dihydro-1H-inden-1-yl)oxy)nicotinate* (**25h**). In a manner similar to that described for the preparation of compound **25a**, **24h** (700 mg, 3.91 mmol) and methyl 5-hydroxynicotinate (718 g, 4.69 mmol) were treated with DIAD and Ph_3_P to afford **25h** as a yellow solid (800 g, 65%). ^1^H NMR (CDCl_3_, 600 MHz) δ 8.84 (s, 1H), 8.52 (d, *J* = 2.4 Hz, 1H), 7.89 (s, 1H), 7.10 (d, *J* = 7.8 Hz, 1H), 6.74 (s, 1H), 6.69 (d, *J* = 8.4 Hz, 1H), 5.88–5.74 (m, 1H), 3.97 (s, 3H), 3.08–3.02 (m, 1H), 2.88–2.82 (m, 1H), 2.63–2.56 (m, 1H), 2.22–2.14 (m, 1H). HRMS (ESI^+^) calcd for C_16_H_15_N_2_O_5_ (M + H)^+^ 315.0975 found 315.0986.
*Methyl 5-((4-Amino-2,3-dihydro-1H-inden-1-yl)oxy)nicotinate* (**26a**). To a solution of compound **25a** (500 mg, 1.60 mmol) and NiCl_2_·6H_2_O (760 mg, 4.48 mmol) in MeOH (800 mL) was slowly added NaBH_4_ (250 mg, 6.40 mmol), and the mixture was stirred at rt for 2 h. The reaction was quenched with saturated NH_4_Cl (50 mL) and extracted with EtOAc. The organic phase was washed with water and brine, dried over anhydrous K_2_CO_3,_ and concentrated in vacuo. The residue was purified by flash column chromatography (EtOAc/hexanes) to afford compound **26a** as a white solid (290 mg, 64%). ^1^H NMR (DMSO-*d*_6_, 600 MHz) δ 8.70 (s, 1H), 8.56 (d, *J* = 3.0 Hz, 1H), 7.88–7.86 (m, 1H), 6.93 (dd, *J* = 7.8, 7.8 Hz, 1H), 6.58 (d, *J* = 7.2 Hz, 1H), 6.56 (d, *J* = 8.4, 1H), 5.95 (dd, *J* = 6.6, 3.3 Hz, 1H), 5.02 (s, 2H), 3.90 (s, 3H), 2.82–2.77 (m, 1H), 2.69–2.64 (m, 1H), 2.57–2.51 (m, 1H), 2.05–2.00 (m, 1H). HRMS (ESI^+^) calcd for C_16_H_17_N_2_O_3_ (M + H)^+^ 285.1234 found 285.1241.
*Methyl 5-((5-Amino-1,2,3,4-tetrahydronaphthalen-1-yl)oxy)nicotinate* (**26b**). In a manner similar to that described for the preparation of compound **26a**, **25b** (737 mg, 2.24 mmol) was reduced to afford **26b** as a light yellow solid (620 mg, 94%). ^1^H NMR (CDCl_3_, 600 MHz) δ 8.84 (s, 1H), 8.53 (s, 1H), 7.91 (s, 1H), 7.06 (dd, *J* = 7.8, 7.8 Hz, 1H), 6.78 (d, *J* = 7.8 Hz, 1H), 6.68 (d, *J* = 8.4 Hz, 1H), 5.45–5.40 (m, 1H), 3.96 (s, 3H), 3.68 (bs, 2H), 2.64–2.57 (m, 1H), 2.49–2.41 (m, 1H), 2.19–2.06 (m, 2H), 2.03–1.95 (m, 1H), 1.94–1.86 (m, 1H). HRMS (ESI^+^) calcd for C_17_H_19_N_2_O_3_ (M + H)^+^ 299.1390 found 299.1400.
*(S)-Methyl 5-((4-Amino-2,3-dihydro-1H-inden-1-yl)oxy)nicotinate* (**26c**). In a manner similar to that described for the preparation of compound **26a**, **25c** (250 mg, 0.80 mmol) was reduced to afford **26c** as a light yellow solid (180 mg, 80%). ^1^H NMR (CDCl_3_, 600 MHz) δ 8.84 (s, 1H), 8.52 (d, *J* = 2.4 Hz, 1H), 7.90 (dd, *J* = 1.8, 1.8 Hz, 1H), 7.11 (dd, *J* = 7.8, 7.8 Hz, 1H), 6.85 (d, *J* = 7.8 Hz, 1H), 6.68 (d, *J* = 7.8 Hz, 1H), 5.83 (dd, *J* = 6.0, 3.6 Hz, 1H), 3.96 (s, 3H), 3.00–2.92 (m, 1H), 2.80–2.73 (m, 1H), 2.67–2.60 (m, 1H), 2.28–2.22 (m, 1H). HRMS (ESI^+^) calcd for C_16_H_17_N_2_O_3_ (M + H)^+^ 285.1234 found 285.1237.
*(R)-Methyl 5-((4-Amino-2,3-dihydro-1H-inden-1-yl)oxy)nicotinate* (**26d**). In a manner similar to that described for the preparation of compound **26a**, **25d** (250 mg, 0.80 mmol) was reduced to afford **26d** as a light yellow solid (160 mg, 70%). ^1^H NMR (CDCl_3_, 600 MHz) δ 8.84 (s, 1H), 8.52 (s, 1H), 7.90 (s, 1H), 7.11 (dd, *J* = 7.8, 7.8 Hz, 1H), 6.85 (d, *J* = 7.2 Hz, 1H), 6.67 (d, *J* = 7.8 Hz, 1H), 5.84–5.80 (m, 1H), 3.96 (s, 3H), 3.00–2.92 (m, 1H), 2.80–2.73 (m, 1H), 2.67–2.59 (m, 1H), 2.29–2.22 (m, 1H). HRMS (ESI^+^) calcd for C_16_H_17_N_2_O_3_ (M + H)^+^ 285.1234 found 285.1241.
*Methyl 5-((6-Amino-2,3-dihydro-1H-inden-1-yl)oxy)nicotinate* (**26e**). In a manner similar to that described for the preparation of compound **26a**, **25e** (500 mg, 1.60 mmol) was reduced to afford **26e** as a light yellow solid (270 mg, 59%). ^1^H NMR (CDCl_3_, 600 MHz) δ 8.84 (d, *J* = 1.8 Hz, 1H), 8.53 (d, *J* = 3.0 Hz, 1H), 7.89 (dd, *J* = 3.0, 1.8 Hz, 1H), 7.10 (d, *J* = 7.8 Hz, 1H), 6.74 (d, *J* = 2.4 Hz, 1H), 6.69 (dd, *J* = 8.4, 2.4 Hz, 1H), 5.76 (dd, *J* = 6.6, 4.2 Hz, 1H), 3.97 (s, 3H), 3.65 (bs, 2H), 3.08–3.02 (m, 1H), 2.88–2.82 (m, 1H), 2.63–2.56 (m, 1H), 2.02–1.95 (m, 1H). HRMS (ESI^+^) calcd for C_16_H_17_N_2_O_3_ (M + H)^+^ 285.1234 found 285.1243.
*Methyl 5-((7-Amino-1,2,3,4-tetrahydronaphthalen-1-yl)oxy)nicotinate* (**26f**). In a manner similar to that described for the preparation of compound **26a**, **25f** (682 mg, 2.08 mmol) was reduced to afford **26f** as a white solid (580 mg, 94%). ^1^H NMR (CDCl_3_, 600 MHz) δ 8.83 (s, 1H), 8.53 (s, 1H), 7.90 (s, 1H), 6.96 (d, *J* = 8.4 Hz, 1H), 6.66–6.61 (m, 2H), 5.38–5.34 (m, 1H), 3.96 (s, 3H), 3.60 (bs, 2H), 2.81–2.75 (m, 1H), 2.71–2.63 (m, 1H), 2.12–2.05 (m, 1H), 2.04–1.92 (m, 2H), 1.81–1.74 (m, 1H). HRMS (ESI^+^) calcd for C_17_H_19_N_2_O_3_ (M + H)^+^ 299.1390 found 299.1390.
*(R)-Methyl 5-((6-Amino-2,3-dihydro-1H-inden-1-yl)oxy)nicotinate* (**26g**). In a manner similar to that described for the preparation of compound **26a**, **25g** (314 mg, 1.00 mmol) was reduced to afford **26g** as a yellow solid (200 mg, 70%). ^1^H NMR (CDCl_3_, 600 MHz) δ 8.84 (s, 1H), 8.52 (d, *J* = 2.4 Hz, 1H), 7.89 (s, 1H), 7.10 (d, *J* = 7.8 Hz, 1H), 6.74 (s, 1H), 6.69 (d, *J* = 8.4 Hz, 1H), 5.77–5.74 (m, 1H), 3.96 (s, 3H), 3.08–3.01 (m, 1H), 2.88–2.81 (m, 1H), 2.64–2.56 (m, 1H), 2.22–2.14 (m, 1H). HRMS (ESI^+^) calcd for C_16_H_17_N_2_O_3_ (M + H)^+^ 285.1234 found285.1239.
*(S)-Methyl 5-((6-Amino-2,3-dihydro-1H-inden-1-yl)oxy)nicotinate* (**26h**). In a manner similar to that described for the preparation of compound **25a**, **25h** (314 mg, 1.00 mmol) was reduced to afford **26h** as a yellow solid (220 mg, 77%). ^1^H NMR (CDCl_3_, 600 MHz) δ 8.84 (d, *J* = 1.2 Hz, 1H), 8.52 (d, *J* = 3.0 Hz, 1H), 7.89 (dd, *J* = 2.4, 2.4 Hz, 1H), 7.10 (d, *J* = 7.8 Hz, 1H), 6.74 (d, *J* = 1.8 Hz, 1H), 6.69 (dd, *J* = 7.8, 1.8 Hz, 1H), 5.76 (dd, *J* = 6.0, 3.6 Hz, 1H), 3.97 (s, 3H), 3.07–3.02 (m, 1H), 2.88–2.82 (m, 1H), 2.63–2.56 (m, 1H), 2.21–2.14 (m, 1H). HRMS (ESI^+^) calcd for C_16_H_17_N_2_O_3_ (M + H)^+^ 285.1234 found 285.1239.
*5-((4-Amino-2,3-dihydro-1H-inden-1-yl)oxy)nicotinamide* (**27a**). A solution of methyl ester **26a** (280 mg, 0.98 mmol) in NH_3_/MeOH (~7 N, 10 mL) in a seal tube was heated at 70 °C for 24 h. After the solvent was evaporated in vacuo, the residue was dissolved in EtOAc (150 mL) and the organic layer was washed with H_2_O (150 mL) and then with brine (100 mL). After the organic layer was dried over Na_2_SO_4_ and filtered, the filtrate was concentrated and the residue was purified by flash column chromatography (5% MeOH/CH_2_Cl_2_) to afford compound **27a** as a light yellow solid (260 mg, 98%). ^1^H NMR (DMSO-*d*_6_, 600 MHz) δ 8.64 (s, 1H), 8.43 (d, *J* = 1.8 Hz, 1H), 8.14 (s, 1H), 7.86 (s, 1H), 7.61 (s, 1H), 6.93 (dd, *J* = 7.5, 7.5 Hz, 1H), 6.58 (d, *J* = 7.8, 1H), 6.50 (d, *J* = 7.2 Hz, 1H), 5.90 (dd, *J* = 6.6, 3.6 Hz, 1H), 5.02 (s, 2H), 2.82–2.77 (m, 1H), 2.68–2.63 (m, 1H), 2.58–2.52 (m, 1H), 2.04–2.00 (m, 1H). HRMS (ESI^+^) calcd for C_15_H_16_N_3_O_2_ (M + H)^+^ 270.1237 found 270.1247.
*5-((5-Amino-1,2,3,4-tetrahydronaphthalen-1-yl)oxy)nicotinamide* (**27b**). In a manner similar to that described for the preparation of compound **27a**, aminolysis of methyl ester **26b** (400 mg, 1.34 mmol) in the presence of CaCl_2_ (149 mg, 1.34 mmol) afforded **27b** as a white solid (320 mg, 84%). ^1^H NMR (CD_3_OD, 600 MHz) δ 8.62 (s, 1H), 8.40 (s, 1H), 7.95 (s, 1H), 6.95 (dd, *J* = 7.5, 7.5 Hz, 1H), 6.71 (d, *J* = 7.8 Hz, 1H), 6.68 (d, *J* = 7.2 Hz, 1H), 5.54–5.48 (m, 1H), 2.67–2.60 (m, 1H), 2.50–2.42 (m, 1H), 2.16–2.01 (m, 2H), 2.00–1.95 (m, 1H), 1.92–1.80 (m, 1H). HRMS (ESI^+^) calcd for C_16_H_18_N_3_O_2_ (M + H)^+^ 284.1394 found 284.1397.
*(S)-5-((4-Amino-2,3-dihydro-1H-inden-1-yl)oxy)nicotinamide* (**27c**). In a manner similar to that described for the preparation of compound **27a**, aminolysis of **26c** (180 mg, 0.63 mmol) afforded **27c** as a white solid (160 mg, 94%). ^1^H NMR (CDCl_3_, 600 MHz) δ 8.57 (d, *J* = 1.8 Hz, 1H), 8.50 (d, *J* = 3.0 Hz, 1H), 7.81 (dd, *J* = 2.4, 2.4 Hz, 1H), 7.11 (dd, *J* = 7.8 Hz, 1H), 6.86 (d, *J* = 7.8 Hz, 1H), 6.68 (d, *J* = 7.2 Hz, 1H), 5.84 (dd, *J* = 6.6, 3.6 Hz, 1H), 2.99–2.92 (m, 1H), 2.80–2.73 (m, 1H), 2.68–2.61 (m, 1H), 2.28–2.22 (m, 1H). HRMS (ESI^+^) calcd for C_15_H_16_N_3_O_2_ (M + H)^+^ 270.1237 found 270.1249.
*(R)-5-((4-Amino-2,3-dihydro-1H-inden-1-yl)oxy)nicotinamide* (**27d**). In a manner similar to that described for the preparation of compound **27a**, aminolysis of **26d** (160 mg, 0.56 mmol) afforded **27d** as a white solid (140 mg, 93%). ^1^H NMR (CDCl_3_, 600 MHz) δ 8.59 (d, *J* = 1.8 Hz, 1H), 8.48 (d, *J* = 3.0 Hz, 1H), 7.81 (dd, *J* = 2.4, 2.4 Hz, 1H), 7.11 (dd, *J* = 7.8 Hz, 1H), 6.85 (d, *J* = 7.8 Hz, 1H), 6.67 (d, *J* = 7.8 Hz, 1H), 5.83 (dd, *J* = 7.2, 3.6 Hz, 1H), 2.98–2.91 (m, 1H), 2.78–2.72 (m, 1H), 2.66–2.60 (m, 1H), 2.27–2.21 (m, 1H). HRMS (ESI^+^) calcd for C_15_H_16_N_3_O_2_ (M + H)^+^ 270.1237 found 270.1235.
*5-((6-Amino-2,3-dihydro-1H-inden-1-yl)oxy)nicotinamide* (**27e**). In a manner similar to that described for the preparation of compound **27a**, aminolysis of **26e** (250 mg, 0.88 mmol) afforded **27e** as a light yellow solid (230 mg, 97%). ^1^H NMR (DMSO-*d*_6_, 600 MHz) δ 8.65 (s, 1H), 8.45 (d, *J* = 2.4 Hz, 1H), 8.14 (s, 1H), 7.87 (s, 1H), 7.61 (s, 1H), 6.97 (d, *J* = 7.8 Hz, 1H), 6.59 (s, 1H), 6.56 (d, *J* = 7.2, 1H), 5.86 (dd, *J* = 5.1, 5.1 Hz, 1H), 4.96 (s, 2H), 2.90–2.85 (m, 1H), 2.75–2.69 (m, 1H), 2.57–2.50 (m, 1H), 2.00–1.95 (m, 1H). HRMS (ESI^+^) calcd for C_15_H_16_N_3_O_2_ (M + H)^+^ 270.1237 found 270.1237.
*5-((7-Amino-1,2,3,4-tetrahydronaphthalen-1-yl)oxy)nicotinamide* (**27f**). In a manner similar to that described for the preparation of compound **27a**, aminolysis of methyl ester **26f** (500 mg, 1.73 mmol) in the presence of CaCl_2_ (192 mg, 1.73 mmol) afforded **27f** as a white solid (410 mg, 85%). ^1^H NMR (DMSO-*d*_6_, 600 MHz) δ 8.64 (d, *J* = 1.8 Hz, 1H), 8.45 (d, *J* = 3.0 Hz, 1H), 8.14 (s, 1H), 7.89 (dd, *J* = 1.8, 1.8 Hz, 1H), 7.61 (s, 1H), 6.82 (d, *J* = 8.4 Hz, 1H), 6.52–6.47 (m, 2H), 5.49 (t, *J* = 4.8 Hz, 1H), 4.86 (s, 2H), 2.68–2.62 (m, 1H), 2.60–2.53 (m, 1H), 1.96–1.91 (m, 2H), 1.86–1.79 (m, 1H), 1.73–1.66 (m, 1H). HRMS (ESI^+^) calcd for C_16_H_18_N_3_O_2_ (M + H)^+^ 284.1394 found 284.1399.
*(R)-5-((6-Amino-2,3-dihydro-1H-inden-1-yl)oxy)nicotinamide* (**27g**). In a manner similar to that described for the preparation of compound **27a**, aminolysis of **26g** (220 mg, 0.77 mmol) afforded **27g** as a light yellow solid (180 mg, 86%). ^1^H NMR (CDCl_3_, 600 MHz) δ 8.57 (s, 1H), 8.50 (d, *J* = 3.0 Hz, 1H), 7.81 (s, 1H), 7.10 (d, *J* = 7.8 Hz, 1H), 6.74 (s, 1H), 6.69 (d, *J* = 8.4 Hz, 1H), 5.77 (dd, *J* = 4.8, 4.8 Hz, 1H), 3.08–3.01 (m, 1H), 2.88–2.82 (m, 1H), 2.63–2.56 (m, 1H), 2.20–2.14 (m, 1H). HRMS (ESI^+^) calcd for C_15_H_16_N_3_O_2_ (M + H)^+^ 270.1237 found 270.1240.
*(S)-5-((6-Amino-2,3-dihydro-1H-inden-1-yl)oxy)nicotinamide* (**27h**). In a manner similar to that described for the preparation of compound **27a**, aminolysis of **26h** (200 mg, 0.70 mmol) afforded **27h** as a light yellow solid (150 mg, 79%). ^1^H NMR (CDCl_3_, 600 MHz) δ 8.58 (d, *J* = 1.2 Hz, 1H), 8.49 (d, *J* = 3.0 Hz, 1H), 7.80 (dd, *J* = 2.4, 2.4 Hz, 1H), 7.10 (d, *J* = 7.8 Hz, 1H), 6.74 (s, 1H), 6.69 (dd, *J* = 8.4, 2.4 Hz, 1H), 5.77 (dd, *J* = 6.0, 4.2 Hz, 1H), 3.07–3.00 (m, 1H), 2.87–2.81 (m, 1H), 2.62–2.55 (m, 1H), 2.20–2.13 (m, 1H). HRMS (ESI^+^) calcd for C_15_H_16_N_3_O_2_ (M + H)^+^ 270.1237 found 270.1249.

**Initial SIRT1-3 Biochemical Assays.** Initial biochemical assays against human SIRT1-3 were performed at Reaction Biology Corporation (RBC) (Malvern, PA, USA, http://www.reactionbiology.com) as reported previously [29]. Briefly, the concentrations of SIRT1, SIRT2, and SIRT3 were 91, 233, and 917 nM, respectively. A fluorogenic 7-amino-4-methylcoumarin (AMC)-labeled peptide Ac-Arg-His-Lys-Lys(Ac)-AMC was used as the peptide substrate for SIRT1-3. The testing was performed in a two-step fashion. First, 50 μM AMC-labeled substrate was incubated for 2 h at 30 °C to produce the deacetylated substrate. Second, the deacetylated substrate was digested by a mixture of developer to release AMC that was detected at 360/460 Ex/Em. Inhibitors were tested in a 10-dose mode with a 3-fold serial dilution starting at 200 μM (200 μM–10.2 nM). The IC_50_ values were determined from the sigmoidal dose-response curves using GraphPad Prism.

**In-house SIRT1-3 Biochemical Assays.** These assays were performed as reported previously [30]. Briefly, SIRT1, SIRT2, and SIRT3 were used at 0.25, 0.10, and 1.5 µM, respectively. NAD and the peptide substrate ((Ac)RHKK(Ac)-AMC) were used at the *K*_M_ values determined for each enzyme to accurately assess an inhibitor’s selectivity against SIRT1-3. The reaction was allowed to proceed at 37 °C for the desired time and then quenched by the addition of a developing buffer. After the mixture was developed for 20 min at 37 °C, the fluorescence intensity was measured using an excitation at 355 nm and an emission at 460 nm. The data were analyzed using either the Morrison equation (SIRT2) or the four-parameter dose response equation (SIRT1 and 3) in GraphPad Prism. 

**Molecular Modeling.** The modeling study was carried out as described previously [29]. The X-ray crystal structure of human SIRT2 (PDB: 1j8f) [31] was used and the energy minimization was performed using OPLS 2005 forcefield. SIRT2 inhibitors were generated by LigPrep and then docked into the Glide grid (20 × 20 × 20 Å) encompassing the active site of SIRT2. Docking was carried out in both standard SP and XP modes. Structural visualization and representation were performed with PyMOL.

## Figures and Tables

**Table 1:** Inhibitory activity of the constrained analogs *^a^*.

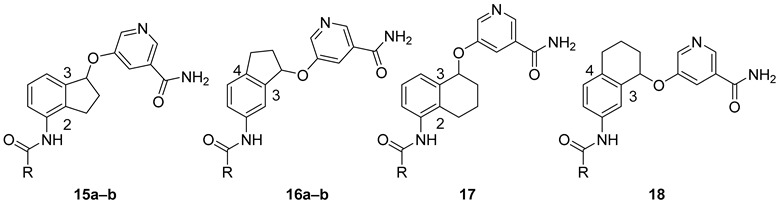				
**Compound**	**R**	**IC_50_ (µM**)		
		**SIRT1**	**SIRT2**	**SIRT3**
**7a** *^b^*	(4-Me-3-NO_2_)-Ph	7.14	0.107	6.39
**7b** *^b^*	4-Py	2.46	0.0341	2.40
**15a**	(4-Me-3-NO_2_)-Ph	25.6	0.281	4.36
**15b**	4-Py	9.99	0.0503	2.86
**16a**	(4-Me-3-NO_2_)-Ph	23.6	0.540	16.0
**16b**	4-Py	36.6	0.109	11.6
**17**	(4-Me-3-NO_2_)-Ph	23.9	3.28	15.0
**18**	(4-Me-3-NO_2_)-Ph	>200	3.00	32.8

*^a^* Inhibitory activity was determined as described in Initial SIRT1-3 Biochemical Assays in the Experimental section. *^b^* Data were previously reported as IC_50_ values [30].

**Table 2:** Inhibitory activity of the constrained analogs: The effect of stereochemistry *^a^*.

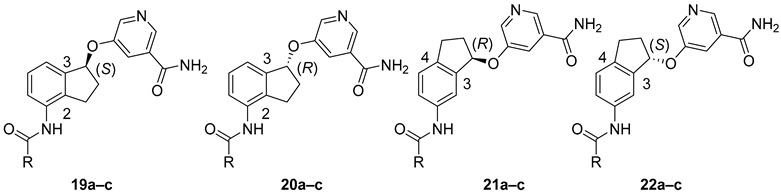				
**Compound**	**R**	***K*_i_^app^** (**µM**)		
		**SIRT1**	**SIRT2**	**SIRT3**
**7b** *^b^*	4-Py	3.0 ± 0.1	0.14 ± 0.02	7.2 ± 0.9
**7c** *^b^*	(4-morpholino)-Ph	5.0 ± 1.2	0.073 ± 0.011	5.4 ± 1.1
**7d**	(4-Ph)-Ph	>100	0.217 ± 0.033	>100
**19a**	4-Py	15.9 ± 1.3	0.147 ± 0.016	12.3 ± 1.5
**19b**	(4-morpholino)-Ph	11.0 ± 1.3	0.132 ± 0.009	5.40 ± 0.50
**19c**	(4-Ph)-Ph	>100	0.064 ± 0.008	>100
**20a**	4-Py	43.5 ± 2.7	0.717 ± 0.061	14.8 ± 1.1
**20b**	(4-morpholino)-Ph	45.8 ± 3.0	0.845 ± 0.055	18.4 ± 1.2
**20c**	(4-Ph)-Ph	>100	0.451 ± 0.043	>100
**21a**	4-Py	>100	1.44 ± 0.18	100 ± 12
**21b**	(4-morpholino)-Ph	>100	0.863 ± 0.087	>100
**21c**	(4-Ph)-Ph	>100	0.895 ± 0.169	>100
**22a**	4-Py	>100	1.11 ± 0.11	50.8 ± 4.2
**22b**	(4-morpholino)-Ph	>100	0.994 ± 0.114	88.3 ± 13.9
**22c**	(4-Ph)-Ph	>100	0.906 ± 0.183	>100

*^a^* Inhibitory activity was determined as described in In-house SIRT1-3 Biochemical Assays in the Experimental section. *^b^* Data were previously reported as *K*_i_^app^ values [30].

## Data Availability

Data are contained within the article and Supplementary Materials.

## Supplementary Materials

The following supporting information can be downloaded at: https://www.mdpi.com/article/10.3390/molecules28227655/s1. ^1^H NMR spectra of the final compounds and synthetic intermediates; HPLC chromatograms (two solvent conditions) of the final compounds; and HRMS spectra of the final compounds.

## Abbreviations

SIRT2, Sirtuin 2; NAD^+^, nicotinamide adenine dinucleotide; SAR, structure-activity relationship.
