# Supplementary material for: 5-((3-Amidobenzyl)oxy)nicotinamides as SIRT2 Inhibitors: A Study of Constrained Analogs
Source: Molecules. 2023 Nov 18;28(22):7655. doi: 10.3390/molecules28227655 (PMC10674942; doi:10.3390/molecules28227655)

Supplementary Materials

5-((3-Amidobenzyl)oxy)nicotinamides as SIRT2 Inhibitors: A Study of Constrained Analogs

Teng Ai, Daniel J. Wilson, Liqiang Chen*

Center for Drug Design, College of Pharmacy, University of Minnesota, Minneapolis, Minnesota 55455, United States

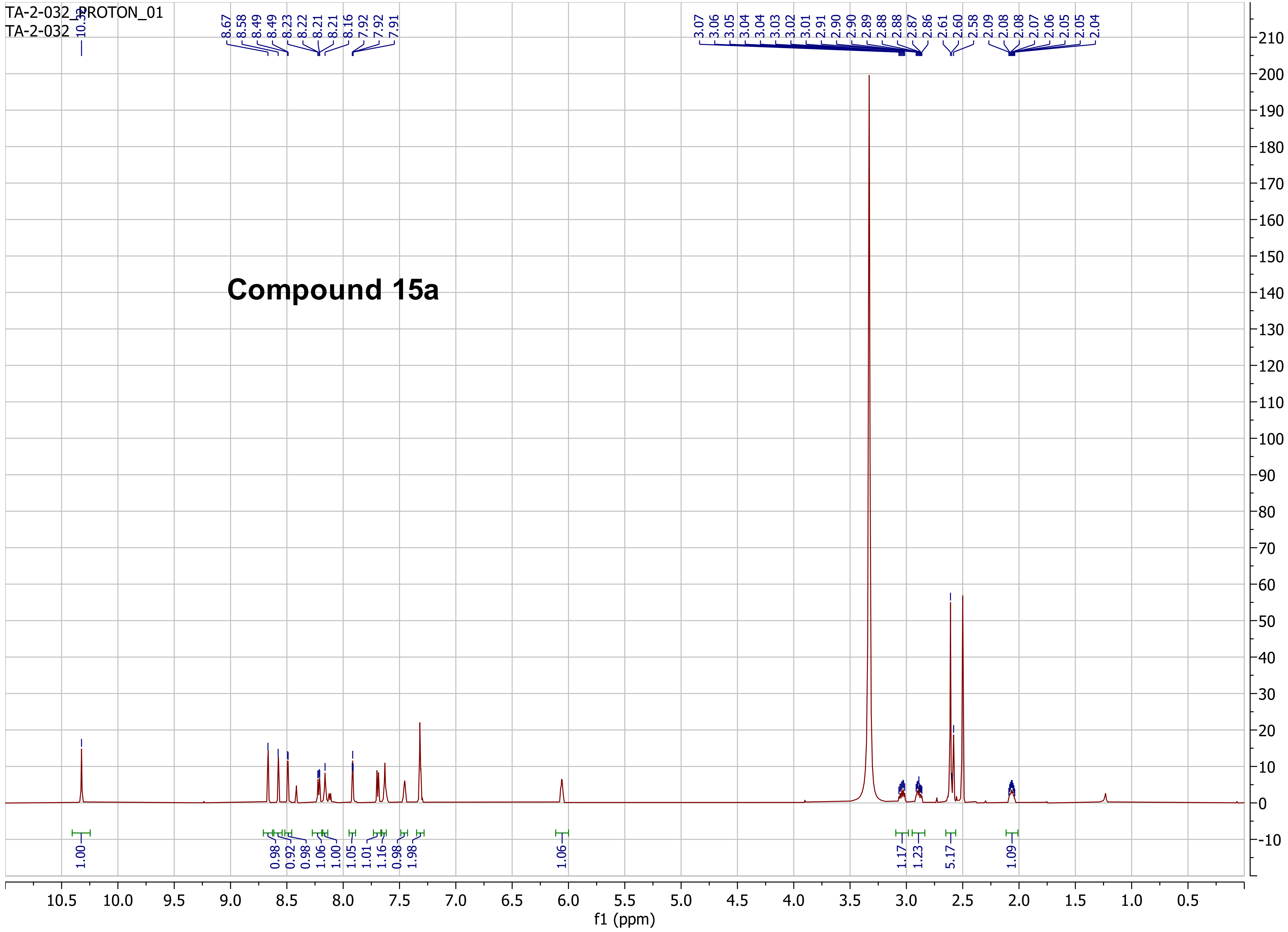
TA-2-032_PROTON_01
TA-2-032
10.32
8.67 8.58 8.49 8.49 8.23 8.22 8.21 8.21 8.16 7.92 7.92 7.91
3.07 3.06 3.05 3.04 3.04 3.03 3.02 3.01 2.91 2.90 2.90 2.89 2.88 2.88 2.87 2.86 2.61 2.60 2.58 2.09 2.08 2.08 2.07 2.06 2.05 2.05 2.04
Compound 15a
1.00 0.98 0.92 0.98 1.06 1.00 1.05 1.01 1.16 0.98 1.98 1.06 1.17 1.23 5.17 1.09
f1 (ppm)

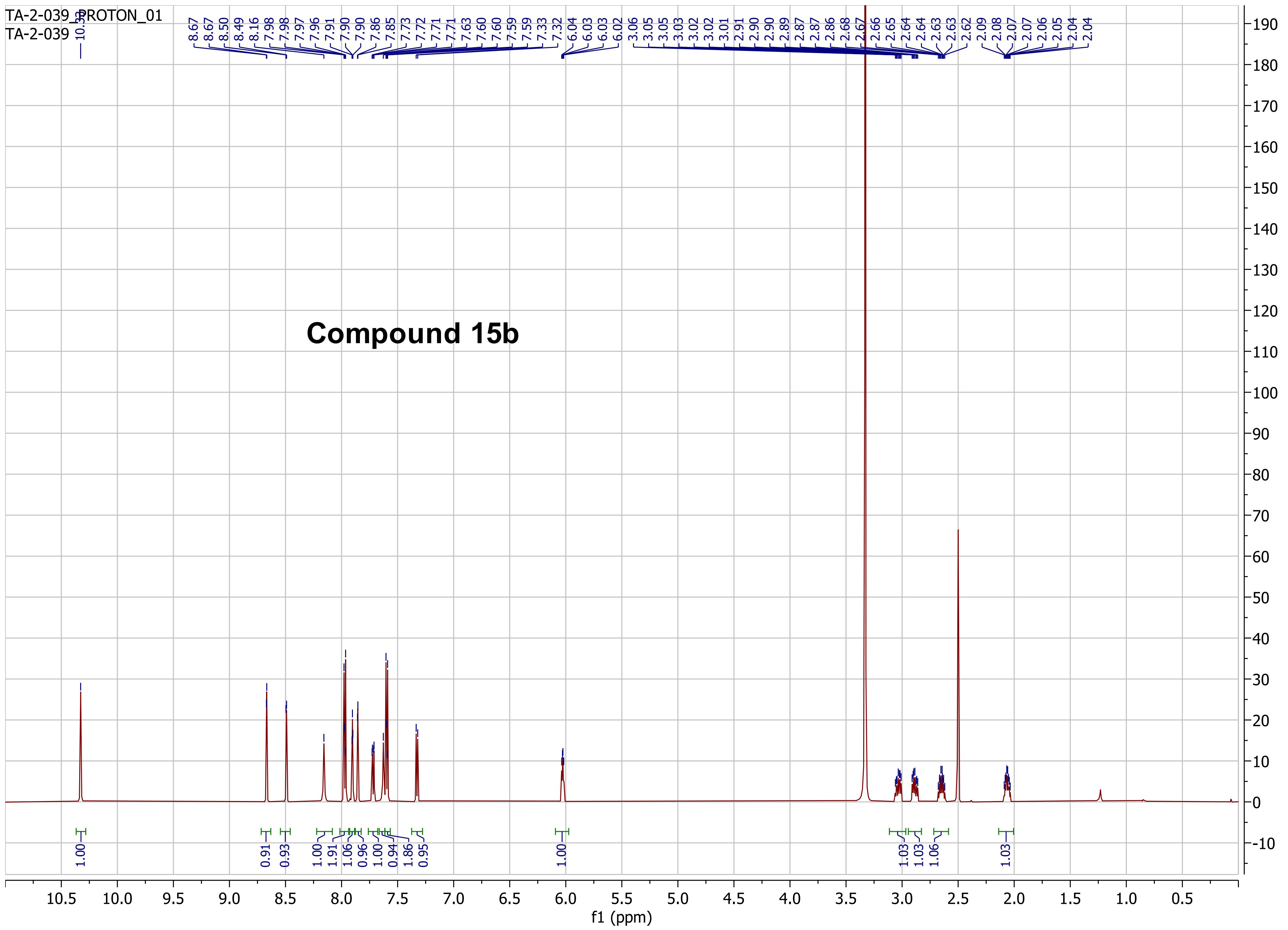
TA-2-039_PROTON_01
TA-2-039
Compound 15b
10.33
8.67 8.67 8.50 8.49 8.16 7.98 7.98 7.97 7.96 7.91 7.90 7.90 7.86 7.85 7.73 7.72 7.71 7.71 7.63 7.60 7.60 7.59 7.59 7.33 7.32 6.04 6.03 6.03 6.02 3.06 3.05 3.05 3.03 3.02 3.02 3.01 2.91 2.90 2.90 2.89 2.87 2.87 2.86 2.68 2.67 2.66 2.65 2.64 2.64 2.63 2.63 2.62 2.09 2.08 2.07 2.07 2.06 2.05 2.04 2.04
1.00 0.91 0.93 1.00 1.91 1.06 0.96 1.00 0.94 1.86 0.95 1.00 1.03 1.03 1.06 1.03
f1 (ppm)

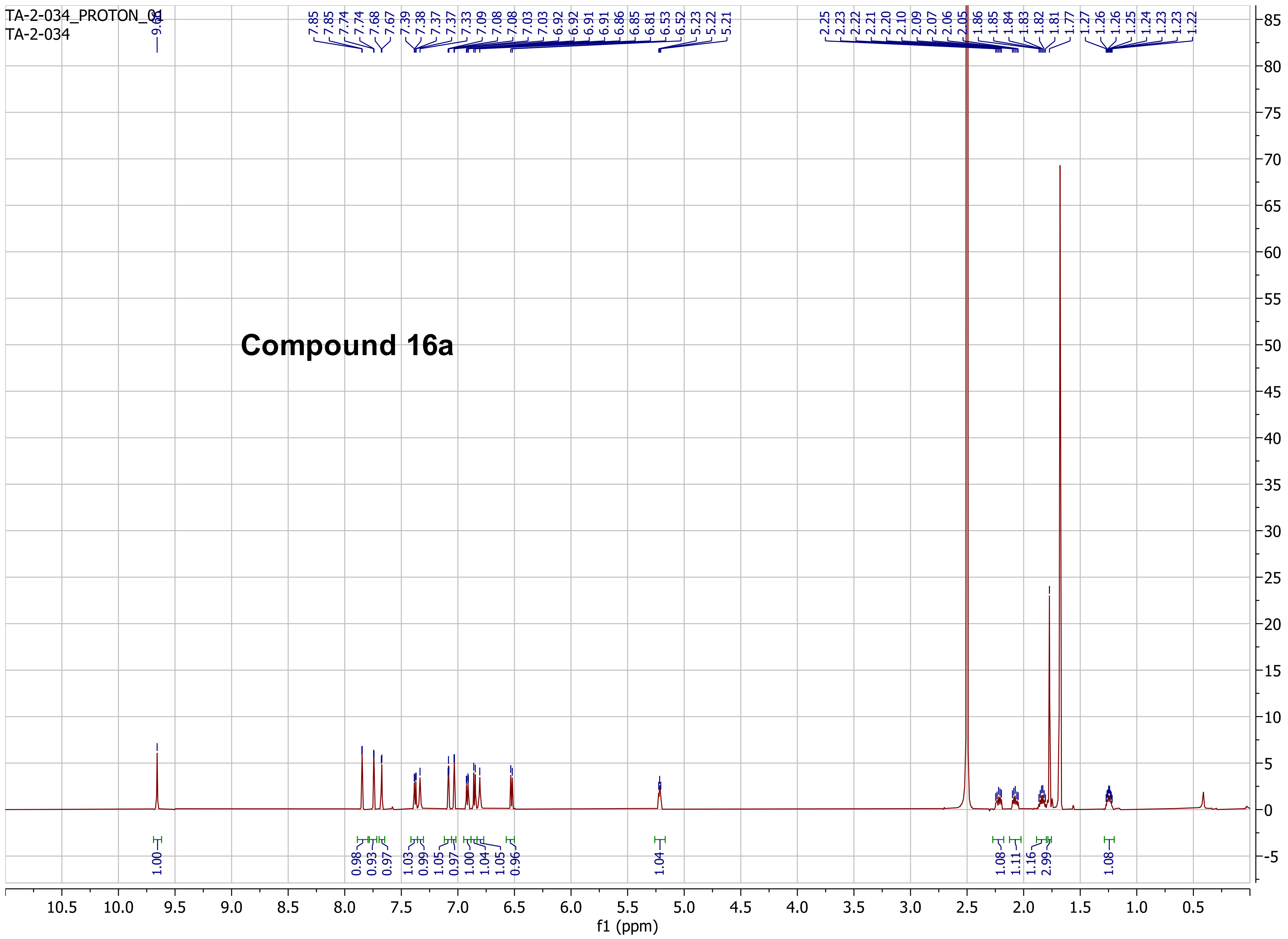
TA-2-034_PROTON_01
TA-2-034
Compound 16a
f1 (ppm)

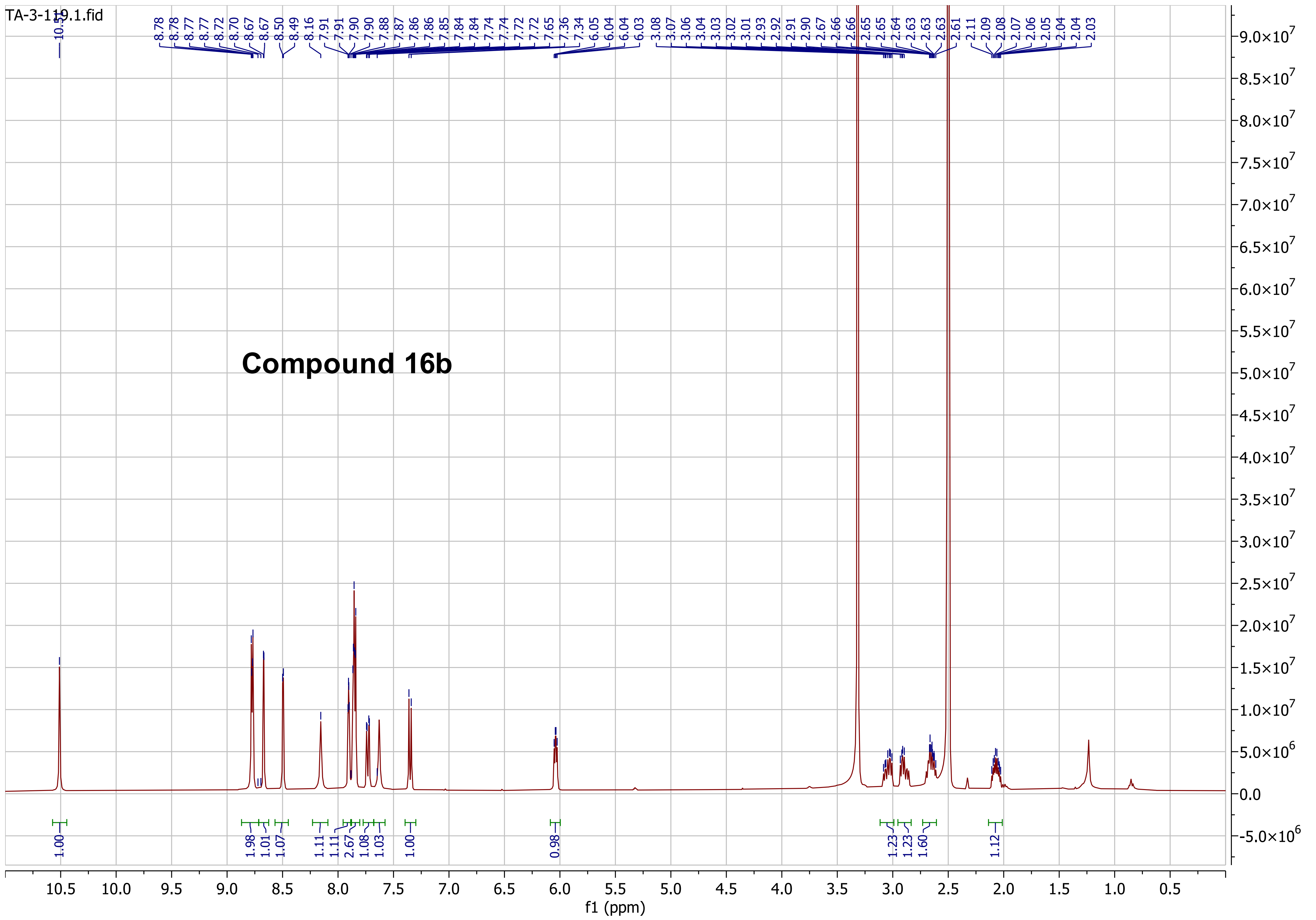

TA-3-119.1.fid
Compound 16b
f1 (ppm)

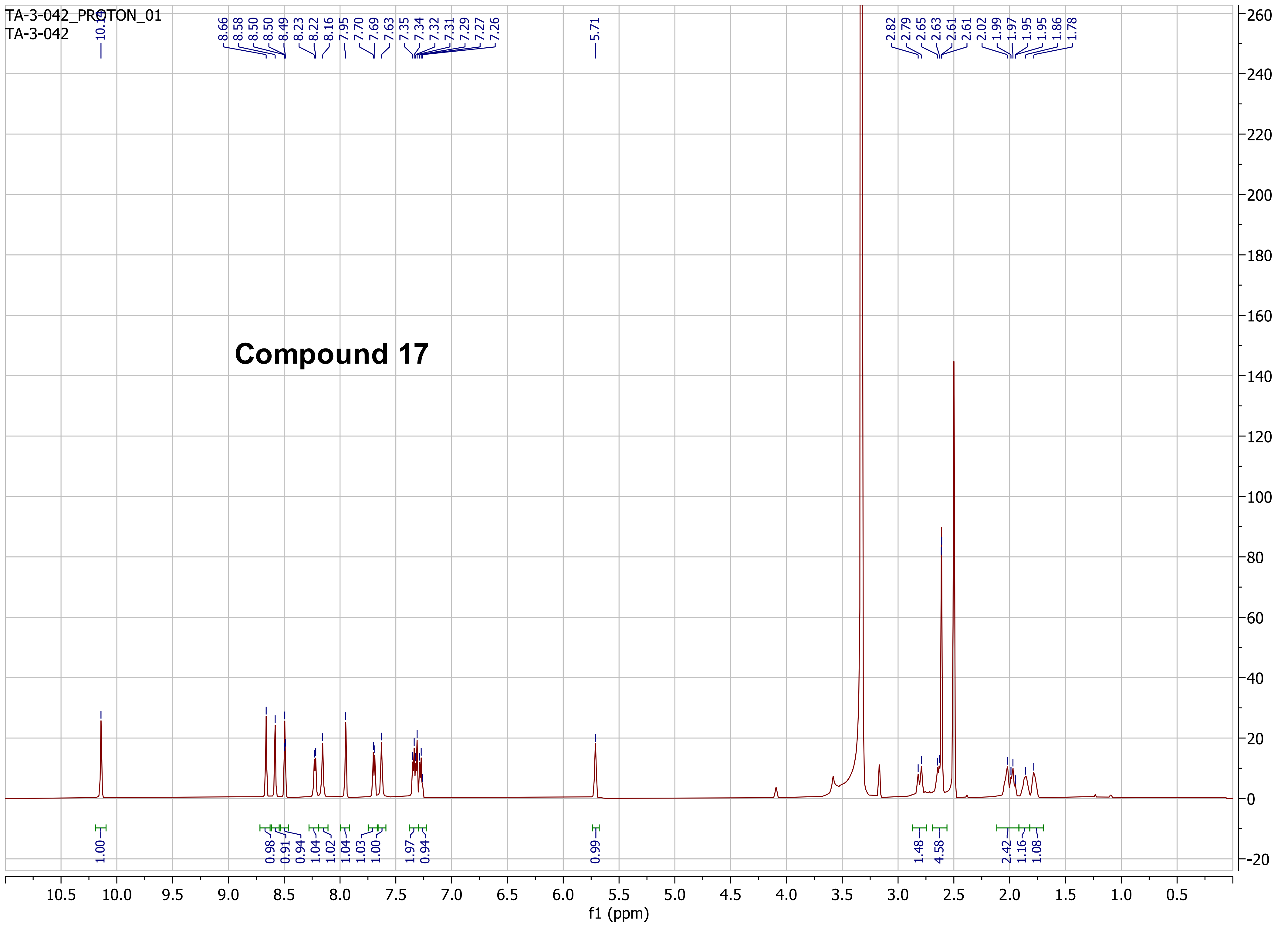

TA-3-042_PROTON_01
TA-3-042
Compound 17
f1 (ppm)

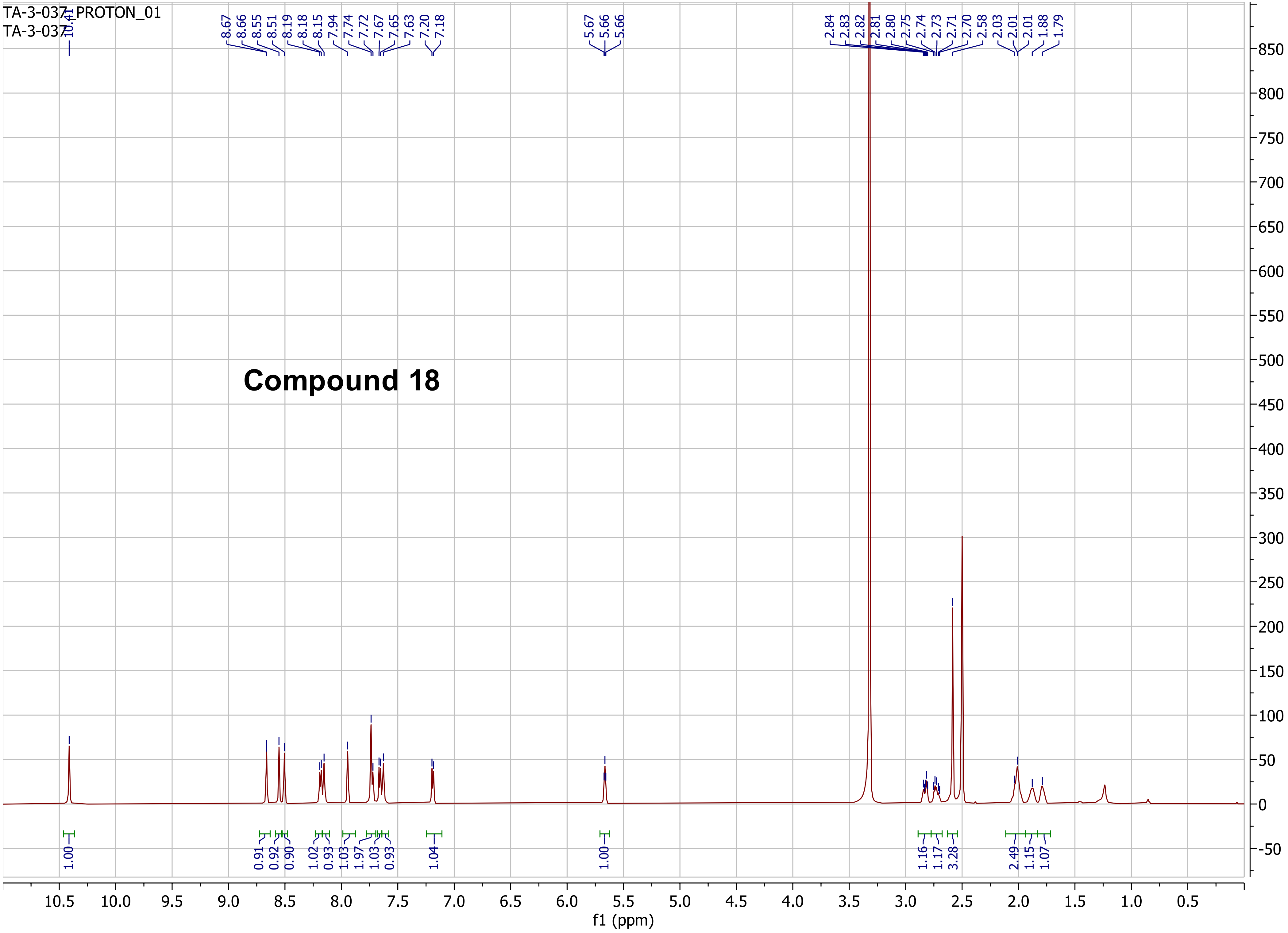

Compound 18

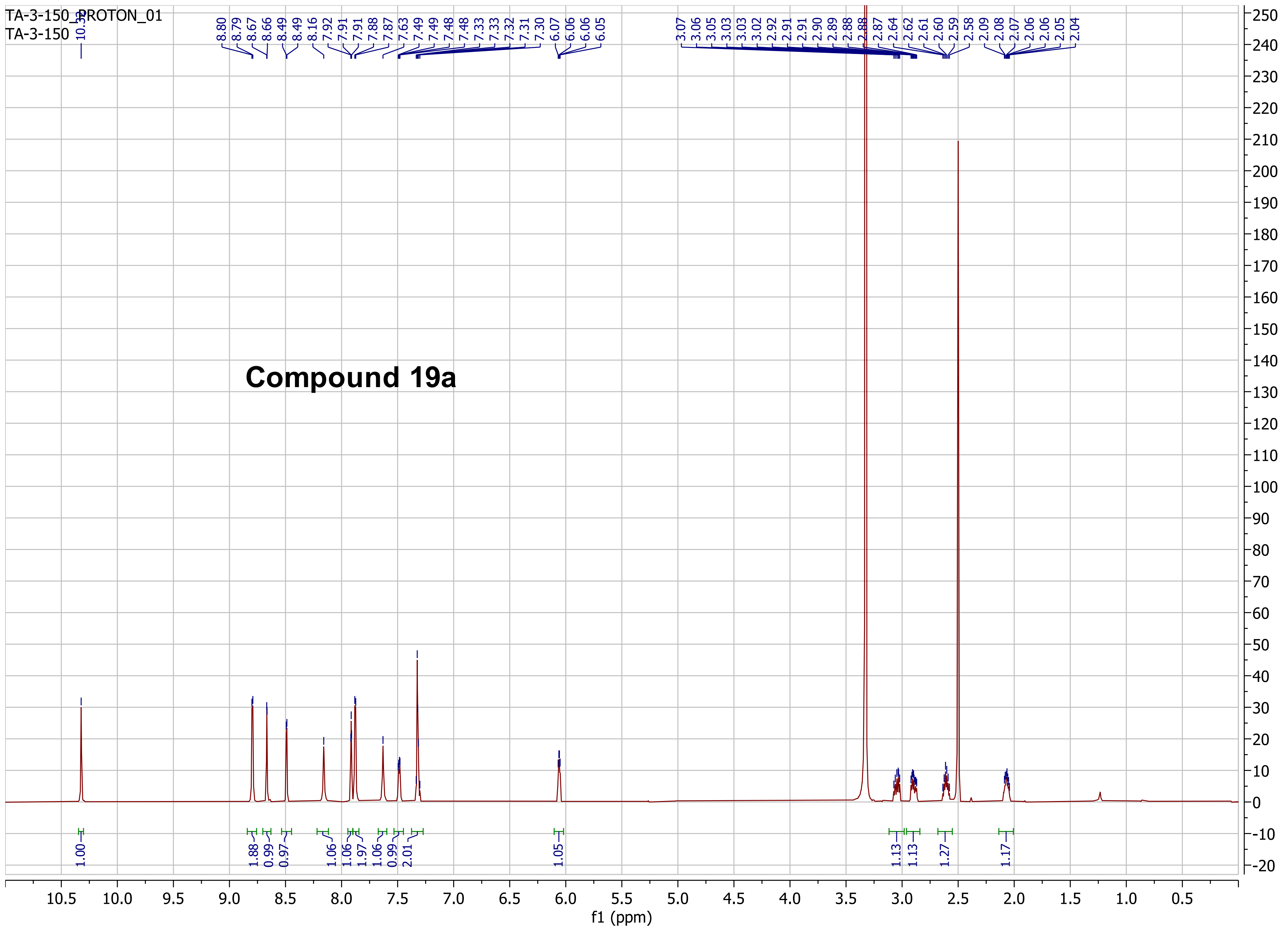
TA-3-150_PROTON_01
TA-3-150
10.32
8.80 8.79 8.67 8.66 8.49 8.49 8.16 7.92 7.91 7.91 7.88 7.87 7.63 7.49 7.49 7.48 7.48 7.33 7.33 7.32 7.31 7.30 6.07 6.06 6.06 6.05
3.07 3.06 3.05 3.03 3.03 3.02 2.92 2.91 2.91 2.90 2.89 2.88 2.88 2.87 2.64 2.62 2.61 2.60 2.59 2.58 2.09 2.08 2.07 2.06 2.06 2.05 2.04
Compound 19a
1.00 1.88 0.99 0.97 1.06 1.06 1.97 1.06 0.99 2.01 1.05 1.13 1.13 1.27 1.17
f1 (ppm)

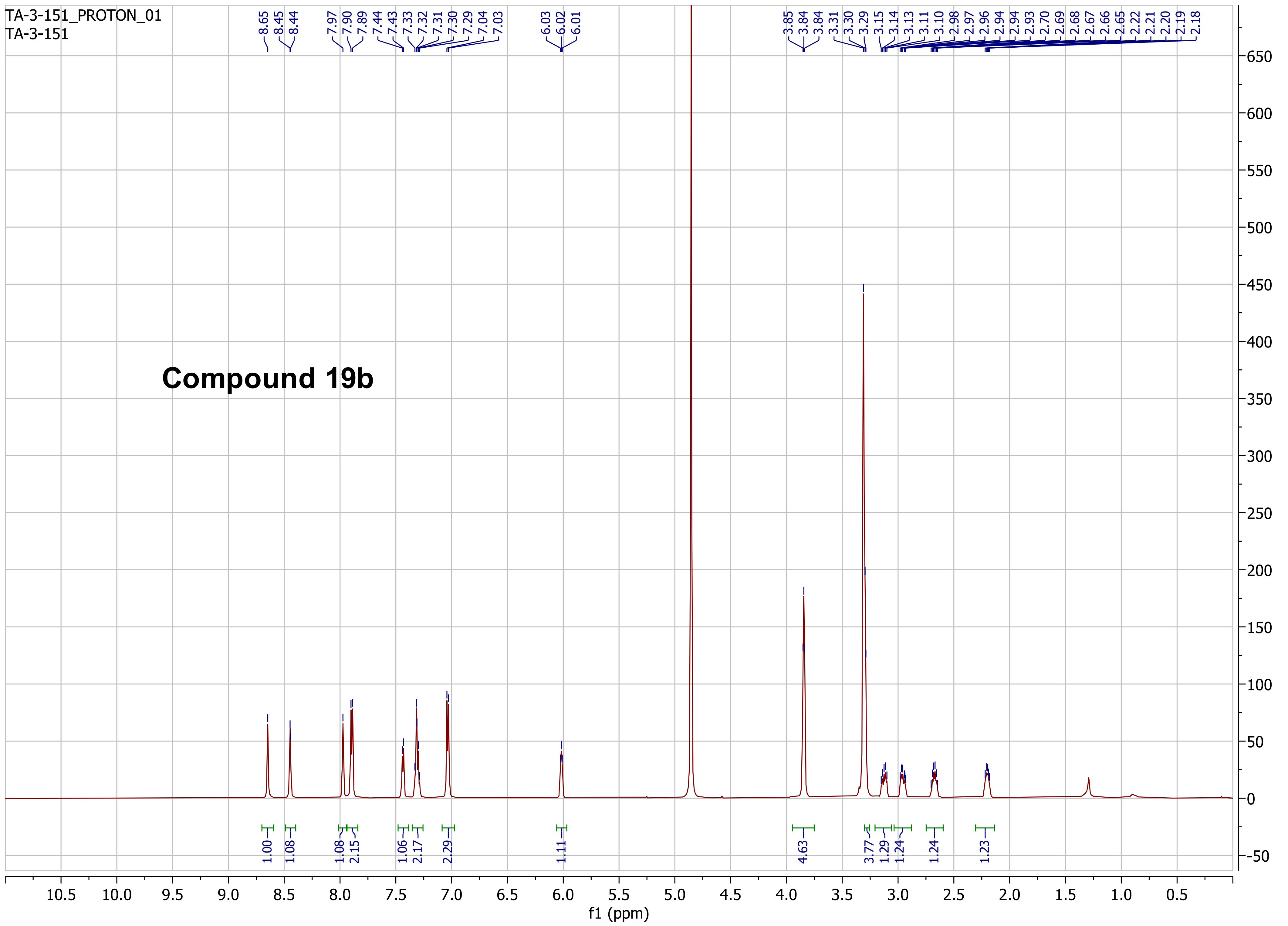
TA-3-151_PROTON_01
TA-3-151
Compound 19b
8.65
8.45
8.44
7.97
7.90
7.89
7.44
7.43
7.33
7.32
7.31
7.30
7.29
7.04
7.03
6.03
6.02
6.01
3.85
3.84
3.84
3.31
3.30
3.29
3.15
3.14
3.13
3.11
3.10
2.98
2.97
2.96
2.94
2.94
2.93
2.70
2.69
2.68
2.67
2.66
2.65
2.22
2.21
2.20
2.19
2.18
1.00
1.08
1.08
2.15
1.06
2.17
2.29
1.11
4.63
3.77
1.29
1.24
1.24
1.23
f1 (ppm)

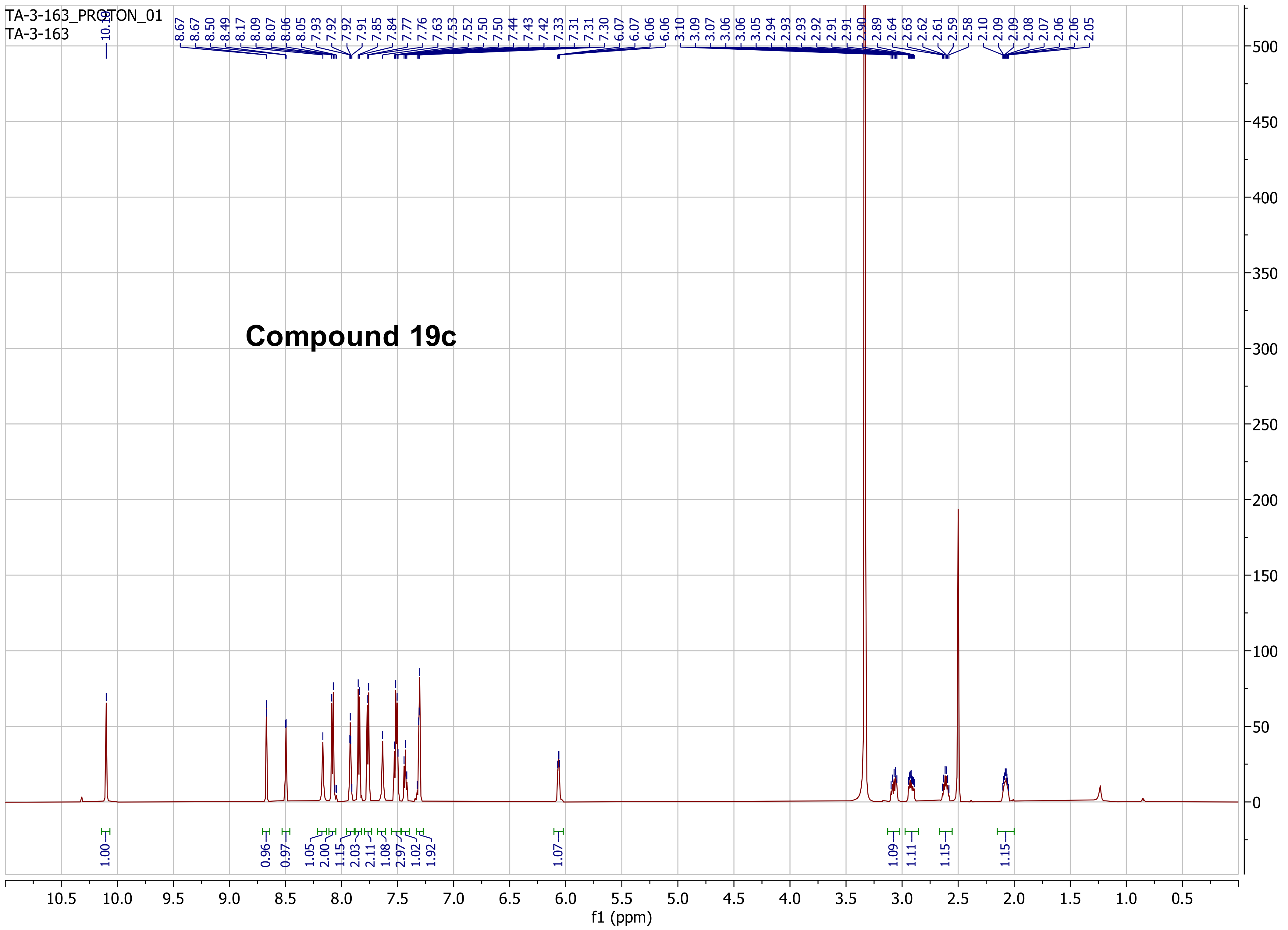
TA-3-163_PROTON_01
TA-3-163
Compound 19c
f1 (ppm)

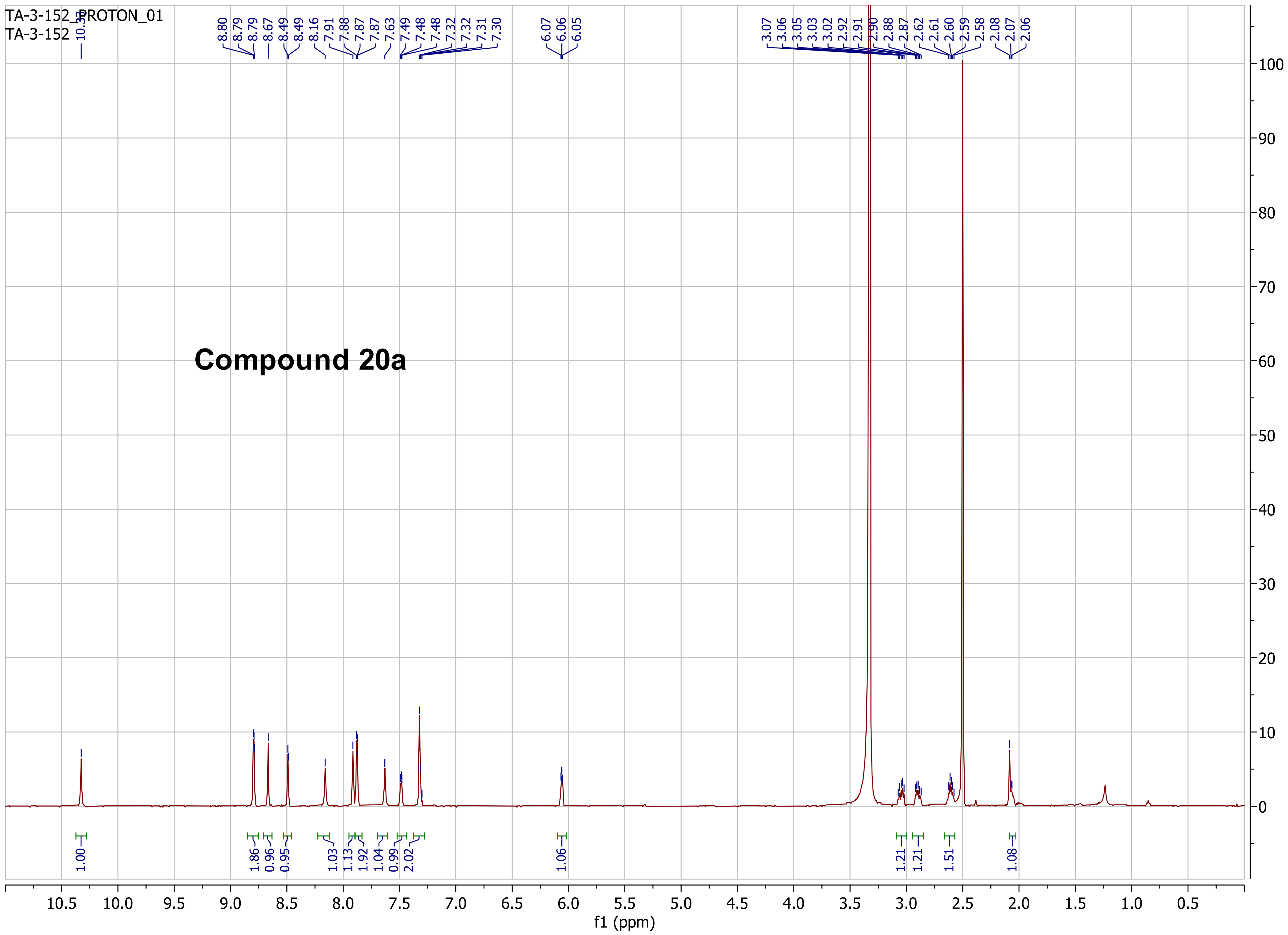
TA-3-152_PROTON_01
TA-3-152
Compound 20a
10.33
8.80
8.79
8.79
8.67
8.49
8.49
8.16
7.91
7.88
7.87
7.87
7.63
7.49
7.48
7.48
7.32
7.32
7.31
7.30
6.07
6.06
6.05
3.07
3.06
3.05
3.03
3.02
2.92
2.91
2.90
2.88
2.87
2.62
2.61
2.60
2.59
2.58
2.08
2.07
2.06
1.00
1.86
0.96
0.95
1.03
1.13
1.92
1.04
0.99
2.02
1.06
1.21
1.21
1.51
1.08
f1 (ppm)

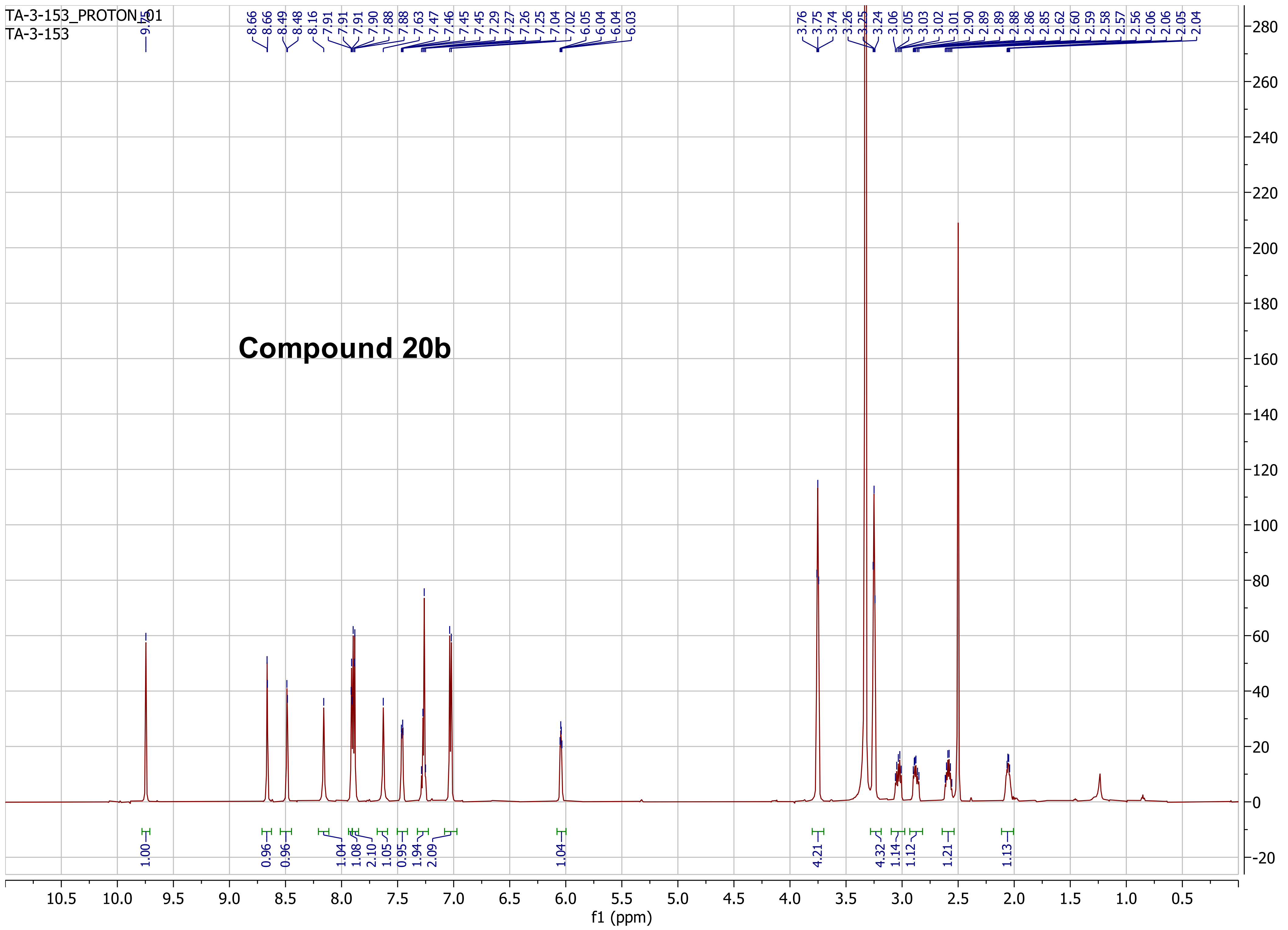
TA-3-153_PROTON_01
TA-3-153
Compound 20b
f1 (ppm)

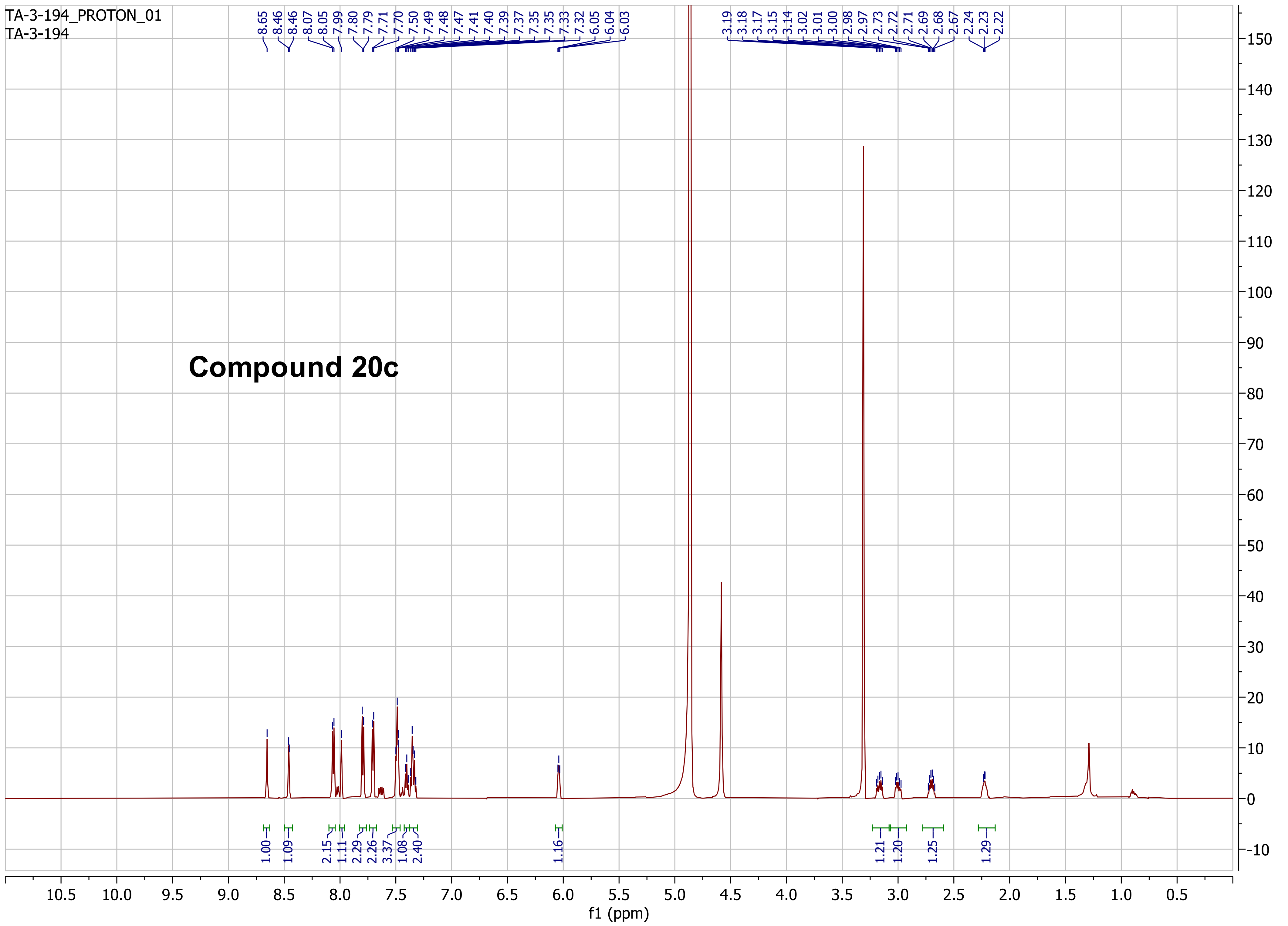
TA-3-194_PROTON_01
TA-3-194
Compound 20c
8.65 8.46 8.46 8.07 8.05 7.99 7.80 7.79 7.71 7.70 7.50 7.49 7.48 7.47 7.41 7.40 7.39 7.37 7.35 7.35 7.33 7.32 6.05 6.04 6.03
3.19 3.18 3.17 3.15 3.14 3.02 3.01 3.00 2.98 2.97 2.73 2.72 2.71 2.69 2.68 2.67 2.24 2.23 2.22
1.00 1.09 2.15 1.11 2.29 2.26 3.37 1.08 2.40 1.16 1.21 1.20 1.25 1.29
f1 (ppm)

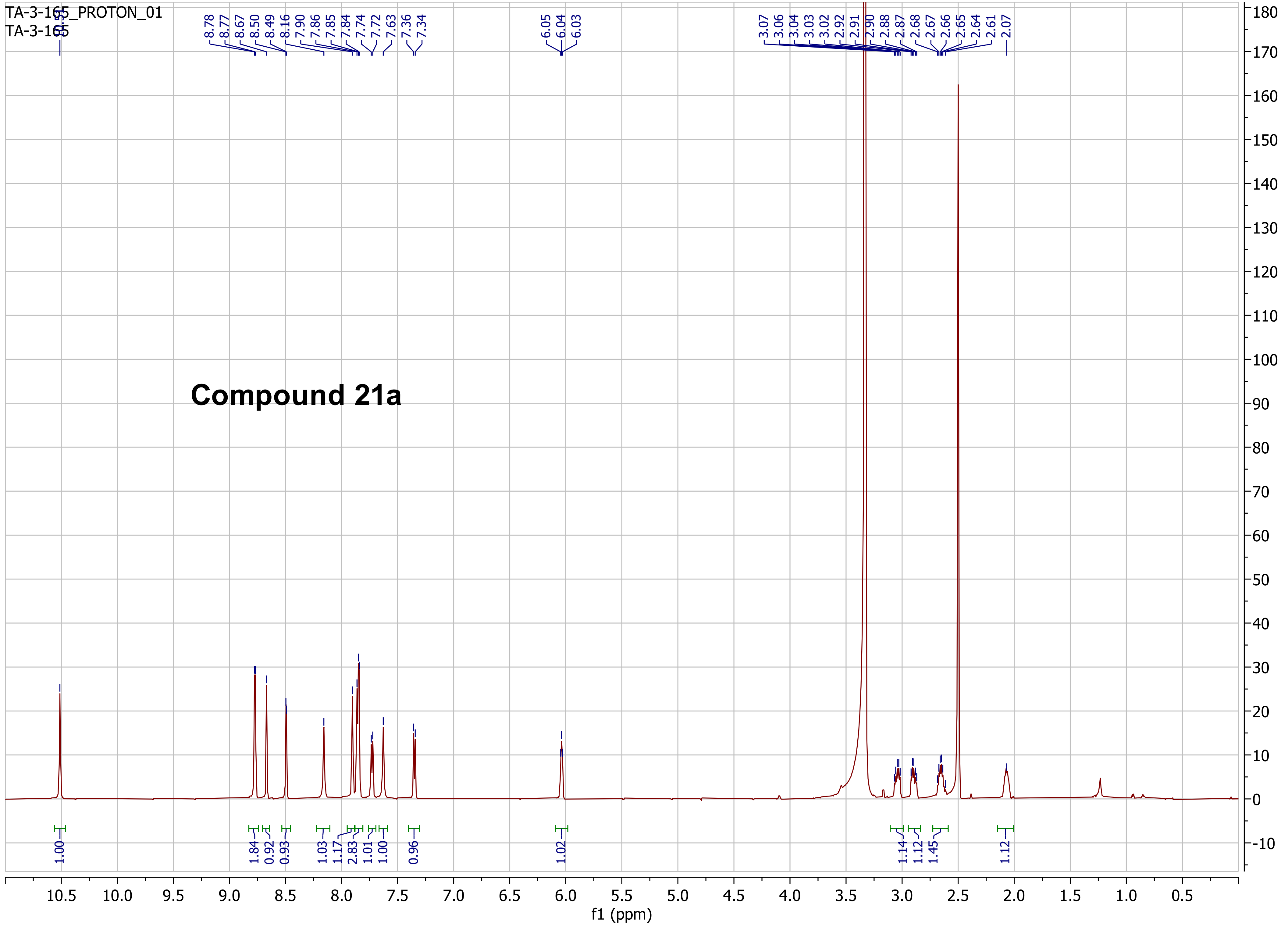

TA-3-165_PROTON_01
TA-3-165
Compound 21a
f1 (ppm)

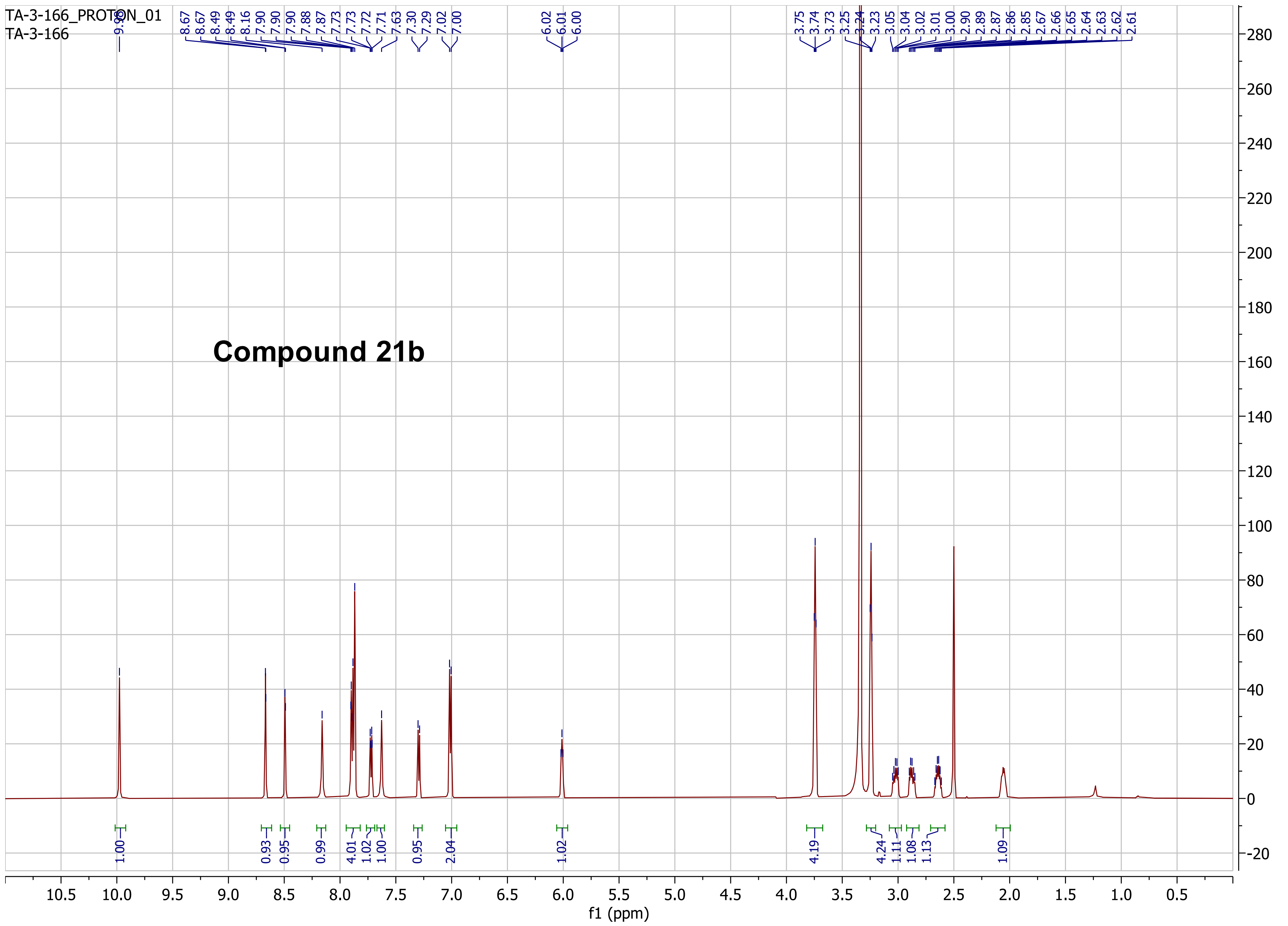

TA-3-166_PROTON_01
TA-3-166
Compound 21b
f1 (ppm)

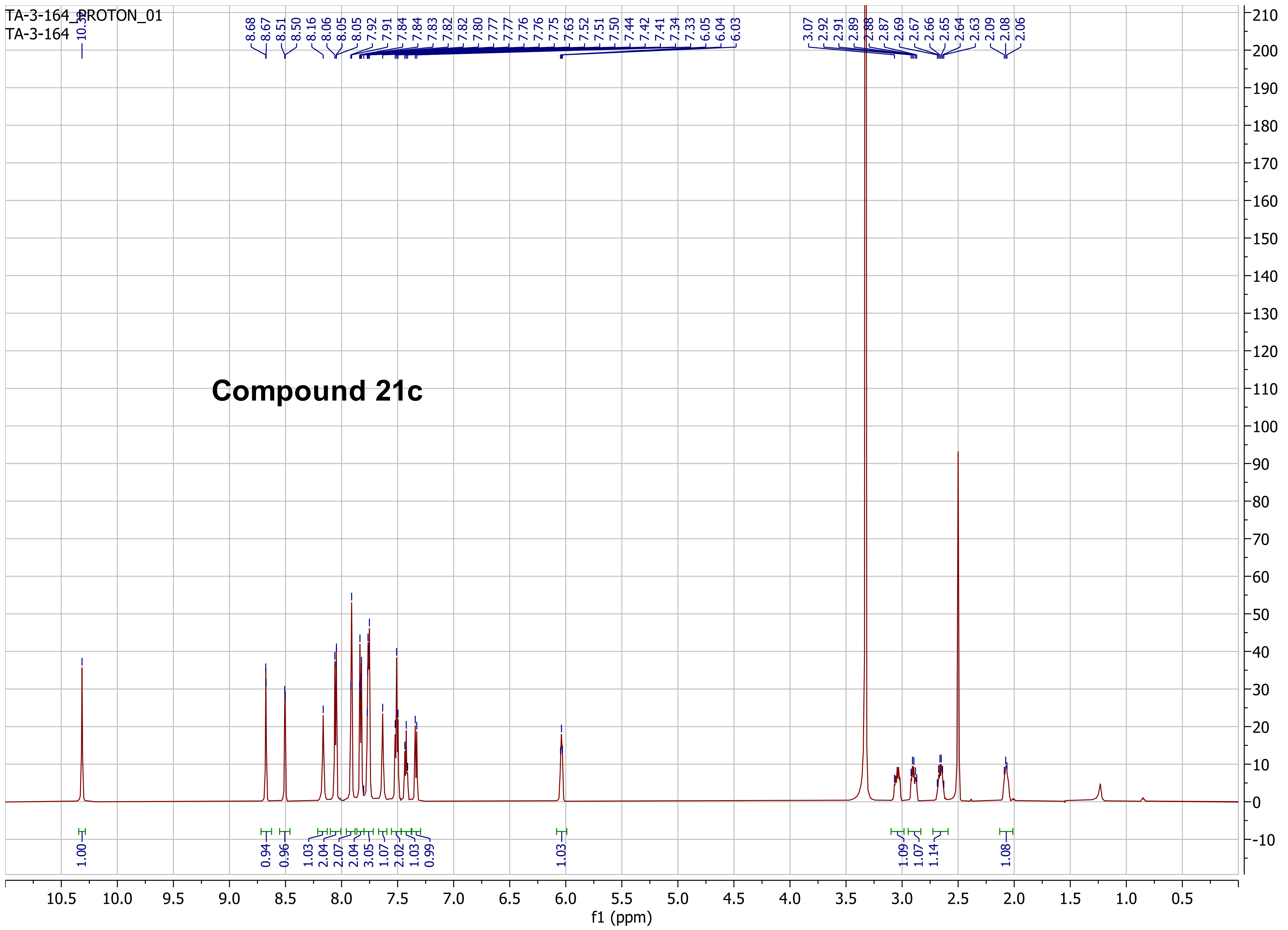

Compound 21c

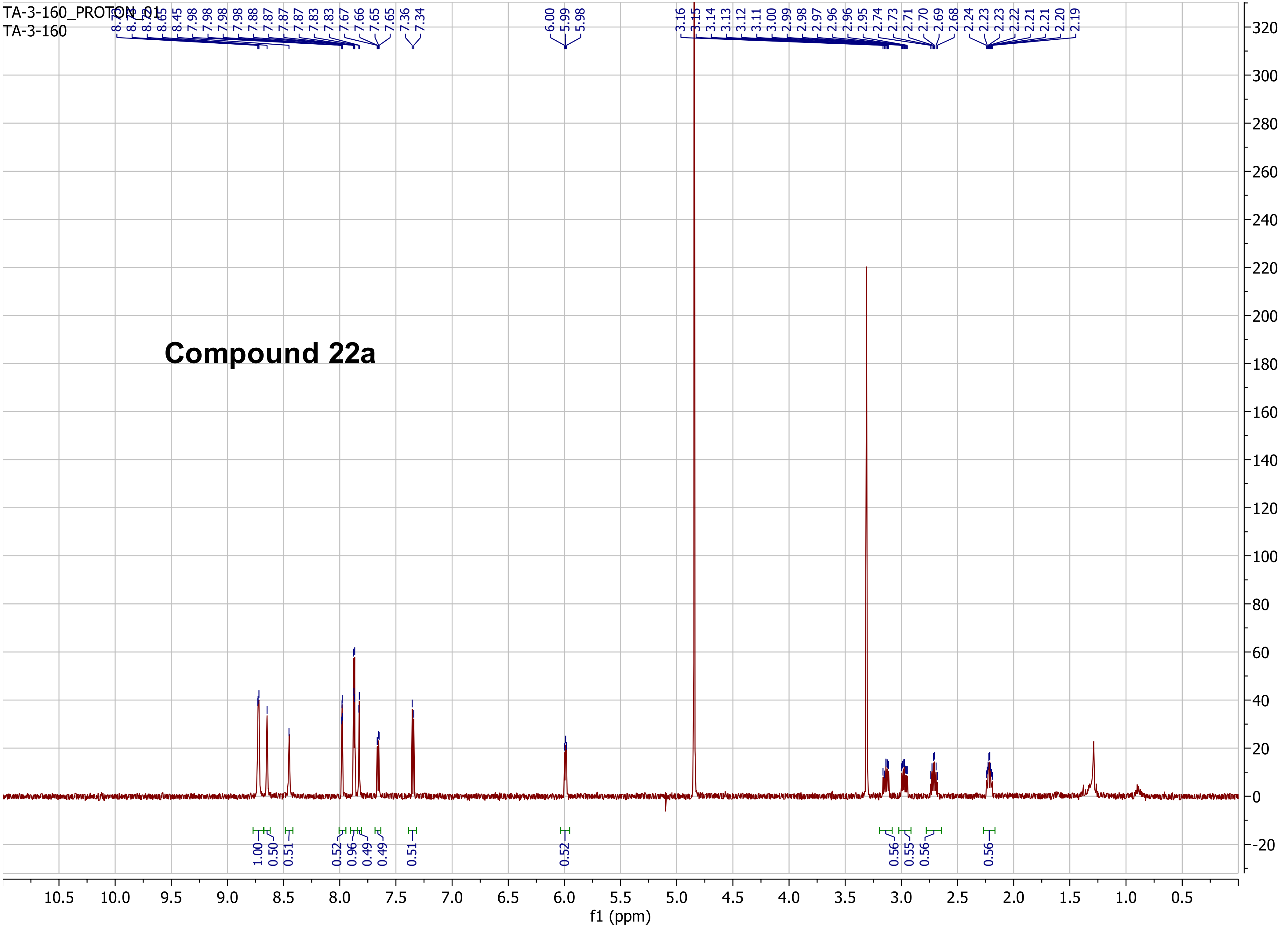
TA-3-160_PROTON_01
TA-3-160
Compound 22a
f1 (ppm)

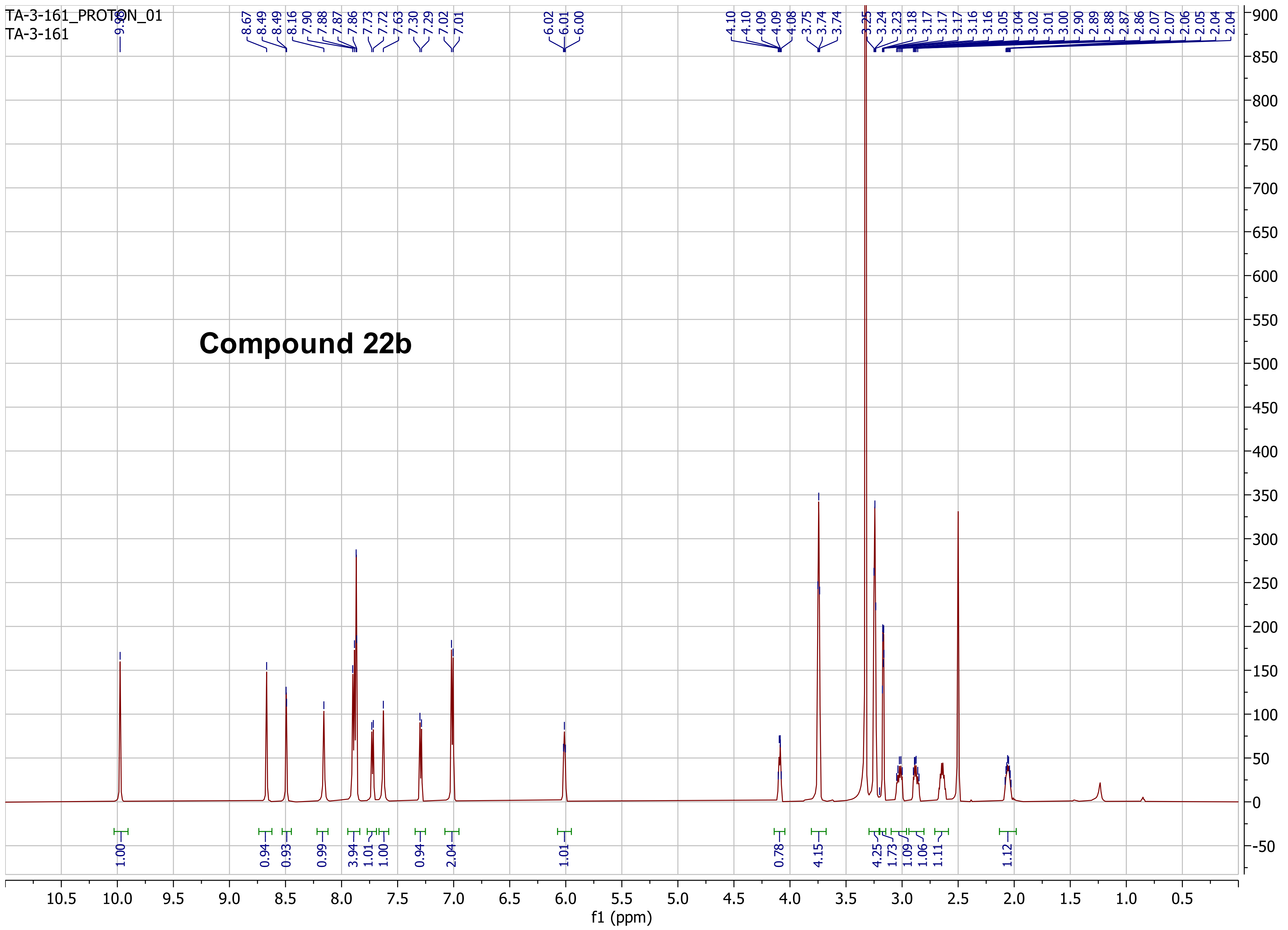
TA-3-161_PROTON_01
TA-3-161
Compound 22b
f1 (ppm)

TA-3-162_PROTON_01
TA-3-162

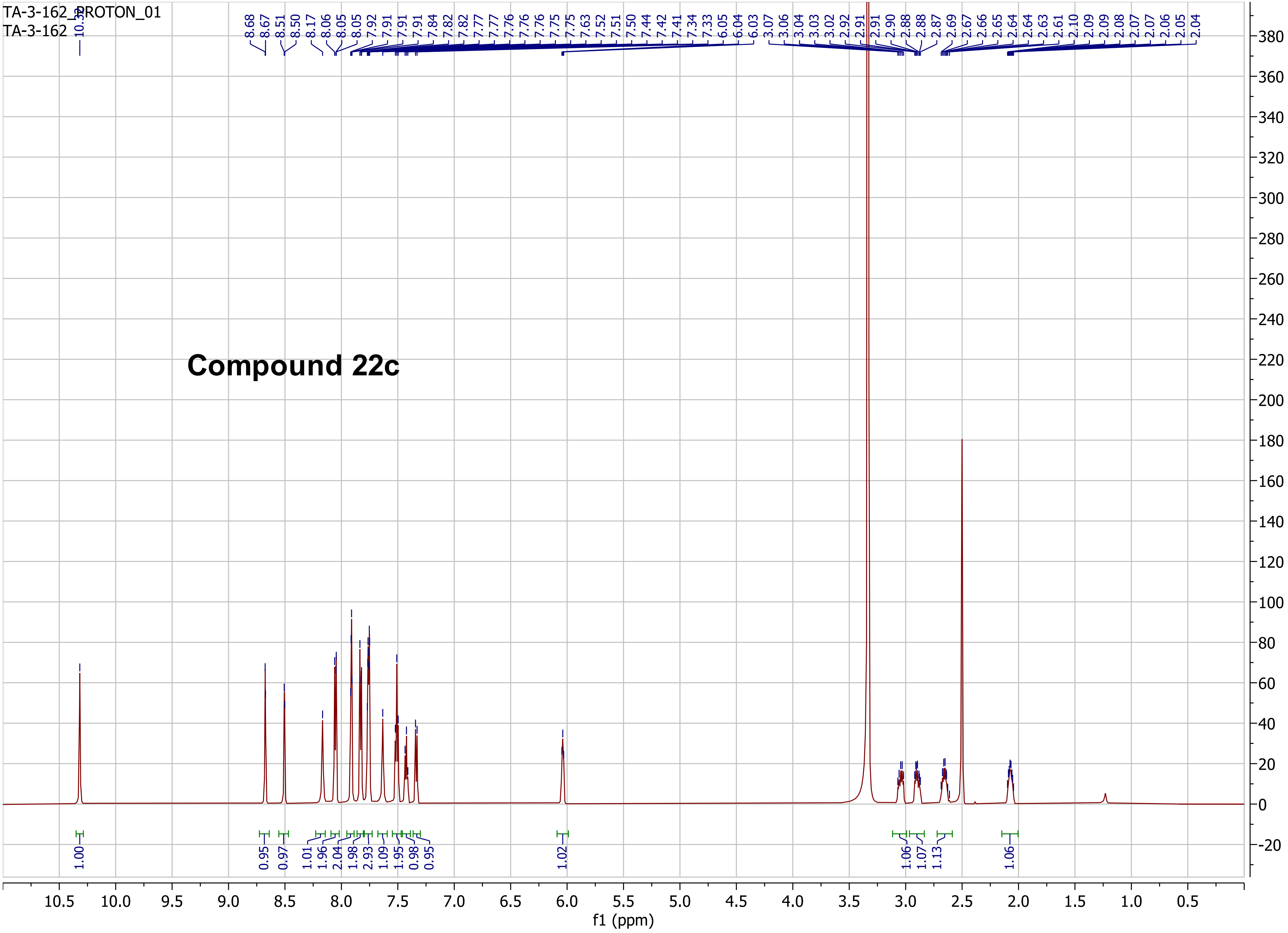

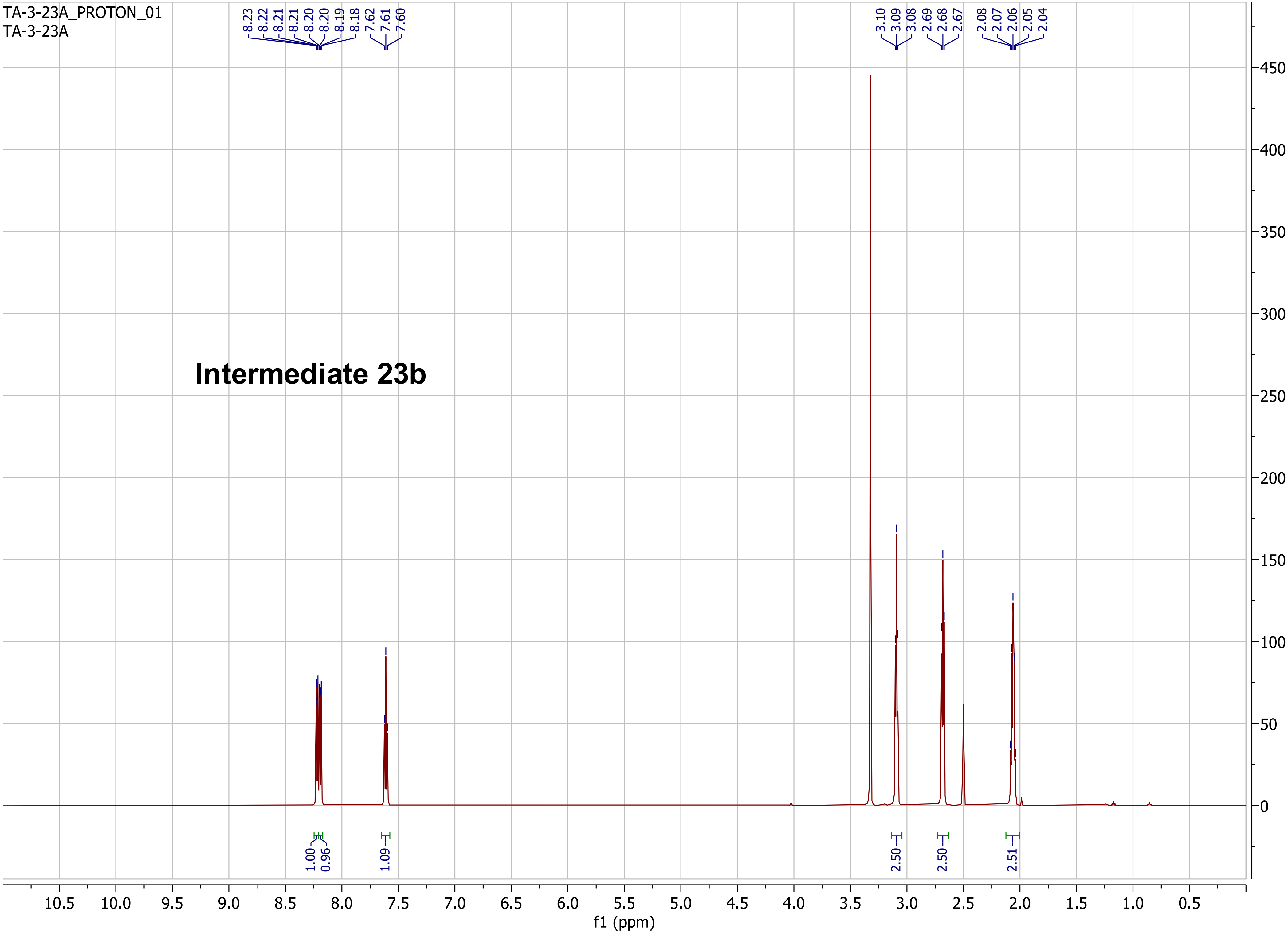
TA-3-23A_PROTON_01
TA-3-23A
Intermediate 23b
8.23 8.22 8.21 8.21 8.20 8.20 8.19 8.18 7.62 7.61 7.60
3.10 3.09 3.08 2.69 2.68 2.67 2.08 2.07 2.06 2.05 2.04
1.00 0.96 1.09 2.50 2.50 2.51
450 400 350 300 250 200 150 100 50 0
10.5 10.0 9.5 9.0 8.5 8.0 7.5 7.0 6.5 6.0 5.5 5.0 4.5 4.0 3.5 3.0 2.5 2.0 1.5 1.0 0.5
f1 (ppm)

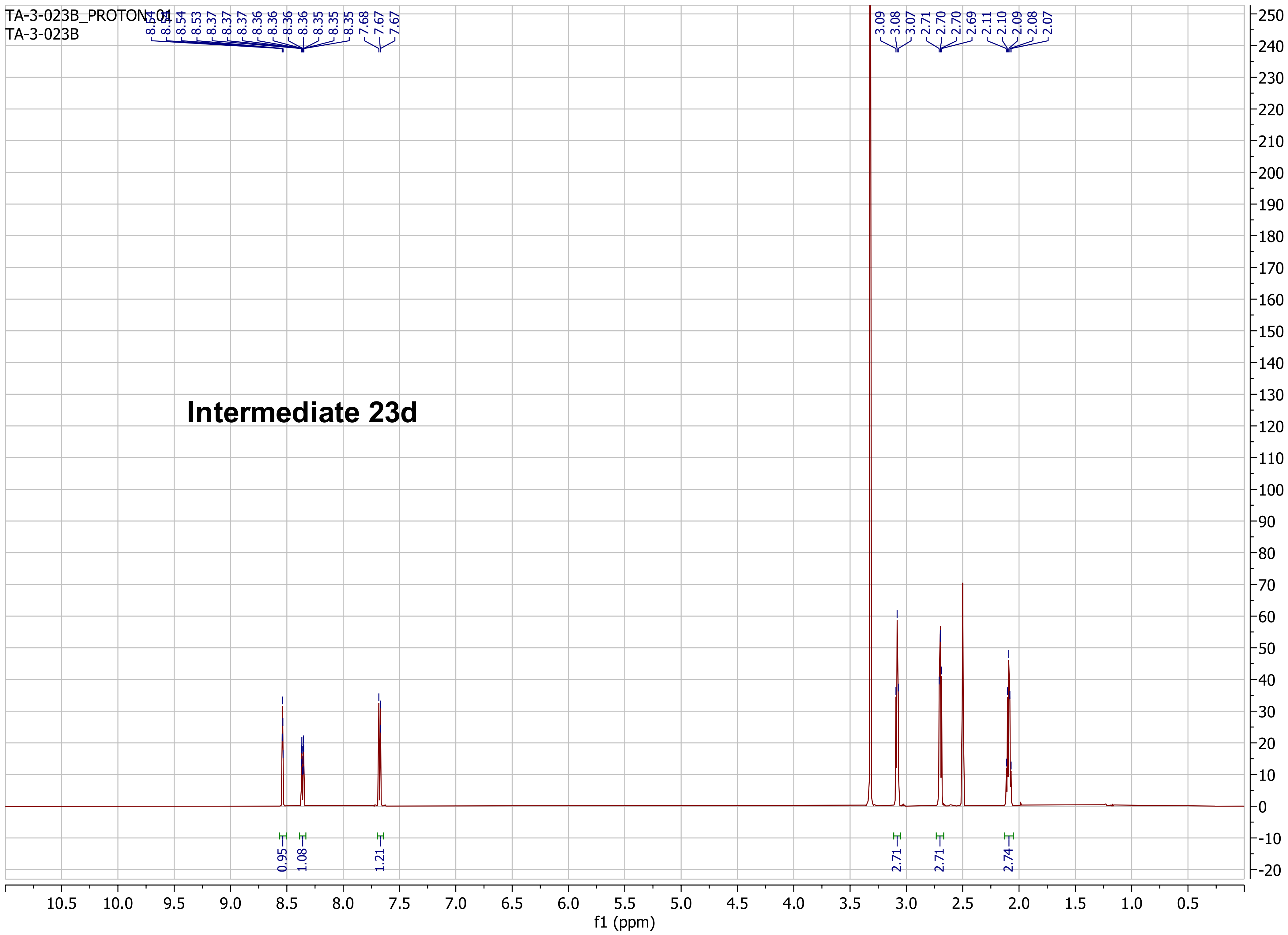

TA-3-023B_PROTON-01
TA-3-023B
Intermediate 23d
f1 (ppm)

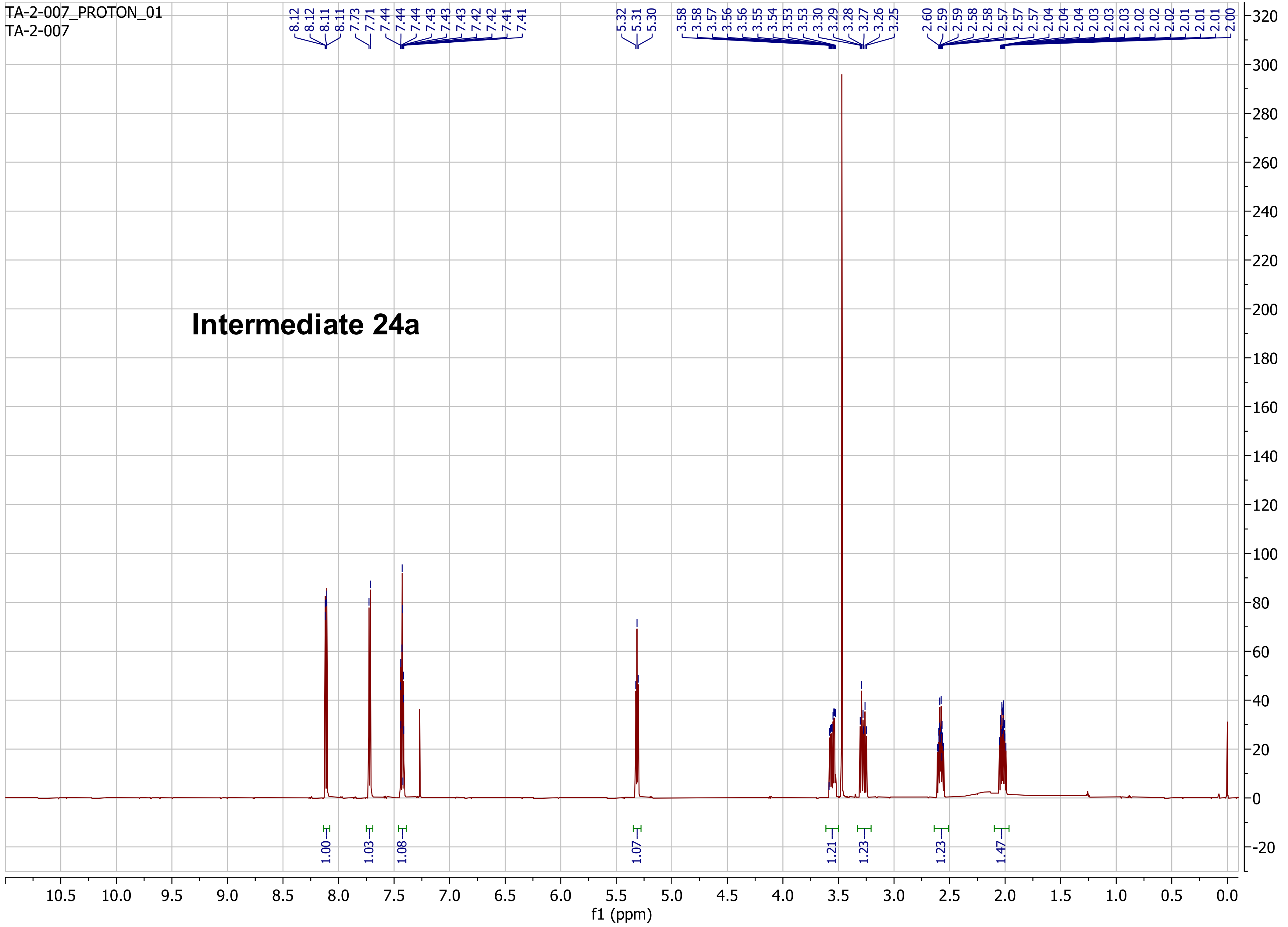
TA-2-007_PROTON_01
TA-2-007
Intermediate 24a
8.12 8.12 8.11 8.11 7.73 7.71 7.44 7.44 7.44 7.43 7.43 7.43 7.42 7.42 7.41 7.41
5.32 5.31 5.30
3.58 3.58 3.57 3.56 3.56 3.55 3.54 3.53 3.53 3.30 3.29 3.28 3.27 3.26 3.25
2.60 2.59 2.59 2.58 2.58 2.57 2.57 2.57 2.04 2.04 2.04 2.03 2.03 2.03 2.02 2.02 2.02 2.01 2.01 2.01 2.00
1.00 1.03 1.08 1.07 1.21 1.23 1.23 1.47
f1 (ppm)

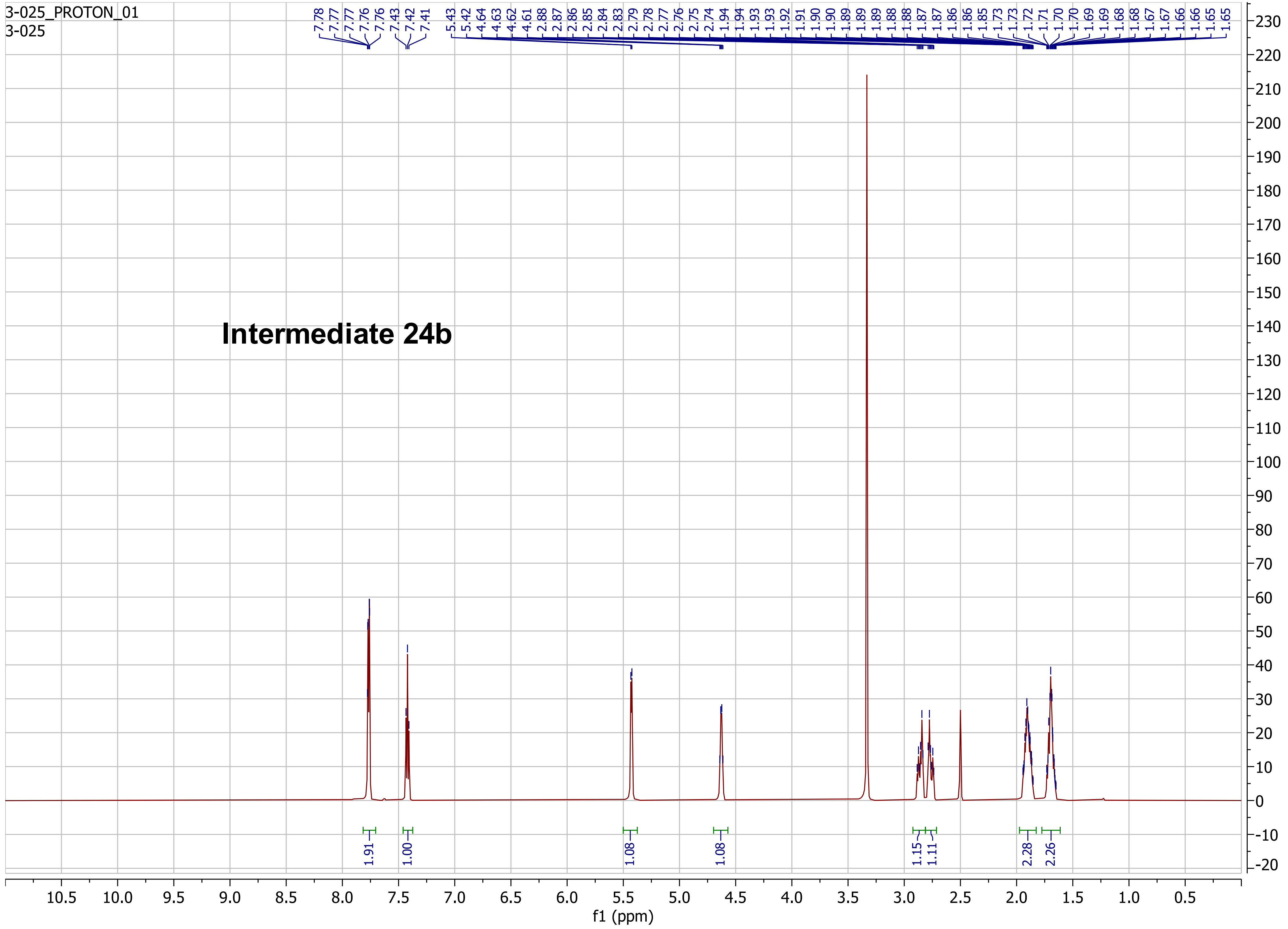

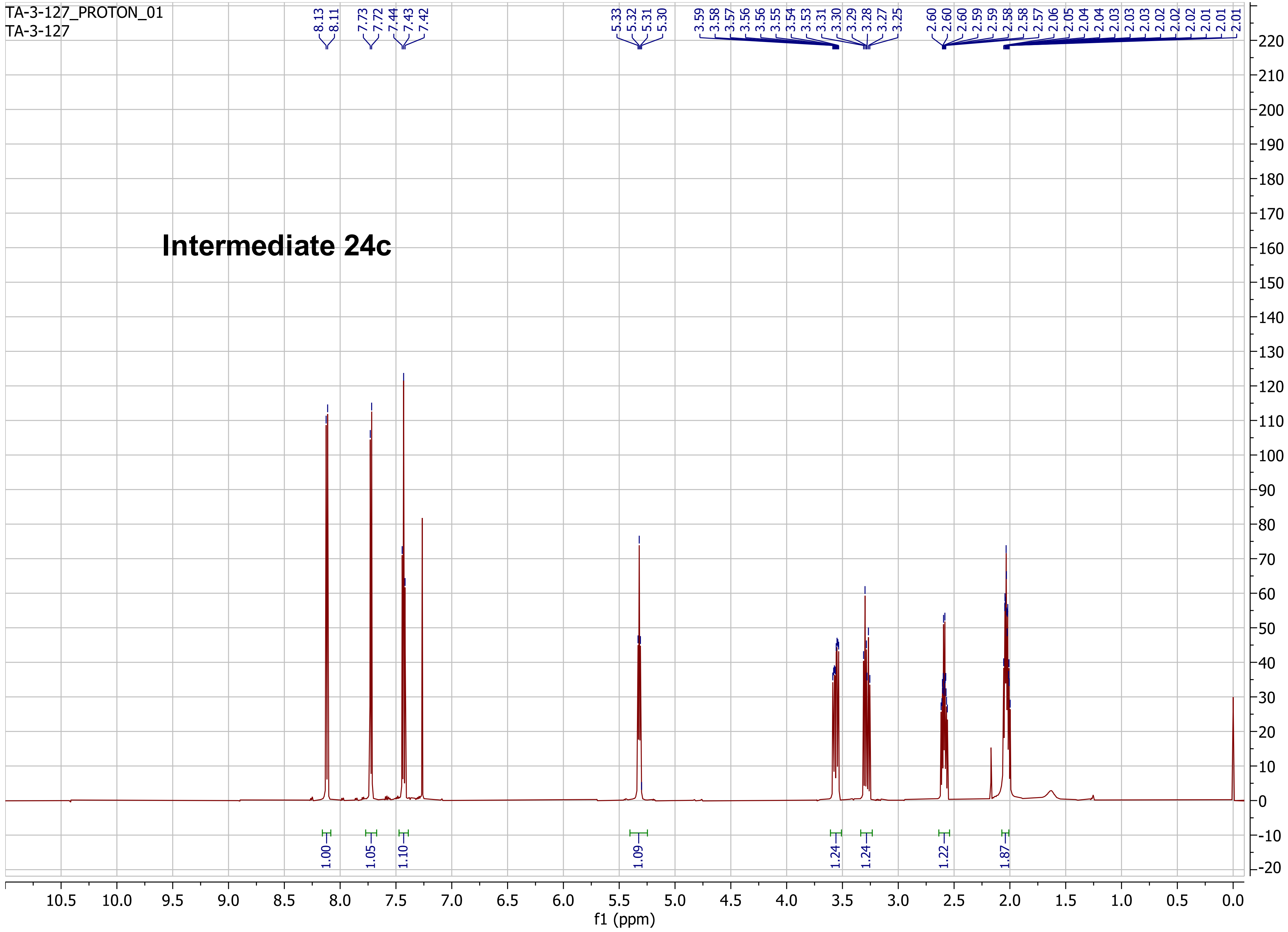

TA-3-127_PROTON_01
TA-3-127
Intermediate 24c
8.13
8.11
7.73
7.72
7.44
7.43
7.42
5.33
5.32
5.31
5.30
3.59
3.58
3.57
3.56
3.56
3.55
3.54
3.53
3.31
3.30
3.29
3.28
3.27
3.25
2.60
2.60
2.60
2.59
2.59
2.58
2.58
2.57
2.06
2.05
2.04
2.04
2.03
2.03
2.03
2.02
2.02
2.02
2.01
2.01
2.01
1.00
1.05
1.10
1.09
1.24
1.24
1.22
1.87
f1 (ppm)

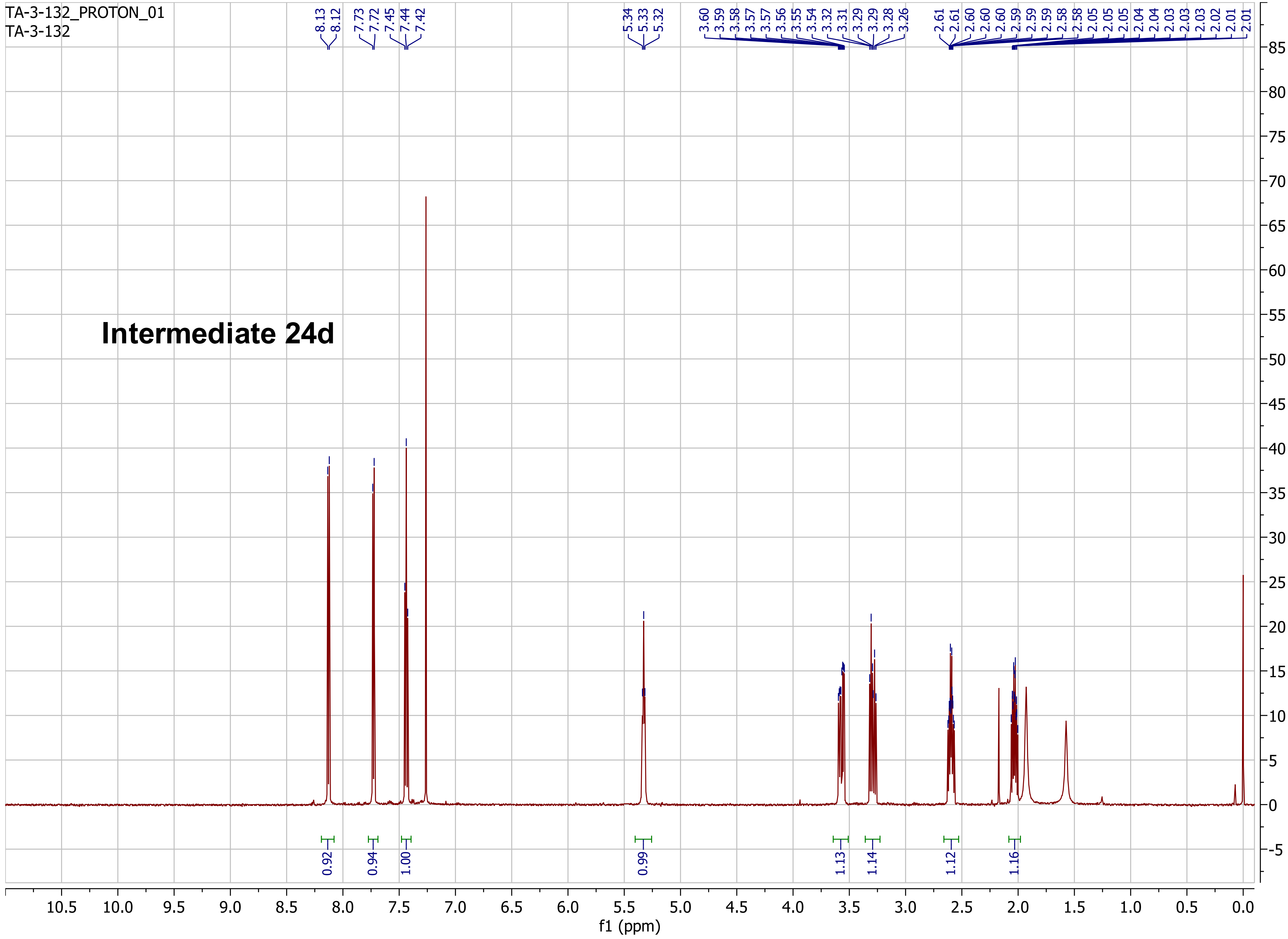
TA-3-132_PROTON_01
TA-3-132
Intermediate 24d
8.13
8.12
7.73
7.72
7.45
7.44
7.42
5.34
5.33
5.32
3.60
3.59
3.58
3.57
3.57
3.56
3.55
3.54
3.32
3.31
3.29
3.29
3.28
3.26
2.61
2.61
2.60
2.60
2.60
2.59
2.59
2.59
2.58
2.58
2.05
2.05
2.05
2.04
2.04
2.03
2.03
2.03
2.02
2.01
2.01
0.92
0.94
1.00
0.99
1.13
1.14
1.12
1.16
f1 (ppm)

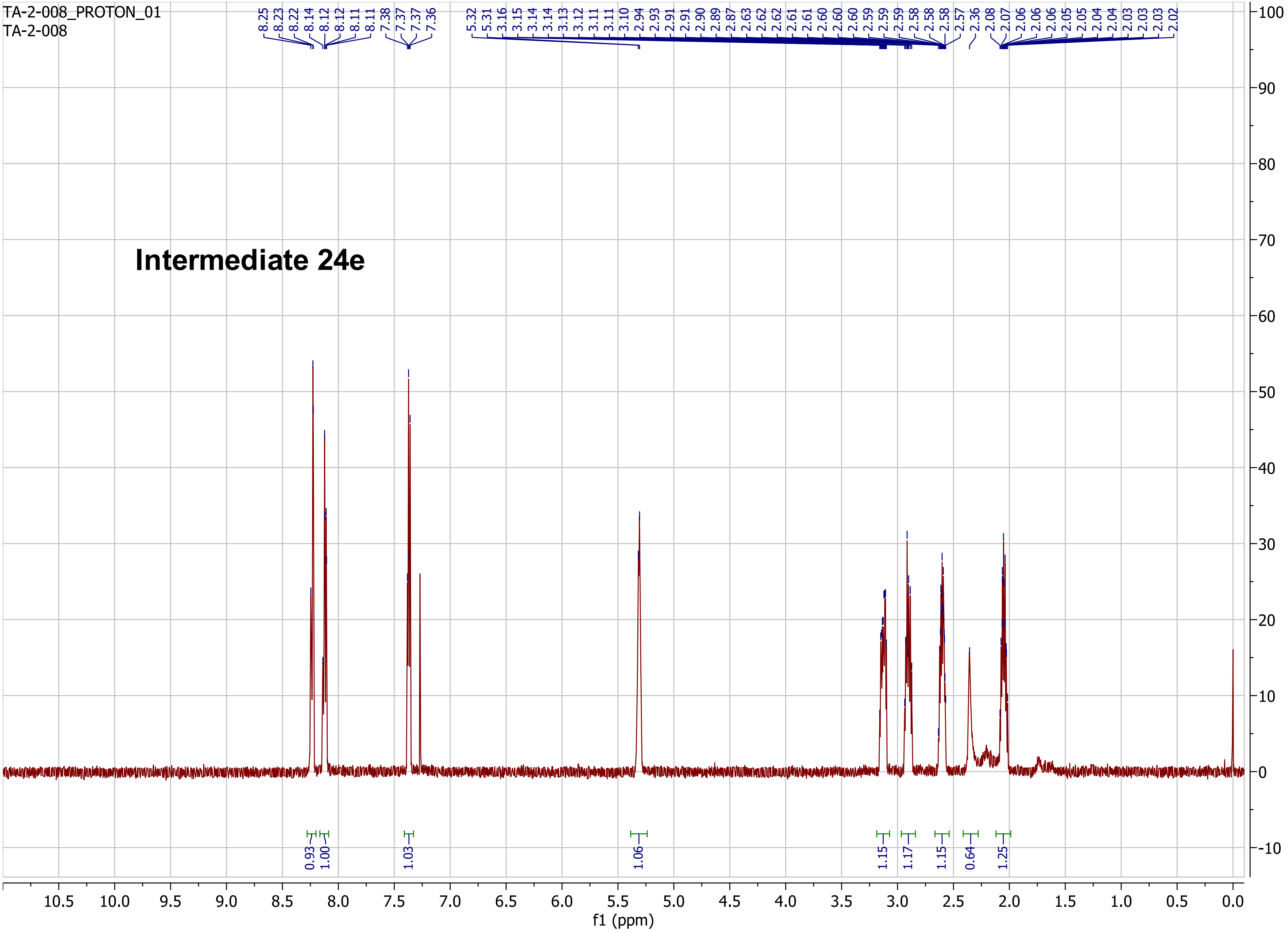

TA-2-008_PROTON_01
TA-2-008
Intermediate 24e
8.25 8.23 8.22 8.14 8.12 8.12 8.11 8.11 7.38 7.37 7.37 7.36
5.32 5.31 3.16 3.15 3.14 3.14 3.13 3.12 3.11 3.11 3.10 2.94 2.93 2.91 2.91 2.90 2.89 2.87 2.63 2.62 2.62 2.61 2.61 2.60 2.60 2.60 2.59 2.59 2.59 2.58 2.58 2.58 2.57 2.36 2.08 2.07 2.06 2.06 2.06 2.05 2.04 2.04 2.03 2.03 2.03 2.02
0.93 1.00 1.03 1.06 1.15 1.17 1.15 0.64 1.25
f1 (ppm)

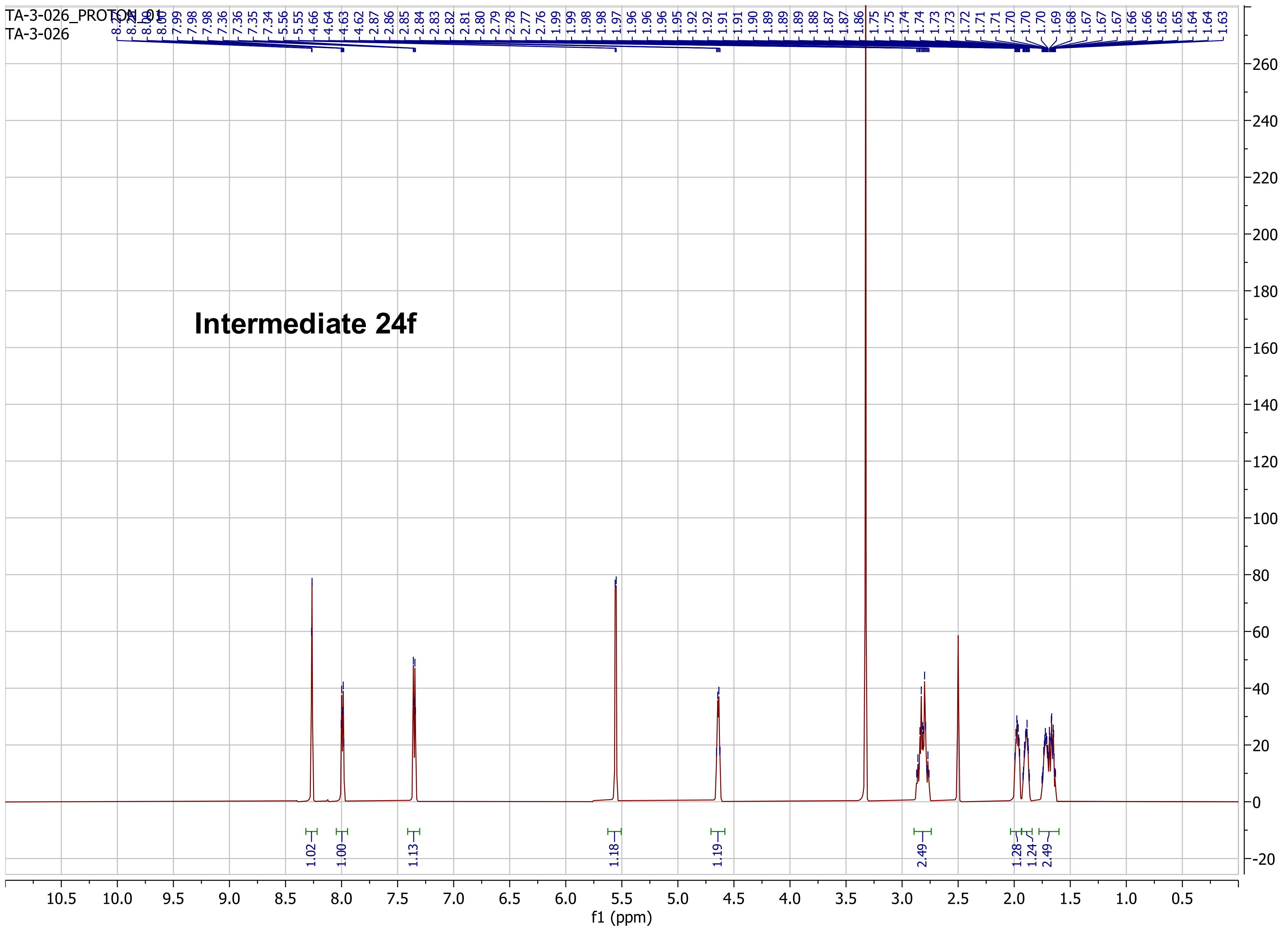
TA-3-026_PROTON_01
TA-3-026
Intermediate 24f
f1 (ppm)
1.02
1.00
1.13
1.18
1.19
2.49
1.28
1.24
2.49

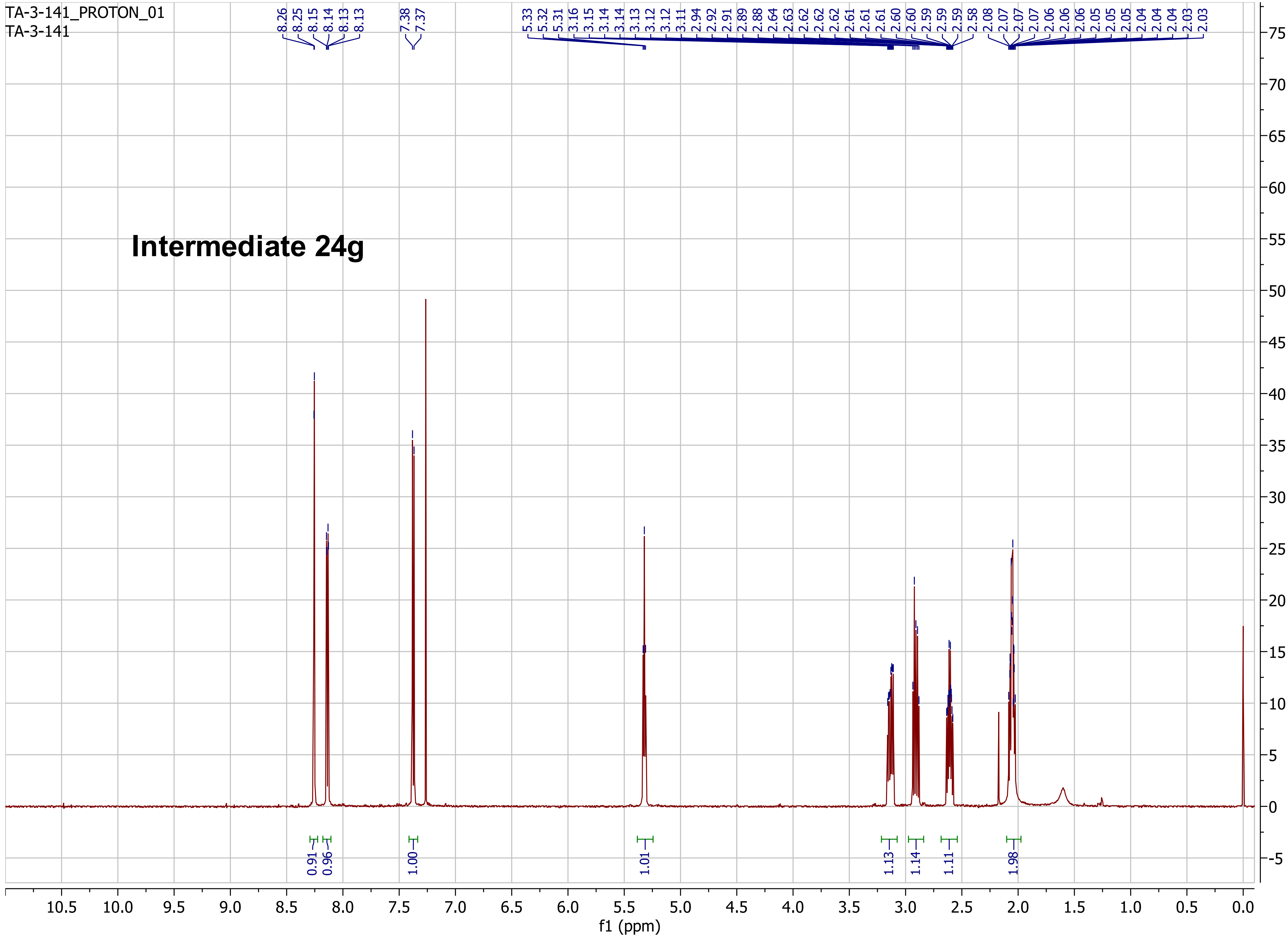
TA-3-141_PROTON_01
TA-3-141
Intermediate 24g
8.26 8.25 8.15 8.14 8.13 8.13
7.38 7.37
5.33 5.32 5.31 3.16 3.15 3.14 3.14 3.13 3.12 3.12 3.11 2.94 2.92 2.91 2.89 2.88 2.64 2.63 2.62 2.62 2.62 2.61 2.61 2.61 2.60 2.60 2.59 2.59 2.58 2.08 2.07 2.07 2.07 2.06 2.06 2.06 2.05 2.05 2.05 2.04 2.04 2.04 2.03 2.03
0.91 0.96 1.00 1.01 1.13 1.14 1.11 1.98
f1 (ppm)

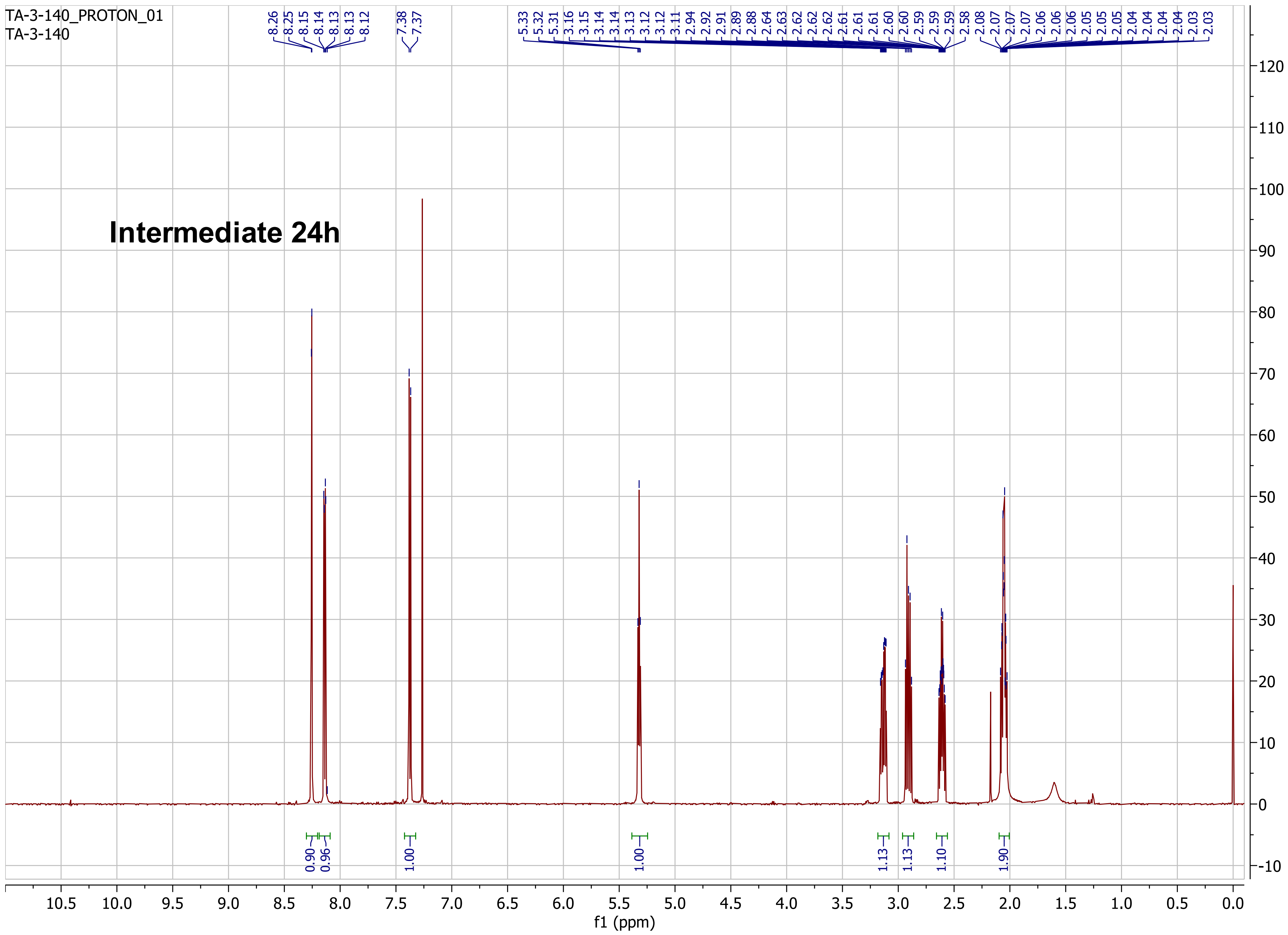

TA-3-140_PROTON_01
TA-3-140
Intermediate 24h
8.26 8.25 8.15 8.14 8.13 8.13 8.12
7.38 7.37
5.33 5.32 5.31 3.16 3.15 3.14 3.14 3.13 3.12 3.12 3.11 2.94 2.92 2.91 2.89 2.88 2.64 2.63 2.62 2.62 2.62 2.61 2.61 2.61 2.60 2.60 2.59 2.59 2.59 2.58 2.08 2.07 2.07 2.07 2.06 2.06 2.05 2.05 2.05 2.04 2.04 2.04 2.04 2.03 2.03
0.90 0.96 1.00 1.00 1.13 1.13 1.10 1.90
f1 (ppm)

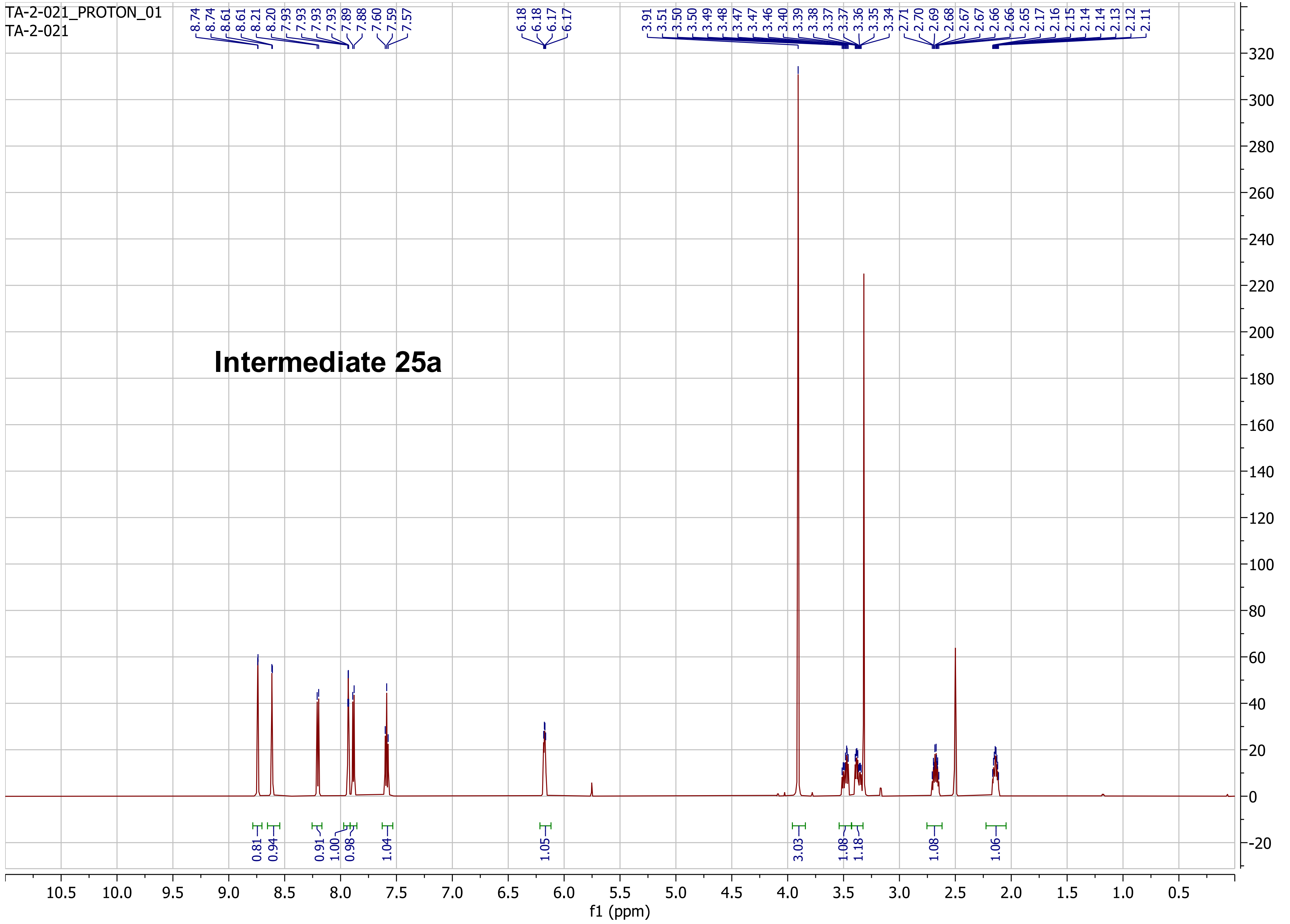
TA-2-021_PROTON_01
TA-2-021
Intermediate 25a
8.74 8.74 8.61 8.61 8.21 8.20 7.93 7.93 7.93 7.93 7.89 7.88 7.60 7.59 7.57
6.18 6.18 6.17 6.17
3.91 3.51 3.50 3.50 3.49 3.48 3.47 3.47 3.46 3.40 3.39 3.38 3.37 3.37 3.36 3.35 3.34 2.71 2.70 2.69 2.68 2.67 2.67 2.66 2.66 2.65 2.17 2.16 2.15 2.14 2.13 2.12 2.11
0.81 0.94 0.91 1.00 0.98 1.04 1.05 3.03 1.08 1.18 1.08 1.06
f1 (ppm)

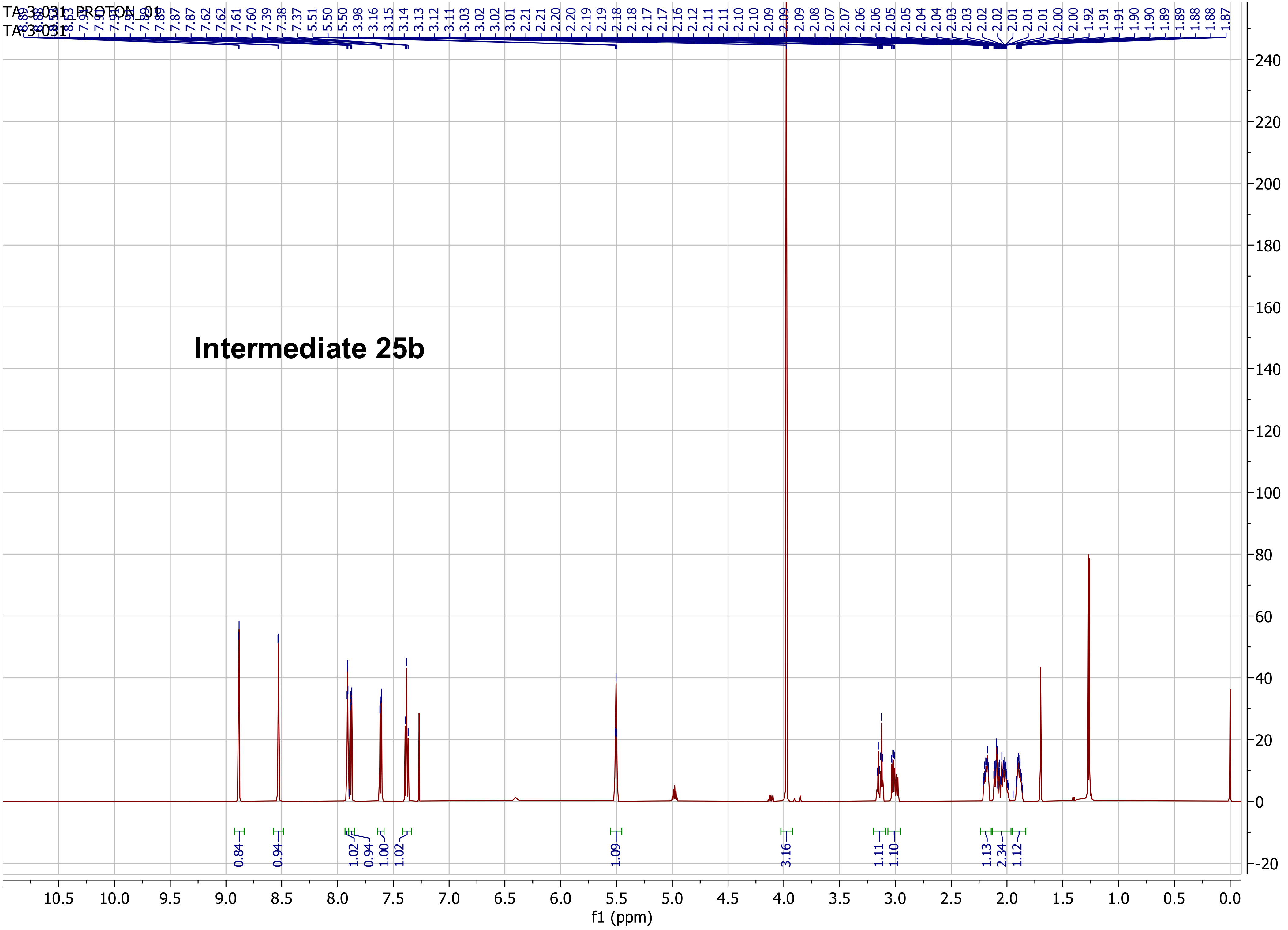
TA-3-031 PROTON 01
TA-3-031
7.87 7.87 7.62 7.62 7.61 7.60 7.39 7.38 7.37 5.51 5.50 5.50 3.98 3.16 3.15 3.14 3.13 3.12 3.11 3.03 3.02 3.02 3.01 2.21 2.21 2.20 2.20 2.19 2.19 2.18 2.18 2.17 2.17 2.16 2.12 2.11 2.11 2.10 2.10 2.09 2.09 2.09 2.08 2.07 2.07 2.06 2.06 2.05 2.05 2.04 2.04 2.03 2.03 2.02 2.02 2.01 2.01 2.01 2.00 1.92 1.91 1.91 1.90 1.90 1.89 1.89 1.88 1.88 1.87
Intermediate 25b
0.84 0.94 1.02 0.94 1.00 1.02 1.09 3.16 1.11 1.10 1.13 2.34 1.12
f1 (ppm)

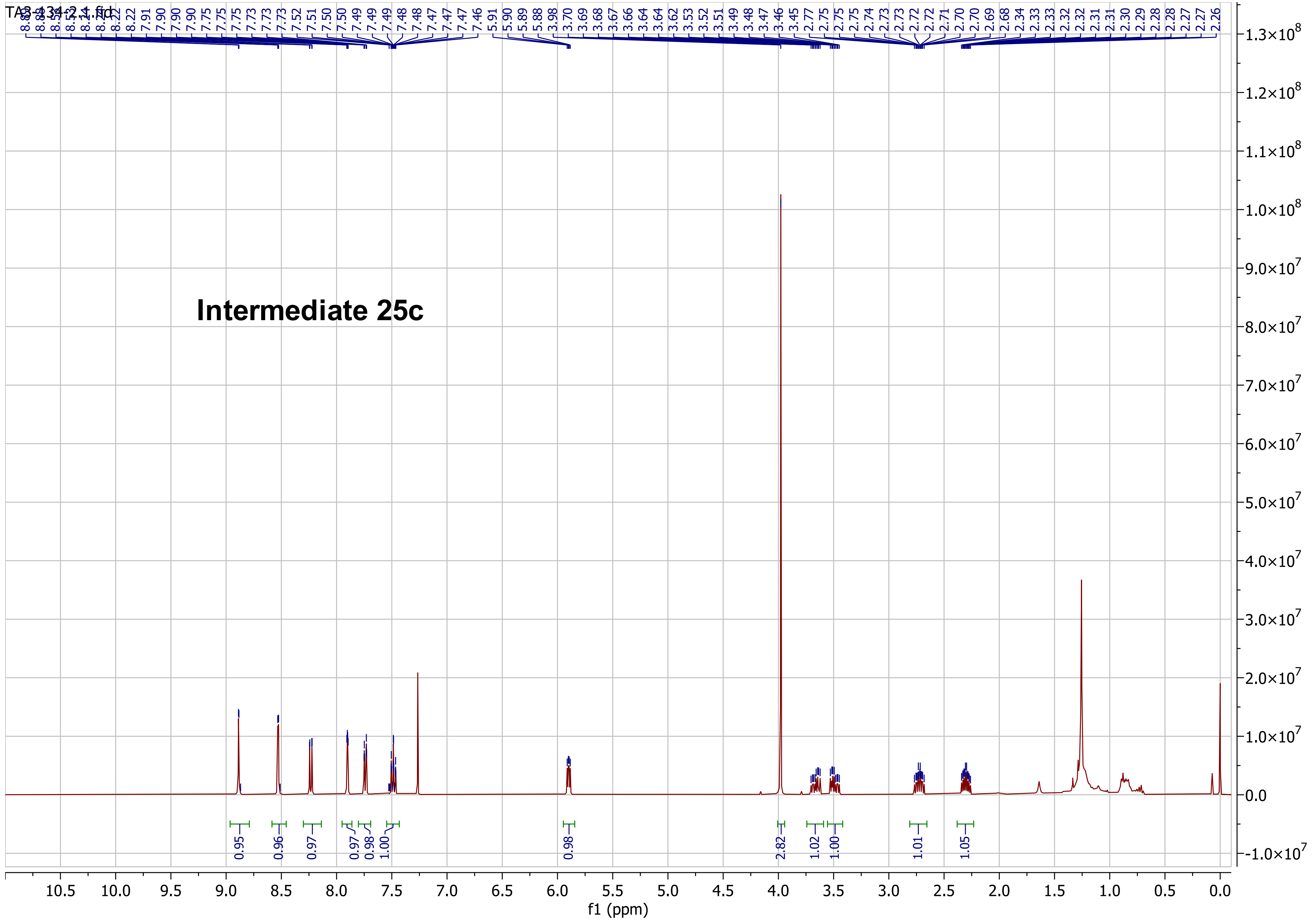

TA3-134-2.1.fid
Intermediate 25c
f1 (ppm)

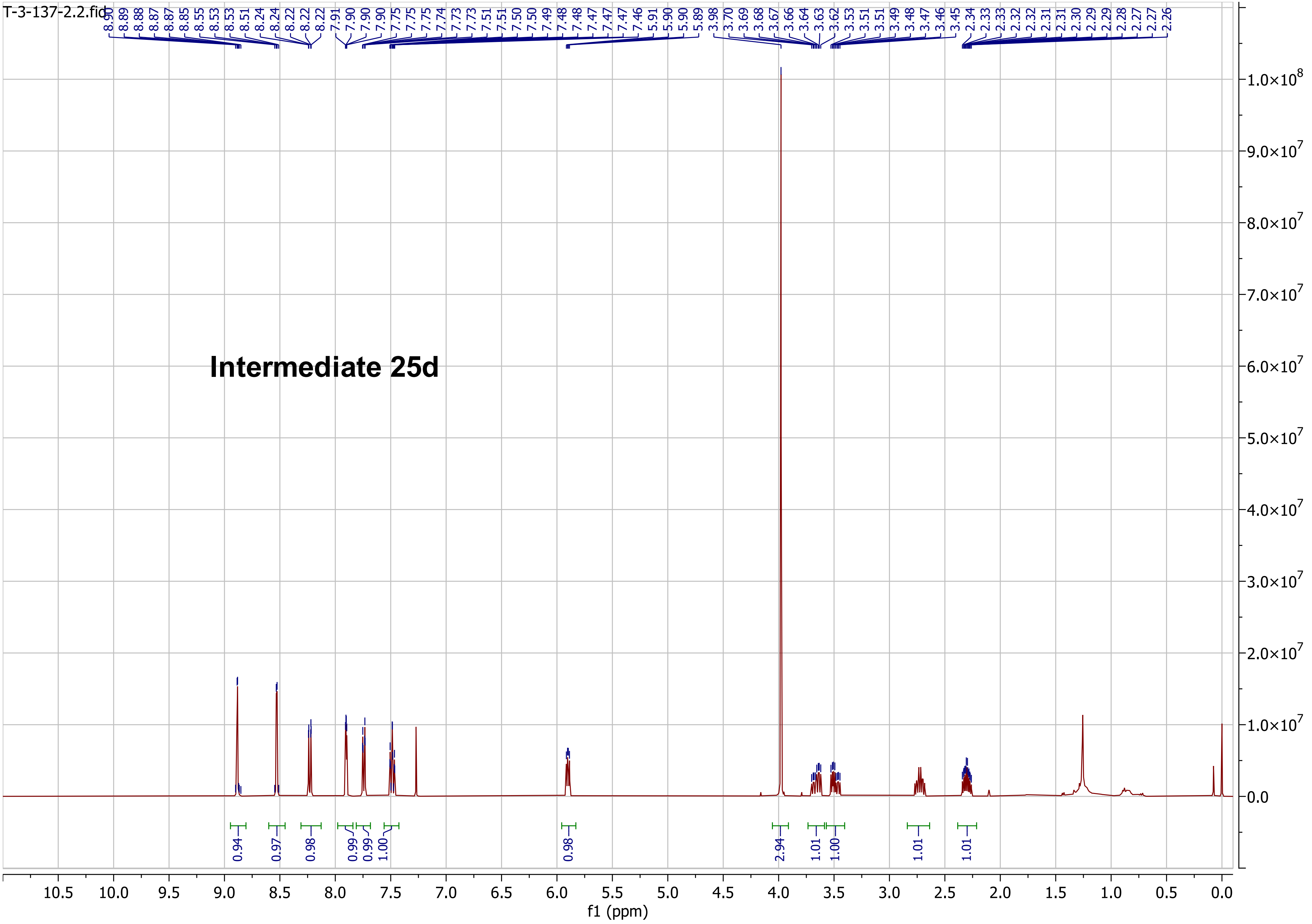
T-3-137-2.2.fid
Intermediate 25d
f1 (ppm)
8.90 8.89 8.88 8.87 8.87 8.85 8.55 8.53 8.53 8.51 8.24 8.24 8.22 8.22 8.22 7.91 7.90 7.90 7.90 7.75 7.75 7.75 7.74 7.73 7.73 7.51 7.51 7.50 7.50 7.49 7.48 7.48 7.47 7.47 7.46 5.91 5.90 5.90 5.89 3.98 3.70 3.69 3.68 3.67 3.66 3.64 3.63 3.62 3.53 3.51 3.51 3.49 3.48 3.47 3.46 3.45 2.34 2.33 2.33 2.32 2.32 2.31 2.31 2.30 2.29 2.29 2.28 2.27 2.27 2.26
0.94 0.97 0.98 0.99 0.99 1.00 0.98 2.94 1.01 1.00 1.01 1.01

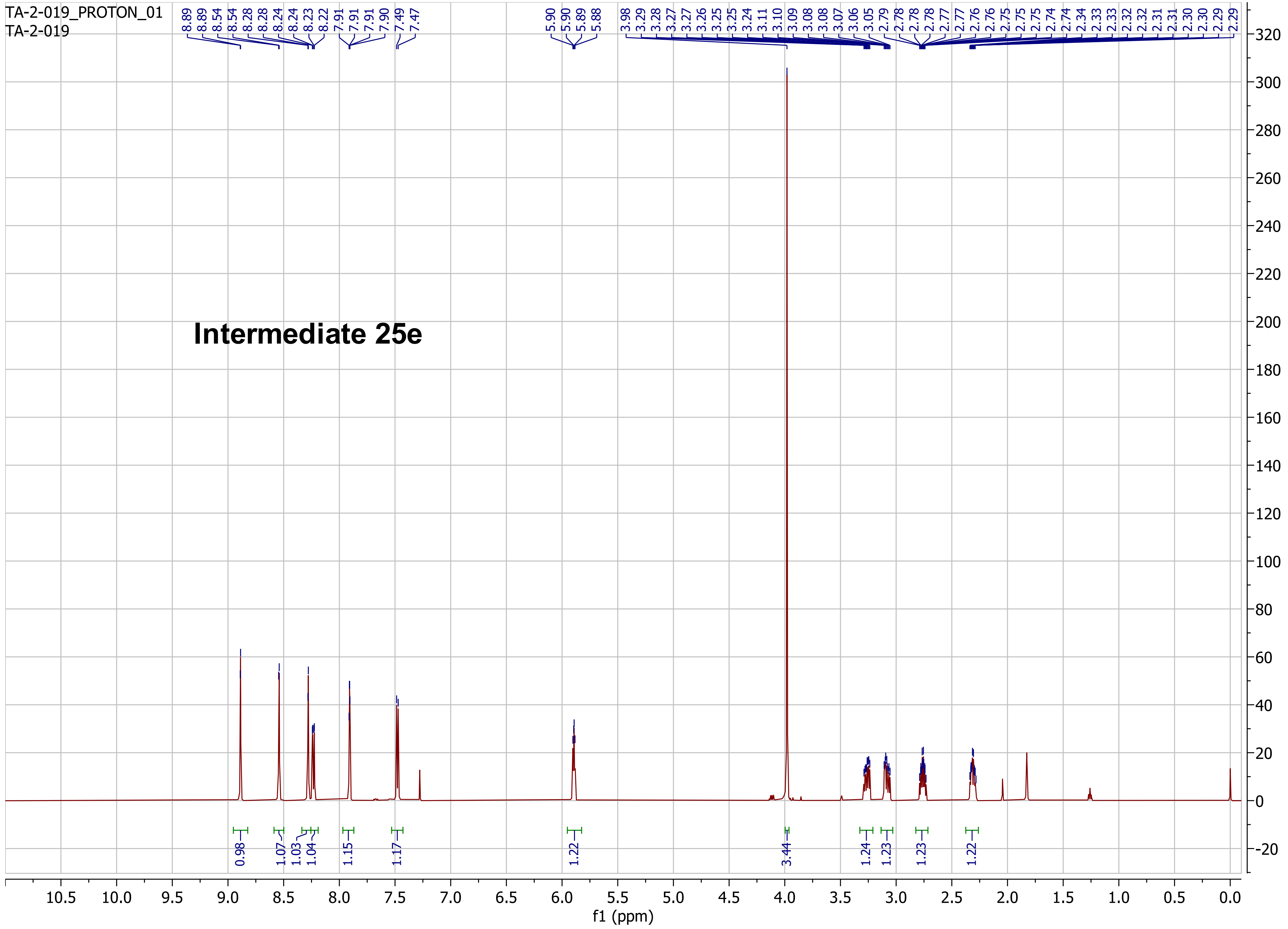

TA-2-019_PROTON_01
TA-2-019
Intermediate 25e
8.89 8.89 8.54 8.54 8.28 8.28 8.24 8.24 8.23 8.22 7.91 7.91 7.91 7.90 7.49 7.47
5.90 5.90 5.89 5.88
3.98 3.29 3.28 3.27 3.27 3.26 3.25 3.25 3.24 3.11 3.10 3.09 3.08 3.08 3.07 3.06 3.05 2.79 2.78 2.78 2.78 2.77 2.77 2.76 2.76 2.75 2.75 2.75 2.74 2.74 2.34 2.33 2.33 2.32 2.32 2.31 2.31 2.30 2.29 2.29
0.98 1.07 1.03 1.04 1.15 1.17 1.22 3.44 1.24 1.23 1.23 1.22
f1 (ppm)

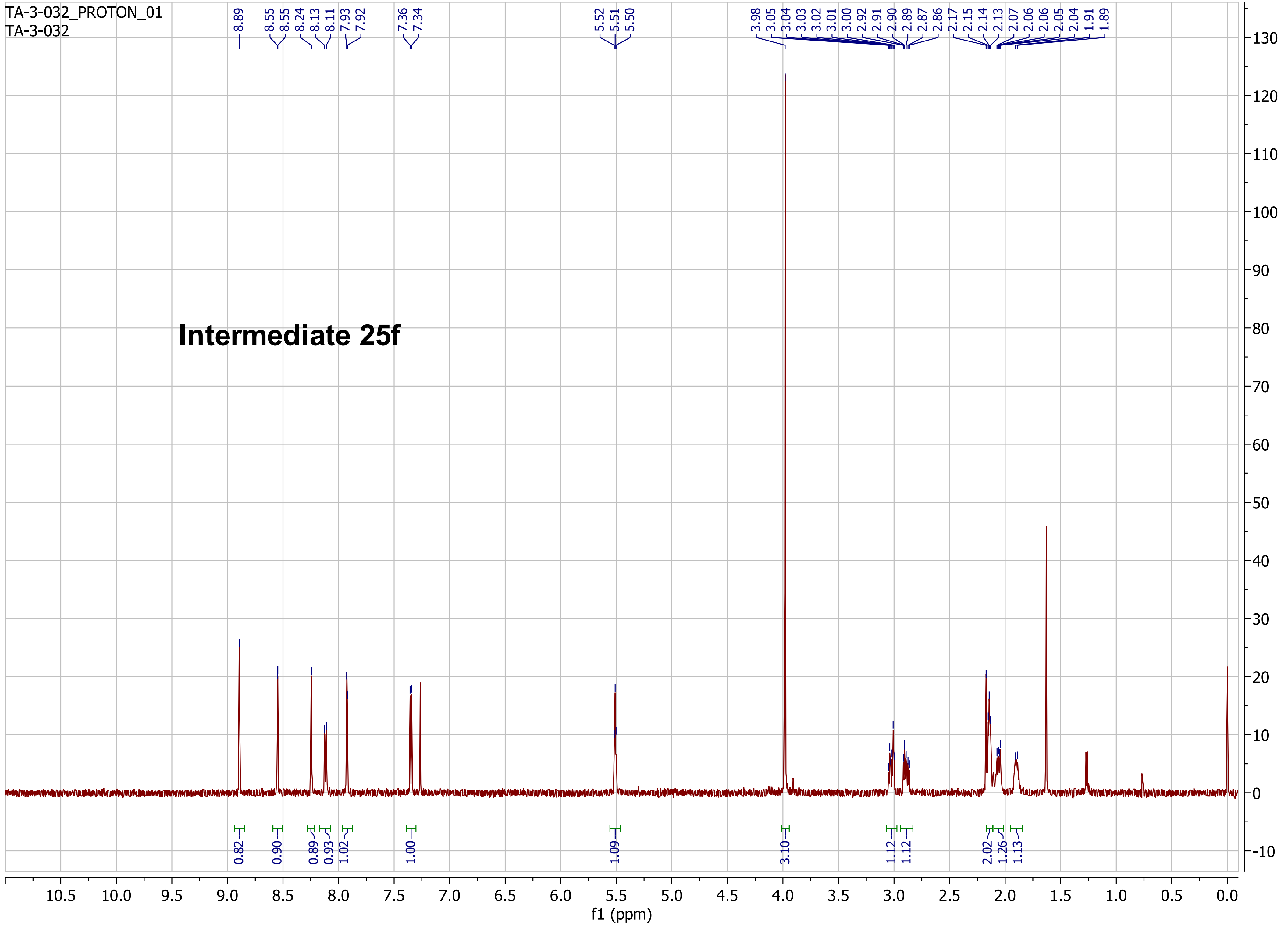
TA-3-032_PROTON_01
TA-3-032
Intermediate 25f
8.89 8.55 8.55 8.24 8.13 8.11 7.93 7.92 7.36 7.34
5.52 5.51 5.50
3.98 3.05 3.04 3.03 3.02 3.01 3.00 2.92 2.91 2.90 2.89 2.87 2.86 2.17 2.15 2.14 2.13 2.07 2.06 2.06 2.05 2.04 1.91 1.89
0.82 0.90 0.89 0.93 1.02 1.00 1.09 3.10 1.12 1.12 2.02 1.26 1.13
f1 (ppm)

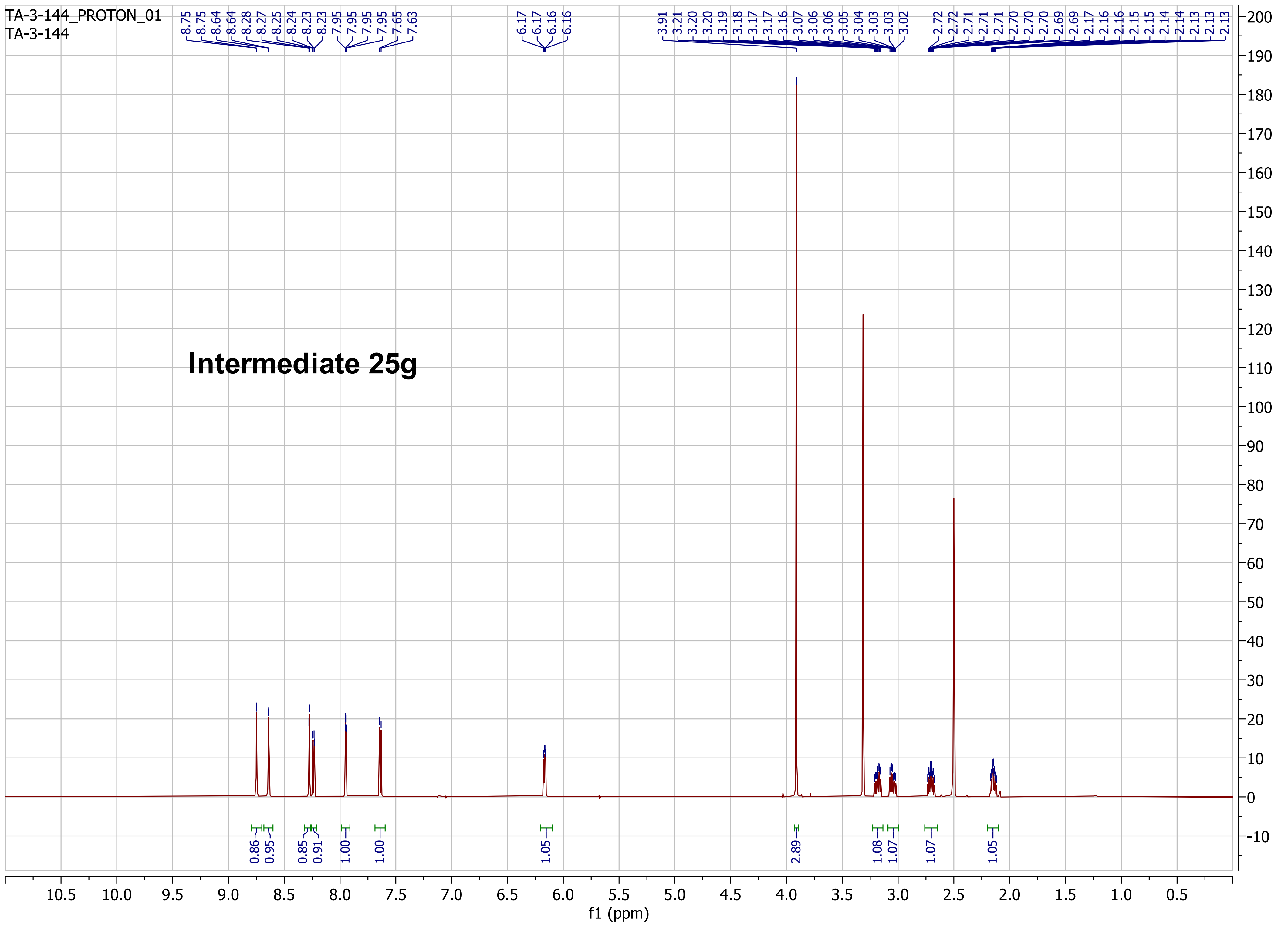

TA-3-144_PROTON_01
TA-3-144
Intermediate 25g
f1 (ppm)

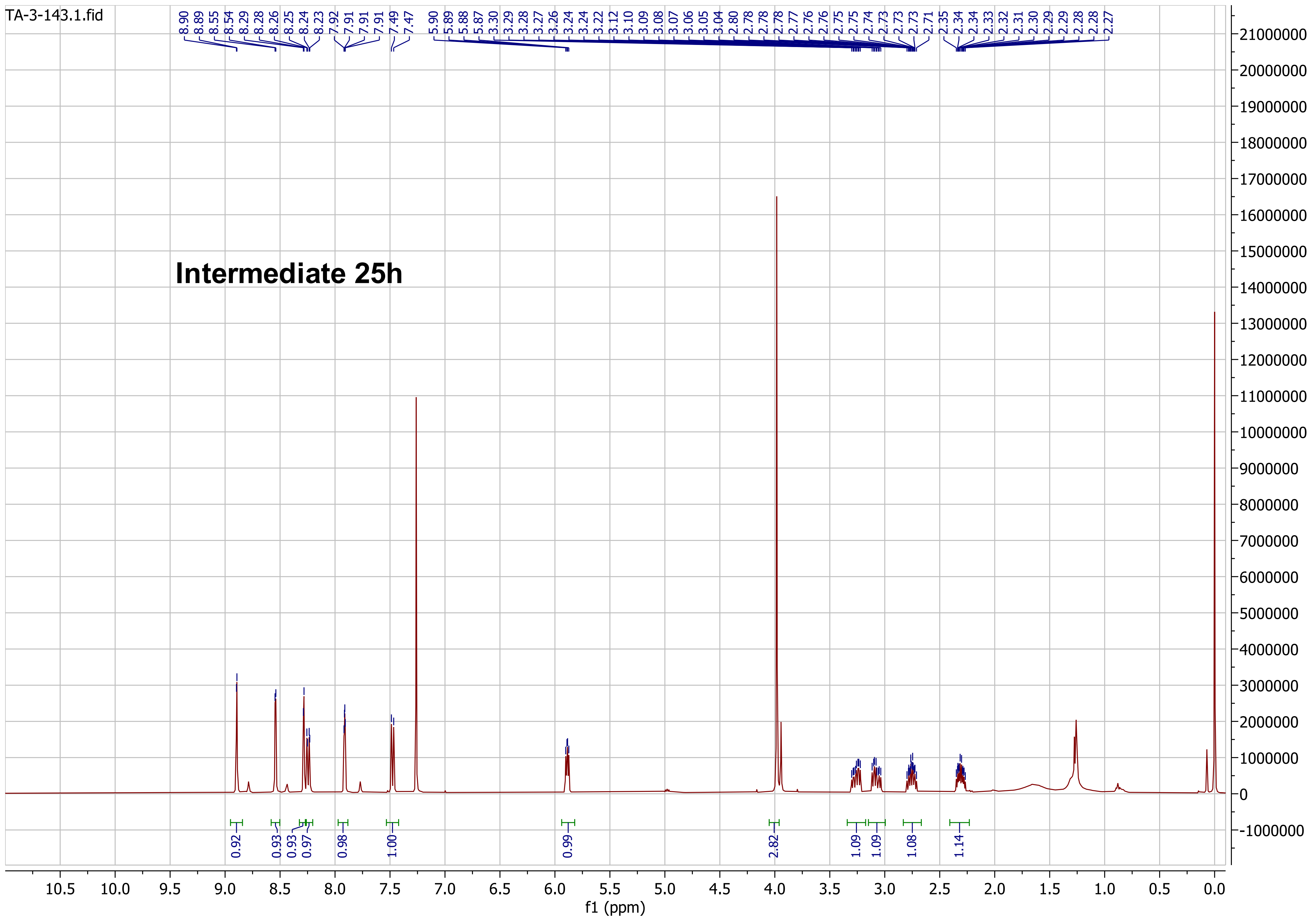
TA-3-143.1.fid
Intermediate 25h
8.90
8.89
8.55
8.54
8.29
8.28
8.26
8.25
8.24
8.23
7.92
7.91
7.91
7.91
7.49
7.47
5.90
5.89
5.88
5.87
3.30
3.29
3.28
3.27
3.26
3.24
3.24
3.22
3.12
3.10
3.09
3.08
3.07
3.06
3.05
3.04
2.80
2.78
2.78
2.78
2.77
2.76
2.76
2.75
2.75
2.74
2.73
2.73
2.73
2.71
2.35
2.34
2.34
2.33
2.32
2.31
2.30
2.29
2.29
2.28
2.28
2.27
0.92
0.93
0.93
0.97
0.98
1.00
0.99
2.82
1.09
1.09
1.08
1.14
f1 (ppm)

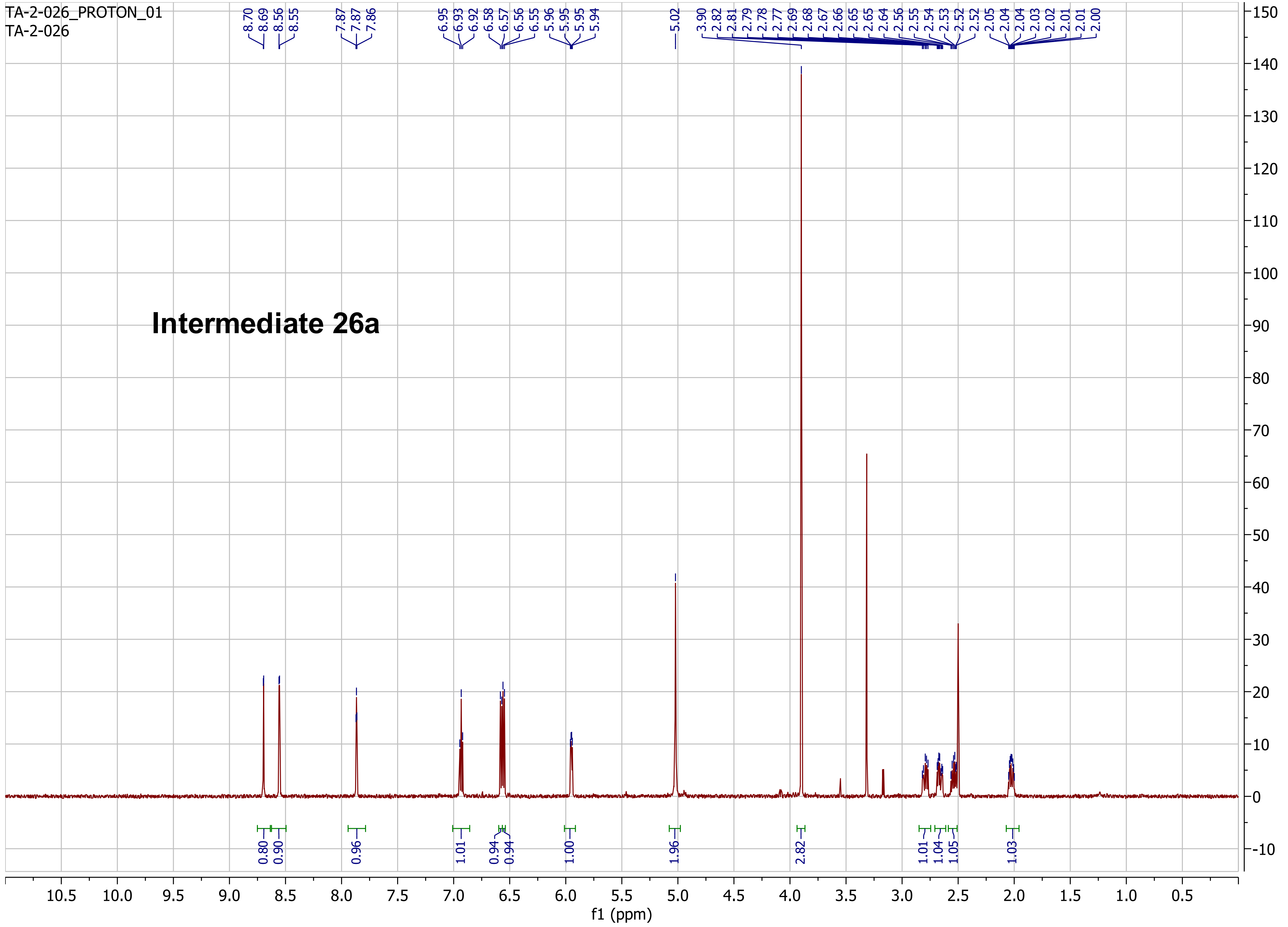
TA-2-026_PROTON_01
TA-2-026
Intermediate 26a
f1 (ppm)

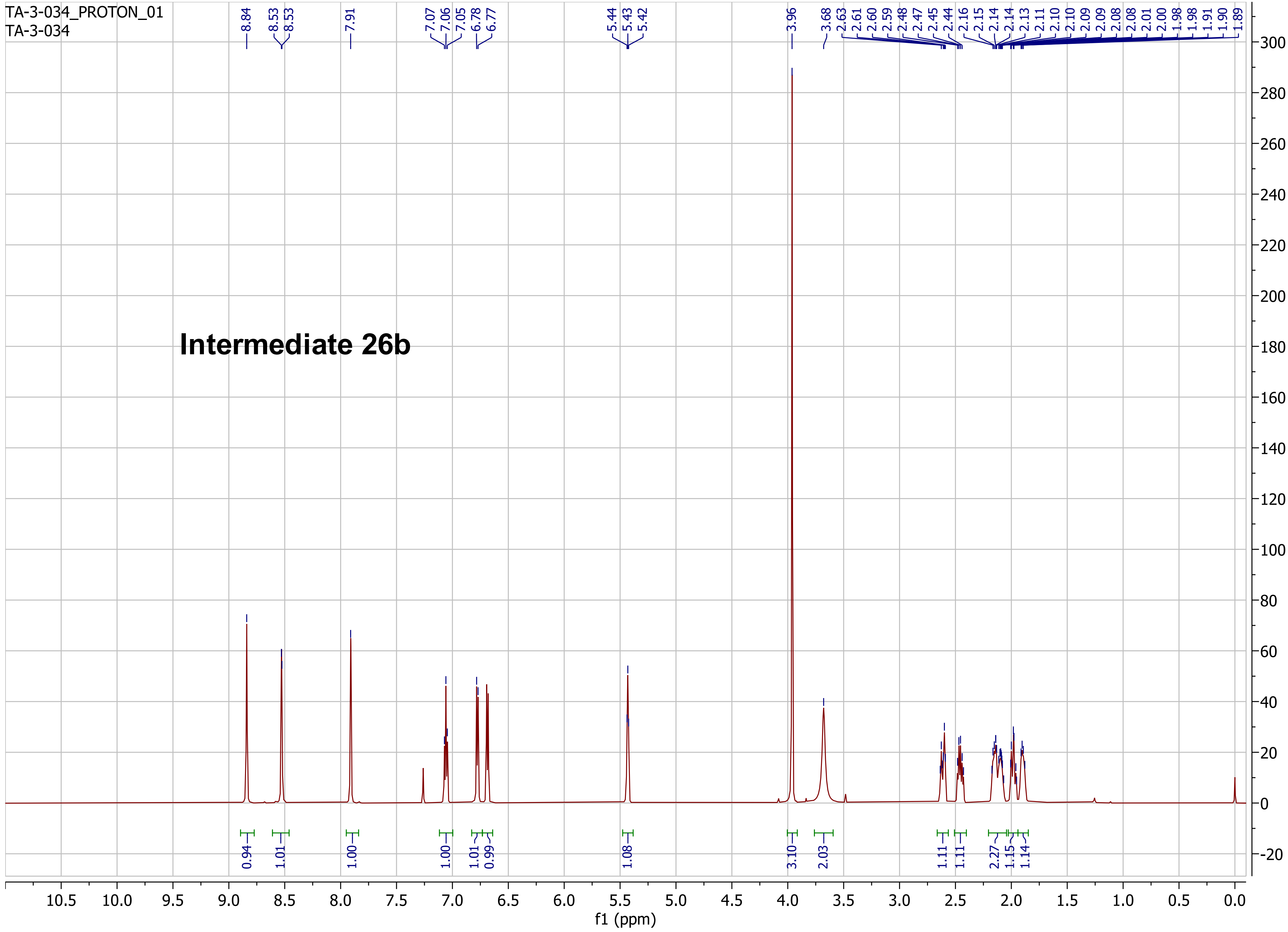

TA-3-034_PROTON_01
TA-3-034
Intermediate 26b
8.84
8.53
8.53
7.91
7.07
7.06
7.05
6.78
6.77
5.44
5.43
5.42
3.96
3.68
2.63
2.61
2.60
2.59
2.48
2.47
2.45
2.44
2.16
2.15
2.14
2.14
2.13
2.11
2.10
2.10
2.09
2.09
2.08
2.08
2.01
2.00
1.98
1.98
1.91
1.90
1.89
0.94
1.01
1.00
1.00
1.01
0.99
1.08
3.10
2.03
1.11
1.11
2.27
1.15
1.14
f1 (ppm)

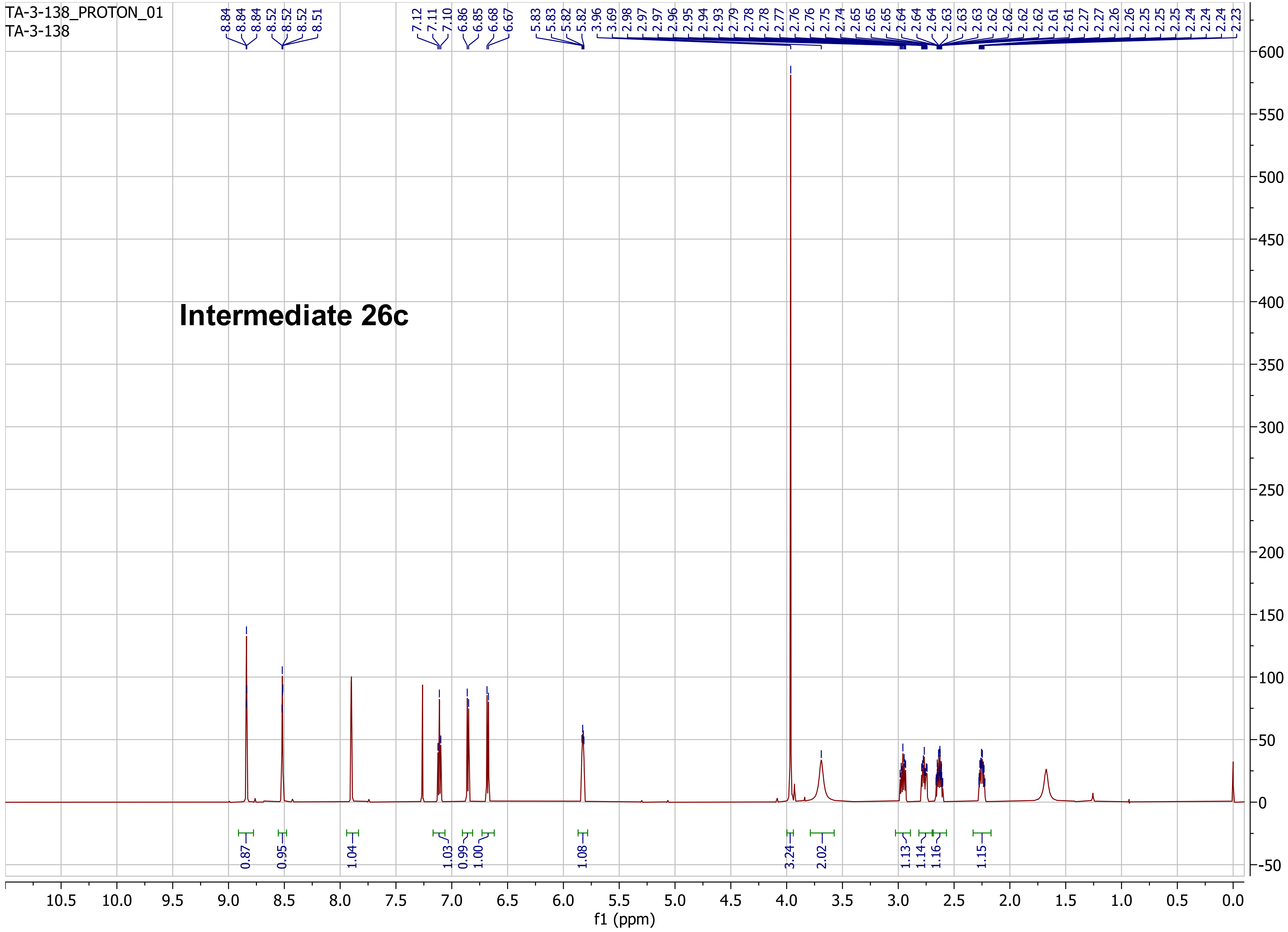

TA-3-138_PROTON_01
TA-3-138
Intermediate 26c
f1 (ppm)

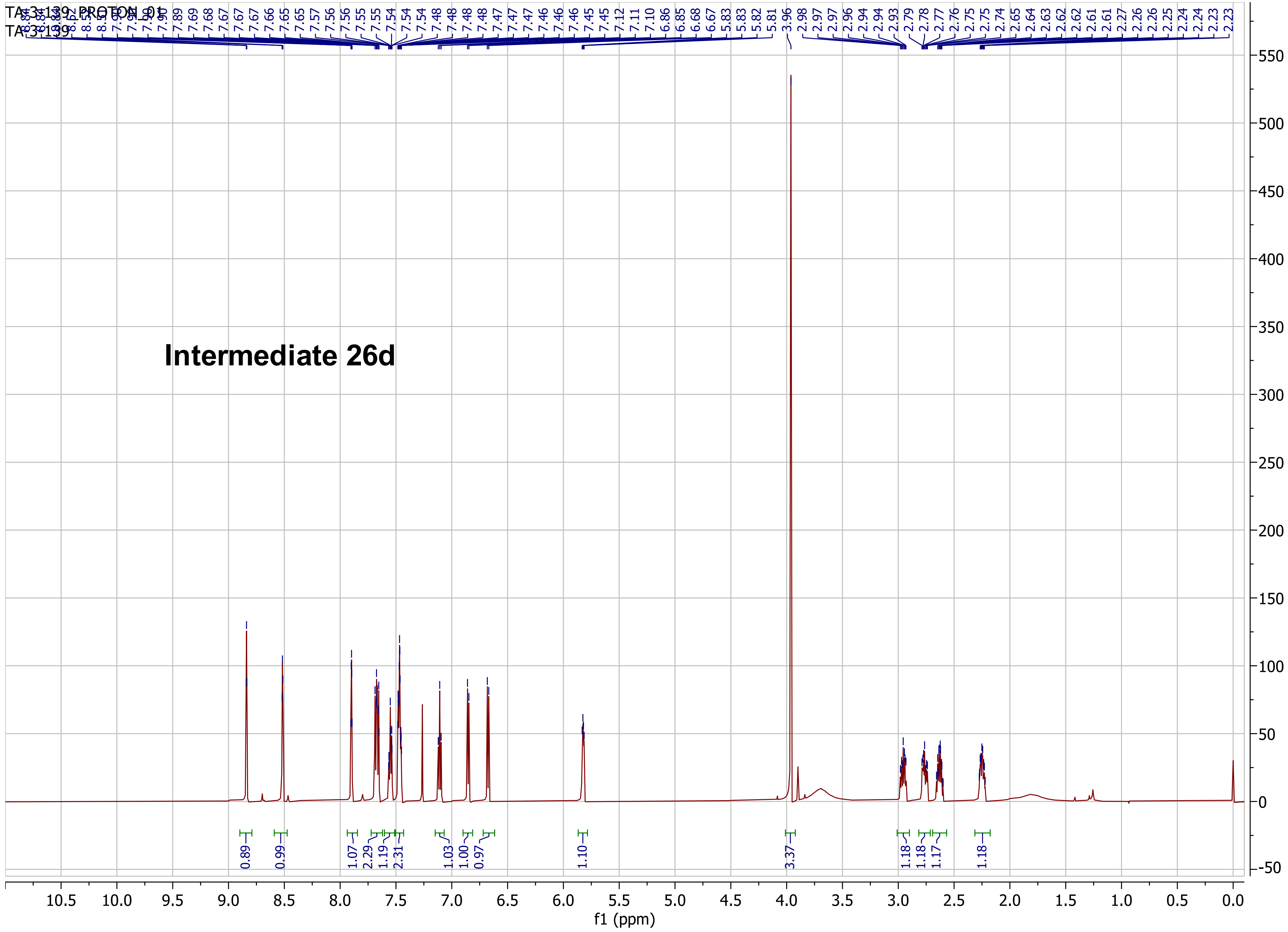
TA-3-139_PROTON_01
TA-3-139
Intermediate 26d
f1 (ppm)

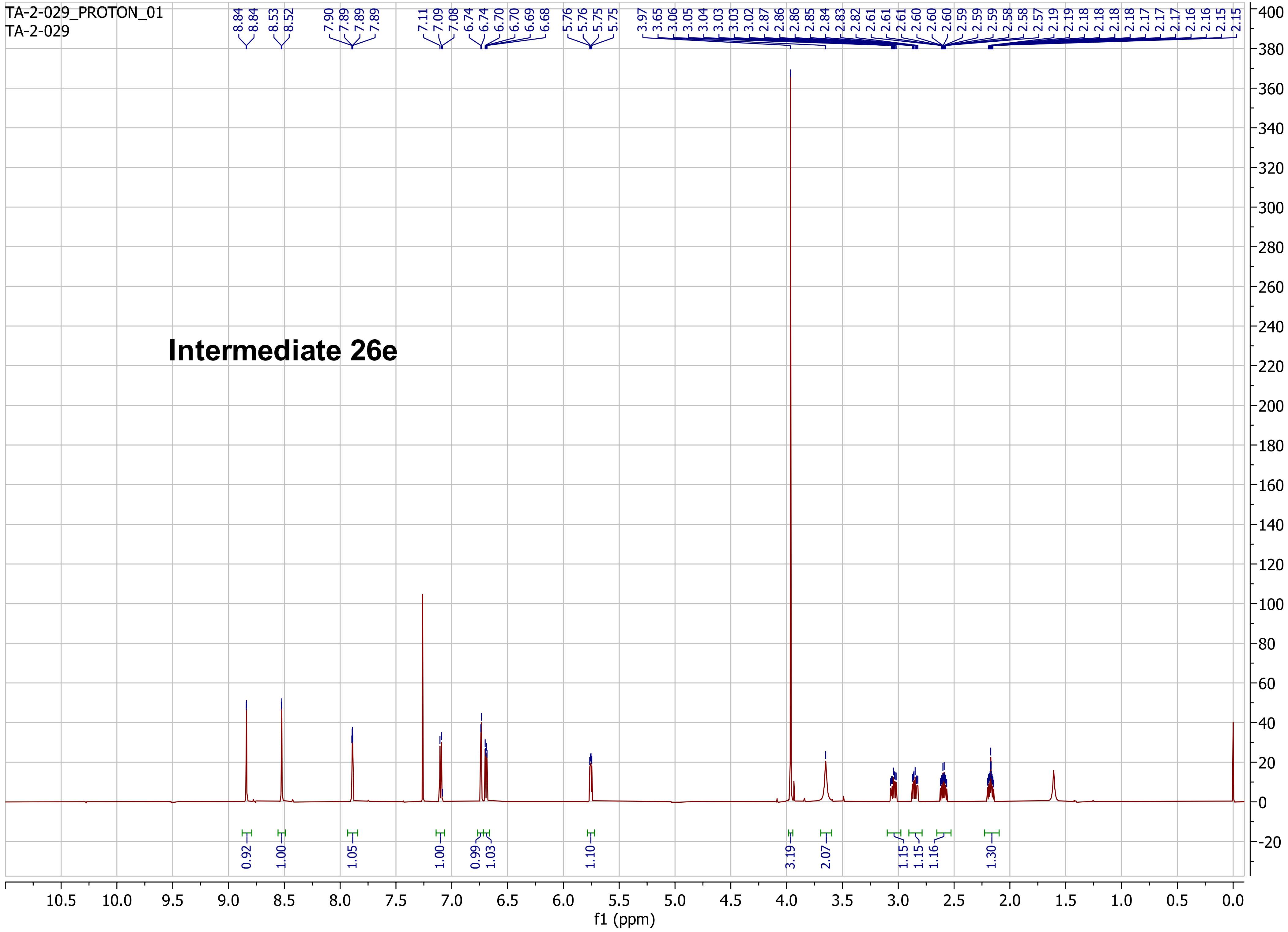
TA-2-029_PROTON_01
TA-2-029
Intermediate 26e
f1 (ppm)

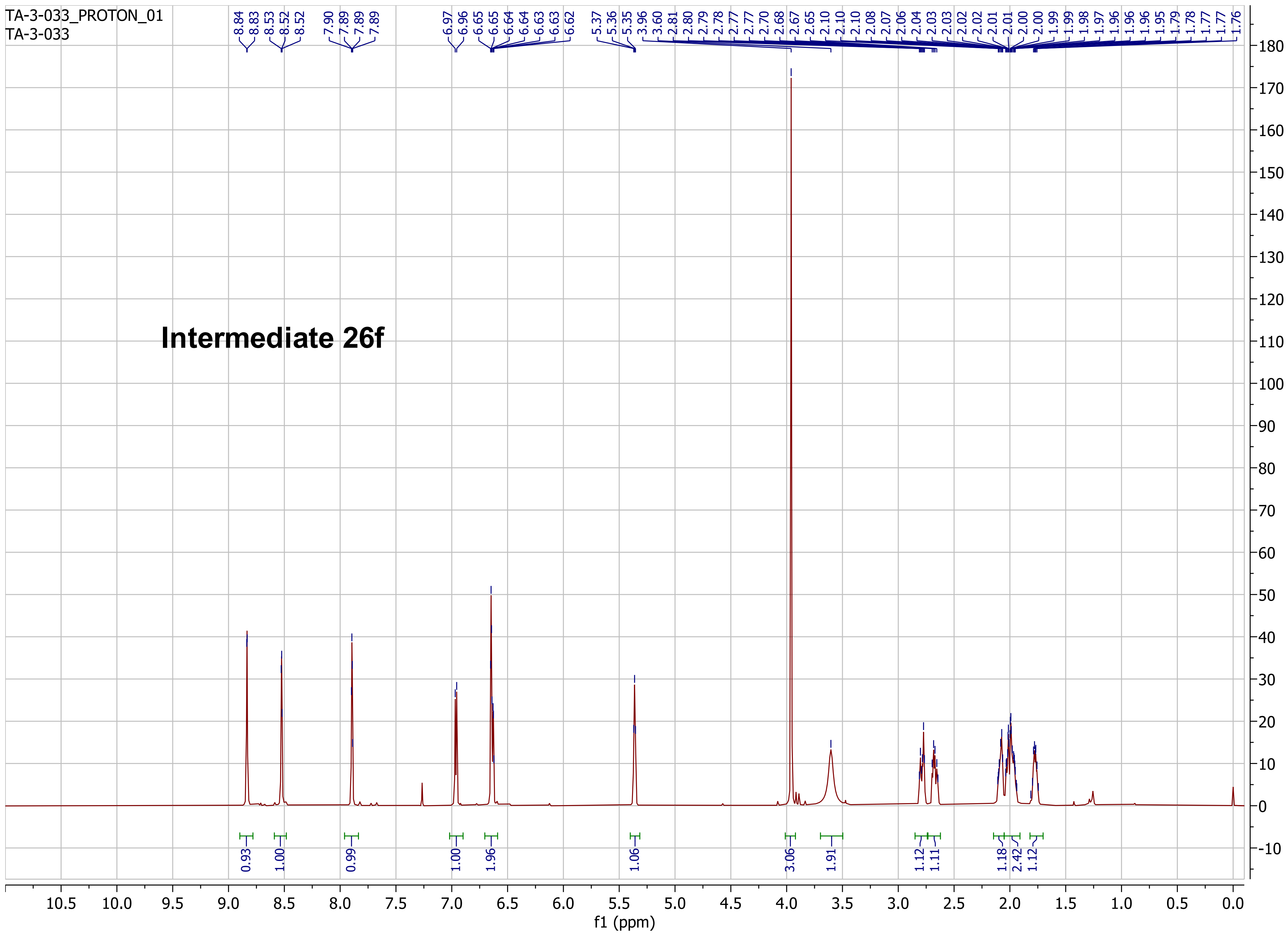
TA-3-033_PROTON_01
TA-3-033
Intermediate 26f
f1 (ppm)

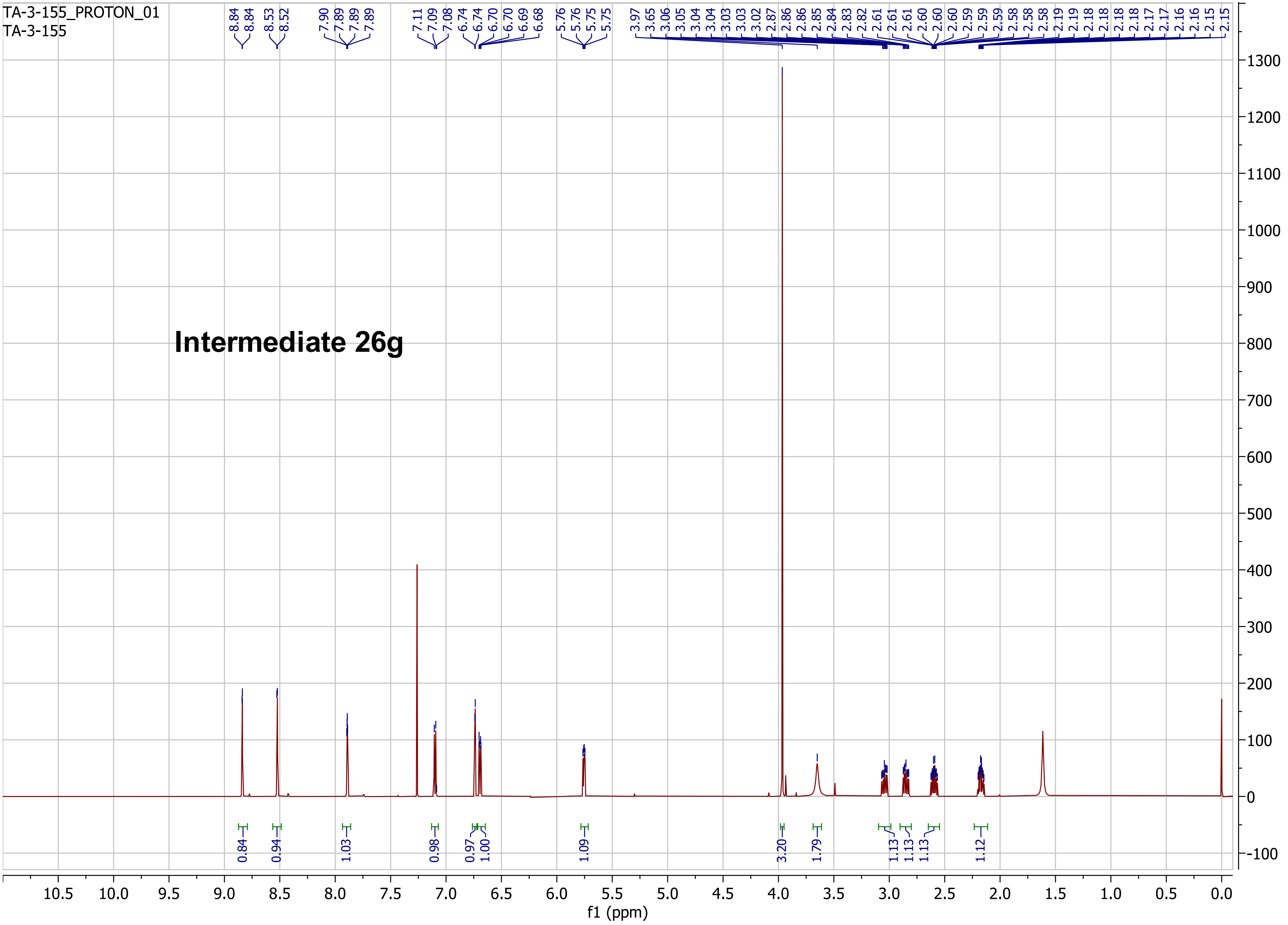
TA-3-155_PROTON_01
TA-3-155
Intermediate 26g
8.84 8.84 8.53 8.52 7.90 7.89 7.89 7.89 7.11 7.09 7.08 6.74 6.74 6.70 6.69 6.68 5.76 5.76 5.75 5.75 3.97 3.65 3.06 3.05 3.04 3.04 3.03 3.03 3.02 2.87 2.86 2.86 2.85 2.84 2.83 2.82 2.61 2.61 2.61 2.60 2.60 2.60 2.59 2.59 2.58 2.58 2.58 2.19 2.19 2.18 2.18 2.18 2.18 2.17 2.17 2.16 2.16 2.15 2.15
0.84 0.94 1.03 0.98 0.97 1.00 1.09 3.20 1.79 1.13 1.13 1.13 1.12
f1 (ppm)

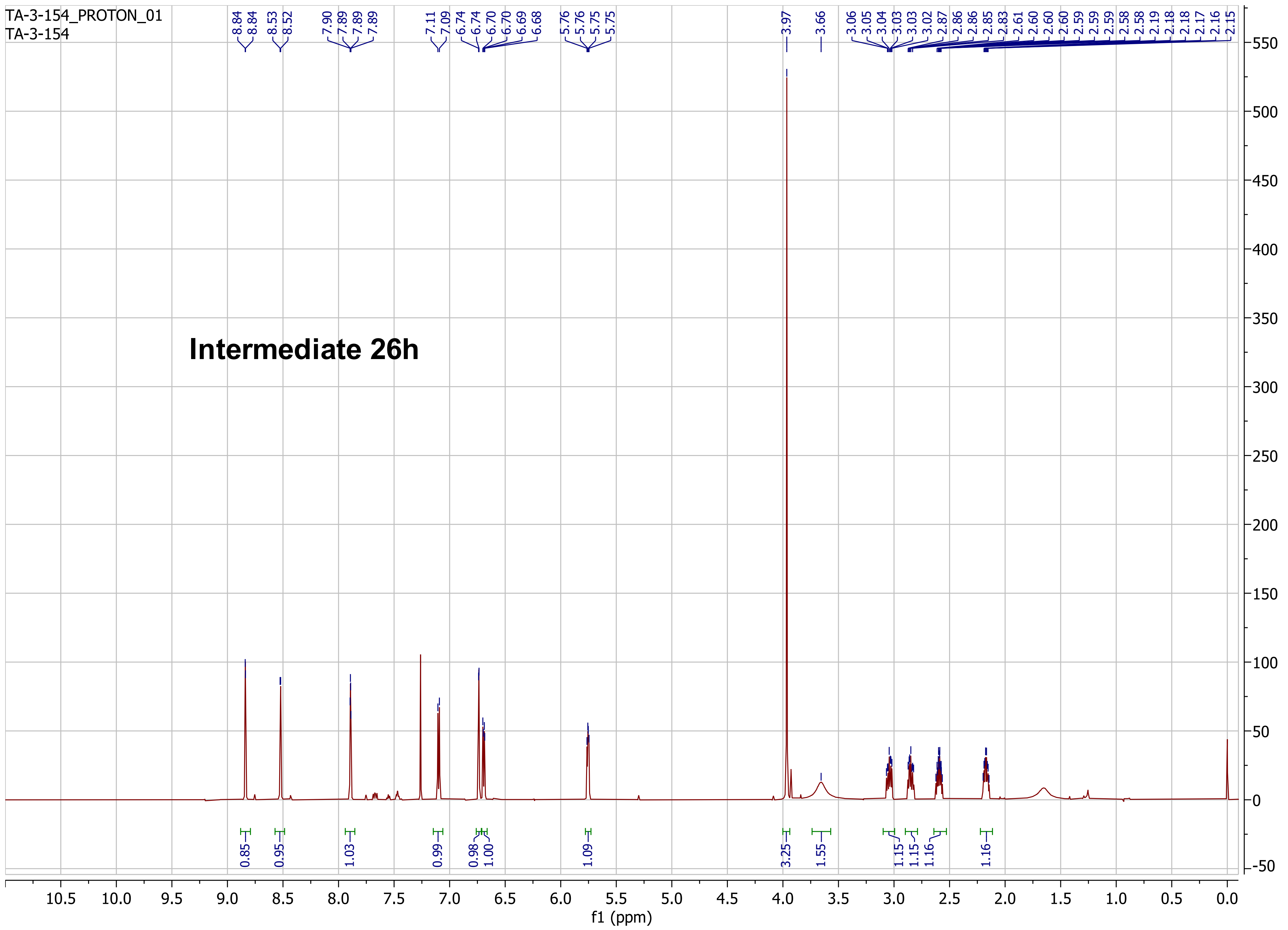
TA-3-154_PROTON_01
TA-3-154
Intermediate 26h
8.84 8.84 8.53 8.52 7.90 7.89 7.89 7.89 7.11 7.09 6.74 6.74 6.70 6.70 6.69 6.68 5.76 5.76 5.75 5.75 3.97 3.66 3.06 3.05 3.04 3.03 3.03 3.02 2.87 2.86 2.86 2.85 2.83 2.61 2.60 2.60 2.60 2.59 2.59 2.59 2.58 2.58 2.19 2.18 2.18 2.17 2.16 2.15
0.85 0.95 1.03 0.99 0.98 1.00 1.09 3.25 1.55 1.15 1.15 1.16 1.16
f1 (ppm)

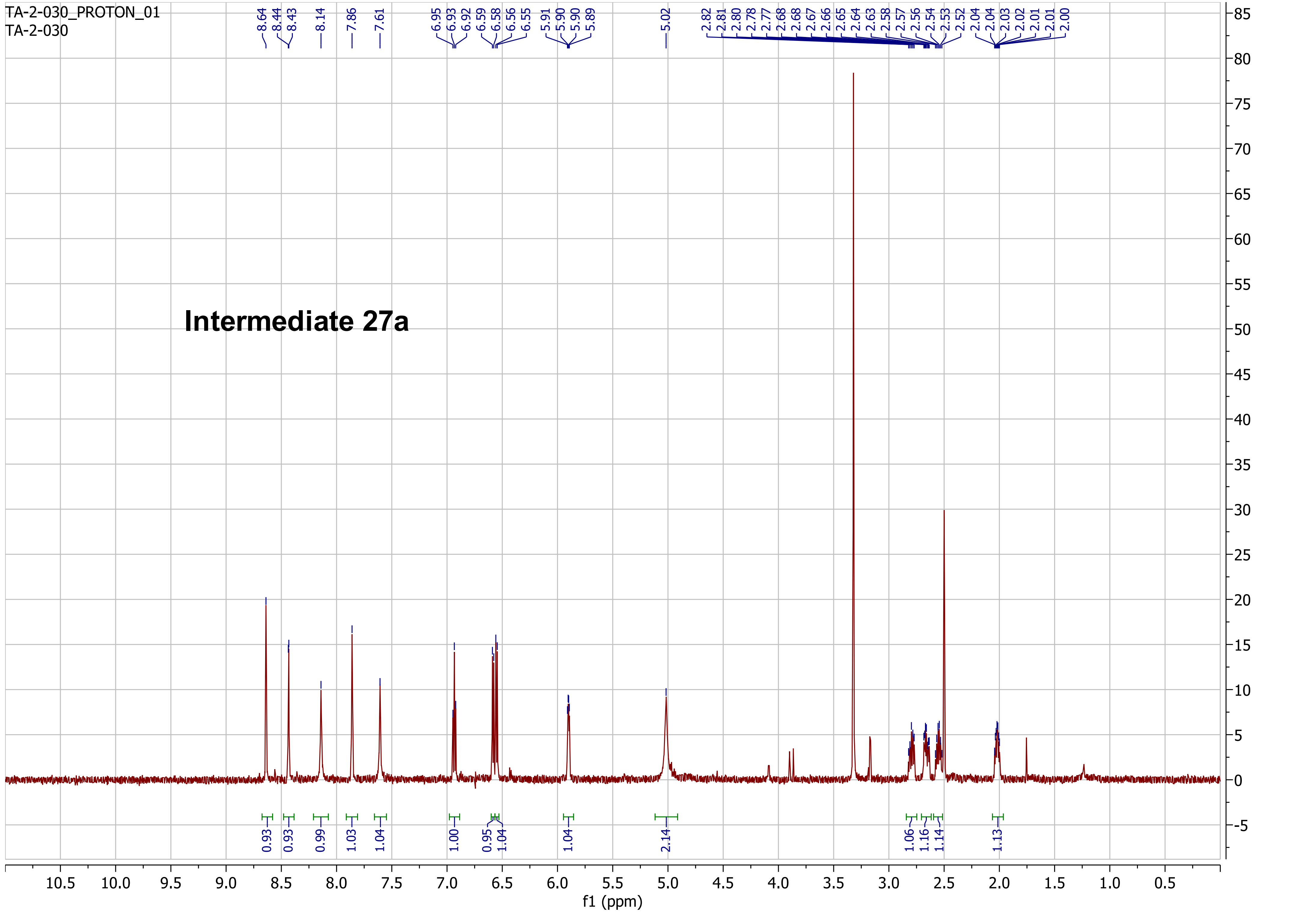
TA-2-030_PROTON_01
TA-2-030
Intermediate 27a
8.64
8.44
8.43
8.14
7.86
7.61
6.95
6.93
6.92
6.59
6.58
6.56
6.55
5.91
5.90
5.90
5.89
5.02
2.82
2.81
2.80
2.78
2.77
2.68
2.68
2.67
2.66
2.65
2.64
2.63
2.58
2.57
2.56
2.54
2.53
2.52
2.04
2.04
2.03
2.02
2.01
2.01
2.00
0.93
0.93
0.99
1.03
1.04
1.00
0.95
1.04
1.04
2.14
1.06
1.16
1.14
1.13
f1 (ppm)

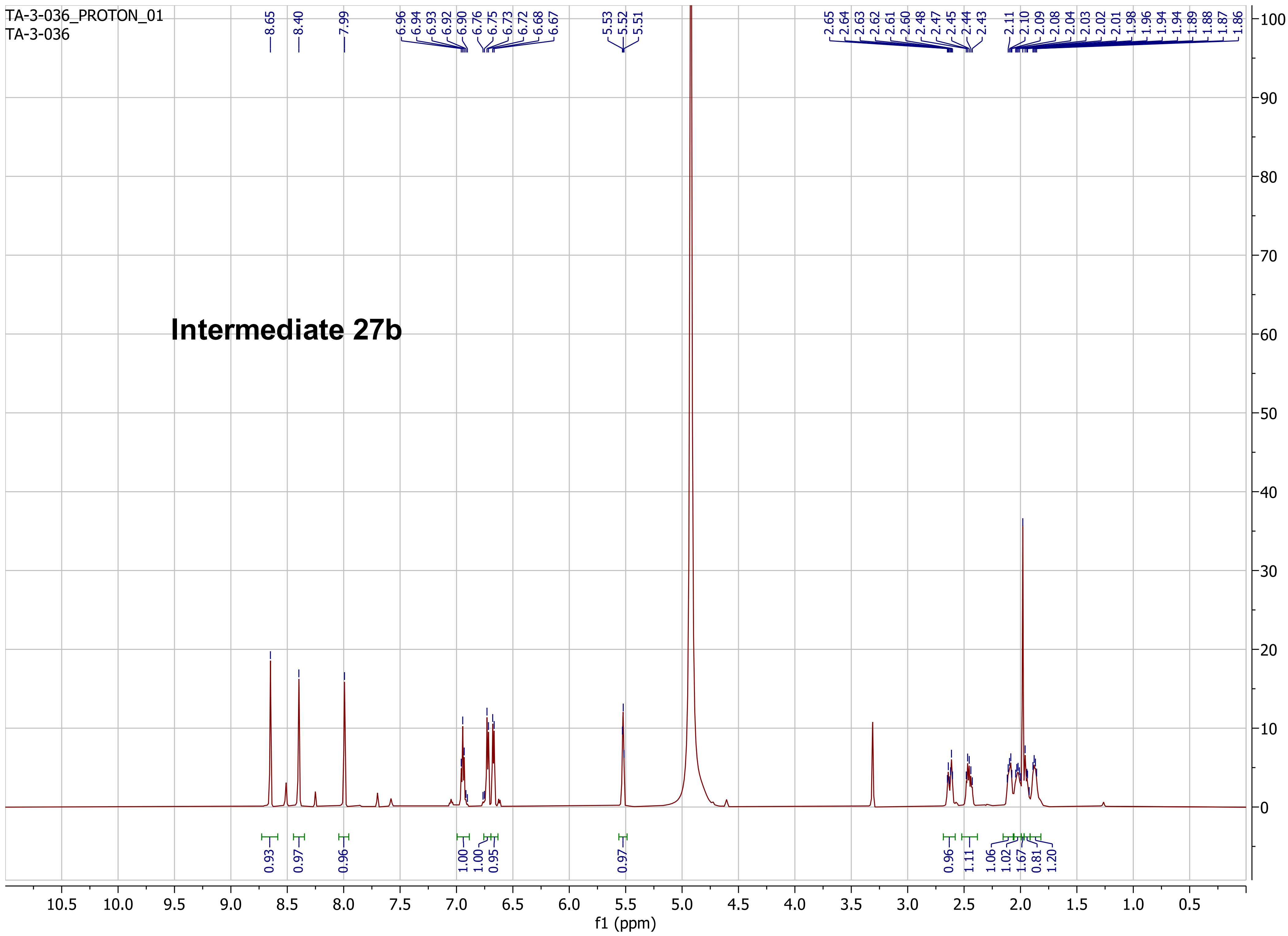
TA-3-036_PROTON_01
TA-3-036
Intermediate 27b
8.65
8.40
7.99
6.96
6.94
6.93
6.92
6.90
6.76
6.75
6.73
6.72
6.68
6.67
5.53
5.52
5.51
2.65
2.64
2.63
2.62
2.61
2.60
2.48
2.47
2.45
2.44
2.43
2.11
2.10
2.09
2.08
2.04
2.03
2.02
2.01
1.98
1.96
1.94
1.94
1.89
1.88
1.87
1.86
0.93
0.97
0.96
1.00
1.00
0.95
0.97
0.96
1.11
1.06
1.02
1.67
0.81
1.20
f1 (ppm)

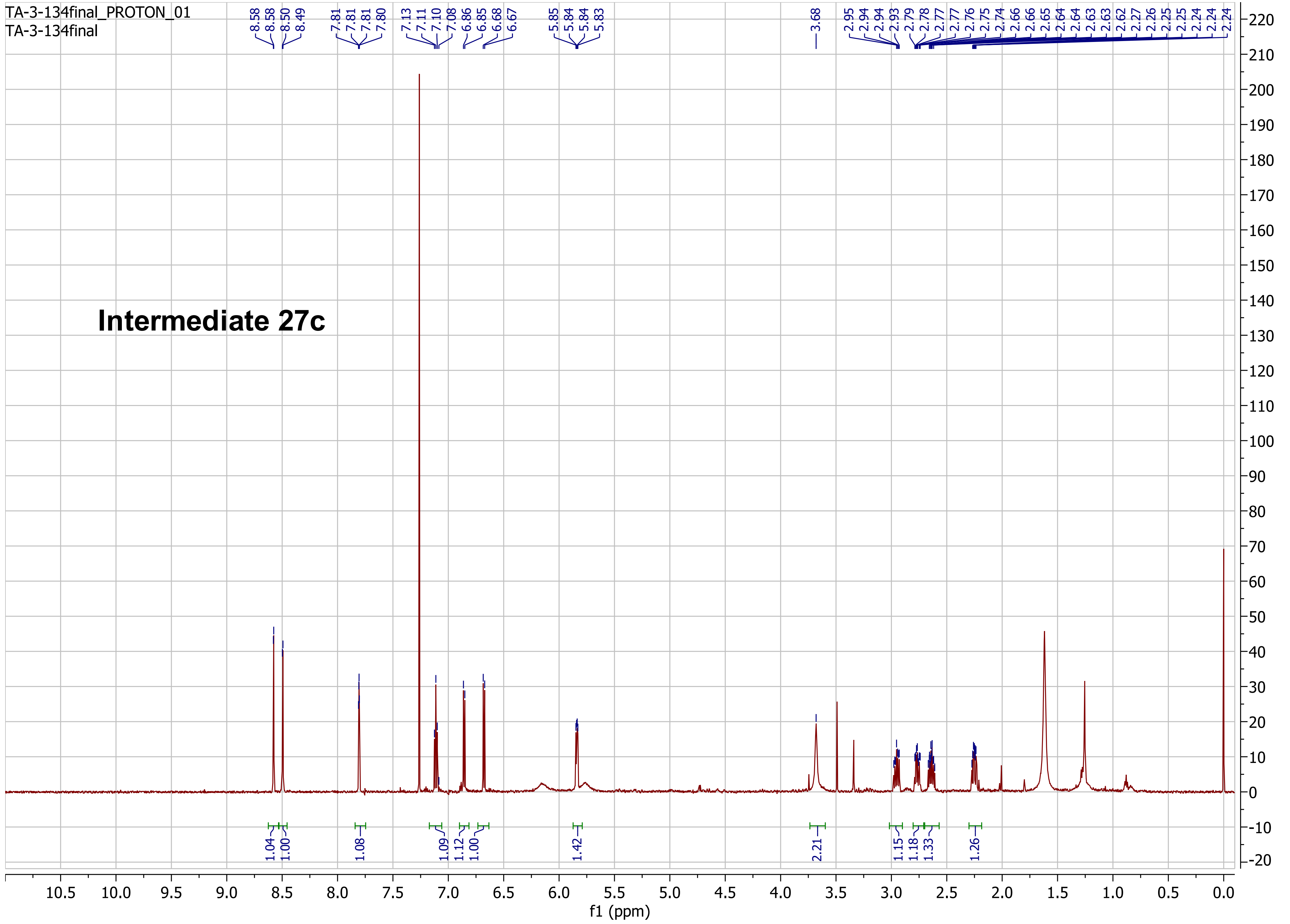

TA-3-134final_PROTON_01
TA-3-134final
Intermediate 27c
8.58 8.58 8.50 8.49 7.81 7.81 7.81 7.80 7.13 7.11 7.10 7.08 6.86 6.85 6.68 6.67 5.85 5.84 5.84 5.83 3.68 2.95 2.94 2.94 2.93 2.79 2.78 2.77 2.77 2.76 2.75 2.74 2.66 2.66 2.65 2.64 2.64 2.63 2.63 2.62 2.27 2.26 2.25 2.25 2.24 2.24 2.24
1.04 1.00 1.08 1.09 1.12 1.00 1.42 2.21 1.15 1.18 1.33 1.26
f1 (ppm)

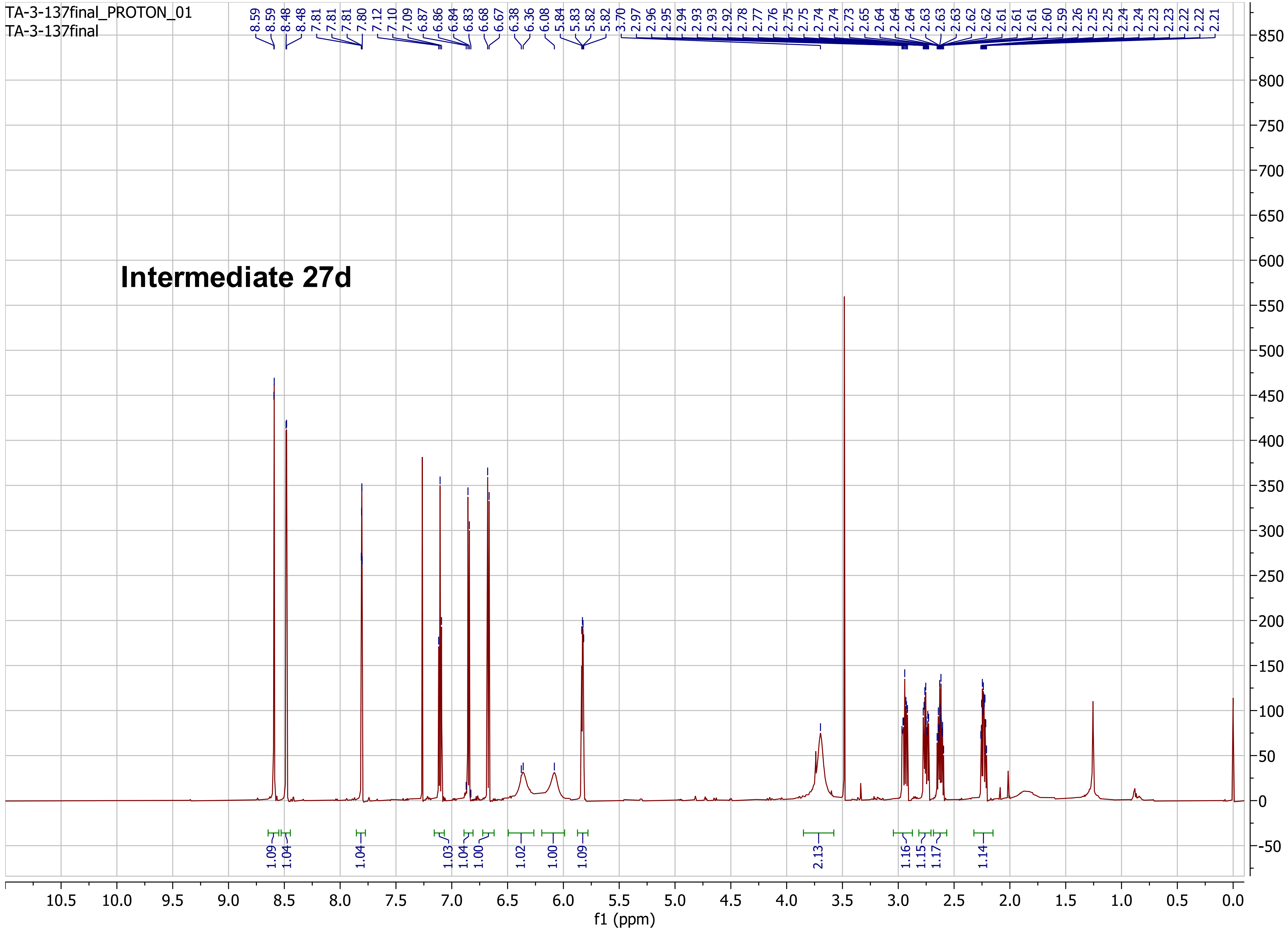
TA-3-137final_PROTON_01
TA-3-137final
8.59
8.59
8.48
8.48
7.81
7.81
7.81
7.80
7.12
7.10
7.09
6.87
6.86
6.84
6.83
6.68
6.67
6.38
6.36
6.08
5.84
5.83
5.82
5.82
3.70
2.97
2.96
2.95
2.94
2.93
2.93
2.92
2.78
2.77
2.76
2.75
2.75
2.74
2.74
2.73
2.65
2.64
2.64
2.64
2.63
2.63
2.63
2.62
2.62
2.61
2.61
2.61
2.60
2.59
2.26
2.25
2.25
2.24
2.24
2.23
2.23
2.22
2.22
2.21
Intermediate 27d
1.09
1.04
1.04
1.03
1.04
1.00
1.02
1.00
1.09
2.13
1.16
1.15
1.17
1.14
f1 (ppm)

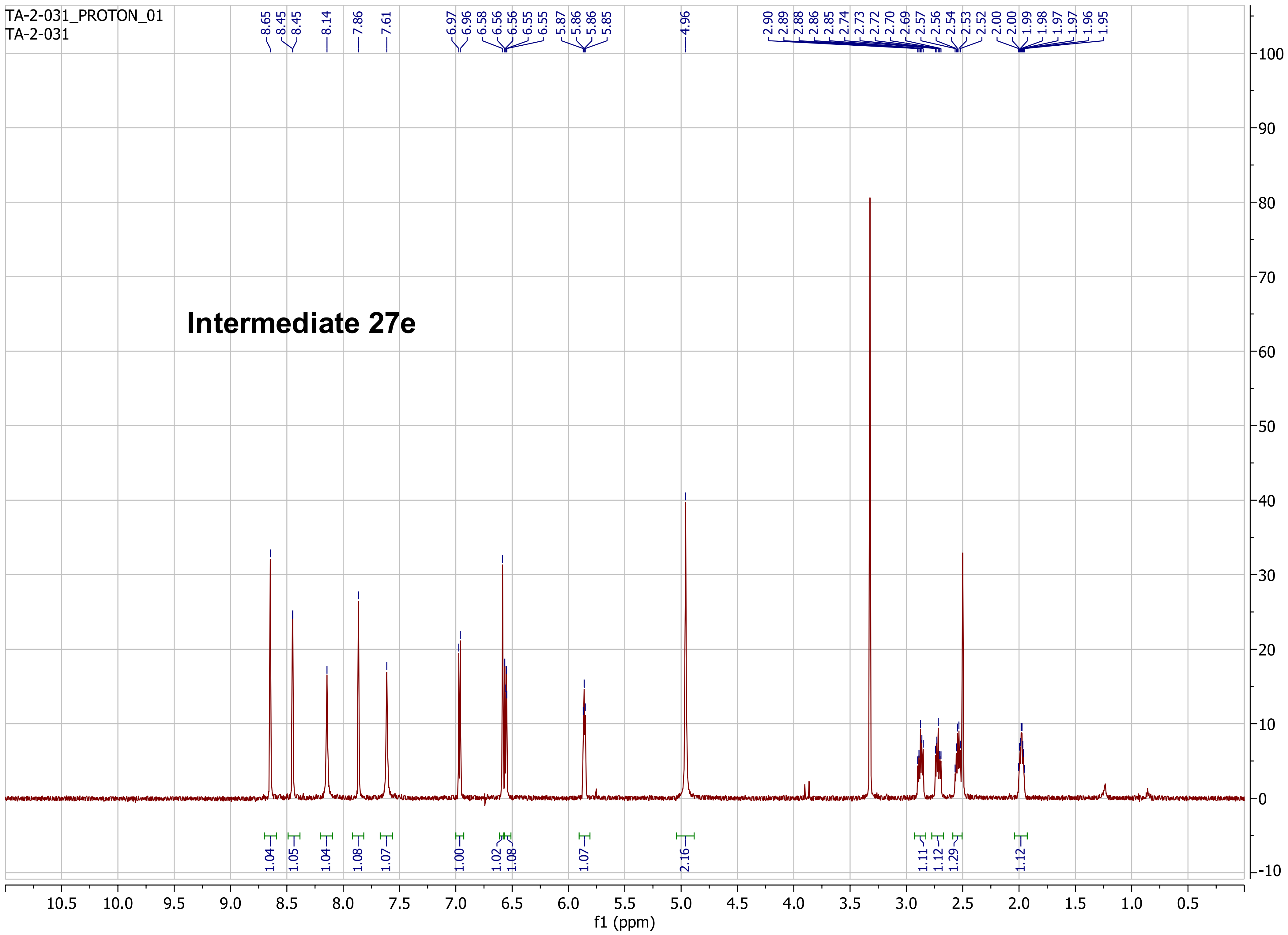

TA-2-031_PROTON_01
TA-2-031
Intermediate 27e
8.65
8.45
8.45
8.14
7.86
7.61
6.97
6.96
6.58
6.56
6.56
6.55
6.55
5.87
5.86
5.86
5.85
4.96
2.90
2.89
2.88
2.86
2.85
2.74
2.73
2.72
2.70
2.69
2.57
2.56
2.54
2.53
2.52
2.00
2.00
1.99
1.98
1.97
1.97
1.96
1.95
1.04
1.05
1.04
1.08
1.07
1.00
1.02
1.08
1.07
2.16
1.11
1.12
1.29
1.12
f1 (ppm)

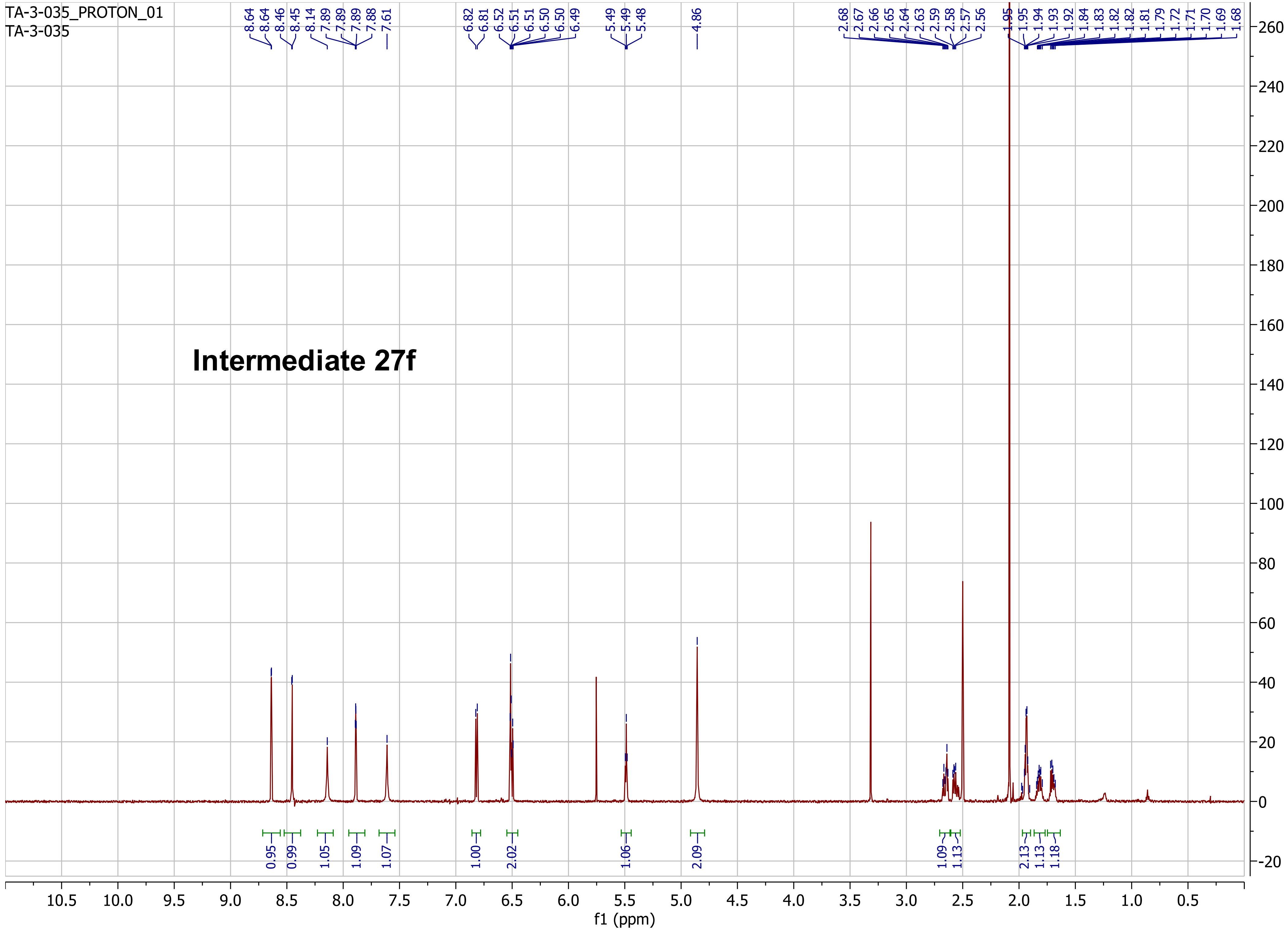

TA-3-035_PROTON_01
TA-3-035
8.64
8.64
8.46
8.45
8.14
7.89
7.89
7.89
7.88
7.61
6.82
6.81
6.52
6.51
6.51
6.50
6.50
6.49
5.49
5.49
5.48
4.86
2.68
2.67
2.66
2.65
2.64
2.63
2.59
2.58
2.57
2.56
1.95
1.95
1.94
1.93
1.92
1.84
1.83
1.82
1.82
1.81
1.79
1.72
1.71
1.70
1.69
1.68
Intermediate 27f
0.95
0.99
1.05
1.09
1.07
1.00
2.02
1.06
2.09
1.09
1.13
2.13
1.13
1.18
f1 (ppm)

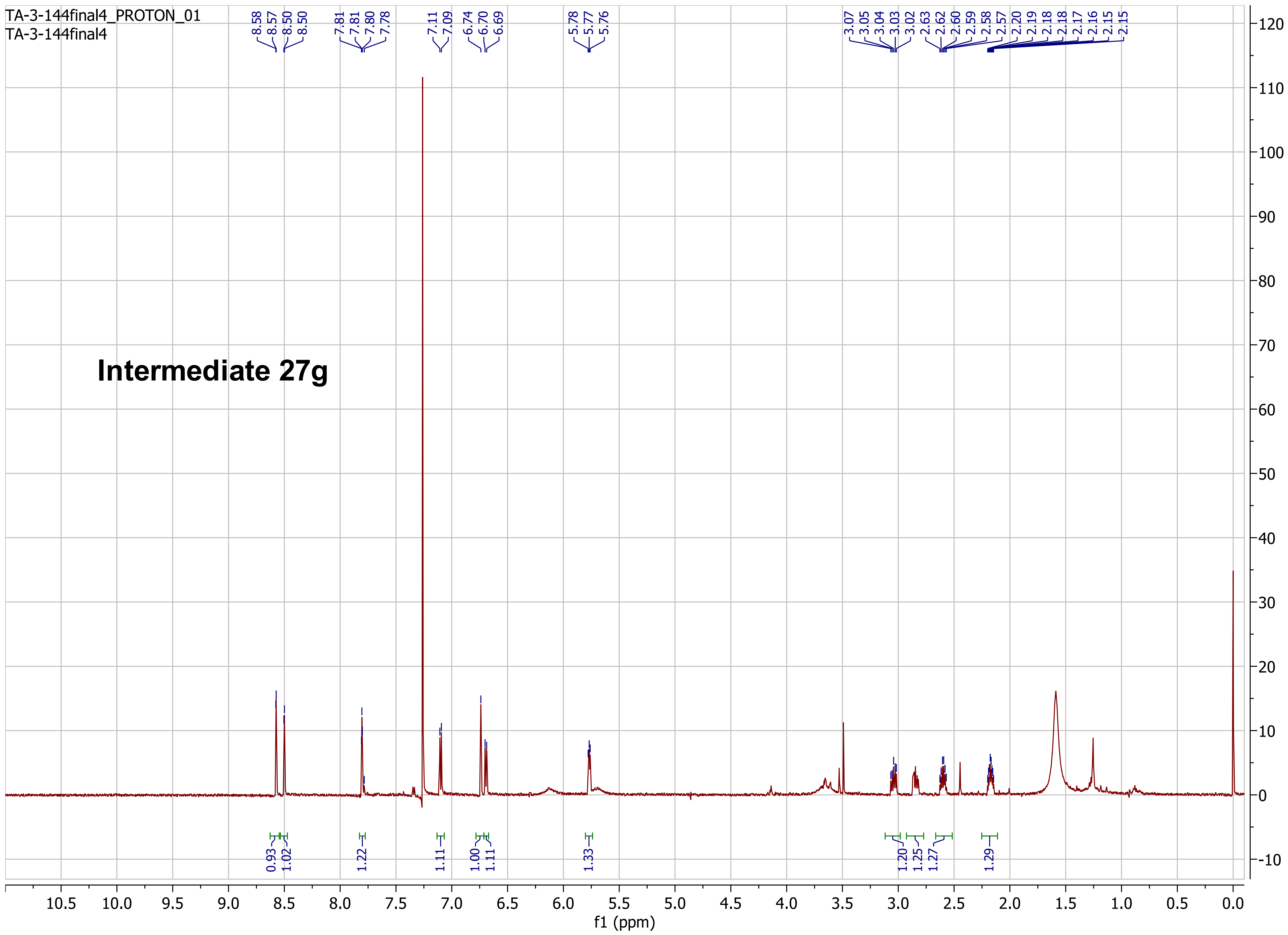

TA-3-144final4_PROTON_01
TA-3-144final4
8.58
8.57
8.50
8.50
7.81
7.81
7.80
7.78
7.11
7.09
6.74
6.70
6.69
5.78
5.77
5.76
3.07
3.05
3.04
3.03
3.02
2.63
2.62
2.60
2.59
2.58
2.57
2.20
2.19
2.18
2.18
2.17
2.16
2.15
2.15
Intermediate 27g
0.93
1.02
1.22
1.11
1.00
1.11
1.33
1.20
1.25
1.27
1.29
f1 (ppm)

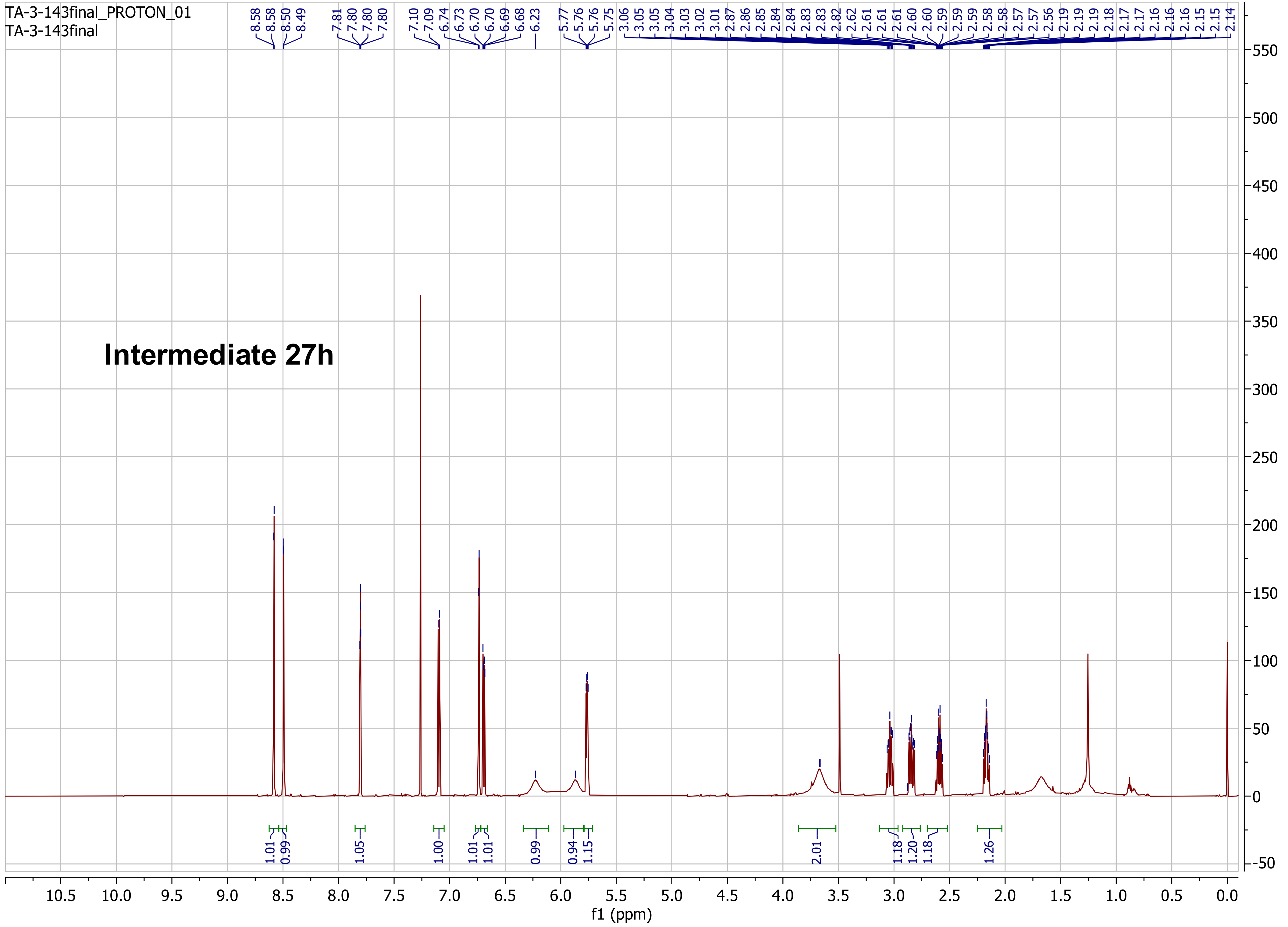
TA-3-143final_PROTON_01
TA-3-143final
8.58 8.58 8.50 8.49 7.81 7.80 7.80 7.80 7.10 7.09 6.74 6.73 6.70 6.70 6.69 6.68 6.23 5.77 5.76 5.76 5.75 3.06 3.05 3.05 3.04 3.03 3.02 3.01 2.87 2.86 2.85 2.84 2.84 2.83 2.83 2.82 2.62 2.61 2.61 2.61 2.60 2.60 2.59 2.59 2.59 2.58 2.58 2.57 2.57 2.56 2.19 2.19 2.19 2.18 2.17 2.17 2.16 2.16 2.16 2.15 2.15 2.14
Intermediate 27h
1.01 0.99 1.05 1.00 1.01 1.01 0.99 0.94 1.15 2.01 1.18 1.20 1.18 1.26
f1 (ppm)

Data File C:\Chem32\1\Data\Sir2 Purity test MeCN_rui 2016-04-15 16-44-07\OnlineEdited16.D
Sample Name: LC0420

Compound 15a

===

Acq. Operator : SYSTEM Seq. Line : 13
Acq. Instrument : CDD Location : P1-B-04
Injection Date : 4/15/2016 8:20:30 PM Inj : 1
Inj Volume : 10.000 µl
Method : C:\Chem32\1\Data\Sir2 Purity test MeCN_rui 2016-04-15 16-44-07\Sir2 purity_30-100%MeCN.M (Sequence Method)
Last changed : 4/15/2016 4:44:07 PM by SYSTEM
Method Info : test

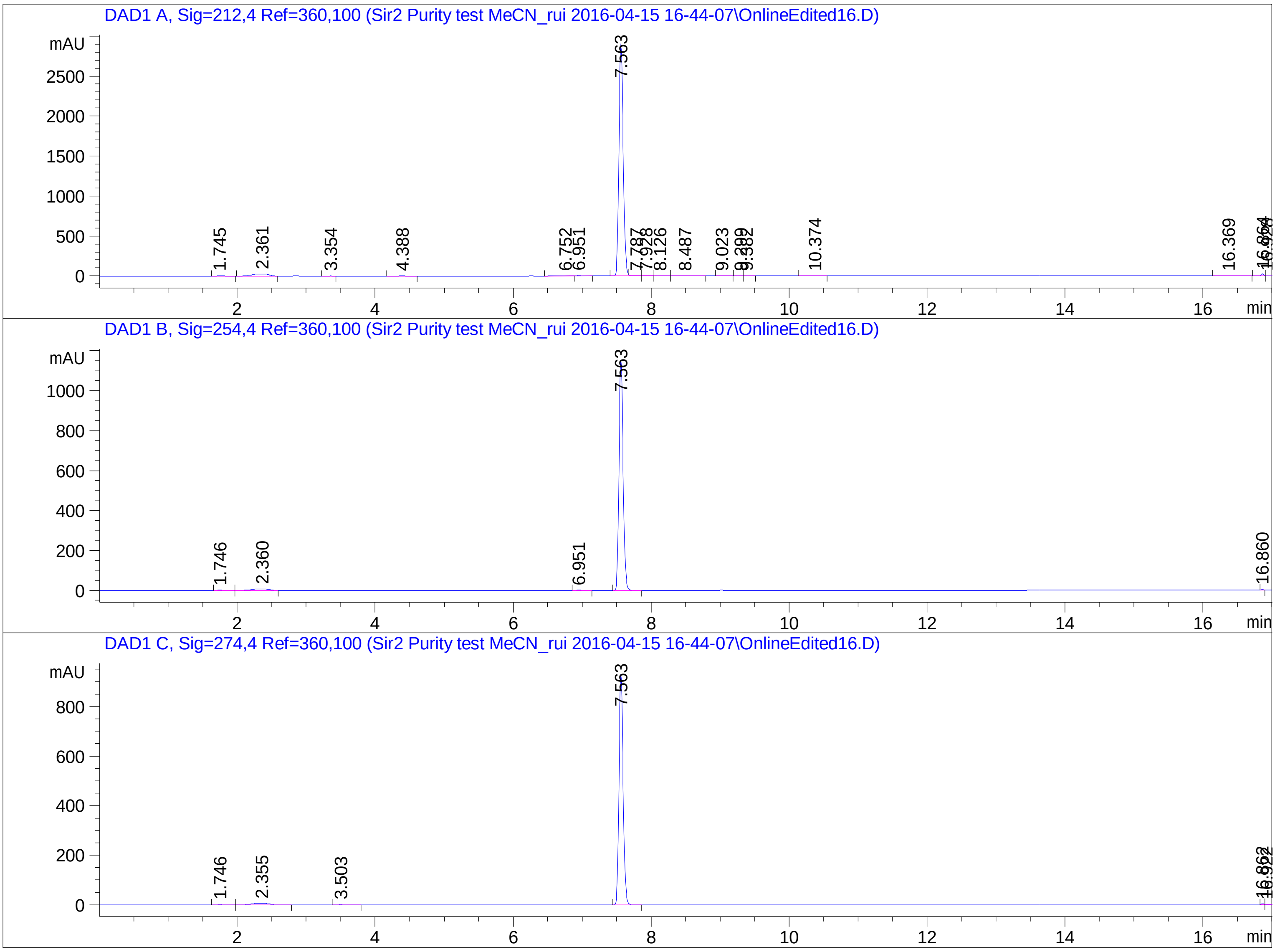

===

Area Percent Report

===

Sorted By : Signal
Multiplier : 1.0000
Dilution : 1.0000
Use Multiplier & Dilution Factor with ISTDs

Signal 1: DAD1 A, Sig=212,4 Ref=360,100

Peak #	RetTime [min]	Type	Width [min]	Area [mAU*s]	Height [mAU]	Area %
1	1.745	BB	0.0799	34.25770	6.06529	0.2593
2	2.361	BB	0.2374	445.55951	27.73595	3.3722
3	3.354	BB	0.0847	12.30412	2.09051	0.0931
4	4.388	BB	0.0752	20.30573	4.14401	0.1537
5	6.752	BV E	0.1112	13.20377	1.61742	0.0999
6	6.951	VB R	0.0708	40.66706	8.65966	0.3078
7	7.563	BV R	0.0685	1.24793e4	2881.26001	94.4478
8	7.787	VV E	0.0859	9.98386	1.66689	0.0756
9	7.928	VB E	0.0674	12.17470	2.76358	0.0921
10	8.126	BB	0.0794	5.90134	1.08546	0.0447
11	8.487	BB	0.1104	8.71485	1.07668	0.0660
12	9.023	BB	0.0692	13.54113	3.08426	0.1025
13	9.299	BV	0.0663	7.10442	1.64785	0.0538
14	9.382	VV	0.0899	8.37466	1.43871	0.0634
15	10.374	BB	0.0781	7.30977	1.41798	0.0553
16	16.369	BB	0.1947	24.75417	1.86637	0.1873
17	16.864	BB	0.0422	62.28437	24.08801	0.4714
18	16.928	BBA	0.0422	7.17131	2.59580	0.0543
Totals :				1.32129e4	2974.30443	

Signal 2: DAD1 B, Sig=254,4 Ref=360,100

Peak #	RetTime [min]	Type	Width [min]	Area [mAU*s]	Height [mAU]	Area %
1	1.746	BB	0.0770	9.21249	1.70479	0.1861
2	2.360	BB	0.2376	130.64775	8.12622	2.6395
3	6.951	VB	0.0723	8.34252	1.72982	0.1685
4	7.563	BB	0.0645	4792.79492	1154.02332	96.8300
5	16.860	BB	0.0373	8.70266	4.01958	0.1758
Totals :				4949.70034	1169.60372	

Signal 3: DAD1 C, Sig=274,4 Ref=360,100

Peak #	RetTime [min]	Type	Width [min]	Area [mAU*s]	Height [mAU]	Area %
1	1.746	BB	0.0770	7.78699	1.44180	0.1946
2	2.355	BB	0.2390	111.03525	6.92636	2.7750
3	3.503	BB	0.1057	7.36647	1.02670	0.1841
4	7.563	BB	0.0644	3860.36646	930.11615	96.4776
5	16.862	BV	0.0397	9.23295	3.89522	0.2307
6	16.922	VBA	0.0482	5.52230	1.68586	0.1380
Totals :				4001.31041	945.09209	

===

*** End of Report ***

Compound 15b

===

Acq. Operator : SYSTEM Seq. Line : 17
Acq. Instrument : CDD Location : P1-B-08
Injection Date : 4/15/2016 9:32:16 PM Inj : 1
Inj Volume : 10.000 µl
Method : C:\Chem32\1\Data\Sir2 Purity test MeCN_rui 2016-04-15 16-44-07\Sir2 purity_30-100%MeCN.M (Sequence Method)
Last changed : 4/15/2016 4:44:07 PM by SYSTEM
Method Info : test

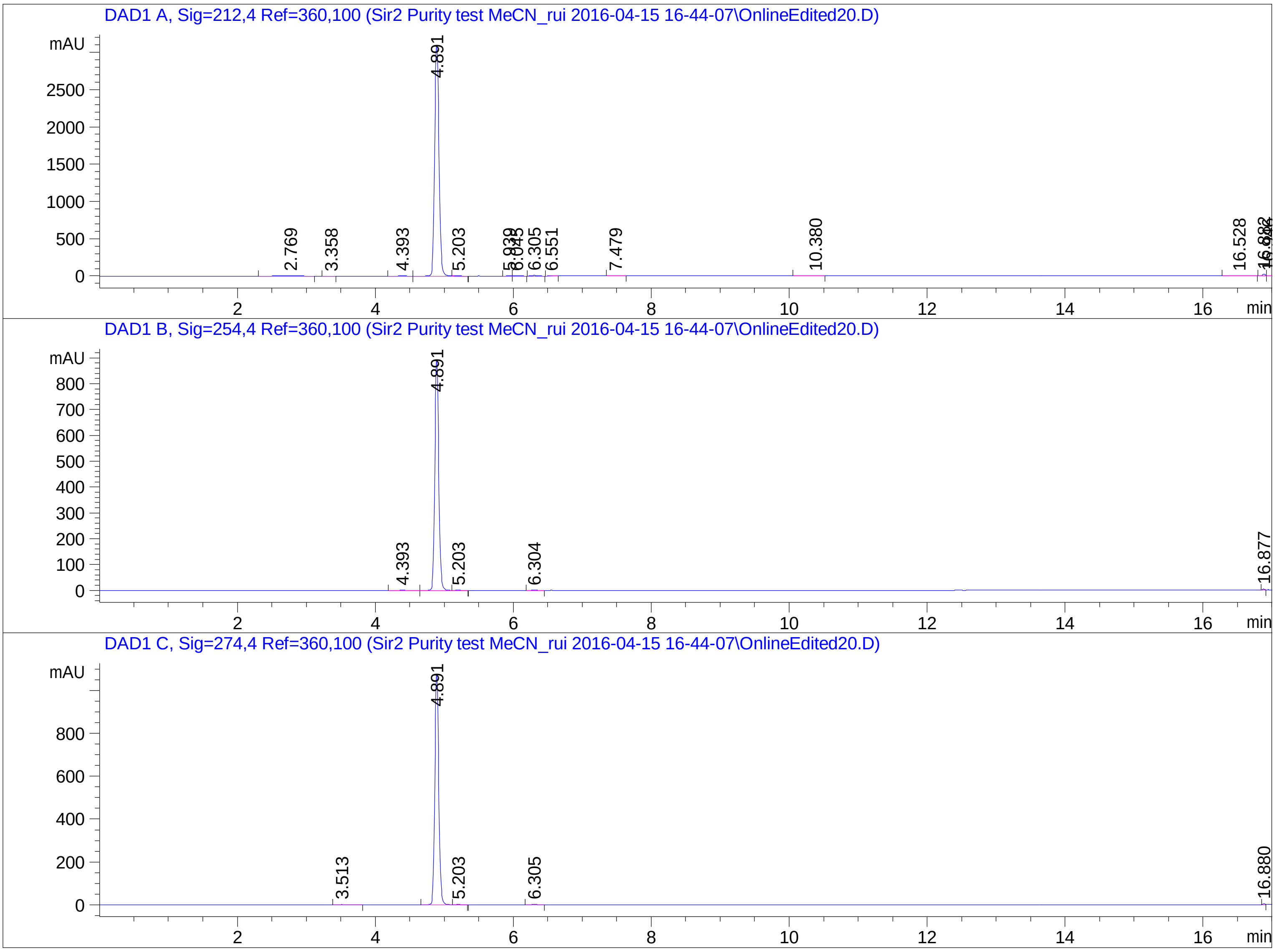

===

Area Percent Report

===

Sorted By : Signal
Multiplier : 1.0000
Dilution : 1.0000
Use Multiplier & Dilution Factor with ISTDs

Signal 1: DAD1 A, Sig=212,4 Ref=360,100

Peak #	RetTime [min]	Type	Width [min]	Area [mAU*s]	Height [mAU]	Area %
1	2.769	BB	0.3503	89.69124	3.32913	0.6379
2	3.358	BB	0.0821	11.45496	1.96372	0.0815
3	4.393	BB	0.0754	28.89342	5.86935	0.2055
4	4.891	BV R	0.0716	1.37323e4	3104.90112	97.6619
5	5.203	VB E	0.0610	24.93639	6.45880	0.1773
6	5.939	VV	0.0621	7.33439	1.85440	0.0522
7	6.045	VB	0.0686	9.38280	2.08290	0.0667
8	6.305	BB	0.0687	38.29269	8.47715	0.2723
9	6.551	BB	0.0627	16.82673	4.20080	0.1197
10	7.479	BB	0.0893	8.61263	1.37098	0.0613
11	10.380	BB	0.0762	7.04870	1.41374	0.0501
12	16.528	BB	0.2113	25.10757	1.81606	0.1786
13	16.882	BB	0.0404	54.55837	22.40466	0.3880
14	16.946	BBA	0.0392	6.62195	2.64574	0.0471
Totals :				1.40611e4	3168.78855	

Signal 2: DAD1 B, Sig=254,4 Ref=360,100

Peak #	RetTime [min]	Type	Width [min]	Area [mAU*s]	Height [mAU]	Area %
1	4.393	BB	0.0784	6.13708	1.18549	0.1708
2	4.891	BV R	0.0623	3563.33325	898.31262	99.1980
3	5.203	VB E	0.0611	6.52240	1.68542	0.1816
4	6.304	BB	0.0674	7.63521	1.73198	0.2126
5	16.877	BB	0.0370	8.51307	3.97494	0.2370
Totals :				3592.14102	906.89045	

Signal 3: DAD1 C, Sig=274,4 Ref=360,100

Peak #	RetTime [min]	Type	Width [min]	Area [mAU*s]	Height [mAU]	Area %
1	3.513	BB	0.1139	8.37626	1.06360	0.1936
2	4.891	BV R	0.0623	4293.36816	1082.89722	99.2457
3	5.203	VB E	0.0604	6.45765	1.69778	0.1493
4	6.305	BB	0.0702	9.12161	1.96493	0.2109
5	16.880	BV	0.0394	8.67743	3.68964	0.2006
Totals :				4326.00112	1091.31317	

==

*** End of Report ***

Data File C:\Chem32\1\Data\Sir2 Purity test MeCN_rui 2016-04-15 16-44-07\OnlineEdited22.D
Sample Name: LC0424

Compound 16a

===

Acq. Operator : SYSTEM Seq. Line : 19
Acq. Instrument : CDD Location : P1-C-01
Injection Date : 4/15/2016 10:08:08 PM Inj : 1
Inj Volume : 10.000 µl
Method : C:\Chem32\1\Data\Sir2 Purity test MeCN_rui 2016-04-15 16-44-07\Sir2 purity_30-100%MeCN.M (Sequence Method)
Last changed : 4/15/2016 4:44:07 PM by SYSTEM
Method Info : test

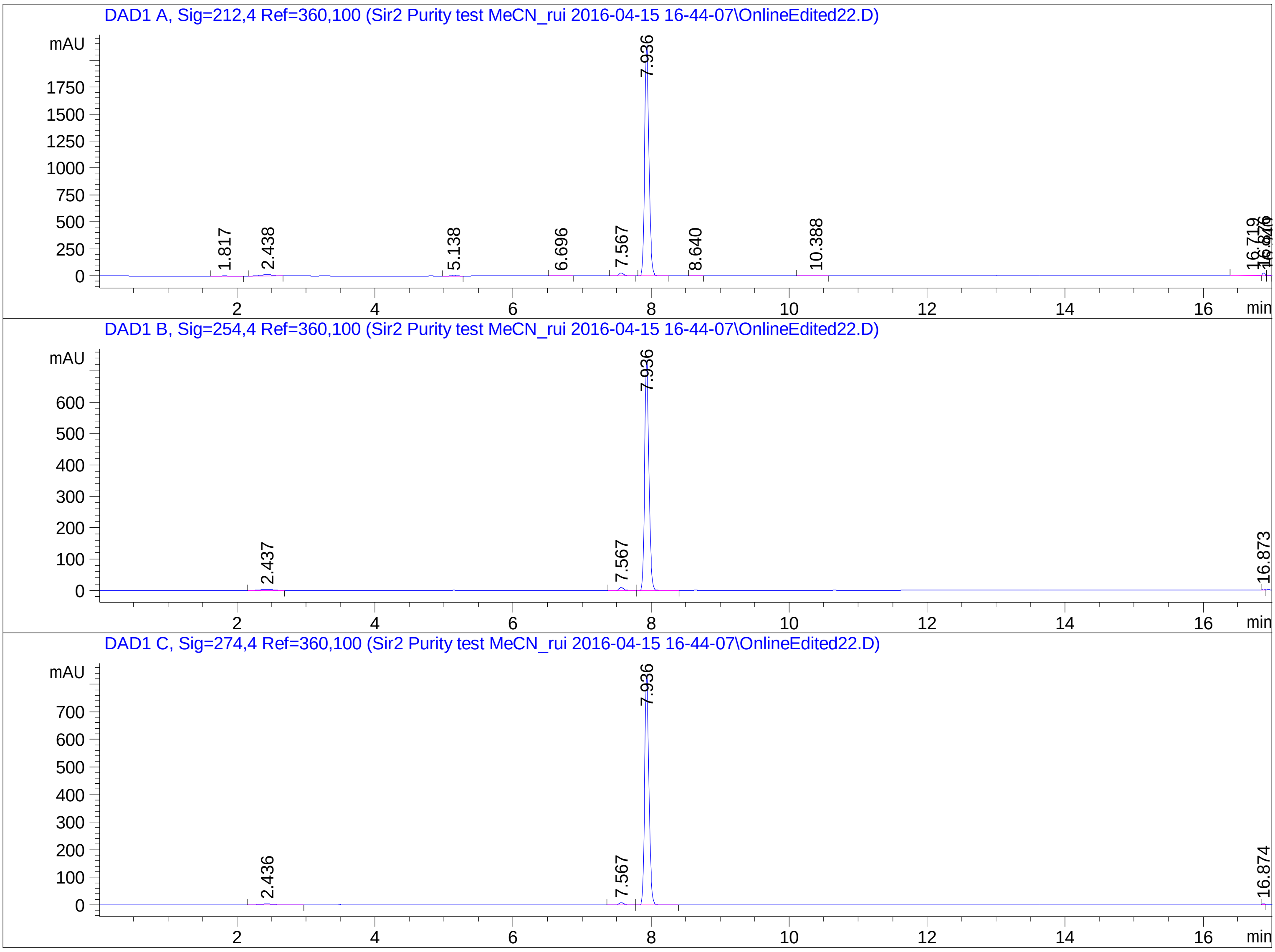

===

Area Percent Report

===

Sorted By : Signal
Multiplier : 1.0000
Dilution : 1.0000
Use Multiplier & Dilution Factor with ISTDs

Signal 1: DAD1 A, Sig=212,4 Ref=360,100

Peak #	RetTime [min]	Type	Width [min]	Area [mAU*s]	Height [mAU]	Area %
1	1.817	BB	0.1071	8.34913	1.06936	0.0866
2	2.438	BB	0.1786	156.39441	12.44976	1.6224
3	5.138	BB	0.0639	27.44308	6.69014	0.2847
4	6.696	BB	0.0944	8.74076	1.30026	0.0907
5	7.567	BB	0.0683	124.27000	27.69481	1.2892
6	7.936	BB	0.0661	9186.12988	2137.25757	95.2963
7	8.640	BB	0.0716	5.58220	1.21518	0.0579
8	10.388	BB	0.0799	5.98445	1.09215	0.0621
9	16.719	BV E	0.2247	50.04313	3.34234	0.5191
10	16.876	VB R	0.0405	60.47182	24.72046	0.6273
11	16.940	BBA	0.0380	6.13327	2.55244	0.0636

Totals : 9639.54214 2219.38447

Signal 2: DAD1 B, Sig=254,4 Ref=360,100

Peak #	RetTime [min]	Type	Width [min]	Area [mAU*s]	Height [mAU]	Area %
1	2.437	BB	0.1803	46.18706	3.63339	1.4268
2	7.567	BB	0.0679	43.50428	9.77919	1.3439
3	7.936	BB	0.0658	3138.62915	735.91669	96.9569
4	16.873	BB	0.0369	8.81914	4.13848	0.2724

Totals : 3237.13963 753.46774

Signal 3: DAD1 C, Sig=274,4 Ref=360,100

Peak #	RetTime [min]	Type	Width [min]	Area [mAU*s]	Height [mAU]	Area %
1	2.436	BB	0.1981	42.43031	3.13097	1.1595
2	7.567	BB	0.0684	35.63557	7.92784	0.9738
3	7.936	BB	0.0657	3572.46753	837.79974	97.6231
4	16.874	BV	0.0369	8.91385	3.86035	0.2436

Totals : 3659.44725 852.71890

==

*** End of Report ***

Data File C:\Chem32\1\Data\Sir2 Purity test MeCN_rui 2016-04-15 16-44-07\OnlineEdited26.D
Sample Name: LC0524

Compound 16b

===

Acq. Operator : SYSTEM Seq. Line : 23
Acq. Instrument : CDD Location : P1-C-05
Injection Date : 4/15/2016 11:19:51 PM Inj : 1
Inj Volume : 10.000 µl
Method : C:\Chem32\1\Data\Sir2 Purity test MeCN_rui 2016-04-15 16-44-07\Sir2 purity_30-100%MeCN.M (Sequence Method)
Last changed : 4/15/2016 4:44:07 PM by SYSTEM
Method Info : test

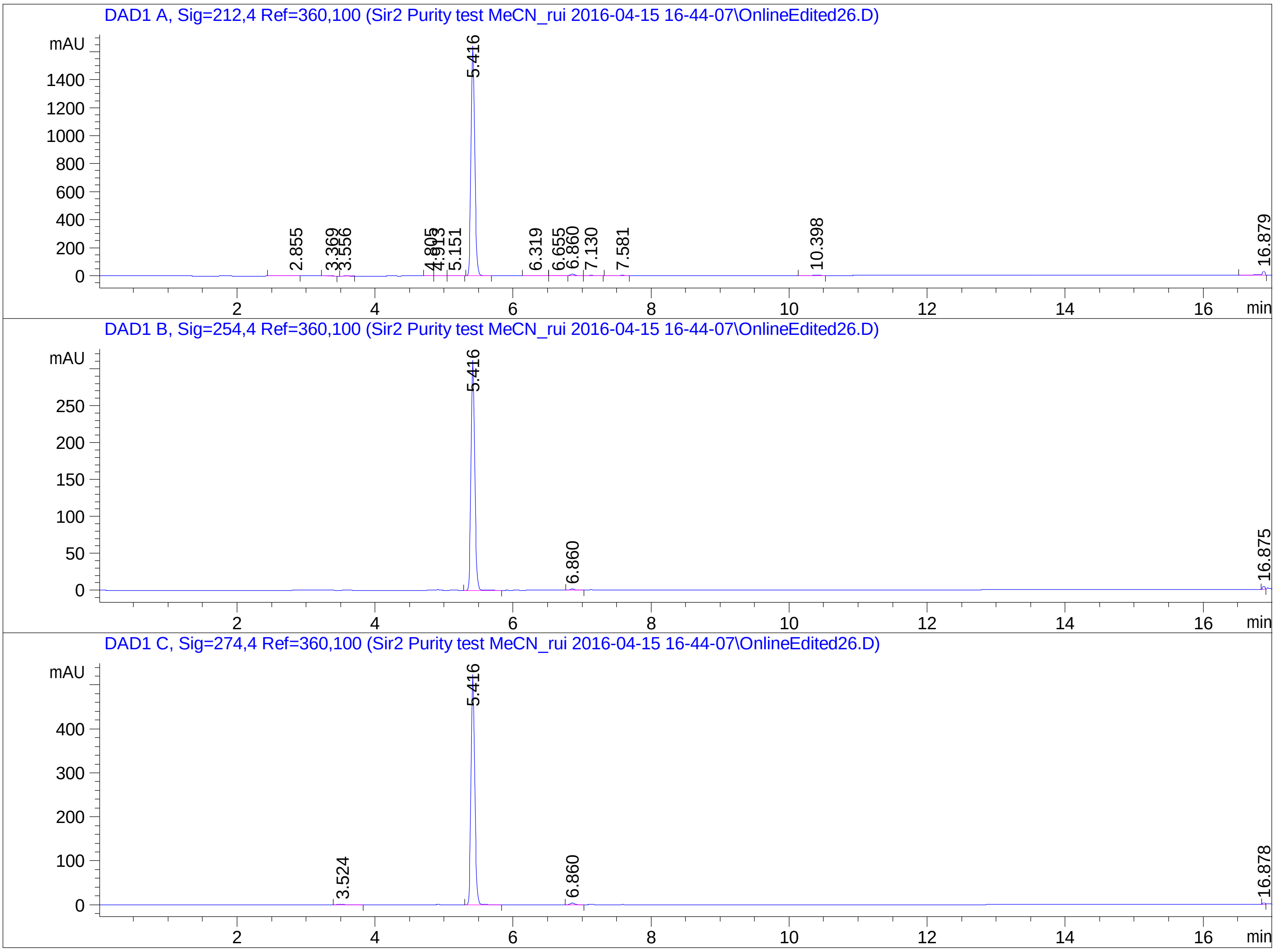

===

Area Percent Report

===

Sorted By : Signal
Multiplier : 1.0000
Dilution : 1.0000
Use Multiplier & Dilution Factor with ISTDs

Signal 1: DAD1 A, Sig=212,4 Ref=360,100

Peak #	RetTime [min]	Type	Width [min]	Area [mAU*s]	Height [mAU]	Area %
1	2.855	BV	0.1355	13.95104	1.36222	0.2133
2	3.369	BB	0.0876	13.23471	2.09846	0.2024
3	3.556	BB	0.0840	8.64549	1.44134	0.1322
4	4.805	BV	0.0621	5.26367	1.33143	0.0805
5	4.913	VB	0.0646	12.91914	2.97798	0.1976
6	5.151	BB	0.0692	12.56777	2.75616	0.1922
7	5.416	BB	0.0603	6271.46436	1651.32056	95.8999
8	6.319	BV R	0.0843	12.04557	2.05665	0.1842
9	6.655	BV E	0.0936	10.35916	1.59962	0.1584
10	6.860	VV R	0.0688	49.76157	11.00259	0.7609
11	7.130	VB	0.0747	11.58895	2.30435	0.1772
12	7.581	BB	0.0862	10.22757	1.80265	0.1564
13	10.398	BB	0.0749	6.70023	1.37479	0.1025
14	16.879	BB	0.0532	100.86498	28.54153	1.5424
Totals :				6539.59422	1711.97033	

Signal 2: DAD1 B, Sig=254,4 Ref=360,100

Peak #	RetTime [min]	Type	Width [min]	Area [mAU*s]	Height [mAU]	Area %
1	5.416	BB	0.0600	1183.16003	313.51331	98.5178
2	6.860	BB	0.0653	8.74658	2.07185	0.7283
3	16.875	BV	0.0380	9.05458	4.07071	0.7539
Totals :				1200.96119	319.65586	

Signal 3: DAD1 C, Sig=274,4 Ref=360,100

Peak #	RetTime [min]	Type	Width [min]	Area [mAU*s]	Height [mAU]	Area %
1	3.524	BB	0.1067	7.60098	1.02254	0.3763
2	5.416	BB	0.0600	1986.40198	526.51404	98.3331
3	6.860	BB	0.0651	17.04157	4.04902	0.8436
4	16.878	BV	0.0404	9.02906	3.70552	0.4470
Totals :				2020.07358	535.29111	

==

*** End of Report ***

Data File C:\Chem32\1\Data\Sir2 Purity test MeCN_rui 2016-04-15 16-44-07\OnlineEdited43.D
Sample Name: LC0480

Compound 17

===

Acq. Operator : SYSTEM Seq. Line : 25
Acq. Instrument : CDD Location : P1-C-07
Injection Date : 4/15/2016 11:55:45 PM Inj : 1
Inj Volume : 10.000 µl
Method : C:\Chem32\1\Data\Sir2 Purity test MeCN_rui 2016-04-15 16-44-07\Sir2 purity_30-100%MeCN.M (Sequence Method)
Last changed : 4/15/2016 4:44:07 PM by SYSTEM
Method Info : test

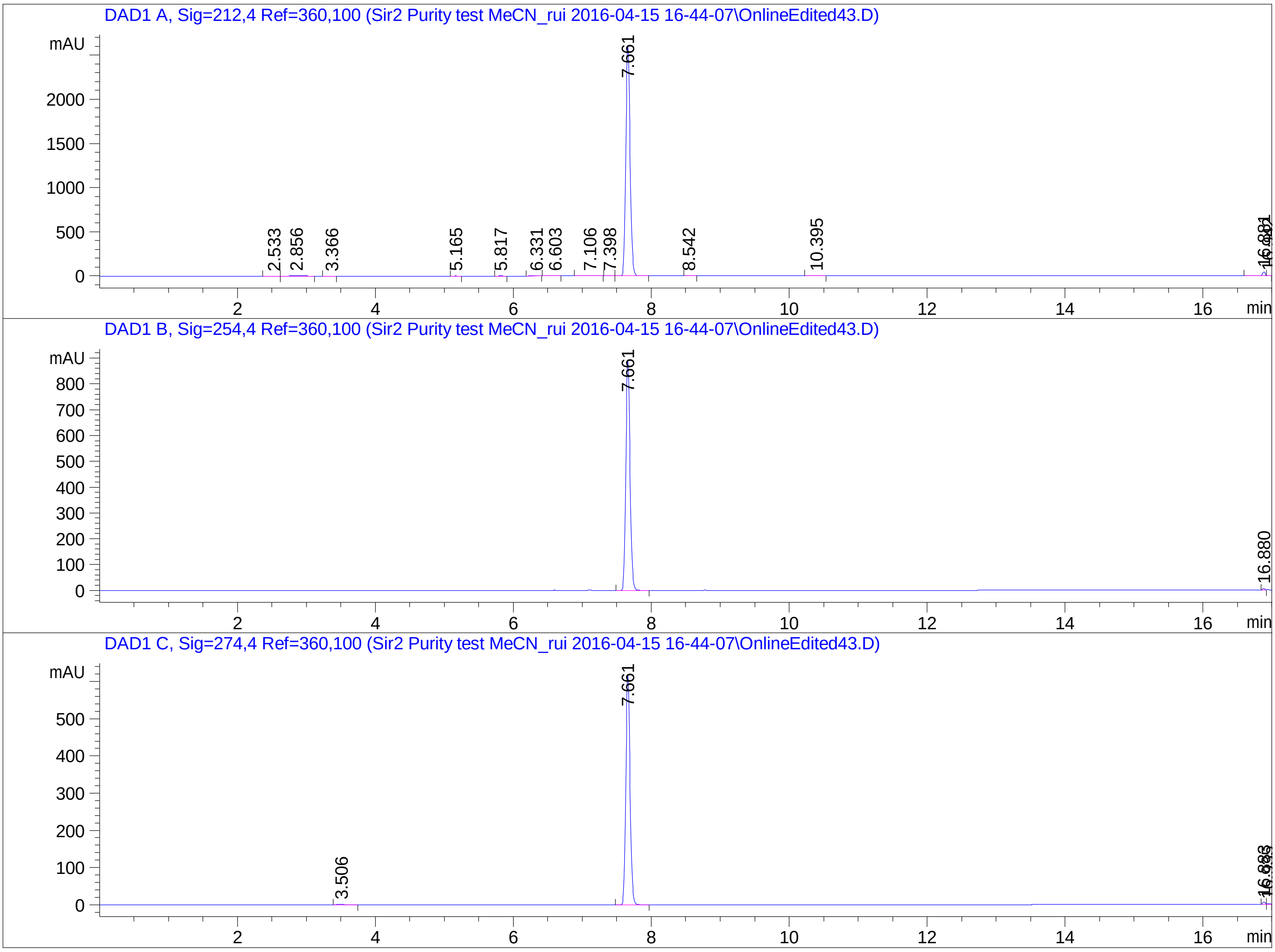

===

Area Percent Report

===

Sorted By : Signal
Multiplier : 1.0000
Dilution : 1.0000
Use Multiplier & Dilution Factor with ISTDs

Signal 1: DAD1 A, Sig=212,4 Ref=360,100

Peak #	RetTime [min]	Type	Width [min]	Area [mAU*s]	Height [mAU]	Area %
1	2.533	BB	0.1374	10.82505	1.17542	0.0944
2	2.856	BB	0.1674	31.36349	2.44848	0.2735
3	3.366	BB	0.0810	9.74245	1.69582	0.0849
4	5.165	BB	0.0605	5.25805	1.37989	0.0458
5	5.817	BB	0.0614	6.62249	1.70313	0.0577
6	6.331	BB	0.0750	15.37029	2.94093	0.1340
7	6.603	BB	0.0735	5.56755	1.17103	0.0485
8	7.106	BB	0.1549	13.94558	1.36786	0.1216
9	7.398	BV	0.0664	5.39589	1.24896	0.0470
10	7.661	VB	0.0683	1.12362e4	2604.29736	97.9673
11	8.542	VV	0.0703	5.03728	1.08239	0.0439
12	10.395	BB	0.0781	7.30391	1.41779	0.0637
13	16.881	BV R	0.0443	110.94479	40.02087	0.9673
14	16.942	VBAE	0.0394	5.76435	2.28741	0.0503
Totals :				1.14693e4	2664.23733	

Signal 2: DAD1 B, Sig=254,4 Ref=360,100

Peak #	RetTime [min]	Type	Width [min]	Area [mAU*s]	Height [mAU]	Area %
1	7.661	BB	0.0652	3763.15649	891.51782	99.6372
2	16.880	BV	0.0406	13.70383	5.58914	0.3628
Totals :				3776.86033	897.10696	

Signal 3: DAD1 C, Sig=274,4 Ref=360,100

Peak #	RetTime [min]	Type	Width [min]	Area [mAU*s]	Height [mAU]	Area %
1	3.506	BB	0.1018	8.71410	1.30743	0.3299
2	7.661	BB	0.0652	2611.00195	618.83527	98.8456
3	16.883	BV	0.0419	16.25802	6.34089	0.6155
4	16.935	VBA	0.0420	5.52143	1.89621	0.2090
Totals :				2641.49550	628.37979	

==

*** End of Report ***

Compound 18

==

Acq. Operator : SYSTEM Seq. Line : 29
Acq. Instrument : CDD Location : P1-D-02
Injection Date : 4/16/2016 1:07:30 AM Inj : 1
Inj Volume : 10.000 µl
Method : C:\Chem32\1\Data\Sir2 Purity test MeCN_rui 2016-04-15 16-44-07\Sir2 purity_30-100%MeCN.M (Sequence Method)
Last changed : 4/15/2016 4:44:07 PM by SYSTEM
Method Info : test

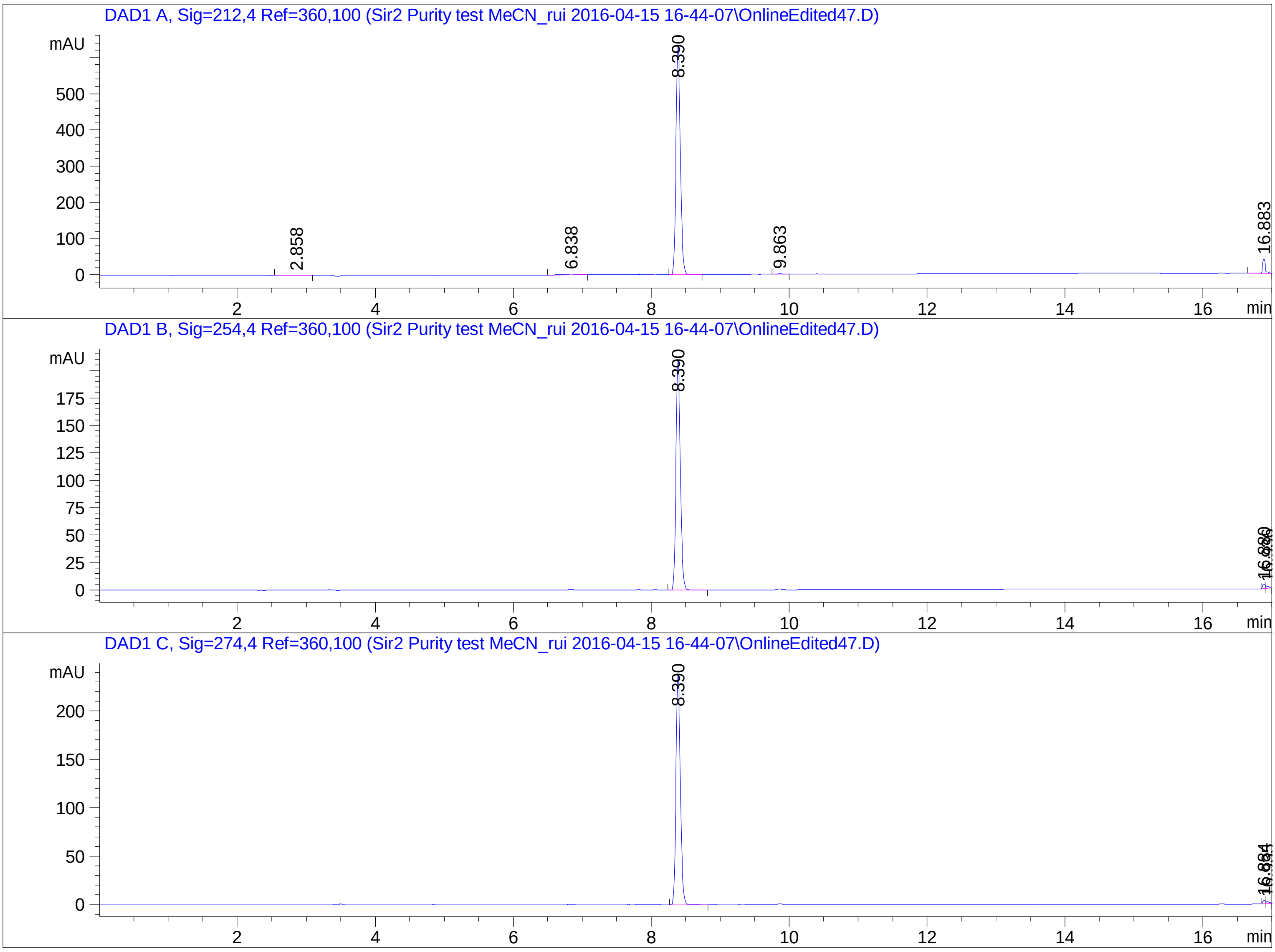

==

Area Percent Report

==

Sorted By : Signal
Multiplier : 1.0000
Dilution : 1.0000
Use Multiplier & Dilution Factor with ISTDs

Signal 1: DAD1 A, Sig=212,4 Ref=360,100

Peak #	RetTime [min]	Type	Width [min]	Area [mAU*s]	Height [mAU]	Area %
1	2.858	BB	0.1603	13.84749	1.13495	0.4714
2	6.838	BB	0.1042	16.57284	2.19201	0.5642
3	8.390	BB	0.0671	2779.38135	633.97571	94.6166
4	9.863	BB	0.0746	11.82981	2.43675	0.4027
5	16.883	BV R	0.0459	115.88936	39.82915	3.9451

Totals : 2937.52084 679.56856

Signal 2: DAD1 B, Sig=254,4 Ref=360,100

Peak #	RetTime [min]	Type	Width [min]	Area [mAU*s]	Height [mAU]	Area %
1	8.390	BB	0.0671	920.82233	210.15198	98.3385
2	16.880	BV	0.0415	10.31760	4.08629	1.1019
3	16.936	VBA	0.0412	5.24007	1.84270	0.5596

Totals : 936.38000 216.08097

Signal 3: DAD1 C, Sig=274,4 Ref=360,100

Peak #	RetTime [min]	Type	Width [min]	Area [mAU*s]	Height [mAU]	Area %
1	8.390	BB	0.0671	1044.88550	238.48172	98.6094
2	16.884	BV	0.0426	9.10065	3.47359	0.8589
3	16.935	VBA	0.0418	5.63447	1.94589	0.5317

Totals : 1059.62063 243.90120

==
*** End of Report ***

Compound 19a

===

Acq. Operator	: SYSTEM	Seq. Line :	33
Acq. Instrument	: CDD	Location :	P1-D-06
Injection Date	: 4/16/2016 2:19:14 AM	Inj :	1
		Inj Volume :	10.000 µl

Method : C:\Chem32\1\Data\Sir2 Purity test MeCN_rui 2016-04-15 16-44-07\Sir2 purity_30-100%MeCN.M (Sequence Method)
Last changed : 4/15/2016 4:44:07 PM by SYSTEM
Method Info : test

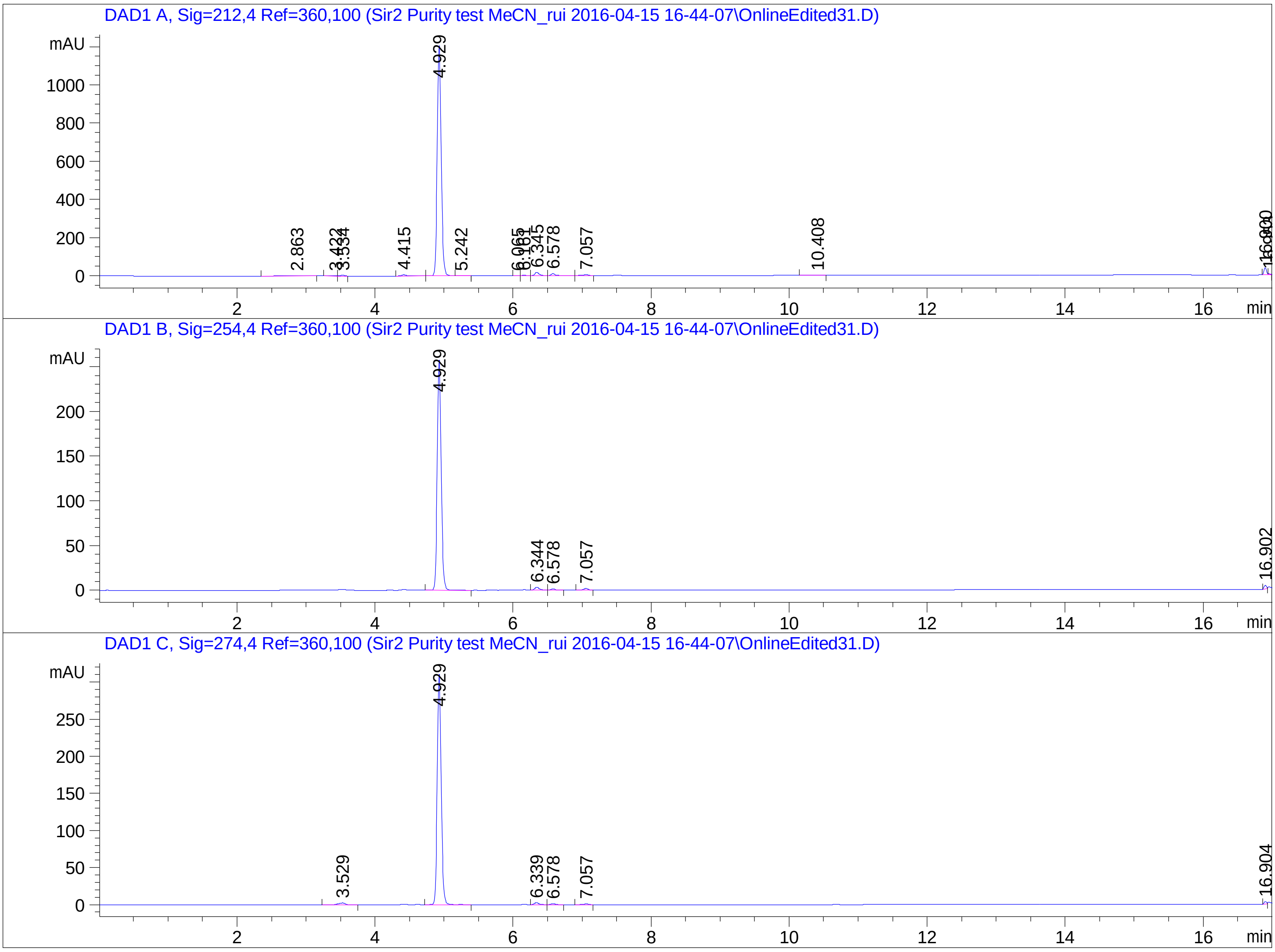

===

Area Percent Report

===

Sorted By	:	Signal
Multiplier	:	1.0000
Dilution	:	1.0000

Use Multiplier & Dilution Factor with ISTDs

Signal 1: DAD1 A, Sig=212,4 Ref=360,100

Peak #	RetTime [min]	Type	Width [min]	Area [mAU*s]	Height [mAU]	Area %
1	2.863	BB	0.3242	53.02539	2.07443	1.0007
2	3.422	BV	0.0840	7.12159	1.18677	0.1344
3	3.534	VB	0.0712	19.54276	4.12819	0.3688
4	4.415	BV R	0.0859	38.51936	6.42624	0.7269
5	4.929	VV R	0.0629	4885.66162	1215.51758	92.2014
6	5.242	VB E	0.0693	6.95860	1.52308	0.1313
7	6.065	VV	0.0575	5.04428	1.35359	0.0952
8	6.161	VB	0.0643	10.59871	2.55853	0.2000
9	6.345	BB	0.0698	78.24517	16.98199	1.4766
10	6.578	BV R	0.0737	50.41822	10.19941	0.9515
11	7.057	VB	0.0795	29.23104	5.37130	0.5516
12	10.408	BB	0.0746	6.84824	1.41264	0.1292
13	16.900	BV R	0.0430	102.31124	38.50151	1.9308
14	16.953	VBAE	0.0351	5.37804	2.50199	0.1015

Totals : 5298.90427 1309.73725

Signal 2: DAD1 B, Sig=254,4 Ref=360,100

Peak #	RetTime [min]	Type	Width [min]	Area [mAU*s]	Height [mAU]	Area %
1	4.929	BV R	0.0627	1038.17200	259.26132	96.2048
2	6.344	BV	0.0714	15.70346	3.30841	1.4552
3	6.578	VB	0.0658	6.62663	1.55214	0.6141
4	7.057	BB	0.0717	8.63590	1.80742	0.8003
5	16.902	BV	0.0420	9.98896	3.88999	0.9257

Totals : 1079.12694 269.81928

Signal 3: DAD1 C, Sig=274,4 Ref=360,100

Peak #	RetTime [min]	Type	Width [min]	Area [mAU*s]	Height [mAU]	Area %
1	3.529	BB	0.1041	16.93034	2.46611	1.3016
2	4.929	BV R	0.0627	1251.75537	312.48953	96.2378
3	6.339	BV	0.0706	12.35552	2.73845	0.9499
4	6.578	VB	0.0647	6.27346	1.50189	0.4823
5	7.057	BB	0.0736	5.58472	1.13167	0.4294
6	16.904	BV	0.0426	7.79049	2.97347	0.5990

Totals : 1300.68990 323.30113

===

*** End of Report ***

Compound 19b

===

Acq. Operator : SYSTEM   Seq. Line : 34
Acq. Instrument : CDD   Location : P1-D-07
Injection Date : 4/16/2016 2:37:12 AM   Inj : 1
Inj Volume : 10.000 µl
Method : C:\Chem32\1\Data\Sir2 Purity test MeCN_rui 2016-04-15 16-44-07\Sir2 purity_30-100%MeCN.M (Sequence Method)
Last changed : 4/15/2016 4:44:07 PM by SYSTEM
Method Info : test

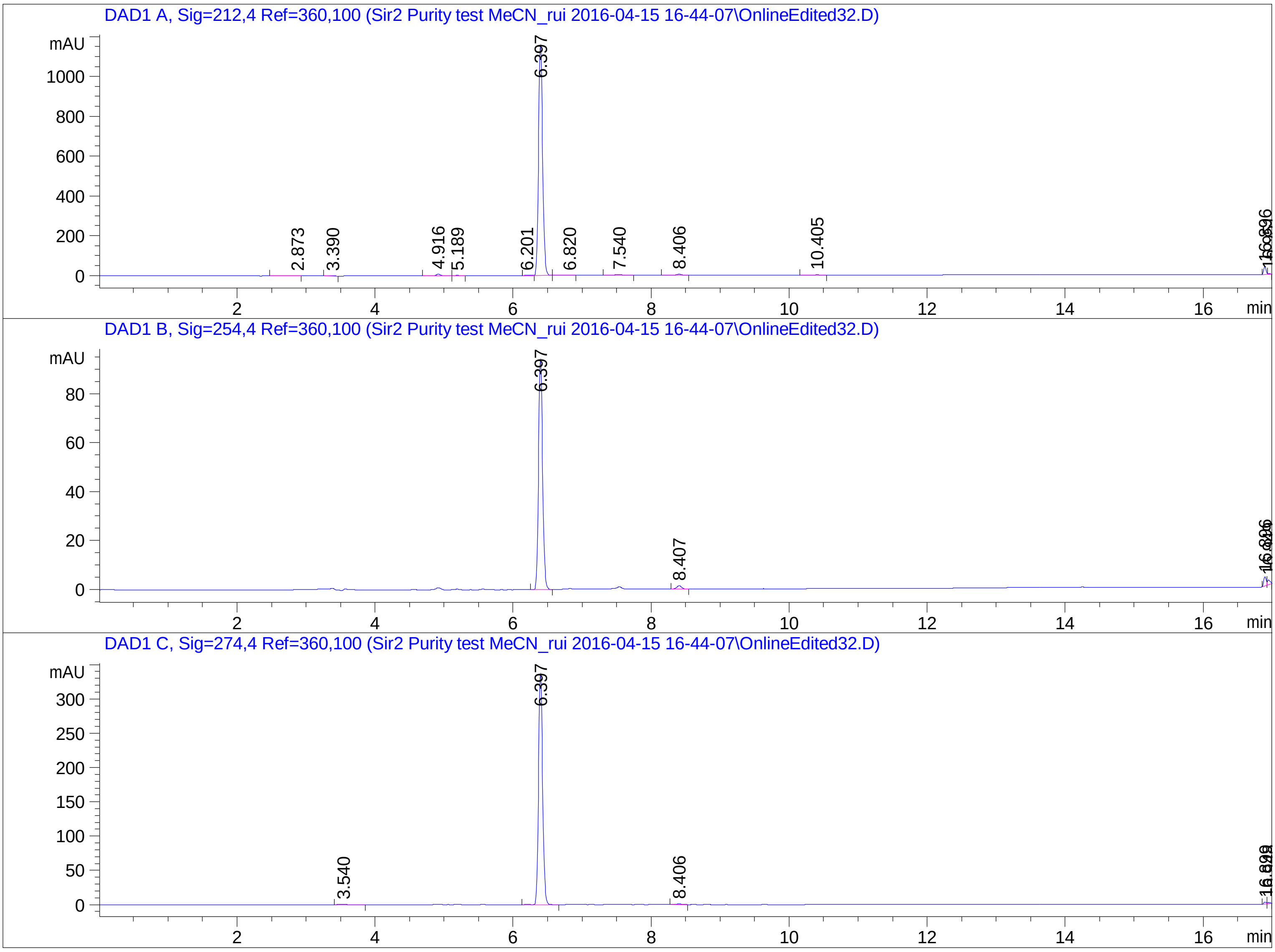

===

Area Percent Report

===

Sorted By : Signal
Multiplier : 1.0000
Dilution : 1.0000
Use Multiplier & Dilution Factor with ISTDs

Signal 1: DAD1 A, Sig=212,4 Ref=360,100

Peak #	RetTime [min]	Type	Width [min]	Area [mAU*s]	Height [mAU]	Area %
1	2.873	BV	0.1379	14.12148	1.37437	0.2843
2	3.390	BB	0.0870	13.01250	2.13705	0.2620
3	4.916	BV R	0.0723	36.58270	7.58489	0.7366
4	5.189	VB	0.0668	7.87881	1.80839	0.1586
5	6.201	BV E	0.0624	8.82808	2.13006	0.1777
6	6.397	VB R	0.0635	4702.12256	1155.73206	94.6727
7	6.820	BV	0.1031	11.27499	1.66391	0.2270
8	7.540	VV R	0.1107	29.21206	3.59896	0.5882
9	8.406	BB	0.0778	25.77858	5.02815	0.5190
10	10.405	BB	0.0757	6.81076	1.37705	0.1371
11	16.896	BV R	0.0416	101.61607	40.05394	2.0459
12	16.951	VBAE	0.0377	9.47649	3.98946	0.1908

Totals : 4966.71510 1226.47829

Signal 2: DAD1 B, Sig=254,4 Ref=360,100

Peak #	RetTime [min]	Type	Width [min]	Area [mAU*s]	Height [mAU]	Area %
1	6.397	BB	0.0632	380.62216	94.03521	94.7648
2	8.407	BB	0.0732	6.19297	1.30965	1.5419
3	16.896	BV	0.0418	9.75478	3.81336	2.4287
4	16.949	VBA	0.0410	5.07924	1.91417	1.2646

Totals : 401.64915 101.07239

Signal 3: DAD1 C, Sig=274,4 Ref=360,100

Peak #	RetTime [min]	Type	Width [min]	Area [mAU*s]	Height [mAU]	Area %
1	3.540	BB	0.1247	7.50122	1.00989	0.5389
2	6.397	VB R	0.0634	1366.12732	336.39005	98.1536
3	8.406	BB	0.0743	5.71022	1.18403	0.4103
4	16.899	BV	0.0428	7.44843	2.82314	0.5352
5	16.948	VBA	0.0418	5.03917	1.85060	0.3621

Totals : 1391.82636 343.25771

===

*** End of Report ***

Data File C:\Chem32\1\Data\Sir2 Purity test MeCN_rui 2016-04-18 20-25-03\1FD-0401.D
Sample Name: LC0535

Compound 19c

===

Acq. Operator : SYSTEM Seq. Line : 4
Acq. Instrument : CDD Location : P1-F-04
Injection Date : 4/18/2016 9:20:44 PM Inj : 1
Inj Volume : 10.000 µl
Method : C:\Chem32\1\Data\Sir2 Purity test MeCN_rui 2016-04-18 20-25-03\Sir2 purity_30-100%MeCN.M (Sequence Method)
Last changed : 4/18/2016 8:25:03 PM by SYSTEM
Method Info : test

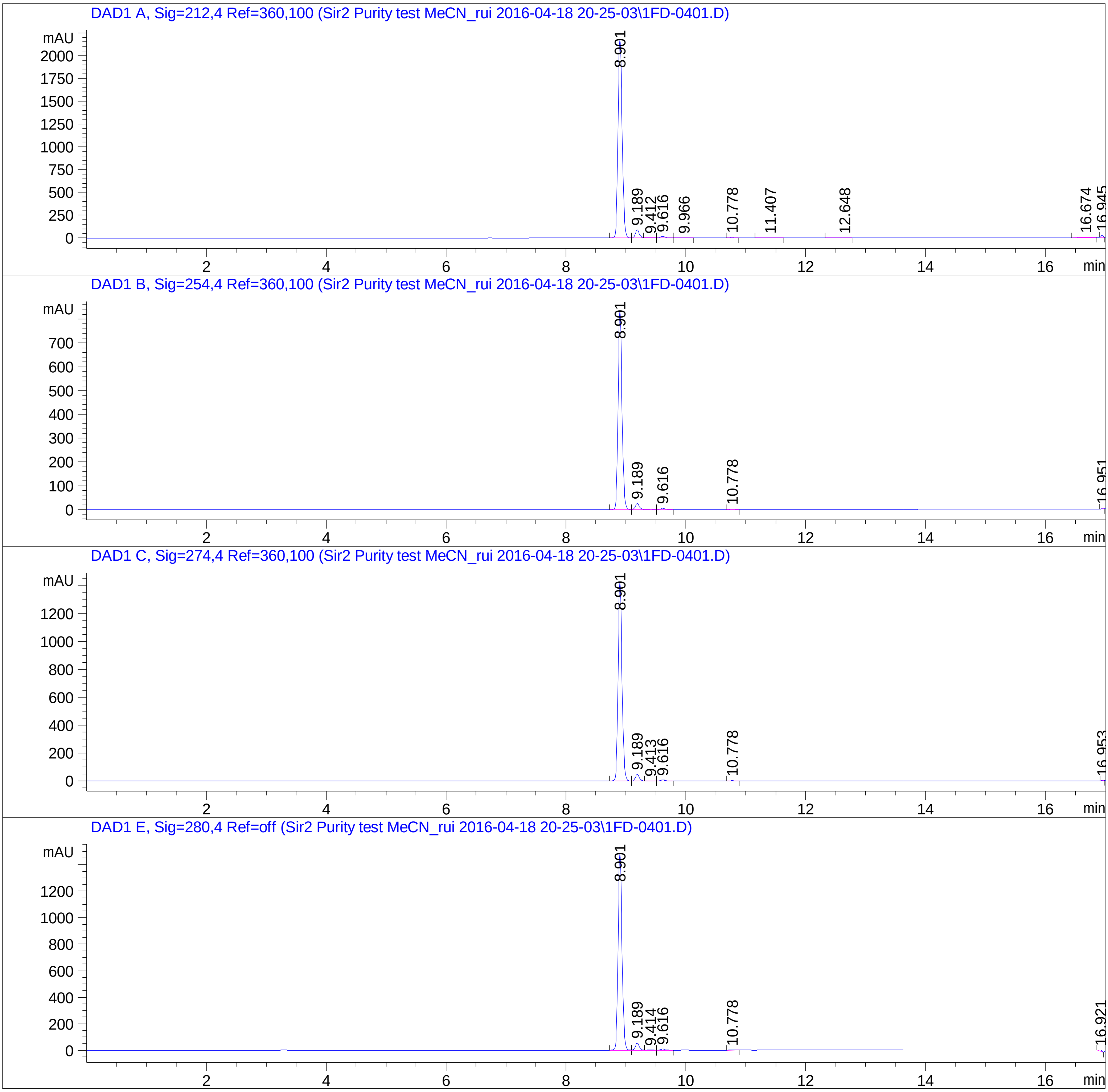

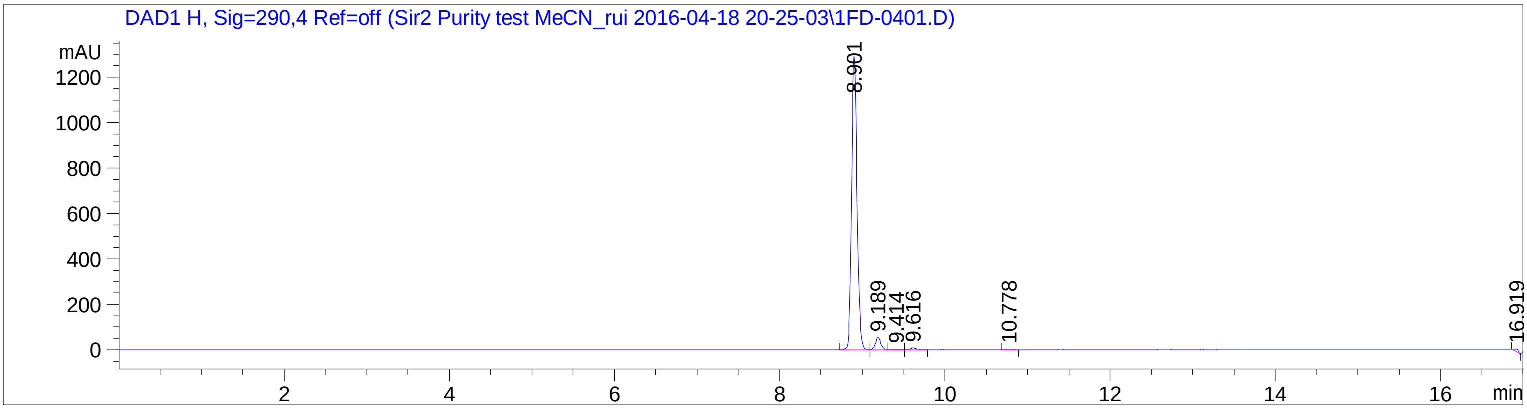

===

Area Percent Report

===

Sorted By : Signal
Multiplier : 1.0000
Dilution : 1.0000
Use Multiplier & Dilution Factor with ISTDs

Signal 1: DAD1 A, Sig=212,4 Ref=360,100

Peak #	RetTime [min]	Type	Width [min]	Area [mAU*s]	Height [mAU]	Area %
1	8.901	BV	0.0770	1.06217e4	2173.48511	94.2936
2	9.189	VV R	0.0718	406.39020	88.16858	3.6077
3	9.412	VB E	0.0741	16.09970	3.34966	0.1429
4	9.616	BB	0.0735	84.63209	17.78101	0.7513
5	9.966	BB	0.0990	10.91131	1.57113	0.0969
6	10.778	BB	0.0751	21.68983	4.59571	0.1926
7	11.407	BB	0.1269	11.40628	1.24444	0.1013
8	12.648	BB	0.1094	14.39342	1.83672	0.1278
9	16.674	BB	0.1529	23.59062	2.20396	0.2094
10	16.945	BBA	0.0398	53.68252	22.52287	0.4766

Totals : 1.12645e4 2316.75919

Signal 2: DAD1 B, Sig=254,4 Ref=360,100

Peak #	RetTime [min]	Type	Width [min]	Area [mAU*s]	Height [mAU]	Area %
1	8.901	BV	0.0712	3802.88037	833.86719	95.9560
2	9.189	VV R	0.0740	120.63305	25.12811	3.0439
3	9.616	BB	0.0737	23.43620	4.90456	0.5914
4	10.778	BB	0.0758	8.79339	1.83971	0.2219
5	16.951	BB	0.0396	7.40627	3.13137	0.1869

Totals : 3963.14928 868.87094

Signal 3: DAD1 C, Sig=274,4 Ref=360,100

Peak #	RetTime [min]	Type	Width [min]	Area [mAU*s]	Height [mAU]	Area %
1	8.901	BV	0.0713	6504.47266	1422.51550	95.9255
2	9.189	VV R	0.0718	216.89243	47.06205	3.1986
3	9.413	VB E	0.0742	7.38368	1.53145	0.1089
4	9.616	BB	0.0737	35.77792	7.49066	0.5276
5	10.778	BB	0.0755	9.55797	2.01080	0.1410
6	16.953	BB	0.0398	6.67313	2.80312	0.0984

Totals : 6780.75777 1483.41359

Signal 4: DAD1 E, Sig=280,4 Ref=off

Peak #	RetTime [min]	Type	Width [min]	Area [mAU*s]	Height [mAU]	Area %
1	8.901	BV	0.0714	6788.86035	1482.45166	95.2473
2	9.189	VV R	0.0717	246.16814	53.47733	3.4537
3	9.414	VB E	0.0742	7.49099	1.55474	0.1051
4	9.616	BB	0.0737	37.93104	7.93981	0.5322
5	10.778	BB	0.0756	9.53864	2.00186	0.1338
6	16.921	BB	0.0565	37.62646	11.10863	0.5279

Totals : 7127.61561 1558.53403

Signal 5: DAD1 H, Sig=290,4 Ref=off

Peak #	RetTime [min]	Type	Width [min]	Area [mAU*s]	Height [mAU]	Area %
1	8.901	BV	0.0714	5929.82666	1294.46326	94.7530
2	9.189	VV R	0.0716	242.29263	52.69270	3.8716
3	9.414	VB E	0.0735	6.37546	1.33959	0.1019
4	9.616	BB	0.0737	34.72555	7.26909	0.5549
5	10.778	BB	0.0753	8.14623	1.71871	0.1302
6	16.919	BB	0.0547	36.82909	11.21660	0.5885

Totals : 6258.19563 1368.69995

===

*** End of Report ***

Data File C:\Chem32\1\Data\Sir2 Purity test MeCN_rui 2016-04-15 16-44-07\OnlineEdited34.D
Sample Name: LC0541

Compound 20a

===

Acq. Operator : SYSTEM Seq. Line : 36
Acq. Instrument : CDD Location : P1-D-09
Injection Date : 4/16/2016 3:13:05 AM Inj : 1
Inj Volume : 10.000 µl
Method : C:\Chem32\1\Data\Sir2 Purity test MeCN_rui 2016-04-15 16-44-07\Sir2 purity_30-100%MeCN.M (Sequence Method)
Last changed : 4/15/2016 4:44:07 PM by SYSTEM
Method Info : test

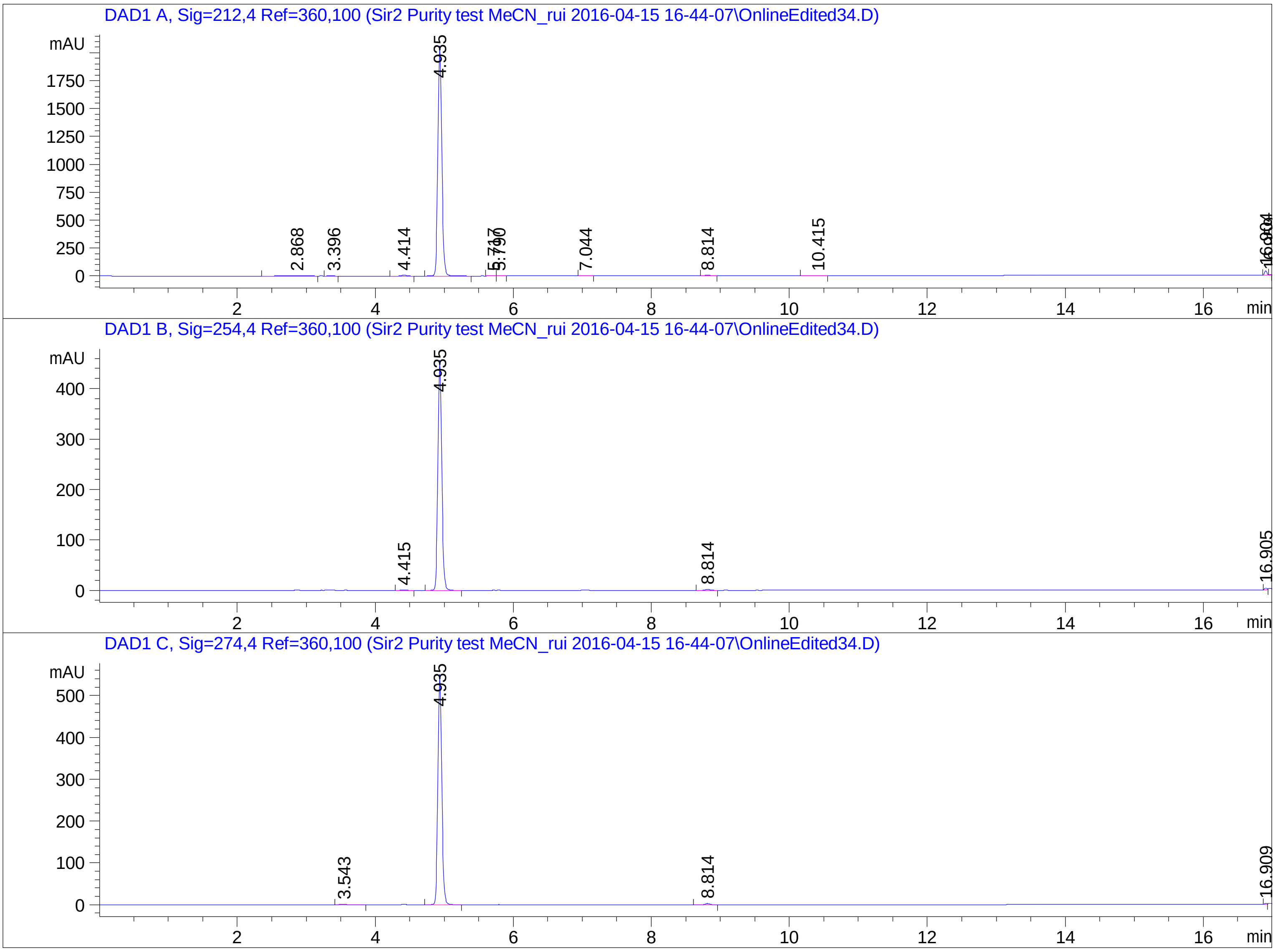

===

Area Percent Report

===

Sorted By : Signal
Multiplier : 1.0000
Dilution : 1.0000
Use Multiplier & Dilution Factor with ISTDs

Signal 1: DAD1 A, Sig=212,4 Ref=360,100

Peak #	RetTime [min]	Type	Width [min]	Area [mAU*s]	Height [mAU]	Area %
1	2.868	BB	0.3395	65.97676	2.39425	0.7685
2	3.396	BB	0.0837	11.13997	1.86584	0.1298
3	4.414	BB	0.0735	37.01006	7.77628	0.4311
4	4.935	BB	0.0629	8301.71875	2065.33374	96.7016
5	5.717	BV	0.0535	6.47774	1.82222	0.0755
6	5.790	VV	0.0608	8.82790	2.11093	0.1028
7	7.044	BB	0.0975	11.83717	1.98621	0.1379
8	8.814	BB	0.0679	25.46918	5.72301	0.2967
9	10.415	BB	0.0749	6.95381	1.42501	0.0810
10	16.904	BV R	0.0414	104.25413	41.33171	1.2144
11	16.959	VBAE	0.0321	5.21862	2.71218	0.0608
Totals :				8584.88408	2134.48137	

Signal 2: DAD1 B, Sig=254,4 Ref=360,100

Peak #	RetTime [min]	Type	Width [min]	Area [mAU*s]	Height [mAU]	Area %
1	4.415	BB	0.0741	5.96790	1.24008	0.3256
2	4.935	BB	0.0622	1809.89917	456.98740	98.7525
3	8.814	BB	0.0682	8.10324	1.80988	0.4421
4	16.905	BV	0.0418	8.79244	3.43676	0.4797
Totals :				1832.76276	463.47413	

Signal 3: DAD1 C, Sig=274,4 Ref=360,100

Peak #	RetTime [min]	Type	Width [min]	Area [mAU*s]	Height [mAU]	Area %
1	3.543	BB	0.1258	8.93170	1.18716	0.4041
2	4.935	BB	0.0622	2181.22754	550.86676	98.6808
3	8.814	BB	0.0684	13.88230	3.09290	0.6280
4	16.909	BV	0.0425	6.34632	2.42631	0.2871
Totals :				2210.38786	557.57313	

*** End of Report ***

Compound 20b

===

Acq. Operator : SYSTEM Seq. Line : 37
Acq. Instrument : CDD Location : P1-E-01
Injection Date : 4/16/2016 3:31:02 AM Inj : 1
Inj Volume : 10.000 µl
Method : C:\Chem32\1\Data\Sir2 Purity test MeCN_rui 2016-04-15 16-44-07\Sir2 purity_30-100%MeCN.M (Sequence Method)
Last changed : 4/15/2016 4:44:07 PM by SYSTEM
Method Info : test

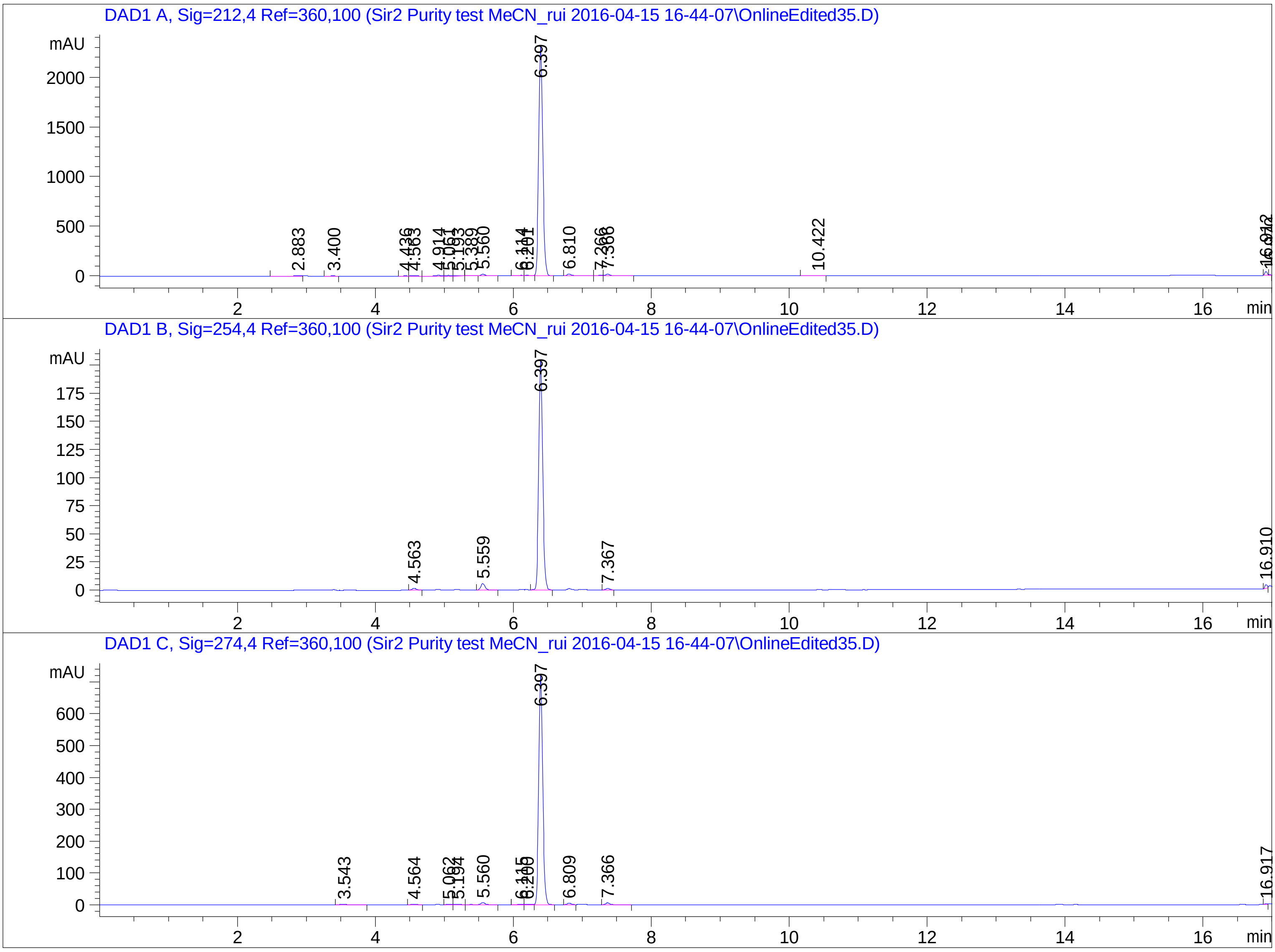

===

Area Percent Report

===

Sorted By : Signal
Multiplier : 1.0000
Dilution : 1.0000
Use Multiplier & Dilution Factor with ISTDs

Signal 1: DAD1 A, Sig=212,4 Ref=360,100

Peak #	RetTime [min]	Type	Width [min]	Area [mAU*s]	Height [mAU]	Area %
1	2.883	BV	0.1543	16.79104	1.43720	0.1639
2	3.400	BB	0.0903	11.91254	1.97618	0.1163
3	4.436	BV	0.0757	5.84956	1.22595	0.0571
4	4.563	VB	0.0736	23.70037	4.97365	0.2314
5	4.914	BV	0.0661	31.54052	7.34483	0.3080
6	5.061	VV	0.0602	23.88740	6.03366	0.2332
7	5.193	VV	0.0767	22.21590	4.41552	0.2169
8	5.389	VV E	0.0790	8.48833	1.57082	0.0829
9	5.560	VB R	0.0727	78.96269	16.85054	0.7710
10	6.114	BV E	0.0751	23.15332	4.57408	0.2261
11	6.201	VV E	0.0788	28.88398	5.19916	0.2820
12	6.397	VB R	0.0644	9667.82422	2329.07349	94.3974
13	6.810	BV R	0.0727	81.48457	16.77758	0.7956
14	7.266	BV	0.0608	22.20163	5.53192	0.2168
15	7.366	VB	0.0718	82.19278	17.19473	0.8025
16	10.422	BB	0.0746	6.85558	1.41209	0.0669
17	16.912	BV R	0.0403	99.71455	41.16579	0.9736
18	16.970	VBAE	0.0318	5.96324	3.12992	0.0582

Totals : 1.02416e4 2469.88710

Signal 2: DAD1 B, Sig=254,4 Ref=360,100

Peak #	RetTime [min]	Type	Width [min]	Area [mAU*s]	Height [mAU]	Area %
1	4.563	BB	0.0701	7.72457	1.72887	0.8826
2	5.559	BB	0.0680	25.69799	5.76098	2.9363
3	6.397	BB	0.0629	827.57452	205.72047	94.5603
4	7.367	BB	0.0658	5.94078	1.39272	0.6788
5	16.910	BV	0.0404	8.24393	3.38779	0.9420

Totals : 875.18180 217.99084

Signal 3: DAD1 C, Sig=274,4 Ref=360,100

Peak #	RetTime [min]	Type	Width [min]	Area [mAU*s]	Height [mAU]	Area %
1	3.543	BB	0.1231	8.46486	1.16108	0.2753
2	4.564	BB	0.0715	9.53507	2.08124	0.3101
3	5.062	BV	0.0627	5.85406	1.40154	0.1904
4	5.194	VV	0.0784	9.37281	1.80904	0.3048
5	5.560	VB R	0.0768	33.88279	6.72347	1.1019
6	6.115	BV E	0.0765	7.77982	1.50102	0.2530
7	6.200	VV E	0.0687	7.16804	1.47553	0.2331
8	6.397	VB R	0.0631	2940.04883	727.78058	95.6173

Peak #	RetTime [min]	Type	Width [min]	Area [mAU*s]	Height [mAU]	Area %
9	6.809	BB	0.0625	18.55397	4.65546	0.6034
10	7.366	BB	0.0718	28.15342	5.88808	0.9156
11	16.917	BV	0.0421	5.99340	2.31978	0.1949

Totals : 3074.80706 756.79680

==

*** End of Report ***

Data File C:\Chem32\1\Data\Sir2 Purity test MeCN_rui 2016-04-19 19-34-54\1FF-0201.D
Sample Name: LC0561

Compound 20c

===

Acq. Operator	: SYSTEM	Seq. Line :	2
Acq. Instrument	: CDD	Location :	P1-F-06
Injection Date	: 4/19/2016 7:54:27 PM	Inj :	1
		Inj Volume :	10.000 µl

Method : C:\Chem32\1\Data\Sir2 Purity test MeCN_rui 2016-04-19 19-34-54\Sir2 purity_30-100%MeCN.M (Sequence Method)
Last changed : 4/19/2016 7:34:54 PM by SYSTEM
Method Info : test

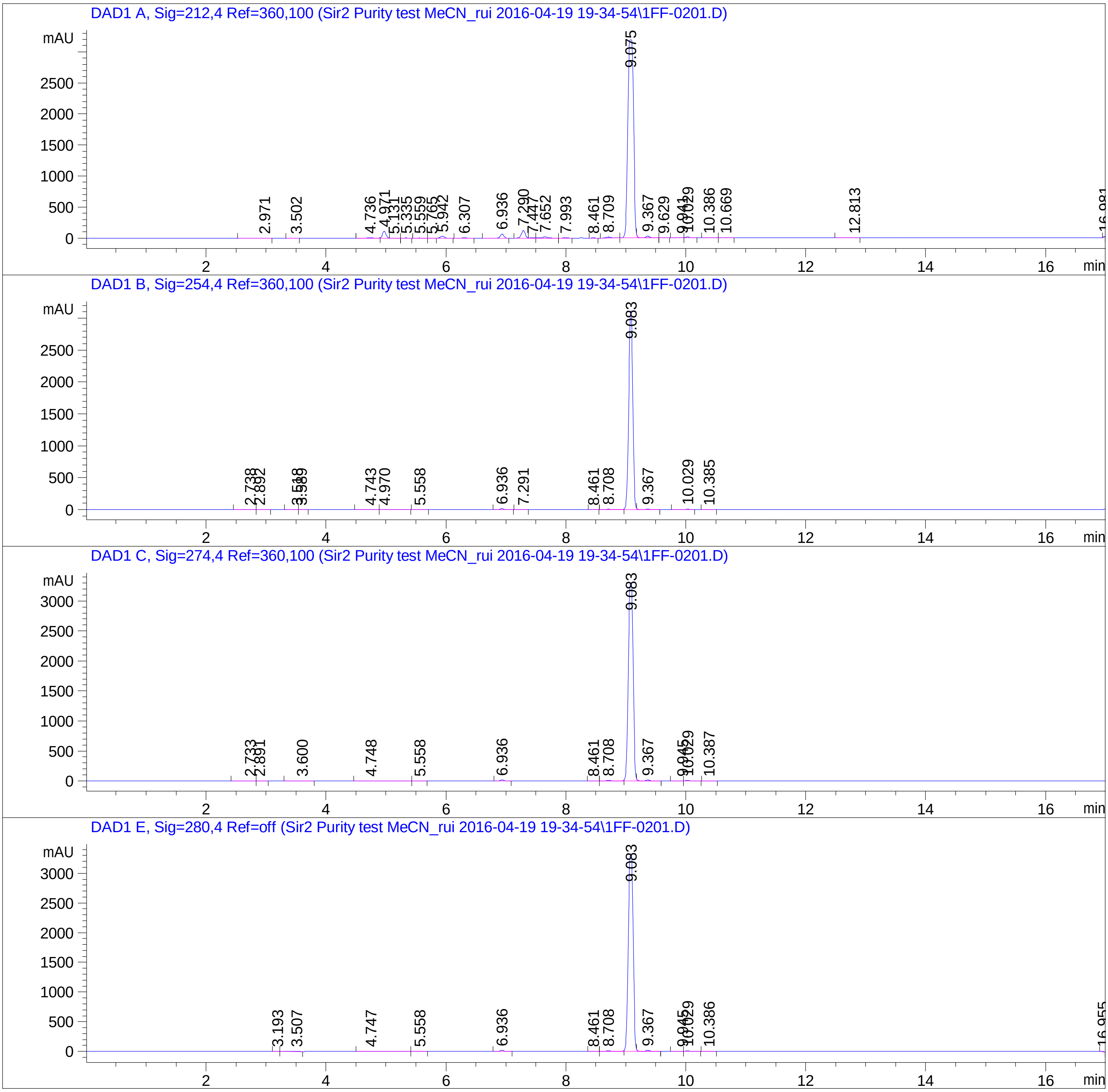

Data File C:\Chem32\1\Data\Sir2 Purity test MeCN_rui 2016-04-19 19-34-54\1FF-0201.D
Sample Name: LC0561

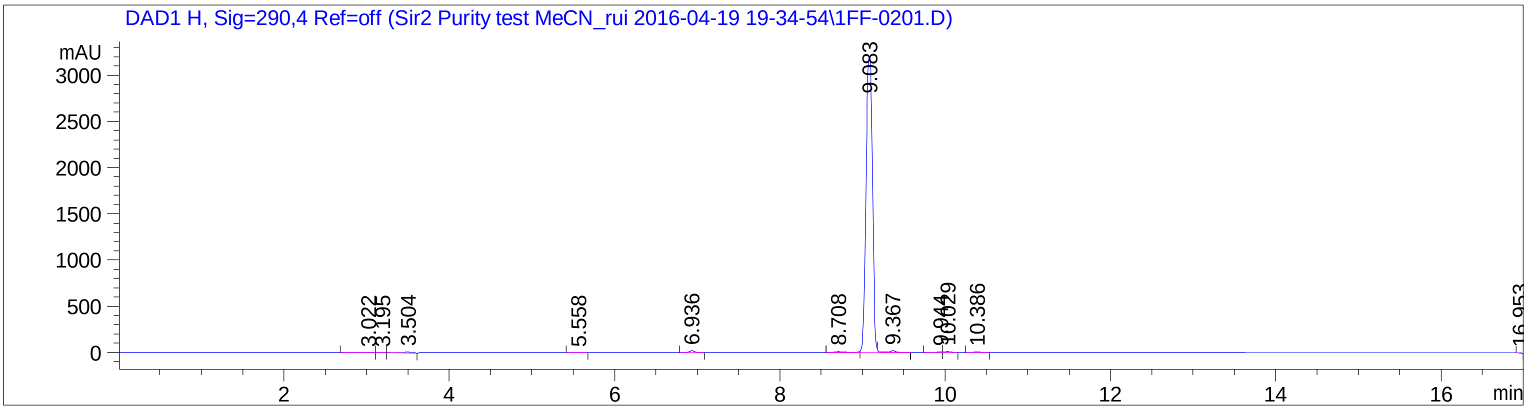

===

Area Percent Report

===

Sorted By : Signal
Multiplier : 1.0000
Dilution : 1.0000
Use Multiplier & Dilution Factor with ISTDs

Signal 1: DAD1 A, Sig=212,4 Ref=360,100

Peak #	RetTime [min]	Type	Width [min]	Area [mAU*s]	Height [mAU]	Area %
1	2.971	BB	0.2929	23.97148	1.08872	0.1032
2	3.502	BB	0.0899	14.01491	2.27523	0.0604
3	4.736	BV E	0.0972	62.34415	8.96105	0.2685
4	4.971	VV R	0.0648	479.99155	114.85512	2.0669
5	5.131	VB E	0.0716	7.13292	1.44495	0.0307
6	5.335	BB	0.0632	14.15882	3.49929	0.0610
7	5.559	BV E	0.0731	16.05919	3.27905	0.0692
8	5.765	VV E	0.0624	8.85818	2.13411	0.0381
9	5.942	VB R	0.0690	140.88983	31.04236	0.6067
10	6.307	BB	0.0856	25.62046	4.42252	0.1103
11	6.936	BB	0.0619	262.16064	66.60017	1.1289
12	7.290	BV R	0.0702	548.85016	122.67003	2.3635
13	7.447	VV E	0.0693	18.03903	3.95026	0.0777
14	7.652	VB E	0.0833	114.17487	20.39448	0.4917
15	7.993	BB	0.0828	16.12177	2.99473	0.0694
16	8.461	VB	0.0616	7.01831	1.79733	0.0302
17	8.709	BB	0.0895	89.84274	15.08728	0.3869
18	9.075	BV R	0.0928	2.10154e4	3192.60181	90.4962
19	9.367	VB E	0.0799	163.21011	29.80590	0.7028
20	9.629	BB	0.0799	5.86247	1.18199	0.0252
21	9.941	BV	0.0698	33.67329	7.03749	0.1450
22	10.029	VB	0.0734	80.61089	16.98809	0.3471
23	10.386	BV	0.0794	24.73715	4.69921	0.1065
24	10.669	VB	0.0935	11.22548	1.73477	0.0483
25	12.813	BB	0.1532	13.95376	1.20395	0.0601
26	16.981	BBA	0.0329	24.48950	12.39571	0.1055

Totals : 2.32224e4 3674.14560

Signal 2: DAD1 B, Sig=254,4 Ref=360,100

Peak #	RetTime [min]	Type	Width [min]	Area [mAU*s]	Height [mAU]	Area %
1	2.738	BV	0.1971	31.17409	2.07719	0.2091
2	2.892	VB	0.1095	12.24975	1.71201	0.0822
3	3.518	BV	0.0703	13.80363	2.86024	0.0926
4	3.589	VB	0.0976	22.42932	3.65286	0.1504
5	4.743	BV	0.1834	29.45404	2.36261	0.1976
6	4.970	VB	0.1071	27.11887	3.40005	0.1819
7	5.558	BB	0.0609	6.04995	1.50622	0.0406
8	6.936	BB	0.0612	62.34792	16.10277	0.4182
9	7.291	BB	0.0688	11.20710	2.57348	0.0752
10	8.461	BB	0.0622	5.47523	1.38207	0.0367
11	8.708	BV E	0.0989	35.39451	5.23376	0.2374
12	9.083	VV R	0.0746	1.45586e4	3113.62842	97.6470
13	9.367	VB E	0.0851	49.32701	8.32594	0.3308
14	10.029	BB	0.0905	35.51141	5.71699	0.2382
15	10.385	BB	0.0749	9.27129	1.90181	0.0622

Totals : 1.49094e4 3172.43642

Signal 3: DAD1 C, Sig=274,4 Ref=360,100

Peak #	RetTime [min]	Type	Width [min]	Area [mAU*s]	Height [mAU]	Area %
1	2.733	BV	0.2013	34.67241	2.23154	0.1838
2	2.891	VB	0.1110	12.65983	1.82239	0.0671
3	3.600	BB	0.1253	40.47093	4.75257	0.2145
4	4.748	BB	0.2824	51.86138	2.51643	0.2749
5	5.558	BB	0.0600	5.85442	1.48354	0.0310
6	6.936	BB	0.0613	78.48625	20.23083	0.4160
7	8.461	VB	0.0629	6.65946	1.65669	0.0353
8	8.708	BV E	0.0993	57.54268	8.46605	0.3050
9	9.083	VV R	0.0909	1.84154e4	3309.42480	97.6167
10	9.367	VB E	0.0826	88.95346	15.57627	0.4715
11	9.945	BV	0.0608	14.37816	3.43916	0.0762
12	10.029	VB	0.0735	46.85403	9.84088	0.2484
13	10.387	BB	0.0786	11.20825	2.23237	0.0594

Totals : 1.88650e4 3383.67351

Signal 4: DAD1 E, Sig=280,4 Ref=off

Peak #	RetTime [min]	Type	Width [min]	Area [mAU*s]	Height [mAU]	Area %
1	3.193	BV	0.0718	7.22377	1.45836	0.0377
2	3.507	VB	0.1553	75.71667	6.43261	0.3946
3	4.747	BB	0.2898	42.30599	2.02454	0.2205

Peak #	RetTime [min]	Type	Width [min]	Area [mAU*s]	Height [mAU]	Area %
4	5.558	BB	0.0604	7.26961	1.82726	0.0379
5	6.936	BB	0.0613	81.83537	21.06169	0.4265
6	8.461	VB	0.0626	6.08404	1.52410	0.0317
7	8.708	BV E	0.0995	61.95412	9.09416	0.3229
8	9.083	VV R	0.0935	1.86966e4	3328.13403	97.4499
9	9.367	VB E	0.0813	98.97906	17.69429	0.5159
10	9.945	BV	0.0609	15.44033	3.68574	0.0805
11	10.029	VB	0.0734	48.94416	10.29948	0.2551
12	10.386	BB	0.0753	15.74745	3.20423	0.0821
13	16.955	BBA	0.0423	27.76842	9.45545	0.1447

Totals : 1.91859e4 3415.89594

Signal 5: DAD1 H, Sig=290,4 Ref=off

Peak #	RetTime [min]	Type	Width [min]	Area [mAU*s]	Height [mAU]	Area %
1	3.022	BV	0.1953	47.24429	3.18072	0.2635
2	3.195	VV	0.0964	31.49477	4.45747	0.1757
3	3.504	VB	0.2045	116.55663	7.44996	0.6501
4	5.558	BB	0.0603	7.24010	1.82488	0.0404
5	6.936	BB	0.0614	73.50731	18.90821	0.4100
6	8.708	BV E	0.0996	60.04515	8.80069	0.3349
7	9.083	VV R	0.0893	1.73973e4	3207.60718	97.0399
8	9.367	VB E	0.0793	94.28597	17.37395	0.5259
9	9.944	BV	0.0608	14.10291	3.37004	0.0787
10	10.029	VB	0.0731	42.58501	9.01387	0.2375
11	10.386	BB	0.0752	16.70947	3.40845	0.0932
12	16.953	BBA	0.0488	26.91956	9.19387	0.1502

Totals : 1.79280e4 3294.58930

==
*** End of Report ***

Compound 21a

===

Acq. Operator : SYSTEM Seq. Line : 39
Acq. Instrument : CDD Location : P1-E-03
Injection Date : 4/16/2016 4:06:52 AM Inj : 1
Inj Volume : 10.000 µl
Method : C:\Chem32\1\Data\Sir2 Purity test MeCN_rui 2016-04-15 16-44-07\Sir2 purity_30-100%MeCN.M (Sequence Method)
Last changed : 4/15/2016 4:44:07 PM by SYSTEM
Method Info : test

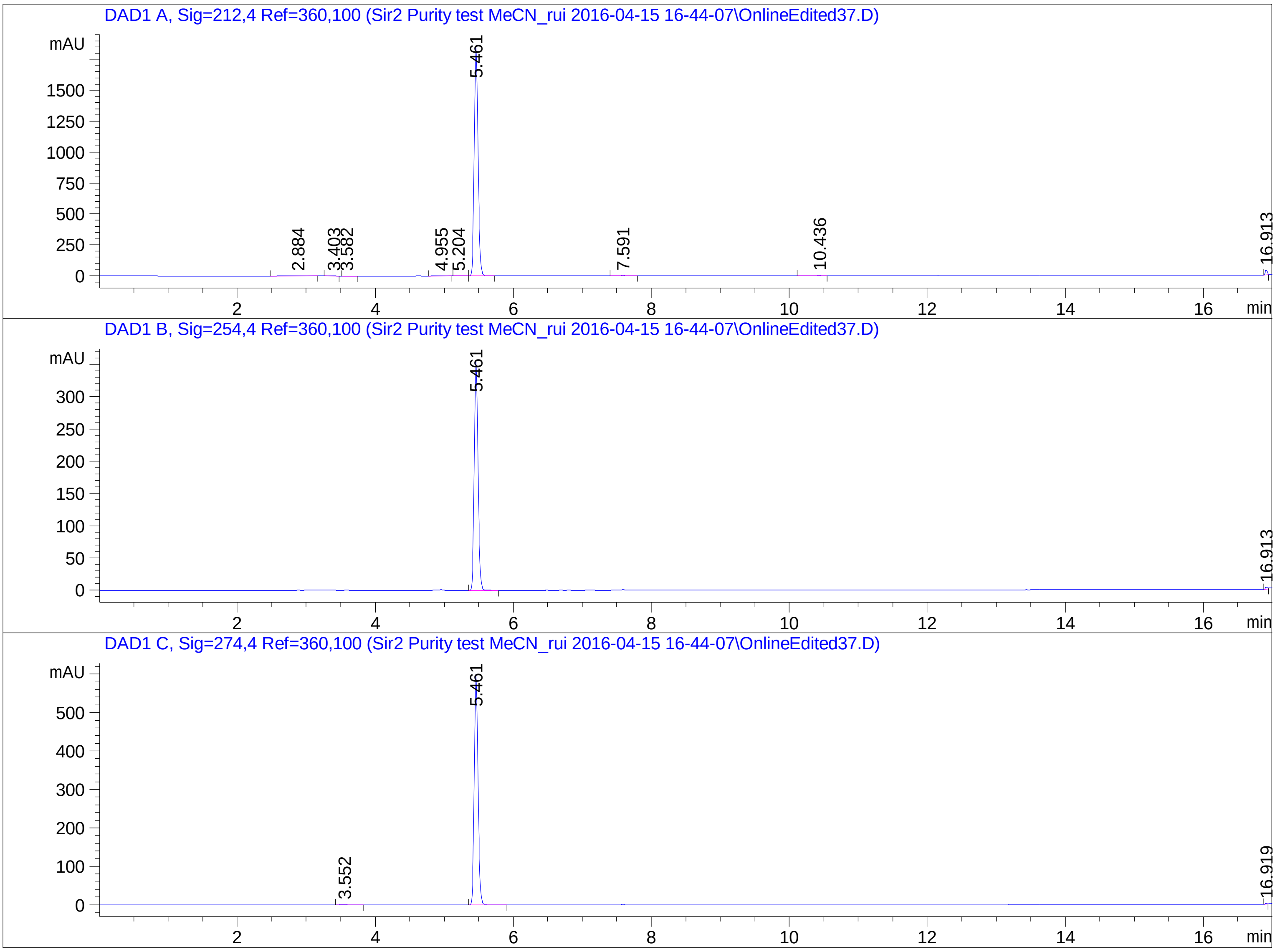

===

Area Percent Report

===

Sorted By : Signal
Multiplier : 1.0000
Dilution : 1.0000
Use Multiplier & Dilution Factor with ISTDs

Signal 1: DAD1 A, Sig=212,4 Ref=360,100

Peak #	RetTime [min]	Type	Width [min]	Area [mAU*s]	Height [mAU]	Area %
1	2.884	BB	0.2049	22.44945	1.40167	0.3042
2	3.403	BB	0.0911	13.65471	2.17994	0.1850
3	3.582	BB	0.0899	9.84433	1.51260	0.1334
4	4.955	VB R	0.0719	17.40546	3.63516	0.2358
5	5.204	BB	0.0622	6.94315	1.67997	0.0941
6	5.461	BB	0.0610	7194.17529	1866.08276	97.4777
7	7.591	BB	0.0776	15.78719	3.09088	0.2139
8	10.436	BB	0.0740	6.78692	1.41333	0.0920
9	16.913	BB	0.0394	93.27946	39.74961	1.2639

Totals : 7380.32596 1920.74593

Signal 2: DAD1 B, Sig=254,4 Ref=360,100

Peak #	RetTime [min]	Type	Width [min]	Area [mAU*s]	Height [mAU]	Area %
1	5.461	BB	0.0605	1366.16907	357.88062	99.4488
2	16.913	BV	0.0405	7.57207	3.10375	0.5512

Totals : 1373.74114 360.98436

Signal 3: DAD1 C, Sig=274,4 Ref=360,100

Peak #	RetTime [min]	Type	Width [min]	Area [mAU*s]	Height [mAU]	Area %
1	3.552	BB	0.1263	8.07735	1.06699	0.3501
2	5.461	BB	0.0605	2293.74487	600.84644	99.4152
3	16.919	BV	0.0426	5.41542	2.06471	0.2347

Totals : 2307.23765 603.97814

===

*** End of Report ***

Data File C:\Chem32\1\Data\Sir2 Purity test MeCN_rui 2016-04-15 16-44-07\OnlineEdited38.D
Sample Name: LC0539

Compound 21b

===

Acq. Operator	: SYSTEM	Seq. Line :	40
Acq. Instrument	: CDD	Location :	P1-E-04
Injection Date	: 4/16/2016 4:24:47 AM	Inj :	1
		Inj Volume :	10.000 µl

Method : C:\Chem32\1\Data\Sir2 Purity test MeCN_rui 2016-04-15 16-44-07\Sir2 purity_30-100%MeCN.M (Sequence Method)
Last changed : 4/15/2016 4:44:07 PM by SYSTEM
Method Info : test

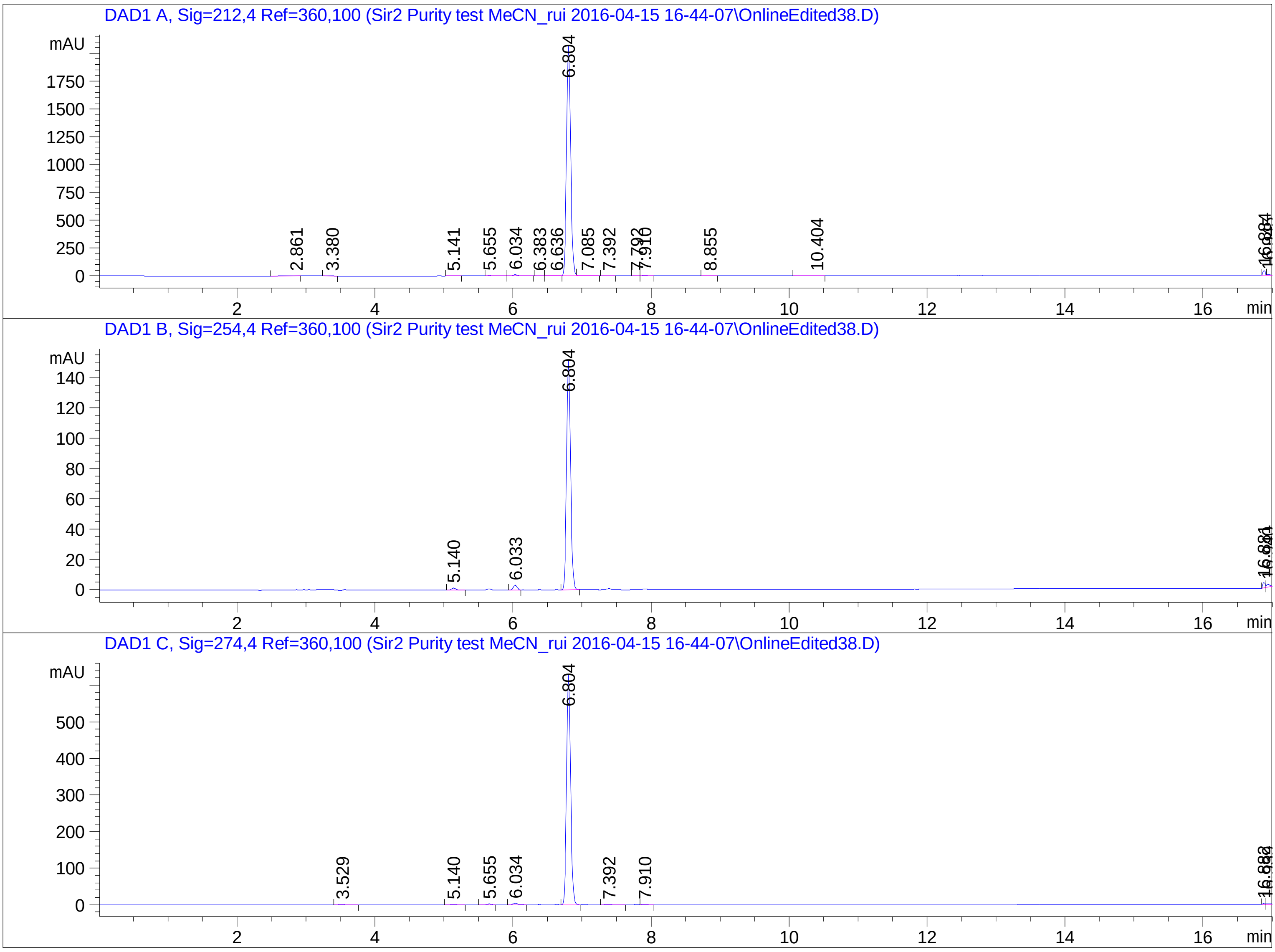

===

Area Percent Report

===

Sorted By : Signal
Multiplier : 1.0000
Dilution : 1.0000
Use Multiplier & Dilution Factor with ISTDs

Signal 1: DAD1 A, Sig=212,4 Ref=360,100

Peak #	RetTime [min]	Type	Width [min]	Area [mAU*s]	Height [mAU]	Area %
1	2.861	BV	0.1160	10.39958	1.23792	0.1149
2	3.380	BB	0.0880	13.33332	2.16097	0.1474
3	5.141	BB	0.0693	17.57088	3.99485	0.1942
4	5.655	VV R	0.0728	26.43756	5.43317	0.2922
5	6.034	BV R	0.0865	58.50658	9.95825	0.6466
6	6.383	BB	0.0601	5.82342	1.54158	0.0644
7	6.636	BV E	0.0668	11.84626	2.72000	0.1309
8	6.804	VV R	0.0651	8729.20703	2073.64307	96.4724
9	7.085	VB E	0.1342	12.77665	1.24056	0.1412
10	7.392	BB	0.0741	10.24076	1.98763	0.1132
11	7.792	BV	0.0646	6.37903	1.53234	0.0705
12	7.910	VB	0.0702	20.19344	4.34787	0.2232
13	8.855	BB	0.0824	5.35865	1.00208	0.0592
14	10.404	BB	0.0729	7.19528	1.47652	0.0795
15	16.884	BV R	0.0404	101.00175	41.43216	1.1162
16	16.945	VBAE	0.0436	12.12395	4.47588	0.1340

Totals : 9048.39415 2158.18483

Signal 2: DAD1 B, Sig=254,4 Ref=360,100

Peak #	RetTime [min]	Type	Width [min]	Area [mAU*s]	Height [mAU]	Area %
1	5.140	BB	0.0685	5.67379	1.31106	0.8668
2	6.033	BB	0.0647	12.09474	3.02032	1.8478
3	6.804	BB	0.0637	622.72363	152.32570	95.1385
4	16.881	BV	0.0400	8.23631	3.43242	1.2583
5	16.940	VBA	0.0446	5.81608	1.96173	0.8886

Totals : 654.54455 162.05124

Signal 3: DAD1 C, Sig=274,4 Ref=360,100

Peak #	RetTime [min]	Type	Width [min]	Area [mAU*s]	Height [mAU]	Area %
1	3.529	BB	0.1036	7.18098	1.02688	0.2684
2	5.140	BB	0.0685	8.15334	1.88176	0.3048
3	5.655	BB	0.0698	10.34470	2.24309	0.3867
4	6.034	BB	0.0729	20.36735	4.33076	0.7613
5	6.804	BB	0.0641	2603.33862	631.55505	97.3090
6	7.392	BB	0.0936	6.77986	1.01962	0.2534
7	7.910	VB	0.0715	6.72201	1.41386	0.2513
8	16.882	BV	0.0411	6.44055	2.57811	0.2407
9	16.939	VBA	0.0453	6.00471	1.98177	0.2244

Totals : 2675.33212 648.03090

===

*** End of Report ***

Data File C:\Chem32\1\Data\Sir2 Purity test MeCN_rui 2016-04-15 16-44-07\OnlineEdited39.D
Sample Name: LC0538

Compound 21c

===

Acq. Operator : SYSTEM Seq. Line : 41
Acq. Instrument : CDD Location : P1-E-05
Injection Date : 4/16/2016 4:42:43 AM Inj : 1
Inj Volume : 10.000 µl
Method : C:\Chem32\1\Data\Sir2 Purity test MeCN_rui 2016-04-15 16-44-07\Sir2 purity_30-100%MeCN.M (Sequence Method)
Last changed : 4/15/2016 4:44:07 PM by SYSTEM
Method Info : test

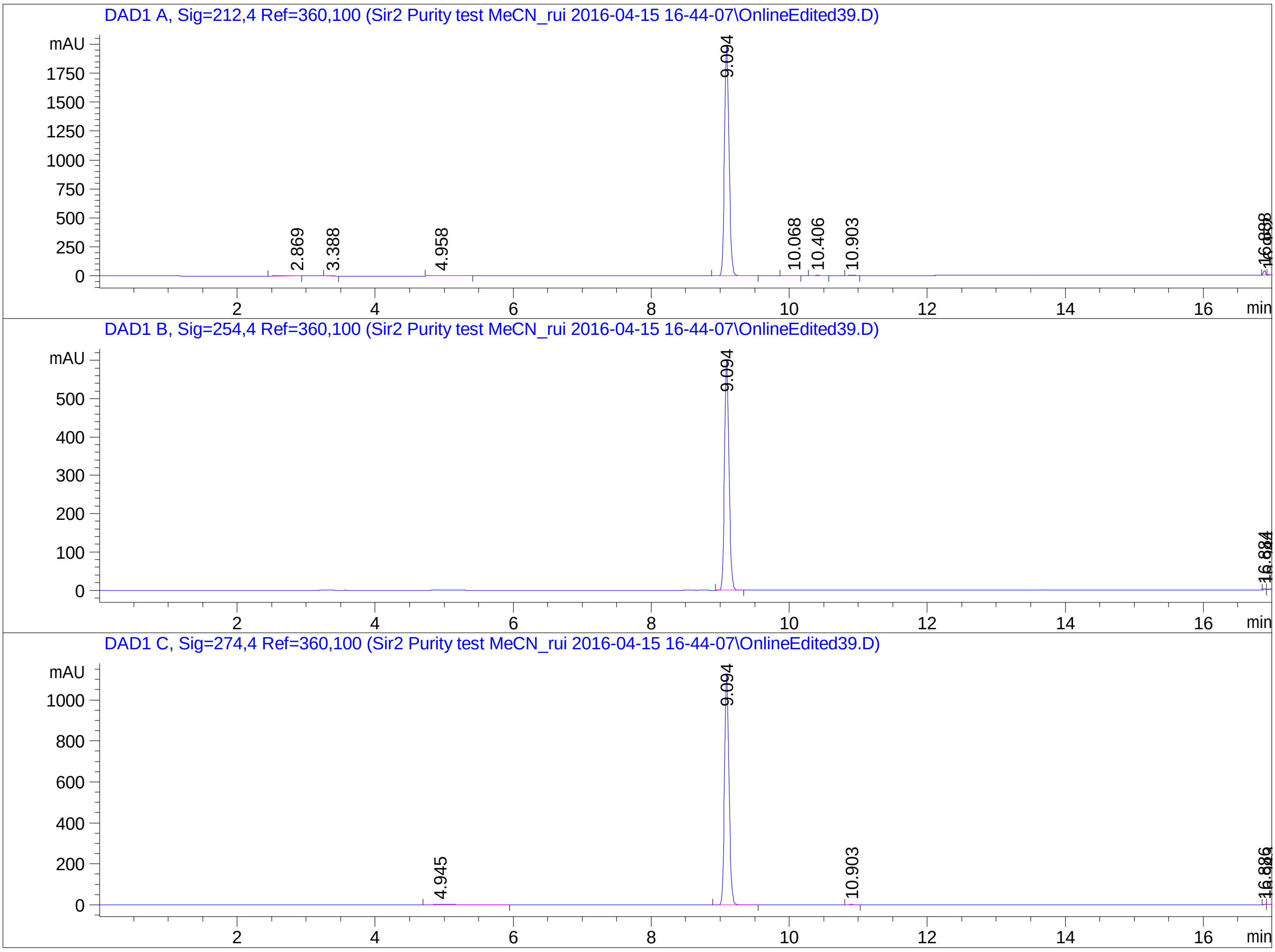

===

Area Percent Report

===

Sorted By : Signal
Multiplier : 1.0000
Dilution : 1.0000
Use Multiplier & Dilution Factor with ISTDs

Signal 1: DAD1 A, Sig=212,4 Ref=360,100

Peak #	RetTime [min]	Type	Width [min]	Area [mAU*s]	Height [mAU]	Area %
1	2.869	BV	0.1364	14.32934	1.41219	0.1530
2	3.388	BB	0.0854	13.45081	2.19805	0.1436
3	4.958	BB	0.2725	41.55598	2.14042	0.4437
4	9.094	BB	0.0716	9169.08691	1995.50012	97.9030
5	10.068	BB	0.0699	5.63814	1.21998	0.0602
6	10.406	BB	0.0733	6.83470	1.44200	0.0730
7	10.903	BB	0.0776	13.00874	2.63465	0.1389
8	16.888	BV R	0.0398	93.03489	39.07999	0.9934
9	16.952	VBAE	0.0431	8.54230	3.30054	0.0912

Totals : 9365.48181 2048.92794

Signal 2: DAD1 B, Sig=254,4 Ref=360,100

Peak #	RetTime [min]	Type	Width [min]	Area [mAU*s]	Height [mAU]	Area %
1	9.094	BB	0.0686	2721.73901	603.20520	99.5438
2	16.884	BV	0.0396	7.39990	3.12706	0.2706
3	16.944	VBA	0.0423	5.07339	1.83032	0.1856

Totals : 2734.21231 608.16258

Signal 3: DAD1 C, Sig=274,4 Ref=360,100

Peak #	RetTime [min]	Type	Width [min]	Area [mAU*s]	Height [mAU]	Area %
1	4.945	BB	0.3006	40.42931	1.91236	0.7837
2	9.094	BB	0.0687	5102.22266	1129.76135	98.8981
3	10.903	BB	0.0777	5.36385	1.08452	0.1040
4	16.886	BV	0.0409	5.80721	2.34419	0.1126
5	16.943	VBA	0.0438	5.24997	1.81025	0.1018

Totals : 5159.07299 1136.91268

==

*** End of Report ***

Data File C:\Chem32\1\Data\Sir2 Purity test MeCN_rui 2016-04-15 16-44-07\OnlineEdited40.D
Sample Name: LC0557

Compound 22a

===

Acq. Operator : SYSTEM   Seq. Line : 42
Acq. Instrument : CDD   Location : P1-E-06
Injection Date : 4/16/2016 5:00:39 AM   Inj : 1
Inj Volume : 10.000 µl
Method : C:\Chem32\1\Data\Sir2 Purity test MeCN_rui 2016-04-15 16-44-07\Sir2 purity_30-100%MeCN.M (Sequence Method)
Last changed : 4/15/2016 4:44:07 PM by SYSTEM
Method Info : test

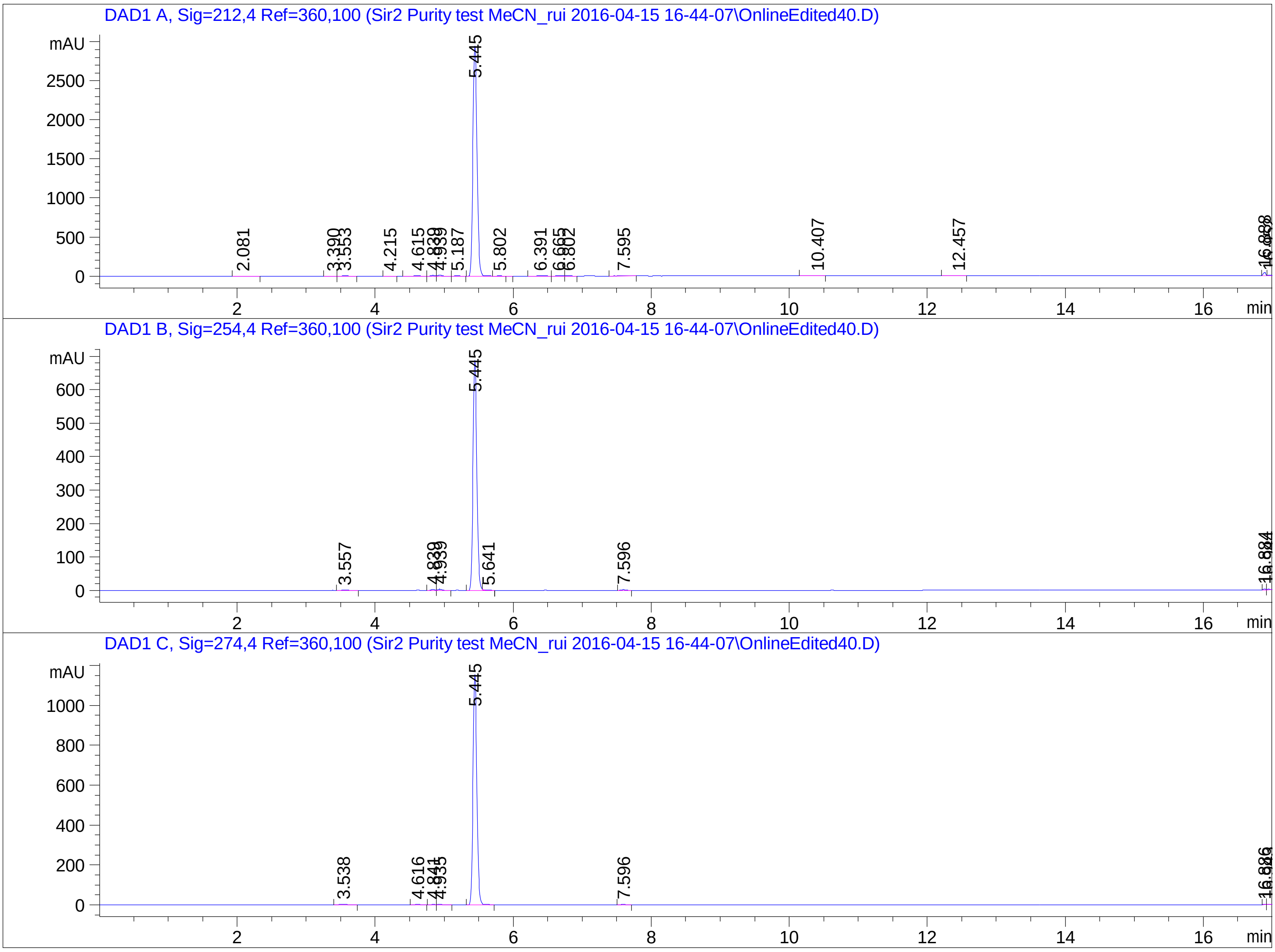

===

Area Percent Report

===

Sorted By : Signal
Multiplier : 1.0000
Dilution : 1.0000
Use Multiplier & Dilution Factor with ISTDs

Signal 1: DAD1 A, Sig=212,4 Ref=360,100

Peak #	RetTime [min]	Type	Width [min]	Area [mAU*s]	Height [mAU]	Area %
1	2.081	BB	0.2107	18.63899	1.17749	0.1469
2	3.390	BB	0.0695	6.96049	1.46227	0.0549
3	3.553	BB	0.0985	26.55713	3.75329	0.2093
4	4.215	BB	0.0832	9.00953	1.71706	0.0710
5	4.615	VB R	0.0783	35.13869	6.37819	0.2769
6	4.839	BV	0.0585	36.77551	9.64666	0.2898
7	4.939	VB	0.0639	53.78519	12.57866	0.4239
8	5.187	BB	0.0623	29.27809	7.37749	0.2307
9	5.445	BV R	0.0665	1.22702e4	2950.33887	96.7052
10	5.802	VV E	0.0846	9.39793	1.64654	0.0741
11	6.391	BV	0.0800	23.53324	4.28727	0.1855
12	6.665	VV	0.1029	8.64895	1.34788	0.0682
13	6.802	VB	0.0720	6.15820	1.28264	0.0485
14	7.595	BB	0.0792	34.23397	6.74761	0.2698
15	10.407	BV	0.0794	7.75513	1.47378	0.0611
16	12.457	BB	0.1073	8.57940	1.12066	0.0676
17	16.888	BV R	0.0397	96.00304	40.37093	0.7566
18	16.952	VBAE	0.0429	7.60337	2.95185	0.0599

Totals : 1.26883e4 3055.65915

Signal 2: DAD1 B, Sig=254,4 Ref=360,100

Peak #	RetTime [min]	Type	Width [min]	Area [mAU*s]	Height [mAU]	Area %
1	3.557	BB	0.0938	7.10773	1.06640	0.2616
2	4.839	BV	0.0591	12.27004	3.17701	0.4516
3	4.939	VB	0.0652	15.84724	3.75558	0.5832
4	5.445	BV R	0.0589	2653.37476	688.85657	97.6503
5	5.641	VB E	0.0689	5.96602	1.26890	0.2196
6	7.596	BB	0.0729	9.77822	2.07857	0.3599
7	16.884	BV	0.0397	7.67684	3.22997	0.2825
8	16.944	VBA	0.0423	5.20053	1.87843	0.1914

Totals : 2717.22138 705.31143

Signal 3: DAD1 C, Sig=274,4 Ref=360,100

Peak #	RetTime [min]	Type	Width [min]	Area [mAU*s]	Height [mAU]	Area %
1	3.538	BB	0.1142	17.50183	2.16856	0.3872
2	4.616	BB	0.0652	12.06503	2.75044	0.2669
3	4.841	BV	0.0591	5.55454	1.43643	0.1229
4	4.935	VB	0.0699	9.14992	1.90973	0.2024
5	5.445	BV R	0.0590	4457.62793	1155.63184	98.6066

Peak #	RetTime [min]	Type	Width [min]	Area [mAU*s]	Height [mAU]	Area %
6	7.596	BB	0.0737	7.18279	1.50464	0.1589
7	16.886	BV	0.0410	6.19305	2.48742	0.1370
8	16.943	VBA	0.0436	5.34298	1.85592	0.1182

Totals : 4520.61807 1169.74497

==

*** End of Report ***

Data File C:\Chem32\1\Data\Sir2 Purity test MeCN_rui 2016-04-15 16-44-07\OnlineEdited41.D
Sample Name: LC0537

Compound 22b

===

Acq. Operator : SYSTEM Seq. Line : 43
Acq. Instrument : CDD Location : P1-E-07
Injection Date : 4/16/2016 5:18:37 AM Inj : 1
Inj Volume : 10.000 µl
Method : C:\Chem32\1\Data\Sir2 Purity test MeCN_rui 2016-04-15 16-44-07\Sir2 purity_30-100%MeCN.M (Sequence Method)
Last changed : 4/15/2016 4:44:07 PM by SYSTEM
Method Info : test

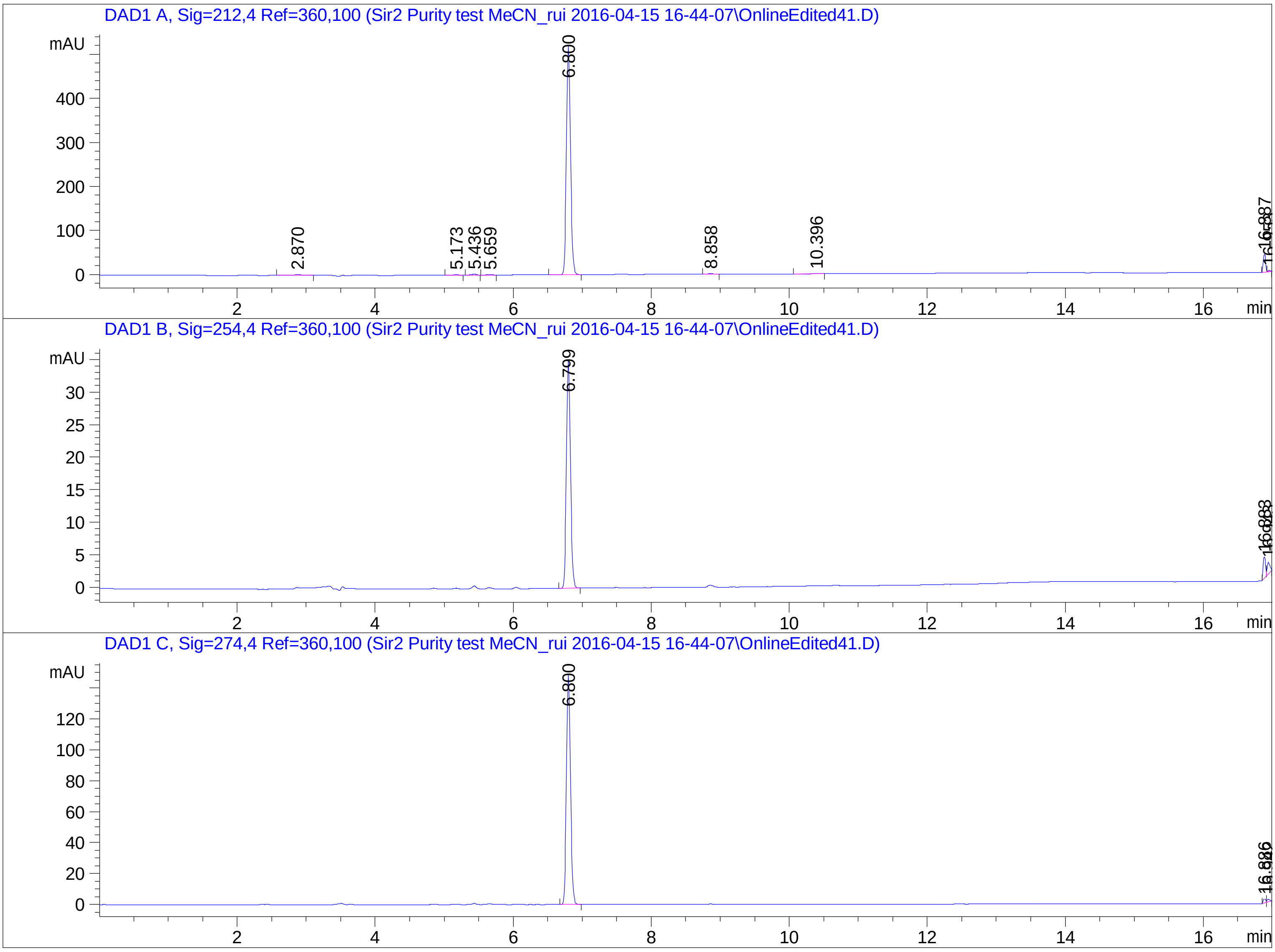

===

Area Percent Report

===

Sorted By : Signal
Multiplier : 1.0000
Dilution : 1.0000
Use Multiplier & Dilution Factor with ISTDs

Signal 1: DAD1 A, Sig=212,4 Ref=360,100

Peak #	RetTime [min]	Type	Width [min]	Area [mAU*s]	Height [mAU]	Area %
1	2.870	BB	0.1529	12.29305	1.04776	0.5300
2	5.173	BB	0.0687	5.20754	1.15230	0.2245
3	5.436	BB	0.0670	11.32366	2.49043	0.4882
4	5.659	BB	0.0670	6.06941	1.38963	0.2617
5	6.800	BB	0.0645	2168.57202	521.86249	93.4907
6	8.858	BB	0.0821	7.22017	1.40258	0.3113
7	10.396	BB	0.0749	5.05013	1.03606	0.2177
8	16.887	BV R	0.0397	96.88270	40.85967	4.1768
9	16.953	VBAE	0.0434	6.93944	2.66257	0.2992

Totals : 2319.55813 573.90349

Signal 2: DAD1 B, Sig=254,4 Ref=360,100

Peak #	RetTime [min]	Type	Width [min]	Area [mAU*s]	Height [mAU]	Area %
1	6.799	BB	0.0642	145.36461	35.20002	91.7995
2	16.883	BV	0.0396	7.82808	3.30787	4.9435
3	16.943	VBA	0.0425	5.15743	1.85331	3.2570

Totals : 158.35012 40.36121

Signal 3: DAD1 C, Sig=274,4 Ref=360,100

Peak #	RetTime [min]	Type	Width [min]	Area [mAU*s]	Height [mAU]	Area %
1	6.800	BB	0.0643	618.88312	149.54994	98.1144
2	16.886	BV	0.0411	6.52207	2.61409	1.0340
3	16.942	VBA	0.0439	5.37177	1.84815	0.8516

Totals : 630.77696 154.01217

===

*** End of Report ***

Data File C:\Chem32\1\Data\Sir2 Purity test MeCN_rui 2016-04-15 16-44-07\OnlineEdited42.D
Sample Name: LC0534

Compound 22c

===

Acq. Operator	: SYSTEM		Seq. Line :	44
Acq. Instrument	: CDD		Location :	P1-E-08
Injection Date	: 4/16/2016 5:36:33 AM		Inj :	1
			Inj Volume :	10.000 µl

Method : C:\Chem32\1\Data\Sir2 Purity test MeCN_rui 2016-04-15 16-44-07\Sir2 purity_30-100%MeCN.M (Sequence Method)
Last changed : 4/15/2016 4:44:07 PM by SYSTEM
Method Info : test

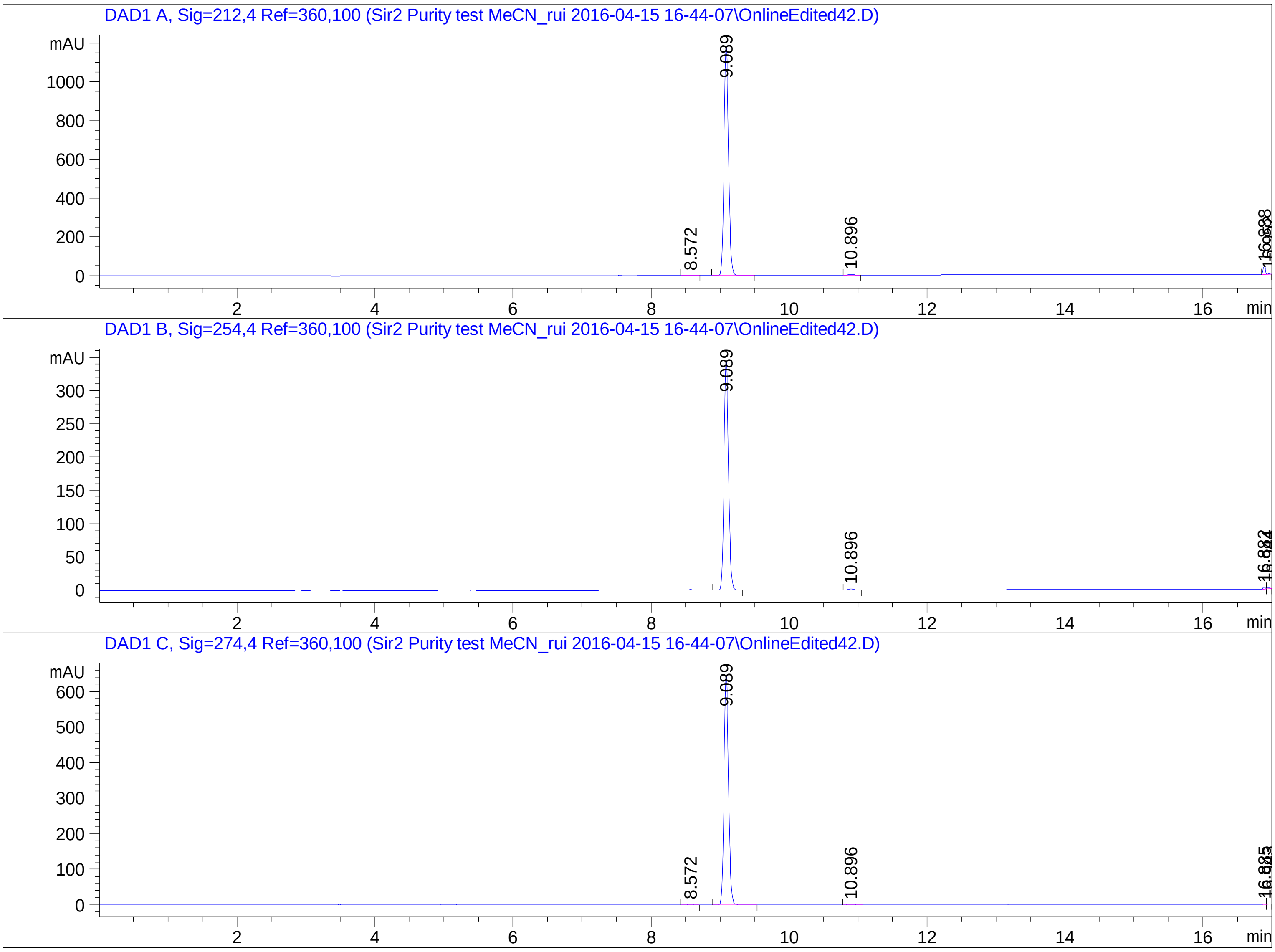

===

Area Percent Report

===

Sorted By : Signal
Multiplier : 1.0000
Dilution : 1.0000
Use Multiplier & Dilution Factor with ISTDs

Signal 1: DAD1 A, Sig=212,4 Ref=360,100

Peak #	RetTime [min]	Type	Width [min]	Area [mAU*s]	Height [mAU]	Area %
1	8.572	BB	0.0683	8.76043	1.95366	0.1595
2	9.089	BB	0.0707	5364.50439	1187.35706	97.6449
3	10.896	BB	0.0789	19.68862	3.89664	0.3584
4	16.888	BV R	0.0397	94.65457	39.87001	1.7229
5	16.952	VBAE	0.0401	6.28480	2.43528	0.1144
Totals :				5493.89281	1235.51264	

Signal 2: DAD1 B, Sig=254,4 Ref=360,100

Peak #	RetTime [min]	Type	Width [min]	Area [mAU*s]	Height [mAU]	Area %
1	9.089	BB	0.0683	1554.30615	346.53513	98.7372
2	10.896	BB	0.0782	7.43809	1.49114	0.4725
3	16.882	BV	0.0364	7.31227	3.23424	0.4645
4	16.944	VBA	0.0427	5.12803	1.83168	0.3258
Totals :				1574.18454	353.09219	

Signal 3: DAD1 C, Sig=274,4 Ref=360,100

Peak #	RetTime [min]	Type	Width [min]	Area [mAU*s]	Height [mAU]	Area %
1	8.572	BB	0.0668	8.24654	1.89257	0.2802
2	9.089	BB	0.0684	2914.98340	649.47290	99.0565
3	10.896	BB	0.0792	8.10738	1.59682	0.2755
4	16.885	BV	0.0408	6.10097	2.46982	0.2073
5	16.943	VBA	0.0439	5.30889	1.82491	0.1804
Totals :				2942.74717	657.25702	

===
*** End of Report ***

Data File C:\Chem32\1\Data\RD SIRT2 2016-07-20 16-41-09\OnlineEdited81.D
Sample Name: LC-0420 **Compound 15a**

===

Acq. Operator : SYSTEM Seq. Line : 13
Acq. Instrument : CDD Location : P1-B-05
Injection Date : 7/20/2016 9:16:08 PM Inj : 1
Inj Volume : 5.000 µl
Different Inj Volume from Sample Entry! Actual Inj Volume : 10.000 µl
Method : C:\Chem32\1\Data\RD SIRT2 2016-07-20 16-41-09\RD VCP Methanol 40-100.M (Sequence Method)
Last changed : 7/20/2016 4:41:10 PM by SYSTEM
Method Info : test

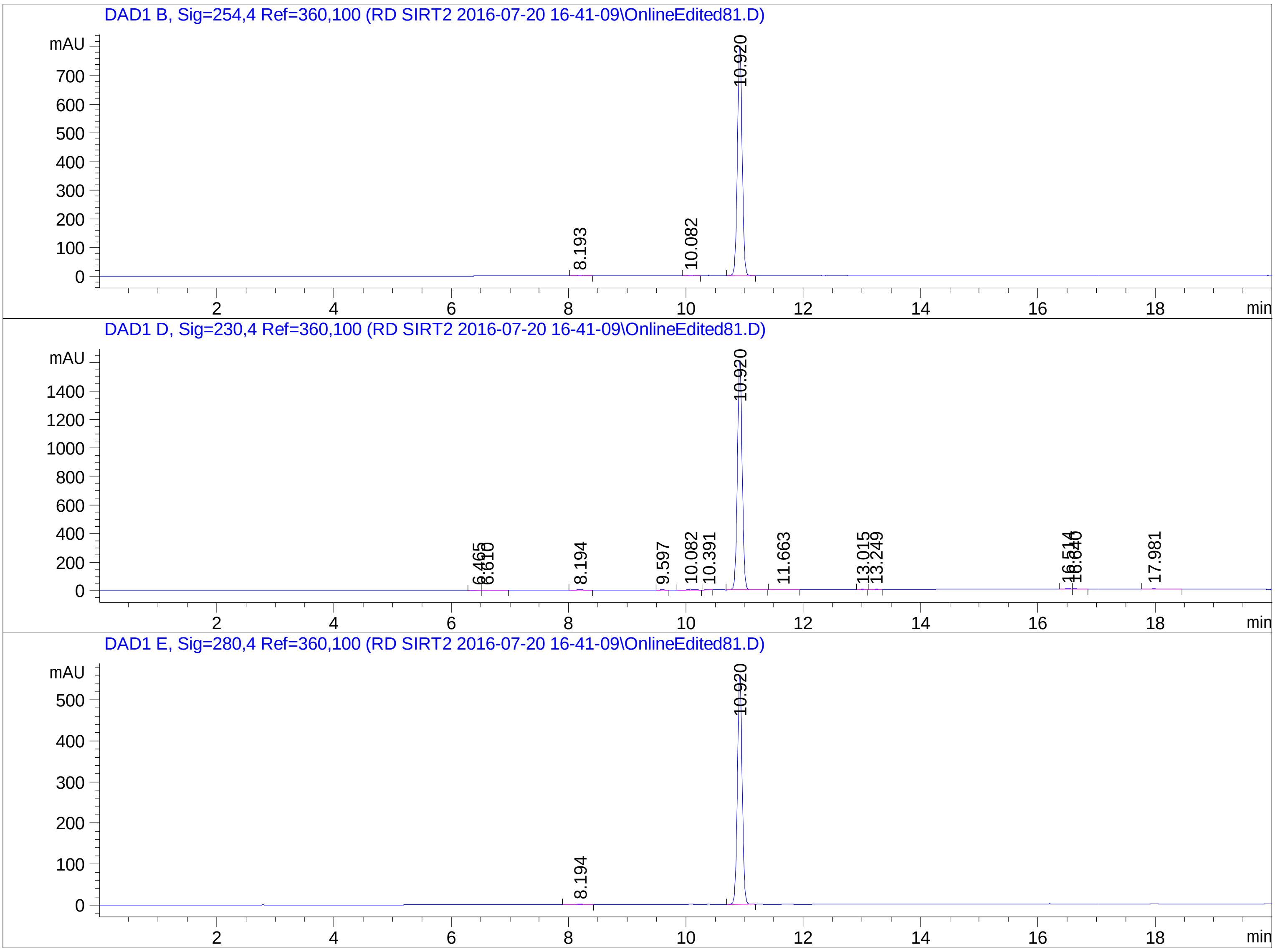

===

Area Percent Report

===

Sorted By : Signal
Multiplier : 1.0000
Dilution : 1.0000
Use Multiplier & Dilution Factor with ISTDs

Signal 1: DAD1 B, Sig=254,4 Ref=360,100

Peak #	RetTime [min]	Type	Width [min]	Area [mAU*s]	Height [mAU]	Area %
1	8.193	BB	0.1333	16.45846	1.93399	0.3623
2	10.082	BB	0.0909	12.70294	2.14944	0.2796
3	10.920	BB	0.0876	4513.63623	802.68030	99.3581

Totals : 4542.79764 806.76373

Signal 2: DAD1 D, Sig=230,4 Ref=360,100

Peak #	RetTime [min]	Type	Width [min]	Area [mAU*s]	Height [mAU]	Area %
1	6.465	BV	0.1063	11.40138	1.61681	0.1227
2	6.610	VB	0.1375	25.43890	2.61543	0.2737
3	8.194	BB	0.1337	33.99773	3.97887	0.3658
4	9.597	BB	0.0800	7.47443	1.45241	0.0804
5	10.082	BB	0.0949	27.36769	4.37919	0.2944
6	10.391	BV	0.0794	10.41096	1.97964	0.1120
7	10.920	BV R	0.0879	9099.55176	1611.47351	97.9020
8	11.663	BB	0.1141	14.82964	1.83820	0.1596
9	13.015	BB	0.0813	8.72295	1.71700	0.0939
10	13.249	BB	0.0806	6.95167	1.33881	0.0748
11	16.514	BV	0.0917	17.31413	2.81722	0.1863
12	16.640	VV	0.0987	14.22445	2.05569	0.1530
13	17.981	BB	0.1122	16.86211	2.18123	0.1814

Totals : 9294.54777 1639.44400

Signal 3: DAD1 E, Sig=280,4 Ref=360,100

Peak #	RetTime [min]	Type	Width [min]	Area [mAU*s]	Height [mAU]	Area %
1	8.194	BB	0.1380	10.83641	1.21591	0.3433
2	10.920	BB	0.0876	3145.68262	559.62396	99.6567

Totals : 3156.51903 560.83987

===

*** End of Report ***

Data File C:\Chem32\1\Data\RD SIRT2 2016-07-20 16-41-09\OnlineEdited85.D
Sample Name: LC-0511

Compound 15b

===

Acq. Operator : SYSTEM Seq. Line : 17
Acq. Instrument : CDD Location : P1-B-09
Injection Date : 7/20/2016 10:47:32 PM Inj : 1
Inj Volume : 5.000 µl
Different Inj Volume from Sample Entry! Actual Inj Volume : 10.000 µl
Method : C:\Chem32\1\Data\RD SIRT2 2016-07-20 16-41-09\RD VCP Methanol 40-100.M (Sequence Method)
Last changed : 7/20/2016 4:41:10 PM by SYSTEM
Method Info : test

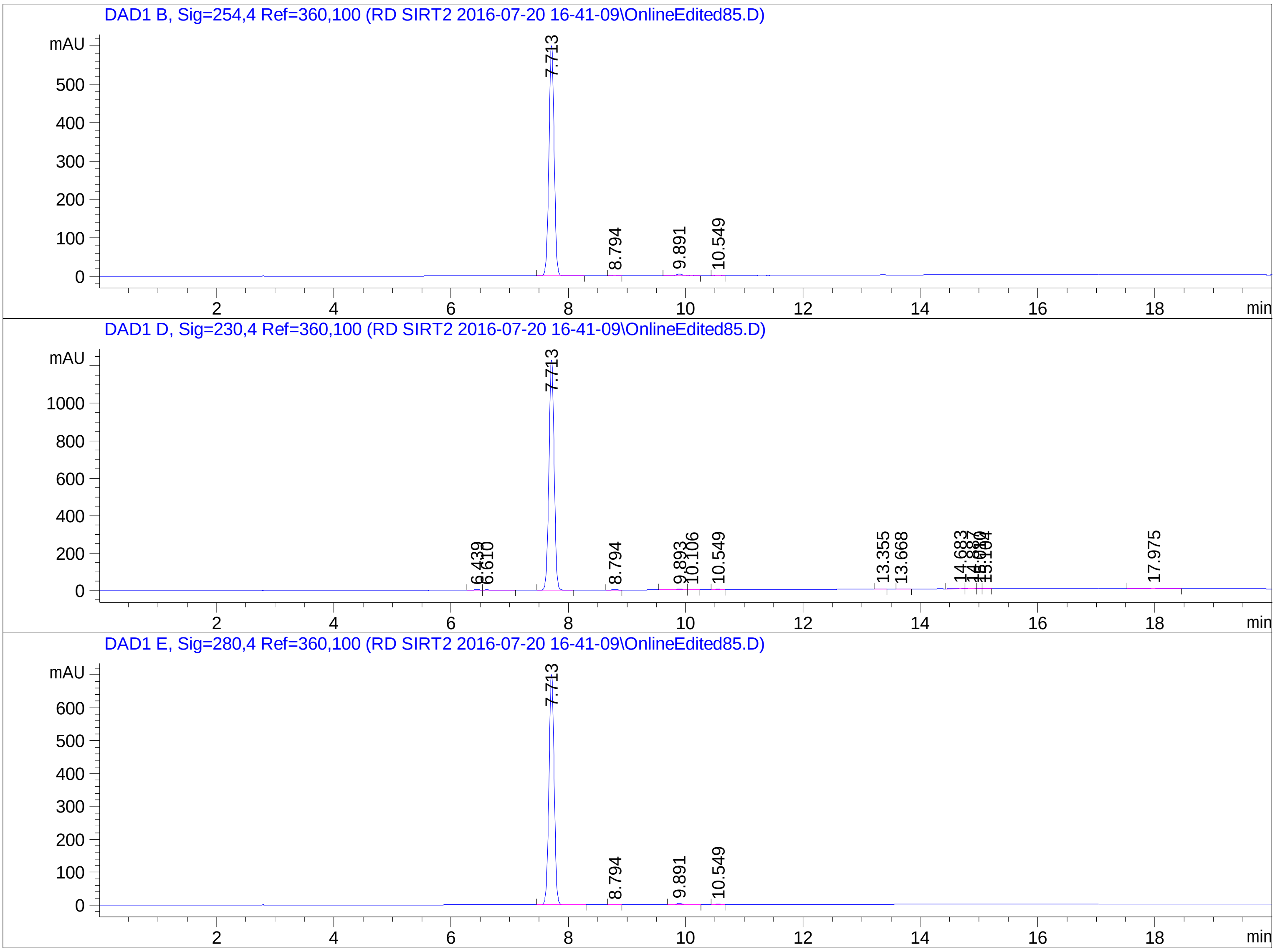

===

Area Percent Report

===

Sorted By : Signal
Multiplier : 1.0000
Dilution : 1.0000
Use Multiplier & Dilution Factor with ISTDs

Signal 1: DAD1 B, Sig=254,4 Ref=360,100

Peak #	RetTime [min]	Type	Width [min]	Area [mAU*s]	Height [mAU]	Area %
1	7.713	BB	0.0954	3672.45776	600.28931	98.8033
2	8.794	BB	0.0924	6.48451	1.10619	0.1745
3	9.891	BV R	0.1081	31.98347	4.33566	0.8605
4	10.549	BB	0.0859	6.01273	1.09859	0.1618

Totals : 3716.93847 606.82975

Signal 2: DAD1 D, Sig=230,4 Ref=360,100

Peak #	RetTime [min]	Type	Width [min]	Area [mAU*s]	Height [mAU]	Area %
1	6.439	BV	0.1095	29.11071	4.06732	0.3742
2	6.610	VB	0.1483	28.07322	2.67843	0.3608
3	7.713	BB	0.0956	7540.19092	1229.24573	96.9120
4	8.794	BB	0.0916	12.91389	2.22866	0.1660
5	9.893	BV	0.1100	31.86764	4.22698	0.4096
6	10.106	VB	0.0950	12.03650	1.92238	0.1547
7	10.549	BB	0.0856	13.79663	2.52960	0.1773
8	13.355	BB	0.0949	8.88917	1.50563	0.1143
9	13.668	BB	0.0807	6.69069	1.28567	0.0860
10	14.683	VV R	0.0844	23.69258	4.16170	0.3045
11	14.887	VV	0.1025	38.59135	5.08497	0.4960
12	15.010	VV	0.0632	7.26466	1.72143	0.0934
13	15.104	VB	0.0695	9.19046	2.00427	0.1181
14	17.975	BB	0.1179	18.14151	2.25320	0.2332

Totals : 7780.44993 1264.91597

Signal 3: DAD1 E, Sig=280,4 Ref=360,100

Peak #	RetTime [min]	Type	Width [min]	Area [mAU*s]	Height [mAU]	Area %
1	7.713	BB	0.0953	4285.66016	700.76459	99.0057
2	8.794	BB	0.0919	6.03008	1.03662	0.1393
3	9.891	BV R	0.1115	30.03063	3.91259	0.6938
4	10.549	BB	0.0853	6.97943	1.28651	0.1612

Totals : 4328.70030 707.00031

==

*** End of Report ***

Data File C:\Chem32\1\Data\RD SIRT2 2016-07-21 10-43-19\1FB-0201.D
Sample Name: LC-0424

Compound 16a

===

Acq. Operator   : SYSTEM   Seq. Line :   2
Acq. Instrument : CDD   Location :   P1-F-02
Injection Date : 7/21/2016 11:07:02 AM   Inj :   1
                                                Inj Volume : 5.000 µl
Different Inj Volume from Sample Entry! Actual Inj Volume : 10.000 µl
Method   : C:\Chem32\1\Data\RD SIRT2 2016-07-21 10-43-19\RD VCP Methanol 40-100.M (
                  Sequence Method)
Last changed   : 7/21/2016 10:43:19 AM by SYSTEM
Method Info   : test

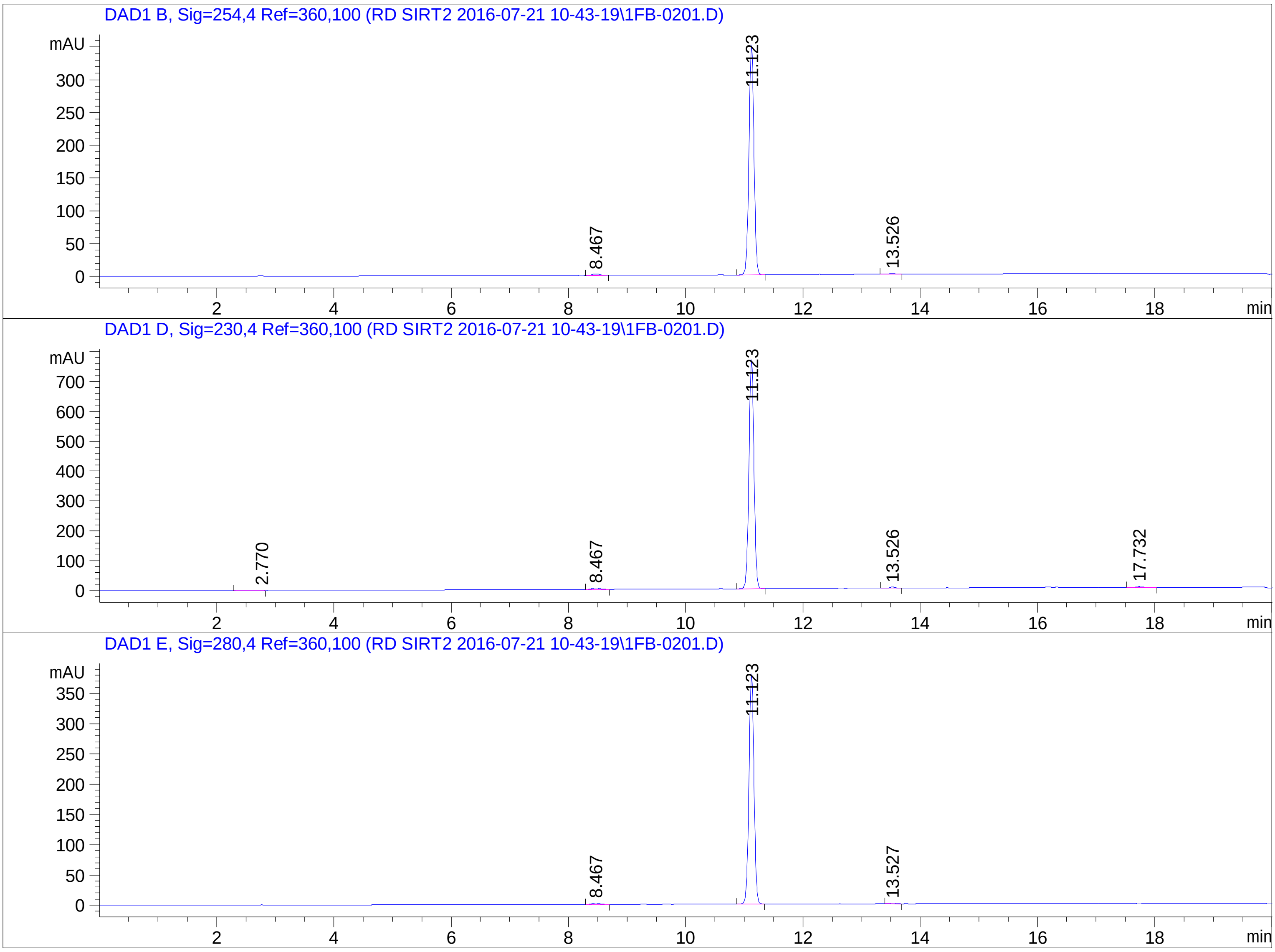

===
                         Area Percent Report
===

Sorted By   :   Signal
Multiplier   :   1.0000
Dilution   :   1.0000
Use Multiplier & Dilution Factor with ISTDs

Signal 1: DAD1 B, Sig=254,4 Ref=360,100

Peak #	RetTime [min]	Type	Width [min]	Area [mAU*s]	Height [mAU]	Area %
1	8.467	BB	0.1404	21.30717	2.38215	1.0071
2	11.123	BB	0.0935	2085.70117	350.20624	98.5832
3	13.526	BB	0.0959	8.66756	1.36777	0.4097

Totals : 2115.67590 353.95615

Signal 2: DAD1 D, Sig=230,4 Ref=360,100

Peak #	RetTime [min]	Type	Width [min]	Area [mAU*s]	Height [mAU]	Area %
1	2.770	BB	0.1110	9.60093	1.15483	0.2061
2	8.467	BB	0.1406	43.64967	4.86856	0.9371
3	11.123	BB	0.0936	4565.21387	765.50922	98.0070
4	13.526	BB	0.0971	23.59867	3.66372	0.5066
5	17.732	BB	0.1034	15.98425	2.34906	0.3432

Totals : 4658.04739 777.54538

Signal 3: DAD1 E, Sig=280,4 Ref=360,100

Peak #	RetTime [min]	Type	Width [min]	Area [mAU*s]	Height [mAU]	Area %
1	8.467	BB	0.1395	21.33203	2.35860	0.9319
2	11.123	BB	0.0935	2259.36499	379.45120	98.6963
3	13.527	BB	0.0902	8.51135	1.45588	0.3718

Totals : 2289.20837 383.26569

===

*** End of Report ***

Data File C:\Chem32\1\Data\RD SIRT2 2016-07-20 16-41-09\OnlineEdited91.D
Sample Name: LC-0524

Compound 16b

===

Acq. Operator : SYSTEM   Seq. Line : 23
Acq. Instrument : CDD   Location : P1-C-06
Injection Date : 7/21/2016 1:04:34 AM   Inj : 1
                    Inj Volume : 5.000 µl
Different Inj Volume from Sample Entry! Actual Inj Volume : 10.000 µl
Method : C:\Chem32\1\Data\RD SIRT2 2016-07-20 16-41-09\RD VCP Methanol 40-100.M (Sequence Method)
Last changed : 7/20/2016 4:41:10 PM by SYSTEM
Method Info : test

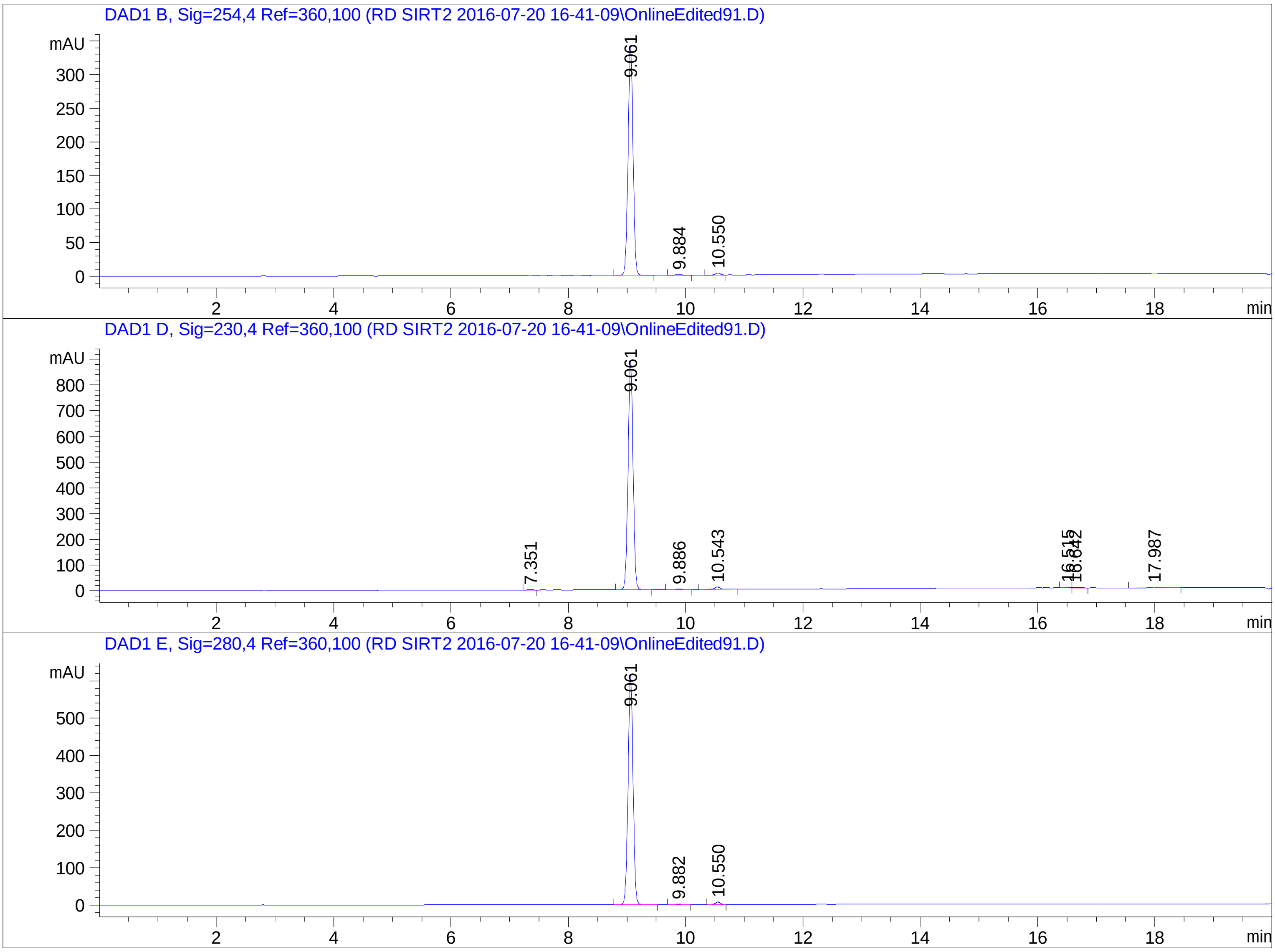

===

Area Percent Report

===

Sorted By : Signal
Multiplier : 1.0000
Dilution : 1.0000
Use Multiplier & Dilution Factor with ISTDs

Signal 1: DAD1 B, Sig=254,4 Ref=360,100

Peak #	RetTime [min]	Type	Width [min]	Area [mAU*s]	Height [mAU]	Area %
1	9.061	BB	0.0924	2008.87170	342.66144	98.8092
2	9.884	BB	0.1035	8.07362	1.15530	0.3971
3	10.550	BB	0.0893	16.13533	2.79606	0.7936
Totals :				2033.08065	346.61279	

Signal 2: DAD1 D, Sig=230,4 Ref=360,100

Peak #	RetTime [min]	Type	Width [min]	Area [mAU*s]	Height [mAU]	Area %
1	7.351	BV	0.0944	8.76639	1.45276	0.1630
2	9.061	BB	0.0925	5254.30908	894.71814	97.7054
3	9.886	BB	0.1074	8.46663	1.13033	0.1574
4	10.543	BV R	0.1053	64.34174	9.23222	1.1965
5	16.515	BV	0.0940	13.16004	2.13342	0.2447
6	16.642	VV	0.1017	10.83476	1.54647	0.2015
7	17.987	BB	0.1142	17.82999	2.25551	0.3316
Totals :				5377.70863	912.46885	

Signal 3: DAD1 E, Sig=280,4 Ref=360,100

Peak #	RetTime [min]	Type	Width [min]	Area [mAU*s]	Height [mAU]	Area %
1	9.061	BB	0.0924	3614.99927	616.68585	98.7346
2	9.882	BB	0.1117	8.33632	1.08426	0.2277
3	10.550	BB	0.0892	37.99265	6.59852	1.0377
Totals :				3661.32824	624.36863	

==

*** End of Report ***

Compound 17

===

Acq. Operator : SYSTEM Seq. Line : 25
Acq. Instrument : CDD Location : P1-C-08
Injection Date : 7/21/2016 1:50:16 AM Inj : 1
Inj Volume : 5.000 µl
Different Inj Volume from Sample Entry! Actual Inj Volume : 10.000 µl
Method : C:\Chem32\1\Data\RD SIRT2 2016-07-20 16-41-09\RD VCP Methanol 40-100.M (Sequence Method)
Last changed : 7/20/2016 4:41:10 PM by SYSTEM
Method Info : test

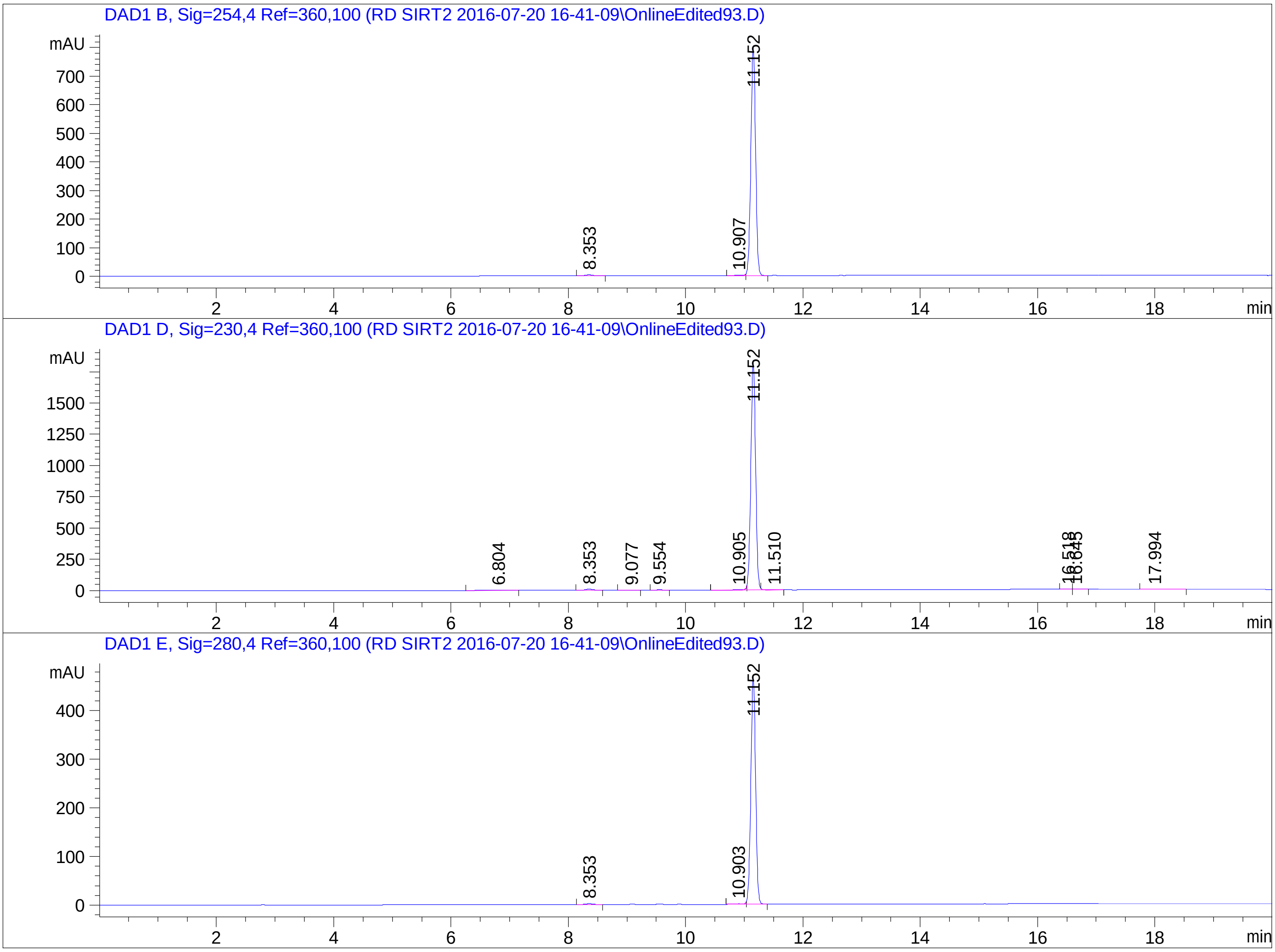

===

Area Percent Report

===

Sorted By : Signal
Multiplier : 1.0000
Dilution : 1.0000
Use Multiplier & Dilution Factor with ISTDs

Signal 1: DAD1 B, Sig=254,4 Ref=360,100

Peak #	RetTime [min]	Type	Width [min]	Area [mAU*s]	Height [mAU]	Area %
1	8.353	BB	0.1447	35.86850	3.84946	0.7873
2	10.907	BV E	0.1228	15.00904	1.80899	0.3294
3	11.152	VB R	0.0874	4504.97217	804.01068	98.8832

Totals : 4555.84971 809.66913

Signal 2: DAD1 D, Sig=230,4 Ref=360,100

Peak #	RetTime [min]	Type	Width [min]	Area [mAU*s]	Height [mAU]	Area %
1	6.804	BB	0.3358	28.12843	1.05258	0.2665
2	8.353	BB	0.1461	82.78275	8.93699	0.7843
3	9.077	BB	0.1077	13.94524	1.94402	0.1321
4	9.554	BB	0.1123	15.12032	2.09304	0.1432
5	10.905	BV E	0.1435	32.72190	3.30415	0.3100
6	11.152	VV R	0.0876	1.03274e4	1836.41357	97.8390
7	11.510	VB E	0.1344	16.50437	1.74417	0.1564
8	16.518	BV	0.0942	11.89883	1.92251	0.1127
9	16.645	VV	0.0980	9.33234	1.36126	0.0884
10	17.994	BB	0.1152	17.67356	2.26096	0.1674

Totals : 1.05555e4 1861.03326

Signal 3: DAD1 E, Sig=280,4 Ref=360,100

Peak #	RetTime [min]	Type	Width [min]	Area [mAU*s]	Height [mAU]	Area %
1	8.353	BB	0.1446	17.53759	1.88356	0.6567
2	10.903	BV E	0.1290	8.74528	1.01026	0.3275
3	11.152	VB R	0.0873	2644.43335	472.40259	99.0159

Totals : 2670.71623 475.29641

==

*** End of Report ***

Compound 18

===

Acq. Operator : SYSTEM   Seq. Line : 29
Acq. Instrument : CDD   Location : P1-D-03
Injection Date : 7/21/2016 3:21:37 AM   Inj : 1
Inj Volume : 5.000 µl
Different Inj Volume from Sample Entry! Actual Inj Volume : 10.000 µl
Method : C:\Chem32\1\Data\RD SIRT2 2016-07-20 16-41-09\RD VCP Methanol 40-100.M (Sequence Method)
Last changed : 7/20/2016 4:41:10 PM by SYSTEM
Method Info : test

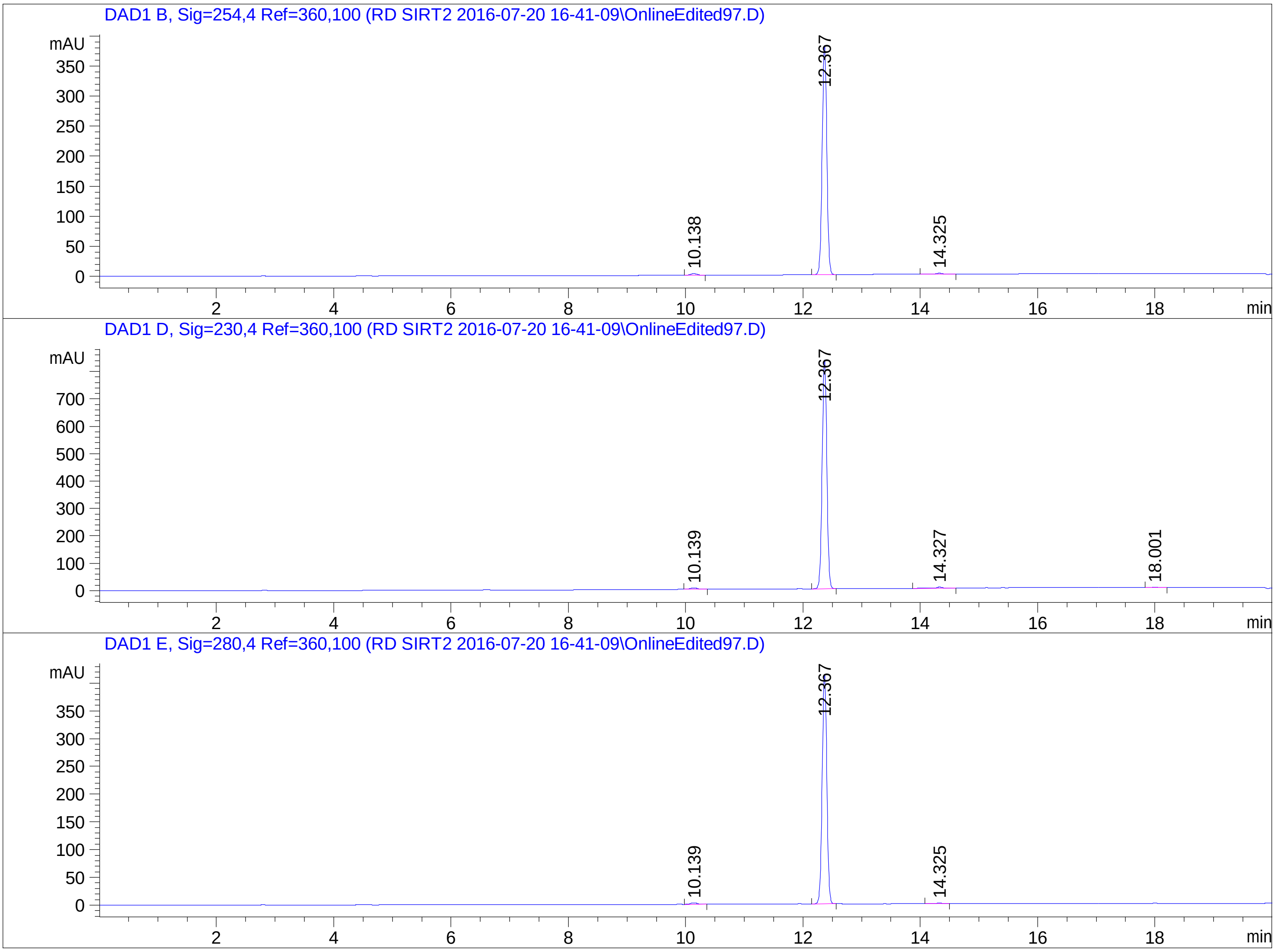

===

Area Percent Report

===

Sorted By : Signal
Multiplier : 1.0000
Dilution : 1.0000
Use Multiplier & Dilution Factor with ISTDs

Signal 1: DAD1 B, Sig=254,4 Ref=360,100

Peak #	RetTime [min]	Type	Width [min]	Area [mAU*s]	Height [mAU]	Area %
1	10.138	BB	0.1217	21.87729	2.78457	1.0299
2	12.367	BB	0.0837	2086.56006	382.41519	98.2250
3	14.325	BB	0.1275	15.82832	1.71855	0.7451

Totals : 2124.26567 386.91832

Signal 2: DAD1 D, Sig=230,4 Ref=360,100

Peak #	RetTime [min]	Type	Width [min]	Area [mAU*s]	Height [mAU]	Area %
1	10.139	BB	0.1231	44.62066	5.59220	0.9571
2	12.367	BB	0.0837	4571.55225	836.88788	98.0604
3	14.327	BV R	0.1101	32.14836	4.16234	0.6896
4	18.001	BB	0.0993	13.65461	2.11682	0.2929

Totals : 4661.97588 848.75923

Signal 3: DAD1 E, Sig=280,4 Ref=360,100

Peak #	RetTime [min]	Type	Width [min]	Area [mAU*s]	Height [mAU]	Area %
1	10.139	BB	0.1248	21.48736	2.70238	0.9374
2	12.367	BB	0.0836	2260.22705	414.37790	98.6012
3	14.325	BB	0.0971	10.57811	1.64299	0.4615

Totals : 2292.29252 418.72327

===

*** End of Report ***

Data File C:\Chem32\1\Data\RD SIRT2 2016-07-20 16-41-09\OnlineEdited103.D
Sample Name: LC-0533

Compound 19a

===

Acq. Operator : SYSTEM Seq. Line : 33
Acq. Instrument : CDD Location : P1-D-07
Injection Date : 7/21/2016 4:52:58 AM Inj : 1
Inj Volume : 5.000 µl
Different Inj Volume from Sample Entry! Actual Inj Volume : 10.000 µl
Method : C:\Chem32\1\Data\RD SIRT2 2016-07-20 16-41-09\RD VCP Methanol 40-100.M (Sequence Method)
Last changed : 7/20/2016 4:41:10 PM by SYSTEM
Method Info : test

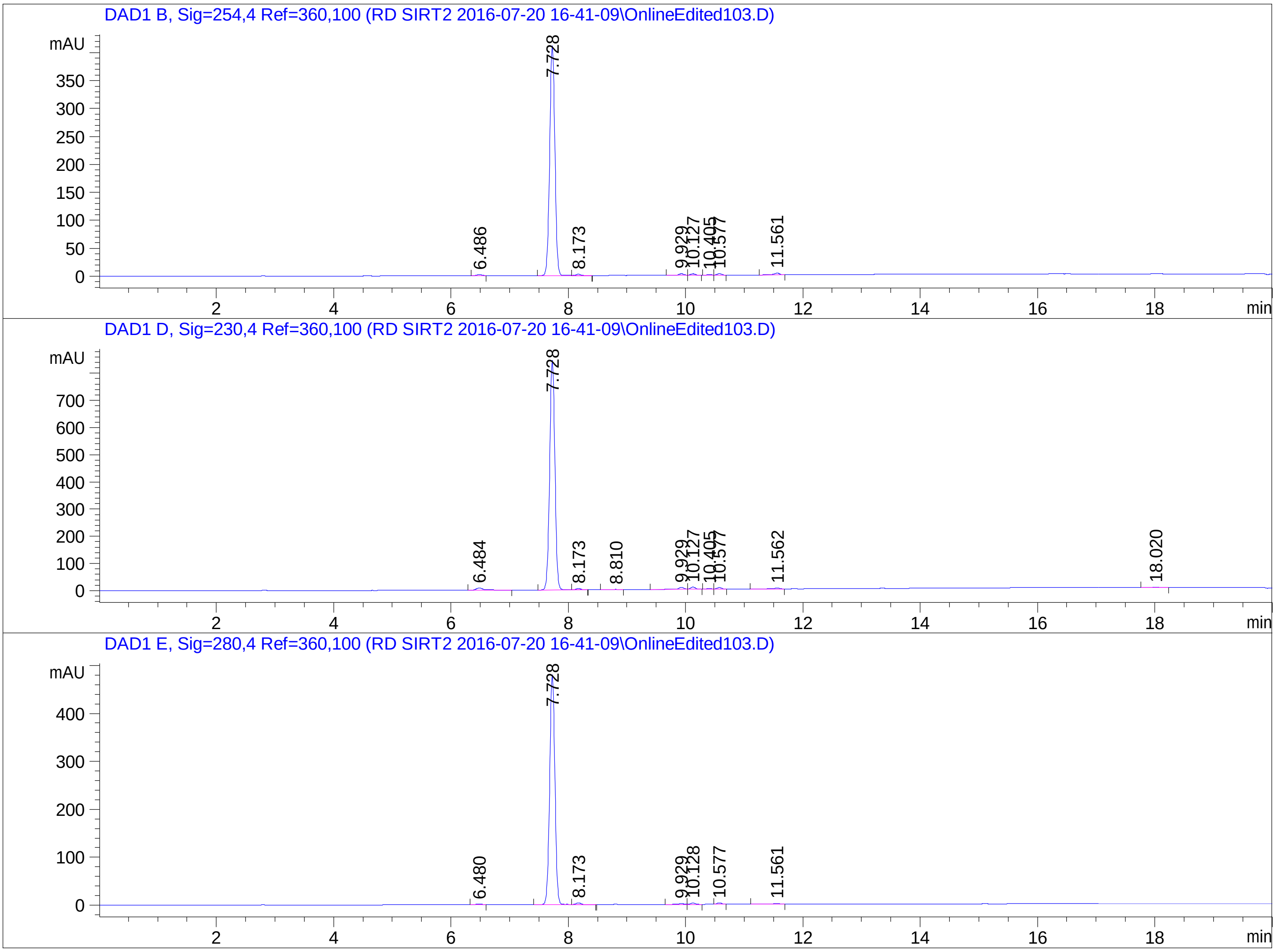

===

Area Percent Report

===

Sorted By : Signal
Multiplier : 1.0000
Dilution : 1.0000
Use Multiplier & Dilution Factor with ISTDs

Signal 1: DAD1 B, Sig=254,4 Ref=360,100

Peak #	RetTime [min]	Type	Width [min]	Area [mAU*s]	Height [mAU]	Area %
1	6.486	BB	0.1024	11.99584	1.88080	0.4556
2	7.728	BV R	0.0957	2528.10669	411.52805	96.0264
3	8.173	VB E	0.0903	13.36069	2.28385	0.5075
4	9.929	BV	0.1004	18.06580	2.75793	0.6862
5	10.127	VB	0.0894	14.78776	2.56105	0.5617
6	10.405	BV	0.0903	6.68160	1.17548	0.2538
7	10.577	VB	0.0899	15.07248	2.66767	0.5725
8	11.561	BB	0.1122	24.64992	3.26016	0.9363
Totals :				2632.72078	428.11499	

Signal 2: DAD1 D, Sig=230,4 Ref=360,100

Peak #	RetTime [min]	Type	Width [min]	Area [mAU*s]	Height [mAU]	Area %
1	6.484	BB	0.1388	79.07143	8.32814	1.4327
2	7.728	BV R	0.0958	5206.85645	845.69189	94.3404
3	8.173	VB E	0.0897	26.82261	4.62516	0.4860
4	8.810	BB	0.1112	8.74934	1.17037	0.1585
5	9.929	BV	0.1070	53.16010	7.29892	0.9632
6	10.127	VB	0.0905	48.62735	8.27775	0.8811
7	10.405	BV	0.0904	13.34979	2.34670	0.2419
8	10.577	VB	0.0906	34.97092	6.12713	0.6336
9	11.562	BB	0.1235	30.75161	3.60618	0.5572
10	18.020	BB	0.0980	16.86144	2.58738	0.3055
Totals :				5519.22103	890.05962	

Signal 3: DAD1 E, Sig=280,4 Ref=360,100

Peak #	RetTime [min]	Type	Width [min]	Area [mAU*s]	Height [mAU]	Area %
1	6.480	BB	0.1044	11.23754	1.71594	0.3691
2	7.728	BV R	0.0957	2950.54419	480.30511	96.9017
3	8.173	VB E	0.0901	20.63910	3.53737	0.6778
4	9.929	BV	0.1043	13.56305	1.92282	0.4454
5	10.128	VB	0.0900	16.89693	2.89942	0.5549
6	10.577	VB	0.0904	17.68674	3.10874	0.5809
7	11.561	BB	0.1138	14.31662	1.86088	0.4702
Totals :				3044.88416	495.35028	

==

*** End of Report ***

Data File C:\Chem32\1\Data\RD SIRT2 2016-07-20 16-41-09\OnlineEdited104.D
Sample Name: LC-0540

Compound 19b

===

Acq. Operator : SYSTEM Seq. Line : 34
Acq. Instrument : CDD Location : P1-D-08
Injection Date : 7/21/2016 5:15:49 AM Inj : 1
Inj Volume : 5.000 µl
Different Inj Volume from Sample Entry! Actual Inj Volume : 10.000 µl
Method : C:\Chem32\1\Data\RD SIRT2 2016-07-20 16-41-09\RD VCP Methanol 40-100.M (Sequence Method)
Last changed : 7/20/2016 4:41:10 PM by SYSTEM
Method Info : test

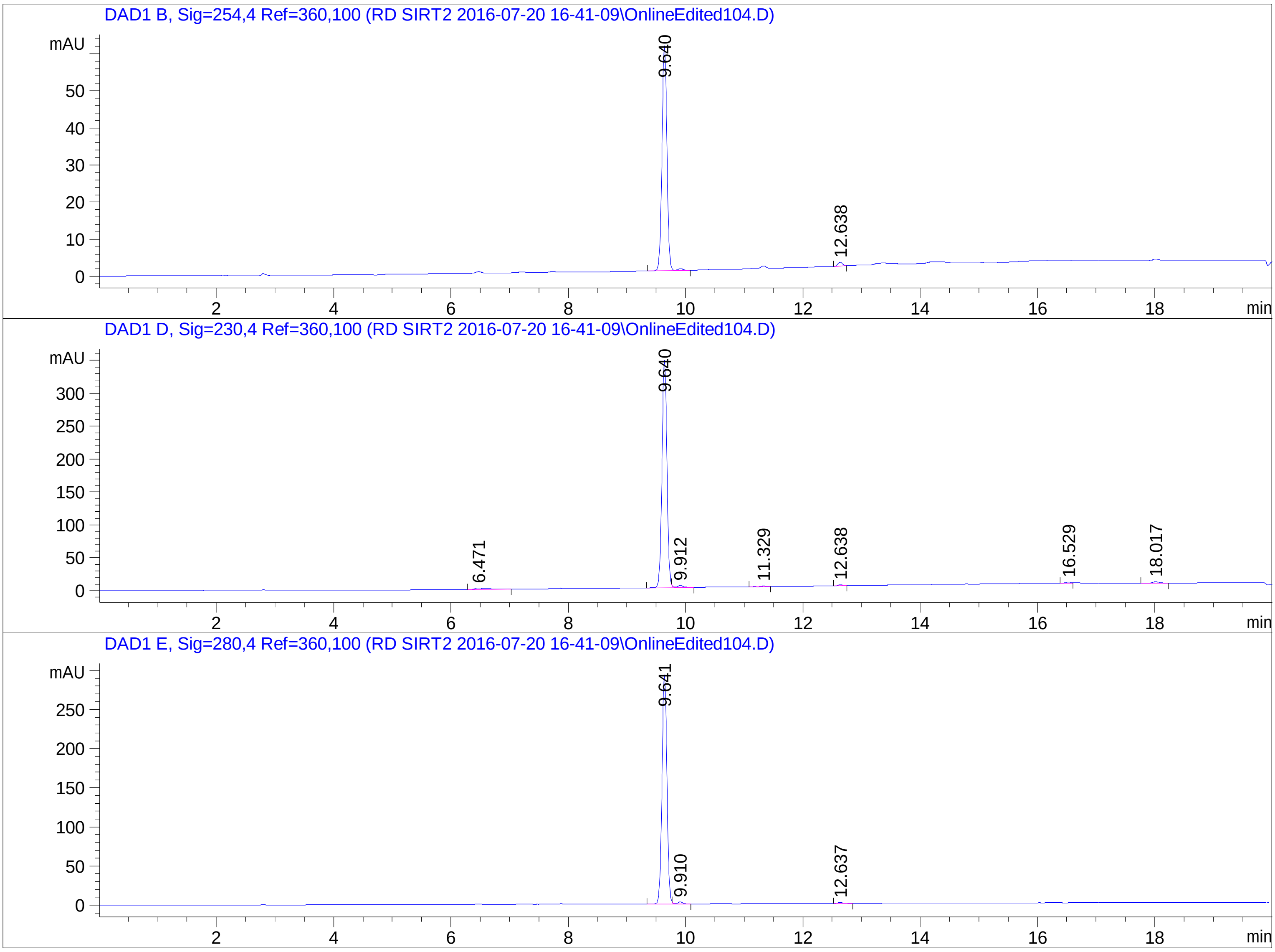

===

Area Percent Report

===

Sorted By : Signal
Multiplier : 1.0000
Dilution : 1.0000
Use Multiplier & Dilution Factor with ISTDs

Signal 1: DAD1 B, Sig=254,4 Ref=360,100

Peak #	RetTime [min]	Type	Width [min]	Area [mAU*s]	Height [mAU]	Area %
1	9.640	BV R	0.0908	357.65024	60.62461	98.5311
2	12.638	BB	0.0809	5.33194	1.02185	1.4689

Totals : 362.98218 61.64646

Signal 2: DAD1 D, Sig=230,4 Ref=360,100

Peak #	RetTime [min]	Type	Width [min]	Area [mAU*s]	Height [mAU]	Area %
1	6.471	BB	0.1748	32.09124	2.51630	1.5179
2	9.640	BV R	0.0903	2022.75220	345.73767	95.6763
3	9.912	VB E	0.0975	19.74686	3.05138	0.9340
4	11.329	BB	0.1044	7.48140	1.05923	0.3539
5	12.638	BB	0.0822	7.88355	1.47983	0.3729
6	16.529	BV	0.0961	8.74571	1.37774	0.4137
7	18.017	BB	0.1013	15.46028	2.33373	0.7313

Totals : 2114.16124 357.55588

Signal 3: DAD1 E, Sig=280,4 Ref=360,100

Peak #	RetTime [min]	Type	Width [min]	Area [mAU*s]	Height [mAU]	Area %
1	9.641	BV R	0.0902	1712.82117	293.09143	98.6368
2	9.910	VB E	0.0929	15.87911	2.61360	0.9144
3	12.637	BB	0.0917	7.79266	1.26766	0.4488

Totals : 1736.49294 296.97269

===

*** End of Report ***

Data File C:\Chem32\1\Data\RD SIRT2 2016-07-21 10-43-19\1FD-0401.D
Sample Name: LC-0535

Compound 19c

===

Acq. Operator : SYSTEM   Seq. Line : 4
Acq. Instrument : CDD   Location : P1-F-04
Injection Date : 7/21/2016 11:52:41 AM   Inj : 1
Inj Volume : 5.000 µl
Different Inj Volume from Sample Entry! Actual Inj Volume : 10.000 µl
Method : C:\Chem32\1\Data\RD SIRT2 2016-07-21 10-43-19\RD VCP Methanol 40-100.M (Sequence Method)
Last changed : 7/21/2016 10:43:19 AM by SYSTEM
Method Info : test

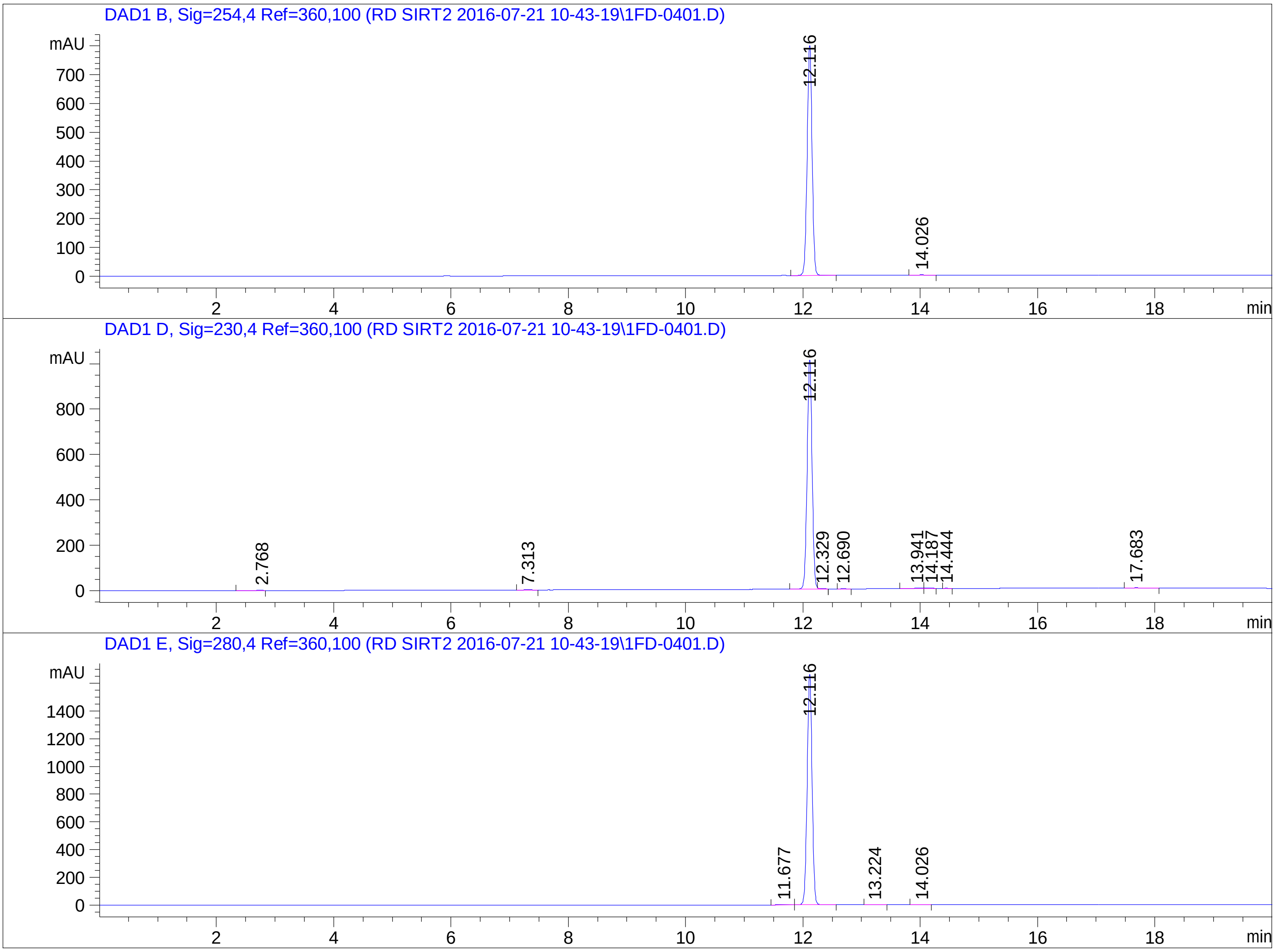

===

Area Percent Report

===

Sorted By : Signal
Multiplier : 1.0000
Dilution : 1.0000
Use Multiplier & Dilution Factor with ISTDs

Signal 1: DAD1 B, Sig=254,4 Ref=360,100

Peak #	RetTime [min]	Type	Width [min]	Area [mAU*s]	Height [mAU]	Area %
1	12.116	BB	0.0916	4636.44238	800.61377	99.7084
2	14.026	BB	0.1059	13.55839	1.84174	0.2916

Totals : 4650.00077 802.45551

Signal 2: DAD1 D, Sig=230,4 Ref=360,100

Peak #	RetTime [min]	Type	Width [min]	Area [mAU*s]	Height [mAU]	Area %
1	2.768	BB	0.1049	8.82315	1.13259	0.1481
2	7.313	BB	0.1045	7.48587	1.11323	0.1256
3	12.116	BV R	0.0916	5858.89453	1011.65424	98.3296
4	12.329	VB E	0.0796	7.75098	1.46916	0.1301
5	12.690	BB	0.0794	5.97229	1.17264	0.1002
6	13.941	BV	0.1073	23.02696	2.94097	0.3865
7	14.187	VB	0.1101	24.69013	2.99834	0.4144
8	14.444	VB	0.0698	5.99529	1.35079	0.1006
9	17.683	BB	0.1032	15.78221	2.26720	0.2649

Totals : 5958.42142 1026.09916

Signal 3: DAD1 E, Sig=280,4 Ref=360,100

Peak #	RetTime [min]	Type	Width [min]	Area [mAU*s]	Height [mAU]	Area %
1	11.677	BB	0.1073	11.49272	1.57119	0.1186
2	12.116	BB	0.0917	9655.46973	1665.05383	99.6822
3	13.224	BB	0.0979	7.71723	1.15559	0.0797
4	14.026	BB	0.0871	11.57047	2.07252	0.1195

Totals : 9686.25015 1669.85313

==

*** End of Report ***

Data File C:\Chem32\1\Data\RD SIRT2 2016-07-20 16-41-09\OnlineEdited106.D
Sample Name: LC-0541

Compound 20a

===

Acq. Operator : SYSTEM   Seq. Line : 36
Acq. Instrument : CDD   Location : P1-E-01
Injection Date : 7/21/2016 6:01:29 AM   Inj : 1
Inj Volume : 5.000 µl
Different Inj Volume from Sample Entry! Actual Inj Volume : 10.000 µl
Method : C:\Chem32\1\Data\RD SIRT2 2016-07-20 16-41-09\RD VCP Methanol 40-100.M (Sequence Method)
Last changed : 7/20/2016 4:41:10 PM by SYSTEM
Method Info : test

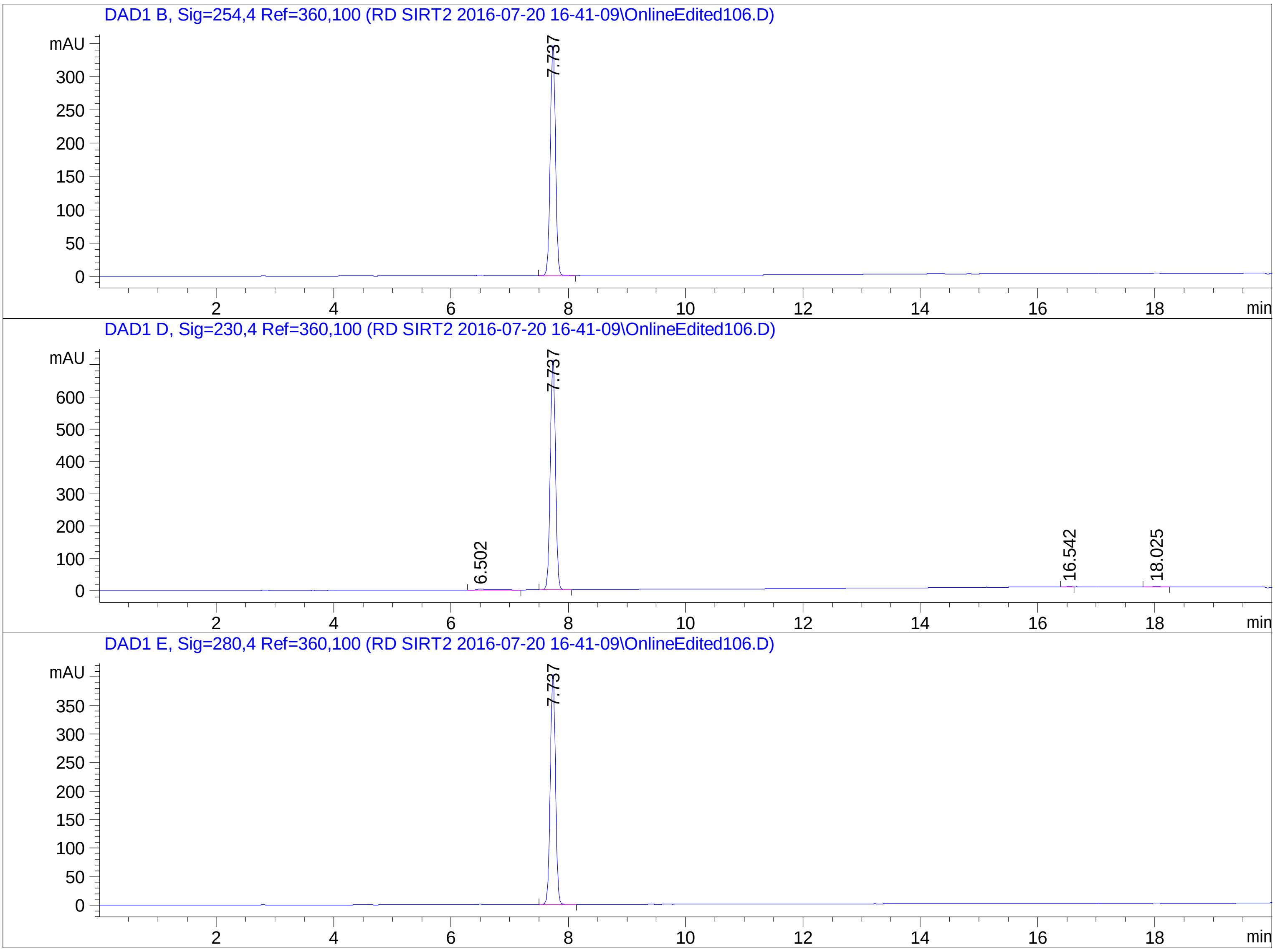

===

Area Percent Report

===

Sorted By : Signal
Multiplier : 1.0000
Dilution : 1.0000
Use Multiplier & Dilution Factor with ISTDs

Signal 1: DAD1 B, Sig=254,4 Ref=360,100

Peak #	RetTime [min]	Type	Width [min]	Area [mAU*s]	Height [mAU]	Area %
1	7.737	BB	0.0957	2123.18237	345.43533	100.0000

Totals : 2123.18237 345.43533

Signal 2: DAD1 D, Sig=230,4 Ref=360,100

Peak #	RetTime [min]	Type	Width [min]	Area [mAU*s]	Height [mAU]	Area %
1	6.502	BB	0.1744	46.79127	3.67814	1.0524
2	7.737	BB	0.0958	4376.38916	710.96106	98.4326
3	16.542	BV	0.0961	8.32808	1.31062	0.1873
4	18.025	BB	0.0983	14.56958	2.22677	0.3277

Totals : 4446.07809 718.17659

Signal 3: DAD1 E, Sig=280,4 Ref=360,100

Peak #	RetTime [min]	Type	Width [min]	Area [mAU*s]	Height [mAU]	Area %
1	7.737	BB	0.0957	2477.27515	403.12604	100.0000

Totals : 2477.27515 403.12604

==

*** End of Report ***

Data File C:\Chem32\1\Data\RD SIRT2 2016-07-20 16-41-09\OnlineEdited107.D
Sample Name: LC-0556

Compound 20b

===

Acq. Operator : SYSTEM   Seq. Line : 37
Acq. Instrument : CDD   Location : P1-E-02
Injection Date : 7/21/2016 6:24:19 AM   Inj : 1
Inj Volume : 5.000 µl
Different Inj Volume from Sample Entry! Actual Inj Volume : 10.000 µl
Method : C:\Chem32\1\Data\RD SIRT2 2016-07-20 16-41-09\RD VCP Methanol 40-100.M (Sequence Method)
Last changed : 7/20/2016 4:41:10 PM by SYSTEM
Method Info : test

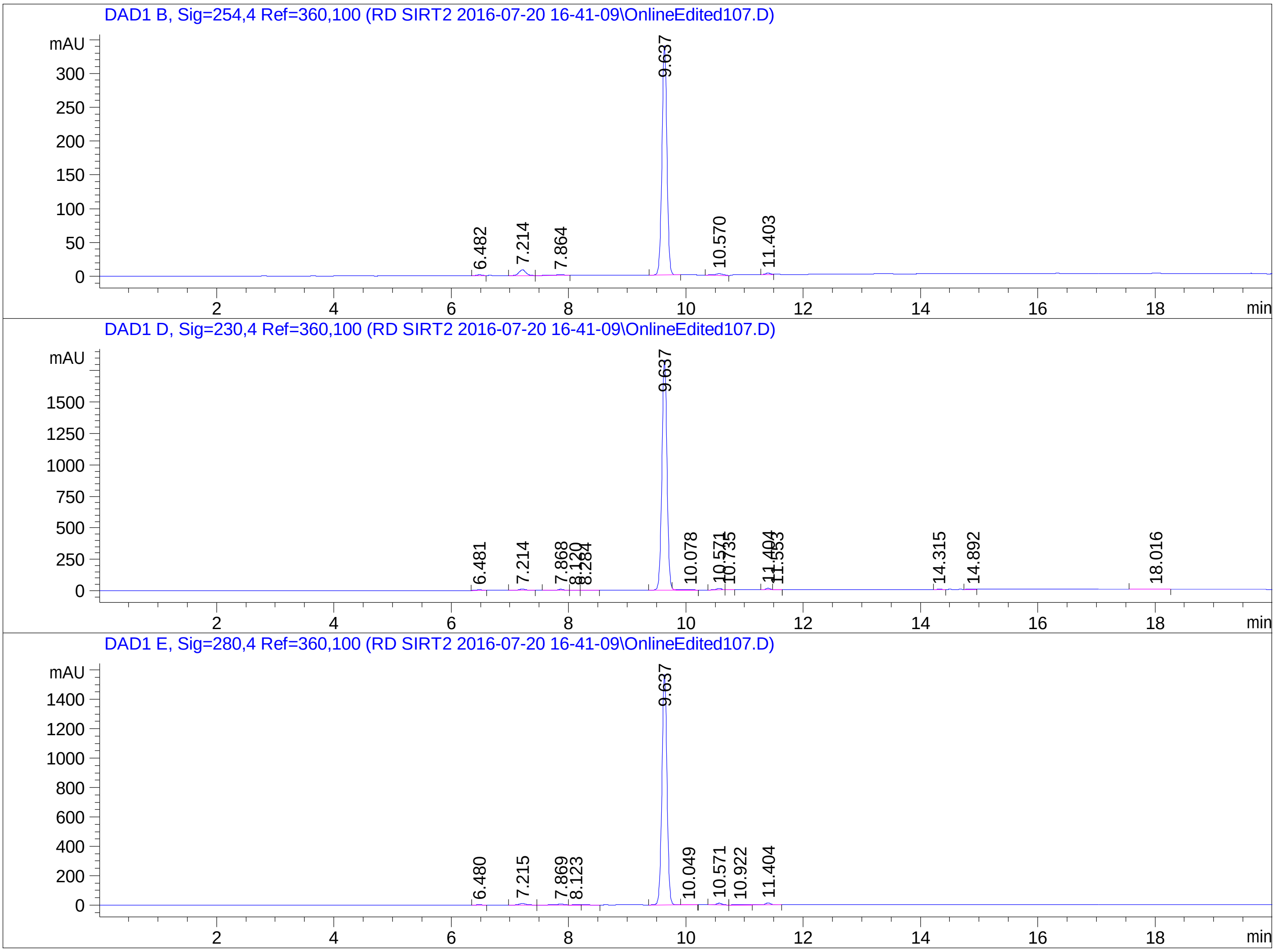

===

Area Percent Report

===

Sorted By : Signal
Multiplier : 1.0000
Dilution : 1.0000
Use Multiplier & Dilution Factor with ISTDs

Signal 1: DAD1 B, Sig=254,4 Ref=360,100

Peak #	RetTime [min]	Type	Width [min]	Area [mAU*s]	Height [mAU]	Area %
1	6.482	BB	0.0985	7.06549	1.13634	0.3320
2	7.214	BB	0.1242	68.22322	8.44874	3.2053
3	7.864	BB	0.1680	16.99800	1.43656	0.7986
4	9.637	BB	0.0908	1999.68823	339.21512	93.9497
5	10.570	BB	0.1339	23.19562	2.46303	1.0898
6	11.403	BV	0.0891	13.29632	2.31204	0.6247

Totals : 2128.46688 355.01183

Signal 2: DAD1 D, Sig=230,4 Ref=360,100

Peak #	RetTime [min]	Type	Width [min]	Area [mAU*s]	Height [mAU]	Area %
1	6.481	BB	0.1024	35.25259	5.52707	0.3087
2	7.214	BB	0.1272	78.76927	9.45400	0.6898
3	7.868	BV	0.1152	60.45124	7.91050	0.5294
4	8.120	VV	0.1006	7.61487	1.15981	0.0667
5	8.284	VB	0.1178	10.86510	1.32322	0.0951
6	9.637	BV R	0.0942	1.10005e4	1827.47119	96.3303
7	10.078	VB E	0.2558	27.59331	1.54927	0.2416
8	10.571	BV	0.0924	71.80737	11.89938	0.6288
9	10.735	VB	0.0830	17.12562	3.17276	0.1500
10	11.404	BV R	0.0883	70.24521	12.36404	0.6151
11	11.553	VB E	0.0760	6.02960	1.25727	0.0528
12	14.315	BV	0.0928	7.17471	1.14973	0.0628
13	14.892	VV	0.1179	10.99280	1.25834	0.0963
14	18.016	BB	0.1025	15.13963	2.19490	0.1326

Totals : 1.14196e4 1887.69149

Signal 3: DAD1 E, Sig=280,4 Ref=360,100

Peak #	RetTime [min]	Type	Width [min]	Area [mAU*s]	Height [mAU]	Area %
1	6.480	BB	0.1010	8.69016	1.38820	0.0890
2	7.215	BB	0.1295	77.15298	9.04280	0.7906
3	7.869	BV R	0.1311	56.74624	6.29980	0.5815
4	8.123	VV E	0.1126	8.68707	1.17012	0.0890
5	9.637	BV R	0.0941	9408.98438	1566.49939	96.4107
6	10.049	VB E	0.1236	8.80943	1.12216	0.0903
7	10.571	BB	0.0993	76.16367	11.49812	0.7804
8	10.922	BV E	0.1871	26.94633	1.92803	0.2761
9	11.404	VB R	0.0976	87.09547	13.44311	0.8924

Totals : 9759.27572 1612.39174

===

*** End of Report ***

Data File C:\Chem32\1\Data\RD SIRT2 2016-07-20 16-41-09\OnlineEdited108.D
Sample Name: LC-0561

Compound 20c

===

Acq. Operator : SYSTEM Seq. Line : 38
Acq. Instrument : CDD Location : P1-E-03
Injection Date : 7/21/2016 6:47:09 AM Inj : 1
Inj Volume : 5.000 µl
Different Inj Volume from Sample Entry! Actual Inj Volume : 10.000 µl
Method : C:\Chem32\1\Data\RD SIRT2 2016-07-20 16-41-09\RD VCP Methanol 40-100.M (Sequence Method)
Last changed : 7/20/2016 4:41:10 PM by SYSTEM
Method Info : test

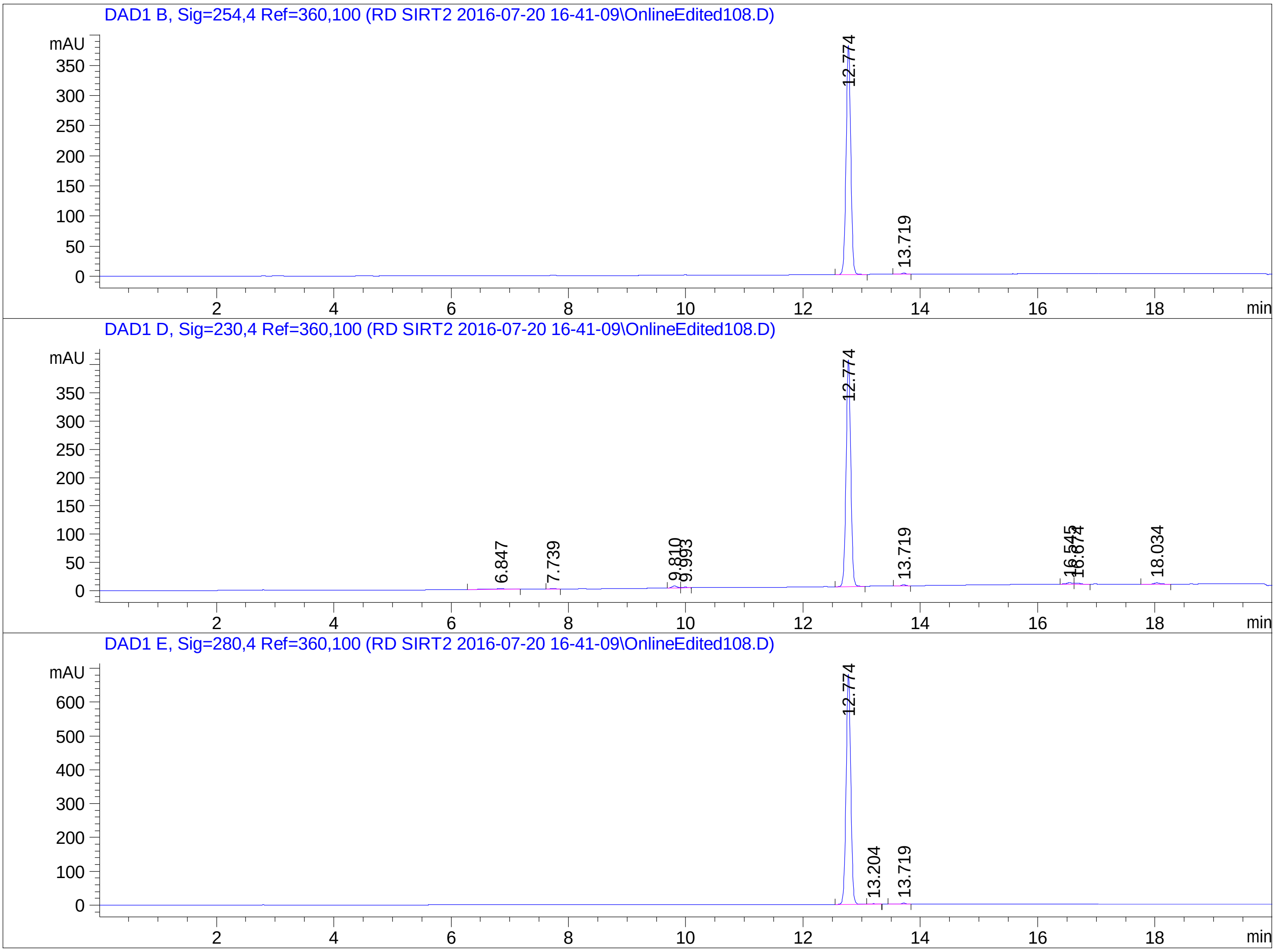

===

Area Percent Report

===

Sorted By : Signal
Multiplier : 1.0000
Dilution : 1.0000
Use Multiplier & Dilution Factor with ISTDs

Signal 1: DAD1 B, Sig=254,4 Ref=360,100

Peak #	RetTime [min]	Type	Width [min]	Area [mAU*s]	Height [mAU]	Area %
1	12.774	BB	0.0840	2090.93628	380.90067	99.5268
2	13.719	BB	0.0806	9.94177	1.91527	0.4732

Totals : 2100.87805 382.81594

Signal 2: DAD1 D, Sig=230,4 Ref=360,100

Peak #	RetTime [min]	Type	Width [min]	Area [mAU*s]	Height [mAU]	Area %
1	6.847	BB	0.2140	33.59001	2.06222	1.4441
2	7.739	BB	0.0951	7.82613	1.28368	0.3365
3	9.810	BV	0.0925	20.14105	3.43261	0.8659
4	9.993	VB	0.0801	6.33969	1.22992	0.2726
5	12.774	BB	0.0841	2205.48584	401.50620	94.8173
6	13.719	BB	0.0809	8.95713	1.71712	0.3851
7	16.545	BV	0.0977	16.66931	2.56943	0.7166
8	16.674	VV	0.0992	12.54076	1.84791	0.5391
9	18.034	BB	0.1014	14.48838	2.18467	0.6229

Totals : 2326.03830 417.83375

Signal 3: DAD1 E, Sig=280,4 Ref=360,100

Peak #	RetTime [min]	Type	Width [min]	Area [mAU*s]	Height [mAU]	Area %
1	12.774	BV R	0.0840	3734.02515	680.39209	99.2932
2	13.204	VB E	0.0838	8.70740	1.64330	0.2315
3	13.719	BB	0.0812	17.87073	3.40702	0.4752

Totals : 3760.60327 685.44242

==

*** End of Report ***

Data File C:\Chem32\1\Data\RD SIRT2 2016-07-20 16-41-09\OnlineEdited109.D
Sample Name: LC-0542

Compound 21a

===

Acq. Operator : SYSTEM Seq. Line : 39
Acq. Instrument : CDD Location : P1-E-04
Injection Date : 7/21/2016 7:10:00 AM Inj : 1
Inj Volume : 5.000 µl
Different Inj Volume from Sample Entry! Actual Inj Volume : 10.000 µl
Method : C:\Chem32\1\Data\RD SIRT2 2016-07-20 16-41-09\RD VCP Methanol 40-100.M (Sequence Method)
Last changed : 7/20/2016 4:41:10 PM by SYSTEM
Method Info : test

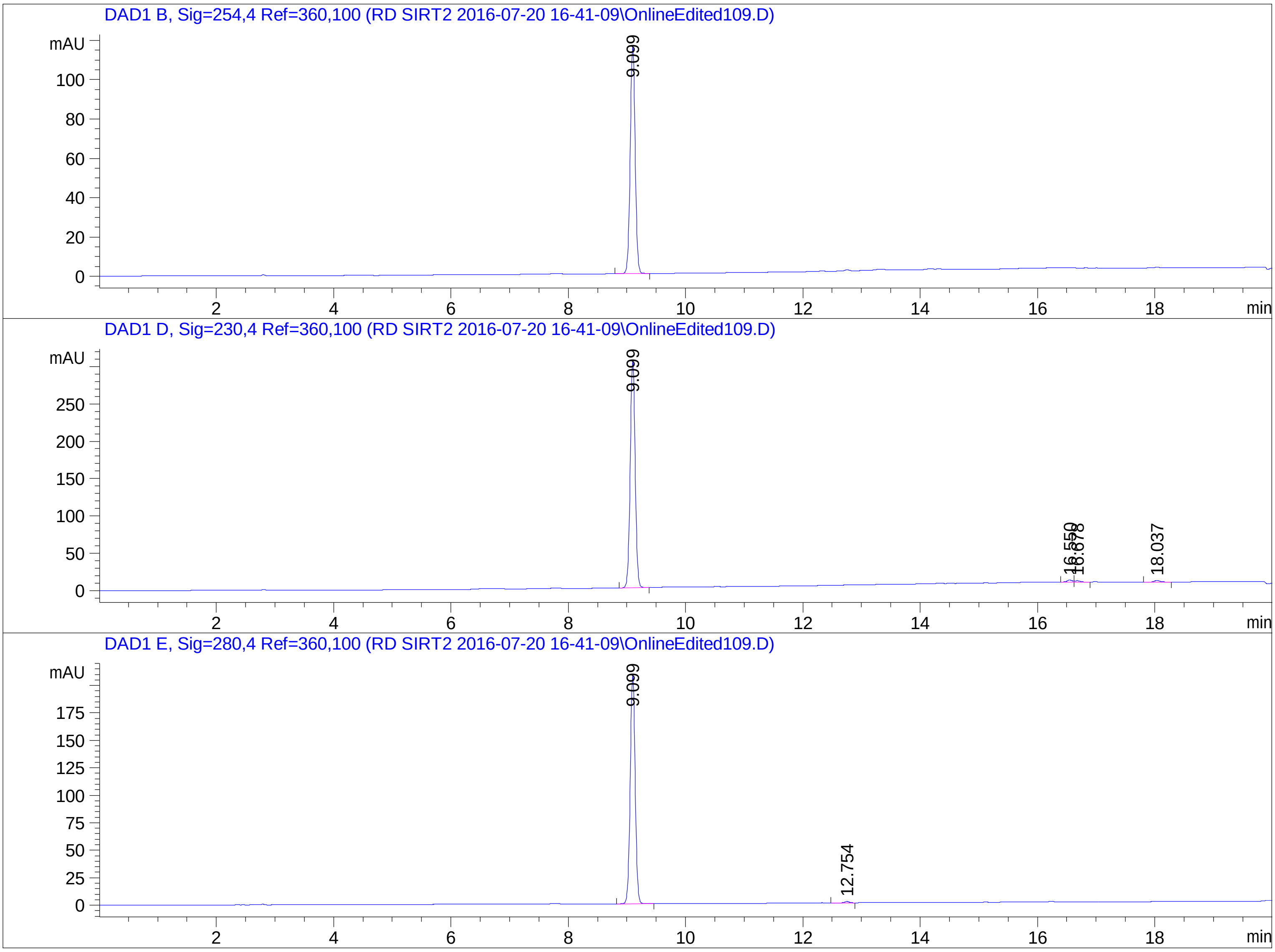

===

Area Percent Report

===

Sorted By : Signal
Multiplier : 1.0000
Dilution : 1.0000
Use Multiplier & Dilution Factor with ISTDs

Signal 1: DAD1 B, Sig=254,4 Ref=360,100

Peak #	RetTime [min]	Type	Width [min]	Area [mAU*s]	Height [mAU]	Area %
1	9.099	BB	0.0906	681.82251	115.90252	100.0000

Totals : 681.82251 115.90252

Signal 2: DAD1 D, Sig=230,4 Ref=360,100

Peak #	RetTime [min]	Type	Width [min]	Area [mAU*s]	Height [mAU]	Area %
1	9.099	BB	0.0906	1791.48523	304.45996	97.6799
2	16.550	BV	0.0959	16.20405	2.55859	0.8835
3	16.678	VV	0.1009	12.12503	1.74776	0.6611
4	18.037	BB	0.1023	14.22153	2.11895	0.7754

Totals : 1834.03584 310.88526

Signal 3: DAD1 E, Sig=280,4 Ref=360,100

Peak #	RetTime [min]	Type	Width [min]	Area [mAU*s]	Height [mAU]	Area %
1	9.099	BB	0.0906	1226.75720	208.50642	99.4463
2	12.754	BB	0.0869	6.83019	1.19077	0.5537

Totals : 1233.58739 209.69719

==

*** End of Report ***

Data File C:\Chem32\1\Data\RD SIRT2 2016-07-20 16-41-09\OnlineEdited110.D
Sample Name: LC-0539

Compound 21b

===

Acq. Operator : SYSTEM   Seq. Line : 40
Acq. Instrument : CDD   Location : P1-E-05
Injection Date : 7/21/2016 7:32:49 AM   Inj : 1
Inj Volume : 5.000 µl
Different Inj Volume from Sample Entry! Actual Inj Volume : 10.000 µl
Method : C:\Chem32\1\Data\RD SIRT2 2016-07-20 16-41-09\RD VCP Methanol 40-100.M (Sequence Method)
Last changed : 7/20/2016 4:41:10 PM by SYSTEM
Method Info : test

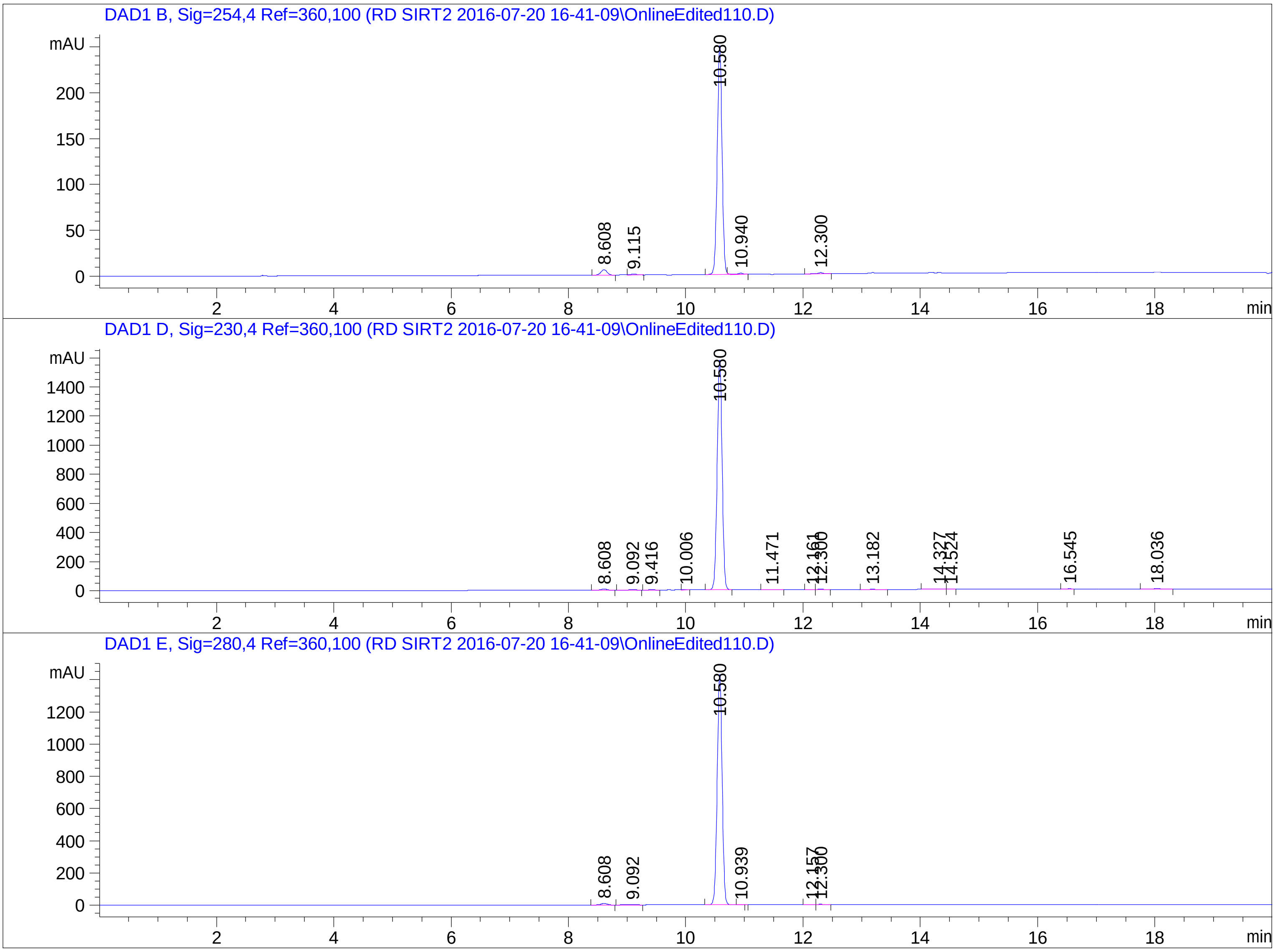

===

Area Percent Report

===

Sorted By : Signal
Multiplier : 1.0000
Dilution : 1.0000
Use Multiplier & Dilution Factor with ISTDs

Signal 1: DAD1 B, Sig=254,4 Ref=360,100

Peak #	RetTime [min]	Type	Width [min]	Area [mAU*s]	Height [mAU]	Area %
1	8.608	BB	0.1125	43.16435	5.95663	2.9140
2	9.115	VB	0.1029	7.48319	1.10609	0.5052
3	10.580	BV R	0.0859	1412.17920	249.97800	95.3351
4	10.940	VB E	0.1119	10.10654	1.31170	0.6823
5	12.300	BB	0.0999	8.34618	1.24943	0.5634

Totals : 1481.27946 259.60185

Signal 2: DAD1 D, Sig=230,4 Ref=360,100

Peak #	RetTime [min]	Type	Width [min]	Area [mAU*s]	Height [mAU]	Area %
1	8.608	BB	0.1126	57.09243	7.86936	0.6046
2	9.092	BB	0.1139	30.90240	4.10267	0.3273
3	9.416	BB	0.1195	9.66486	1.28929	0.1024
4	10.006	VV	0.0900	6.10716	1.01720	0.0647
5	10.580	BB	0.0922	9248.90625	1582.37256	97.9468
6	11.471	BB	0.1342	11.20872	1.14522	0.1187
7	12.161	BV	0.0838	6.52814	1.19430	0.0691
8	12.300	VB	0.0896	25.34690	4.25019	0.2684
9	13.182	BB	0.1270	9.02999	1.00291	0.0956
10	14.327	BV	0.1030	8.76171	1.23206	0.0928
11	14.524	VV	0.0835	5.76590	1.02630	0.0611
12	16.545	BV	0.0977	8.43869	1.29980	0.0894
13	18.036	BB	0.1055	15.03143	2.15064	0.1592

Totals : 9442.78459 1609.95251

Signal 3: DAD1 E, Sig=280,4 Ref=360,100

Peak #	RetTime [min]	Type	Width [min]	Area [mAU*s]	Height [mAU]	Area %
1	8.608	BB	0.1124	63.16225	8.72879	0.7418
2	9.092	VB R	0.1181	25.07605	3.17506	0.2945
3	10.580	BV R	0.0923	8393.91309	1434.25732	98.5845
4	10.939	VB E	0.0742	5.24810	1.17199	0.0616
5	12.157	BV	0.0886	7.34116	1.24842	0.0862
6	12.300	VB	0.0897	19.69093	3.39462	0.2313

Totals : 8514.43157 1451.97620

==

*** End of Report ***

Data File C:\Chem32\1\Data\RD SIRT2 2016-07-20 16-41-09\OnlineEdited111.D
Sample Name: LC-0538

Compound 21c

===

Acq. Operator : SYSTEM Seq. Line : 41
Acq. Instrument : CDD Location : P1-E-06
Injection Date : 7/21/2016 7:55:39 AM Inj : 1
Inj Volume : 5.000 µl
Different Inj Volume from Sample Entry! Actual Inj Volume : 10.000 µl
Method : C:\Chem32\1\Data\RD SIRT2 2016-07-20 16-41-09\RD VCP Methanol 40-100.M (Sequence Method)
Last changed : 7/20/2016 4:41:10 PM by SYSTEM
Method Info : test

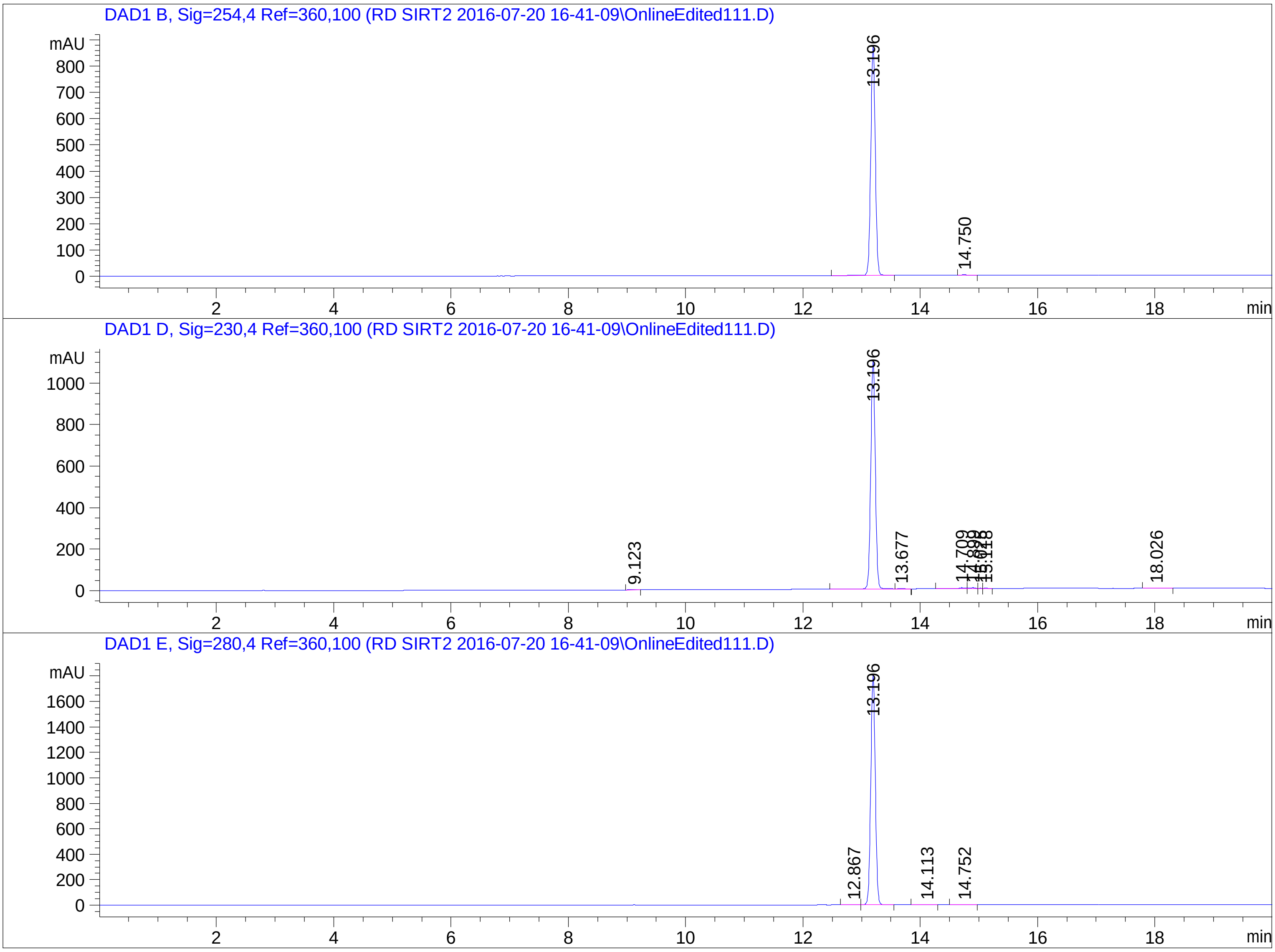

===

Area Percent Report

===

Sorted By : Signal
Multiplier : 1.0000
Dilution : 1.0000
Use Multiplier & Dilution Factor with ISTDs

Signal 1: DAD1 B, Sig=254,4 Ref=360,100

Peak #	RetTime [min]	Type	Width [min]	Area [mAU*s]	Height [mAU]	Area %
1	13.196	VB R	0.0824	4682.74854	876.19794	99.7448
2	14.750	BB	0.0925	11.97920	1.92716	0.2552

Totals : 4694.72773 878.12510

Signal 2: DAD1 D, Sig=230,4 Ref=360,100

Peak #	RetTime [min]	Type	Width [min]	Area [mAU*s]	Height [mAU]	Area %
1	9.123	BB	0.0890	7.01888	1.22221	0.1159
2	13.196	VV R	0.0828	5943.80762	1104.84973	98.1756
3	13.677	VB E	0.0853	7.46227	1.33303	0.1233
4	14.709	BV	0.1267	37.53565	4.10232	0.6200
5	14.899	VV	0.1047	30.74249	3.95327	0.5078
6	15.025	VV	0.0631	5.30016	1.25890	0.0875
7	15.118	VB	0.0708	8.10740	1.72679	0.1339
8	18.026	BB	0.1015	14.28895	2.09739	0.2360

Totals : 6054.26342 1120.54363

Signal 3: DAD1 E, Sig=280,4 Ref=360,100

Peak #	RetTime [min]	Type	Width [min]	Area [mAU*s]	Height [mAU]	Area %
1	12.867	BV	0.0981	12.18941	1.82092	0.1255
2	13.196	VB	0.0824	9681.74023	1811.56824	99.6431
3	14.113	BB	0.1002	9.21157	1.37466	0.0948
4	14.752	VB R	0.0873	13.27586	2.23474	0.1366

Totals : 9716.41707 1816.99856

==
*** End of Report ***

Data File C:\Chem32\1\Data\RD SIRT2 2016-07-20 16-41-09\OnlineEdited112.D
Sample Name: LC-0557

Compound 22a

===

Acq. Operator : SYSTEM Seq. Line : 42
Acq. Instrument : CDD Location : P1-E-07
Injection Date : 7/21/2016 8:18:30 AM Inj : 1
Inj Volume : 5.000 µl
Different Inj Volume from Sample Entry! Actual Inj Volume : 10.000 µl
Method : C:\Chem32\1\Data\RD SIRT2 2016-07-20 16-41-09\RD VCP Methanol 40-100.M (Sequence Method)
Last changed : 7/20/2016 4:41:10 PM by SYSTEM
Method Info : test

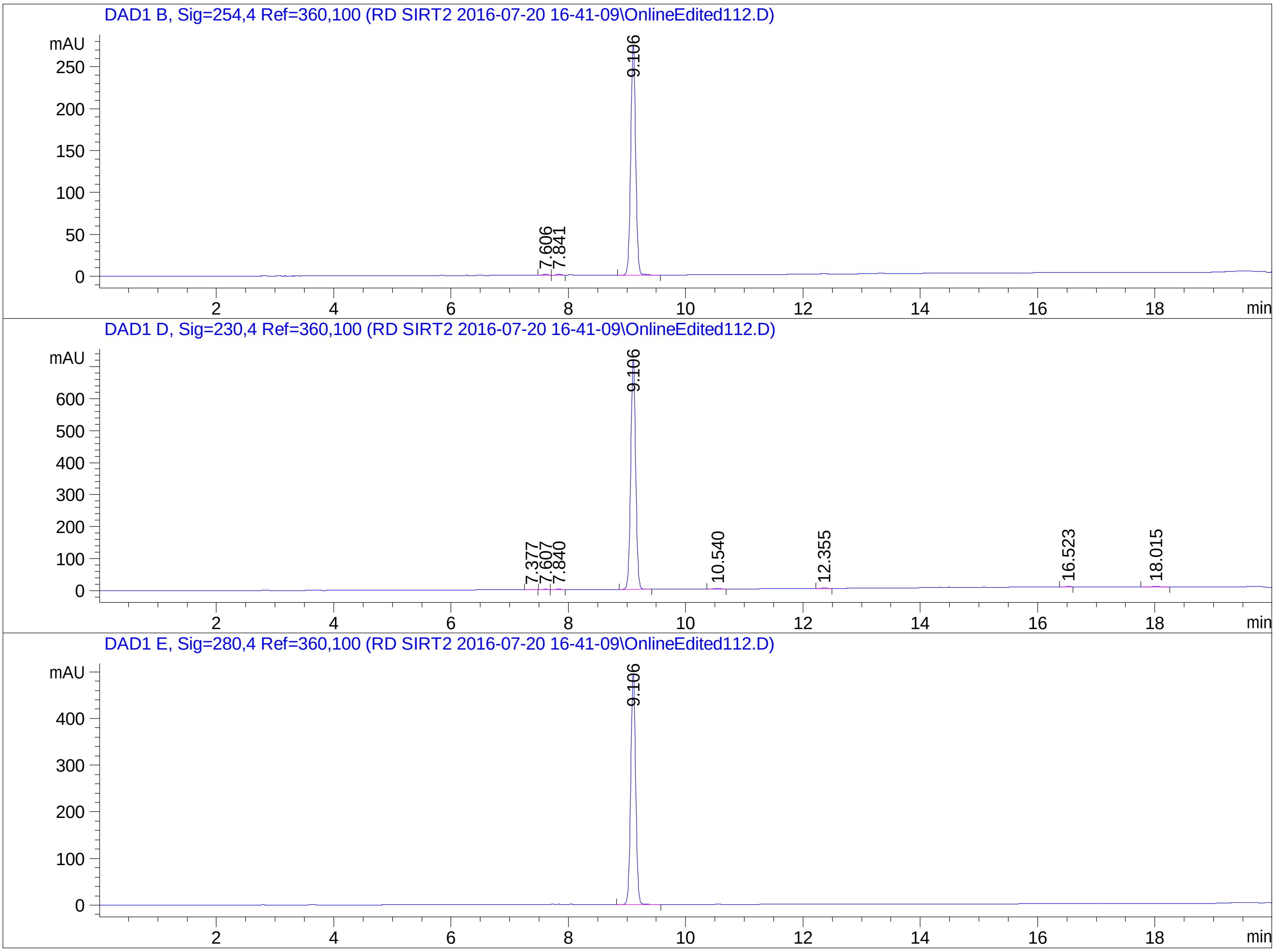

===

Area Percent Report

===

Sorted By : Signal
Multiplier : 1.0000
Dilution : 1.0000
Use Multiplier & Dilution Factor with ISTDs

Signal 1: DAD1 B, Sig=254,4 Ref=360,100

Peak #	RetTime [min]	Type	Width [min]	Area [mAU*s]	Height [mAU]	Area %
1	7.606	BB	0.0914	6.02495	1.04301	0.3727
2	7.841	BB	0.0892	6.19659	1.10918	0.3834
3	9.106	BB	0.0903	1604.17664	273.87274	99.2439

Totals : 1616.39818 276.02493

Signal 2: DAD1 D, Sig=230,4 Ref=360,100

Peak #	RetTime [min]	Type	Width [min]	Area [mAU*s]	Height [mAU]	Area %
1	7.377	BB	0.0900	7.79954	1.37804	0.1830
2	7.607	BB	0.0853	7.24946	1.33699	0.1701
3	7.840	BB	0.0906	9.60503	1.63234	0.2254
4	9.106	BB	0.0903	4192.09863	716.41174	98.3712
5	10.540	BB	0.0935	12.47423	2.03410	0.2927
6	12.355	BB	0.1147	7.26462	1.02450	0.1705
7	16.523	BV	0.0998	8.53197	1.31391	0.2002
8	18.015	BB	0.1022	16.48679	2.45886	0.3869

Totals : 4261.51027 727.59050

Signal 3: DAD1 E, Sig=280,4 Ref=360,100

Peak #	RetTime [min]	Type	Width [min]	Area [mAU*s]	Height [mAU]	Area %
1	9.106	BB	0.0902	2883.72827	493.02127	100.0000

Totals : 2883.72827 493.02127

==

*** End of Report ***

Data File C:\Chem32\1\Data\RD SIRT2 2016-07-20 16-41-09\OnlineEdited113.D
Sample Name: LC-0537

Compound 22b

===

Acq. Operator : SYSTEM Seq. Line : 43
Acq. Instrument : CDD Location : P1-E-08
Injection Date : 7/21/2016 8:41:20 AM Inj : 1
Inj Volume : 5.000 µl
Different Inj Volume from Sample Entry! Actual Inj Volume : 10.000 µl
Method : C:\Chem32\1\Data\RD SIRT2 2016-07-20 16-41-09\RD VCP Methanol 40-100.M (Sequence Method)
Last changed : 7/20/2016 4:41:10 PM by SYSTEM
Method Info : test

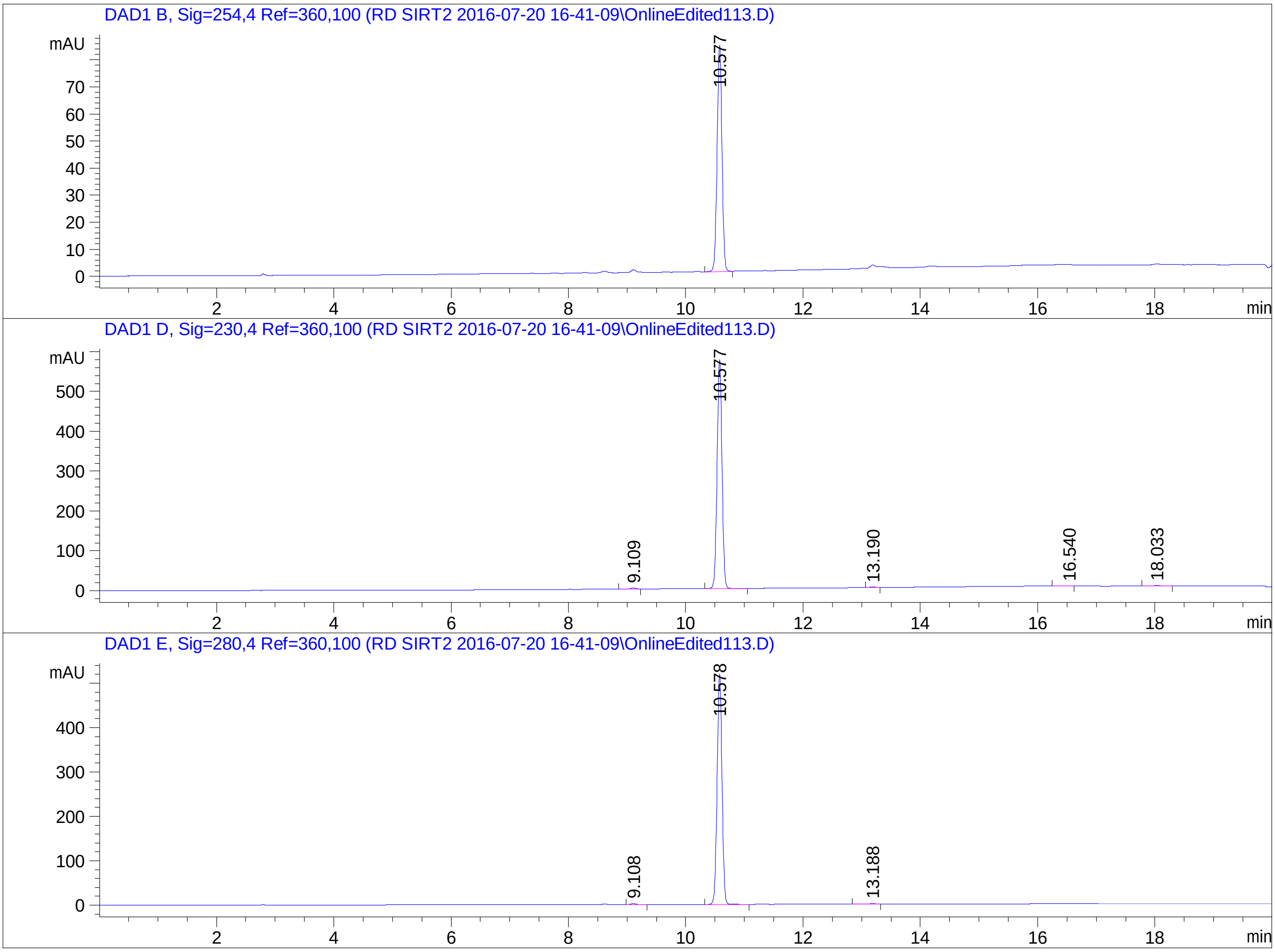

===

Area Percent Report

===

Sorted By : Signal
Multiplier : 1.0000
Dilution : 1.0000
Use Multiplier & Dilution Factor with ISTDs

Signal 1: DAD1 B, Sig=254,4 Ref=360,100

Peak #	RetTime [min]	Type	Width [min]	Area [mAU*s]	Height [mAU]	Area %
1	10.577	BB	0.0889	478.08603	83.41353	100.0000

Totals : 478.08603 83.41353

Signal 2: DAD1 D, Sig=230,4 Ref=360,100

Peak #	RetTime [min]	Type	Width [min]	Area [mAU*s]	Height [mAU]	Area %
1	9.109	BB	0.0926	16.32789	2.77571	0.4867
2	10.577	BB	0.0893	3306.40918	573.17480	98.5506
3	13.190	BB	0.0875	8.59849	1.53138	0.2563
4	16.540	BV	0.1163	9.56588	1.15925	0.2851
5	18.033	BB	0.1026	14.13674	2.09842	0.4214

Totals : 3355.03818 580.73956

Signal 3: DAD1 E, Sig=280,4 Ref=360,100

Peak #	RetTime [min]	Type	Width [min]	Area [mAU*s]	Height [mAU]	Area %
1	9.108	BB	0.0965	12.33896	1.98497	0.4105
2	10.578	BB	0.0893	2986.01172	517.88837	99.3293
3	13.188	BB	0.0925	7.82428	1.29566	0.2603

Totals : 3006.17495 521.16900

==

*** End of Report ***

Data File C:\Chem32\1\Data\RD SIRT2 2016-07-20 16-41-09\OnlineEdited114.D
Sample Name: LC-0534

Compound 22c

===

```
Acq. Operator   : SYSTEM                          Seq. Line :  44
Acq. Instrument : CDD                              Location :   P1-E-09
Injection Date  : 7/21/2016 9:04:09 AM                  Inj :   1
                                                 Inj Volume : 5.000 µl
Different Inj Volume from Sample Entry!  Actual Inj Volume : 10.000 µl
Method          : C:\Chem32\1\Data\RD SIRT2 2016-07-20 16-41-09\RD VCP Methanol 40-100.M (
                  Sequence Method)
Last changed    : 7/20/2016 4:41:10 PM by SYSTEM
Method Info     : test
```

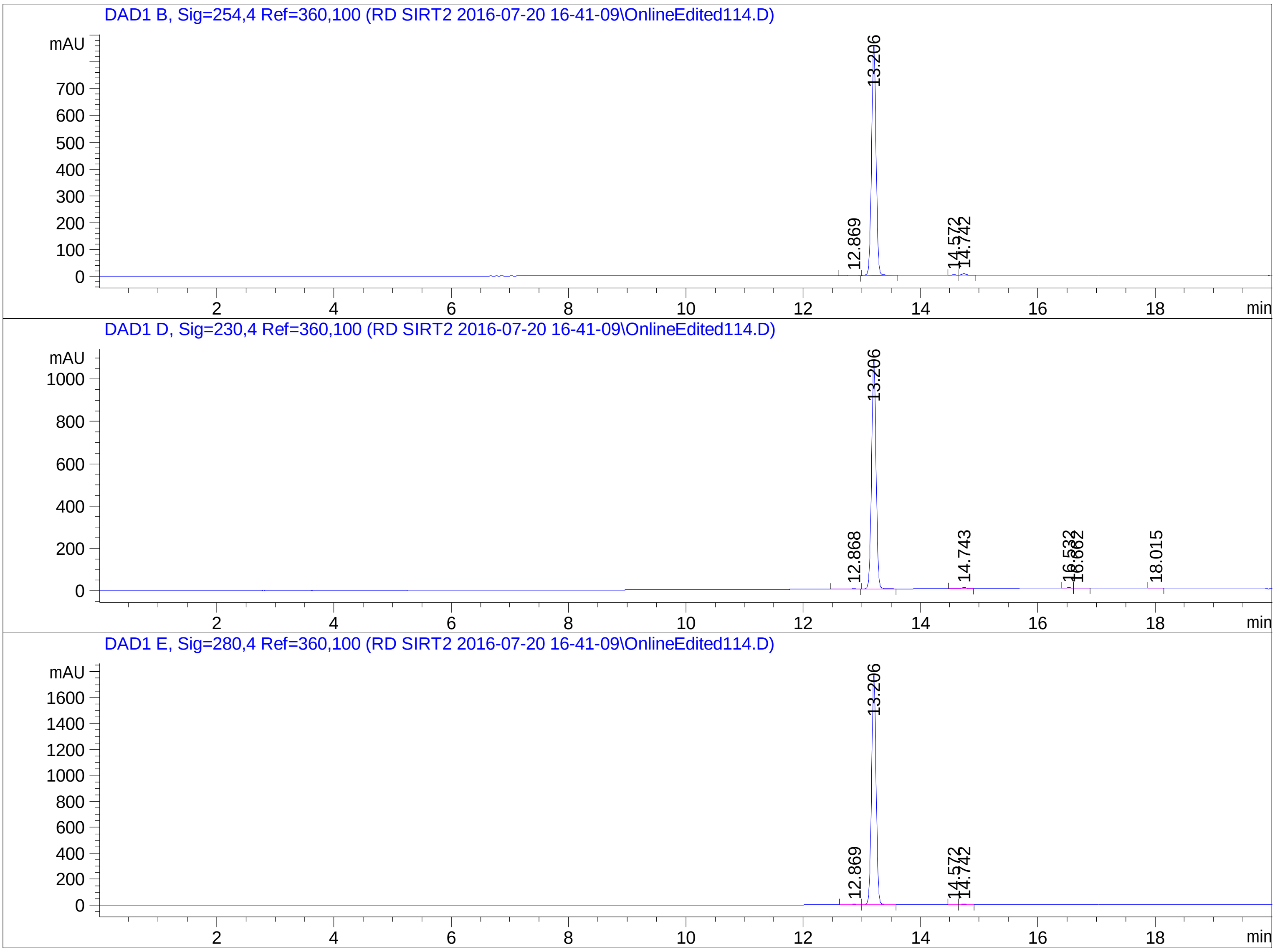

===

Area Percent Report

===

```
Sorted By             :      Signal
Multiplier            :      1.0000
Dilution              :      1.0000
Use Multiplier & Dilution Factor with ISTDs
```

Signal 1: DAD1 B, Sig=254,4 Ref=360,100

Peak #	RetTime [min]	Type	Width [min]	Area [mAU*s]	Height [mAU]	Area %
1	12.869	BB	0.0902	12.29304	2.04224	0.2657
2	13.206	BB	0.0821	4575.25049	859.28052	98.8876
3	14.572	BV	0.0763	8.16465	1.69185	0.1765
4	14.742	VB	0.0809	31.01038	5.93868	0.6702

Totals : 4626.71856 868.95329

Signal 2: DAD1 D, Sig=230,4 Ref=360,100

Peak #	RetTime [min]	Type	Width [min]	Area [mAU*s]	Height [mAU]	Area %
1	12.868	BB	0.1018	13.54542	1.93116	0.2310
2	13.206	BB	0.0822	5777.85645	1083.84375	98.5337
3	14.743	VB R	0.0864	28.74022	5.04623	0.4901
4	16.532	VV	0.0997	18.02506	2.70627	0.3074
5	16.662	VV	0.0995	13.36527	1.91238	0.2279
6	18.015	BB	0.0953	12.30610	2.01241	0.2099

Totals : 5863.83851 1097.45220

Signal 3: DAD1 E, Sig=280,4 Ref=360,100

Peak #	RetTime [min]	Type	Width [min]	Area [mAU*s]	Height [mAU]	Area %
1	12.869	BB	0.0863	28.31993	4.98116	0.2963
2	13.206	BB	0.0822	9481.54199	1778.20898	99.1996
3	14.572	BV	0.0767	12.86234	2.64438	0.1346
4	14.742	VB	0.0808	35.32034	6.77233	0.3695

Totals : 9558.04460 1792.60687

==

*** End of Report ***

**Sample Name**	2-32 hrms2	**Position**	10	**Instrument Name**	Instrument 1	**User Name**	Teng Ai
**Inj Vol**	5	**InjPosition**		**SampleType**	Unknown	**IRM Calibration Status**	Success
**Data Filename**	2-32 HRMS2-MMI_APCI_	**ACQ Method**	MMI_APCI_POSITIVE.M	**Comment**	Easy-Access Method: 'MMI_APCI_POSITIVE'	**Acquired Time**	9/16/2011 3:33:42 PM

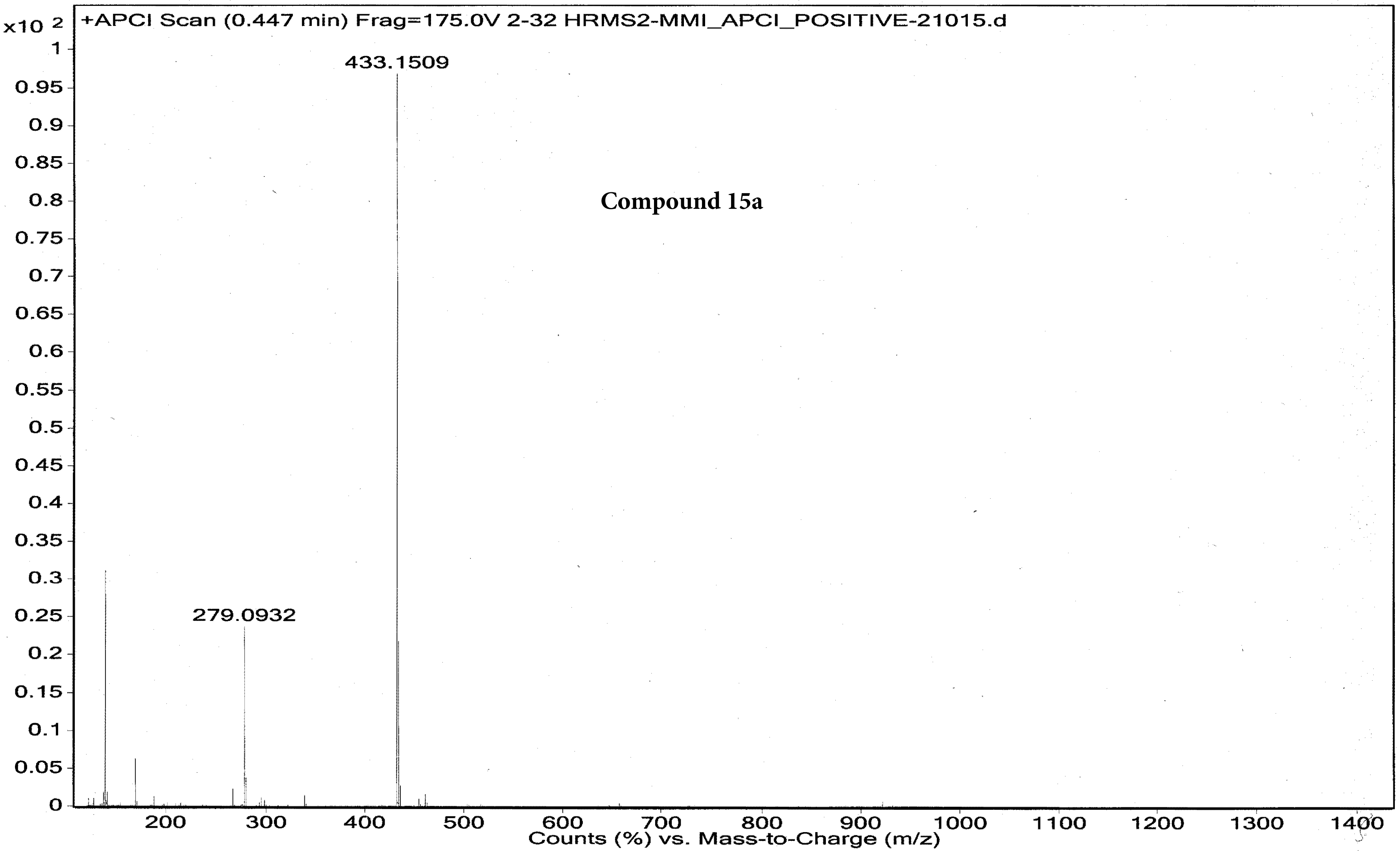

**Sample Name**	3-91hmrs	**Position**	59	**Instrument Name**	Instrument 1	**User Name**	Teng Ai
**Inj Vol**	5	**InjPosition**		**SampleType**	Unknown	**IRM Calibration Status**	Success
**Data Filename**	3-91HMRS-MMI_APCI_PO	**ACQ Method**	MMI_APCI_POSITIVE.M	**Comment**	Easy-Access Method: 'MMI_APCI_POSITIVE'	**Acquired Time**	3/12/2012 10:26:50 AM

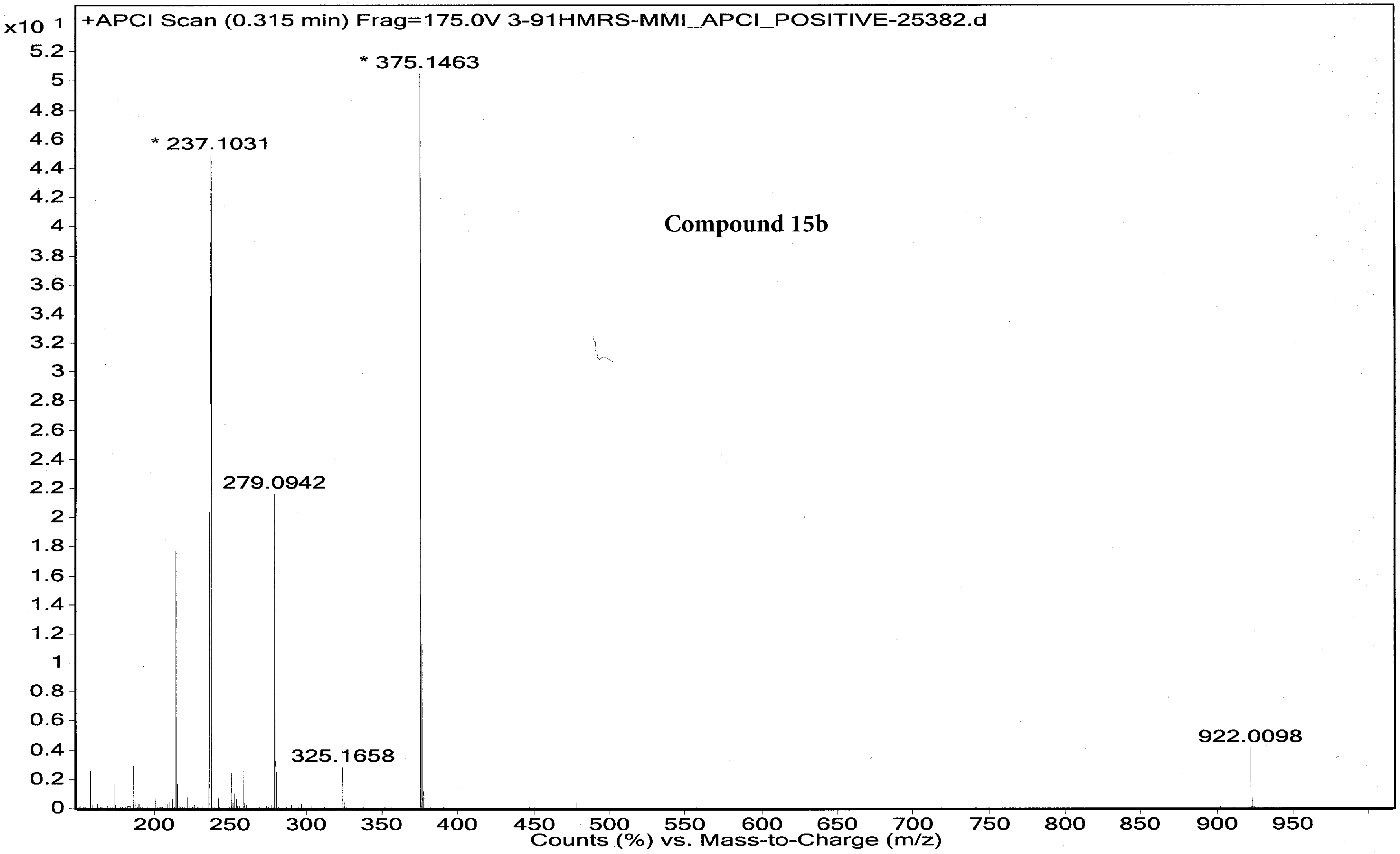

**Sample Name**	2-34 hrms2	**Position**	11	**Instrument Name**	Instrument 1	**User Name**	Teng Ai
**Inj Vol**	5	**InjPosition**		**SampleType**	Unknown	**IRM Calibration Status**	Success
**Data Filename**	2-34 HRMS2-MMI_APCI_	**ACQ Method**	MMI_APCI_POSITIVE.M	**Comment**	Easy-Access Method: 'MMI_APCI_POSITIVE'	**Acquired Time**	9/16/2011 3:38:52 PM

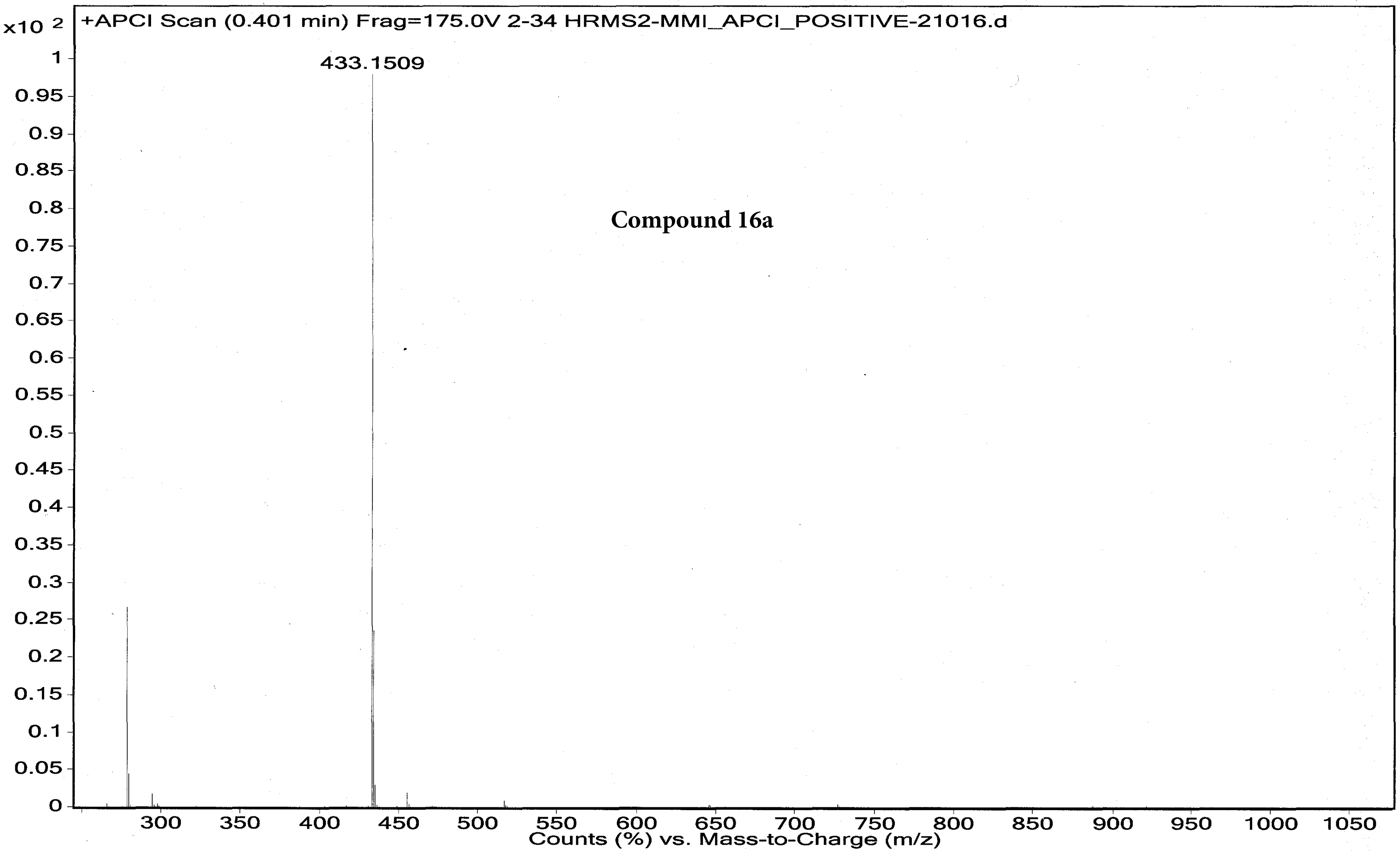

**Sample Name**	3-119hrms	**Position**	16	**Instrument Name**	Instrument 1	**User Name**	Teng Ai
**Inj Vol**	10	**InjPosition**		**SampleType**	Unknown	**IRM Calibration Status**	Success
**Data Filename**	3-119HRMS-MMI_APCI_P	**ACQ Method**	MMI_APCI_POSITIVE.M	**Comment**	Easy-Access Method: 'MMI_APCI_POSITIVE'	**Acquired Time**	3/22/2012 12:51:11 PM

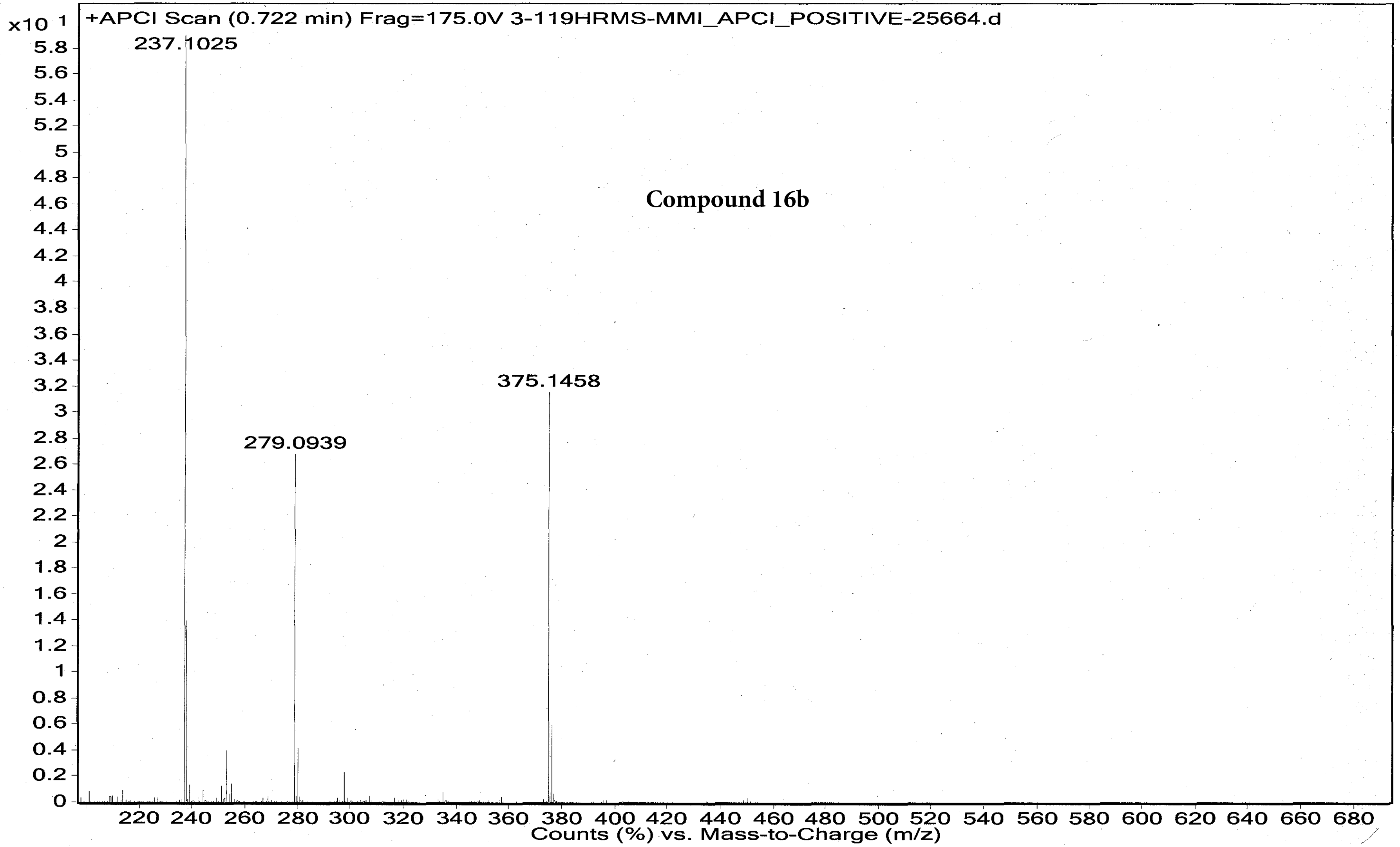

**Sample Name**	3-42	**Position**	44	**Instrument Name**	Instrument 1	**User Name**	Teng Ai
**Inj Vol**	10	**InjPosition**		**SampleType**	Unknown	**IRM Calibration Status**	Success
**Data Filename**	3-42-MMI_APCI_POSITI	**ACQ Method**	MMI_APCI_POSITIVE.M	**Comment**	Easy-Access Method: 'MMI_APCI_POSITIVE'	**Acquired Time**	10/28/2011 12:06:44 PM

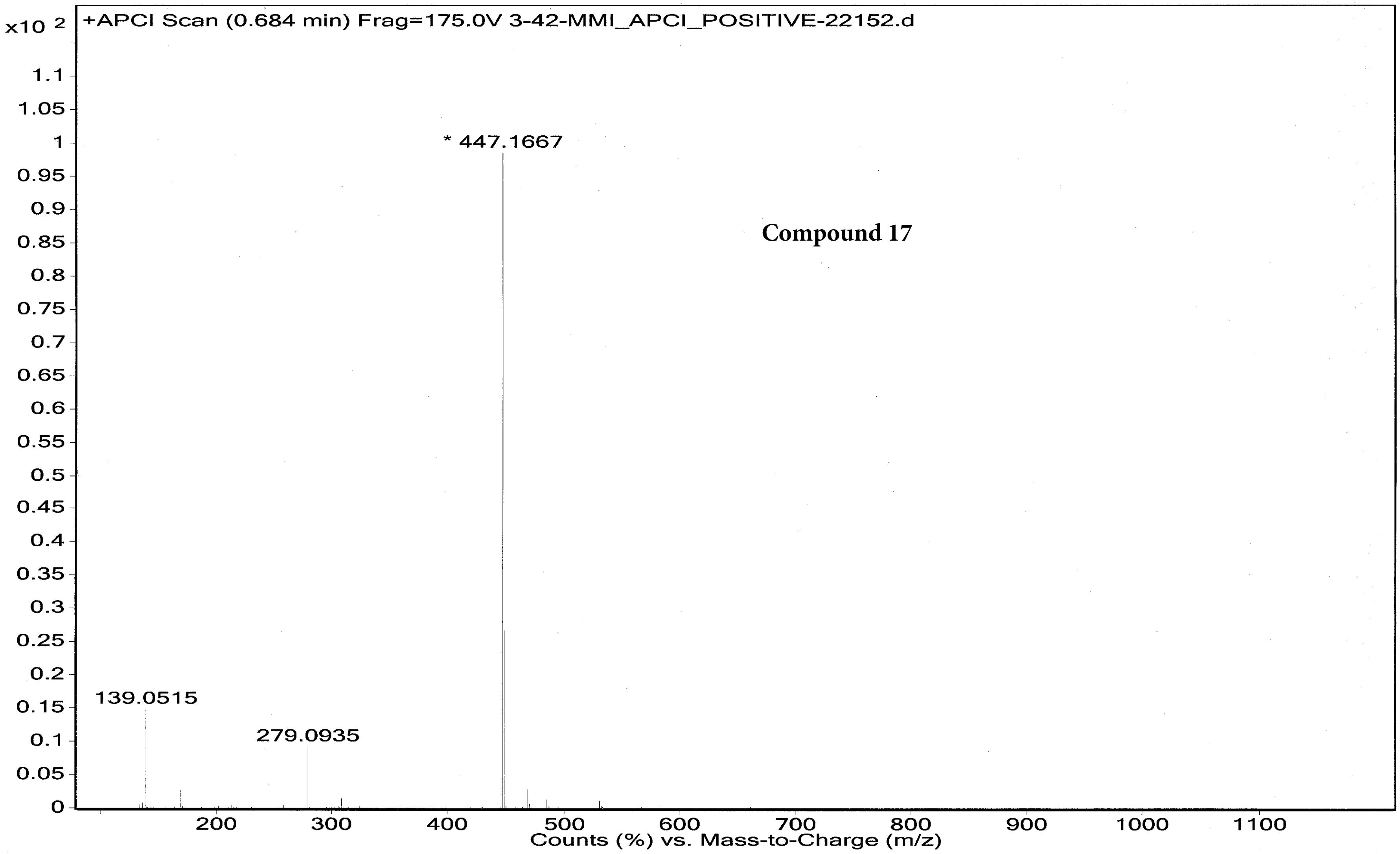

**Sample Name**	3-37hrms	**Position**	57	**Instrument Name**	Instrument 1	**User Name**	Teng Ai
**Inj Vol**	5	**InjPosition**		**SampleType**	Unknown	**IRM Calibration Status**	Success
**Data Filename**	3-37HRMS-MMI_APCI_PO	**ACQ Method**	MMI_APCI_POSITIVE.M	**Comment**	Easy-Access Method: 'MMI_APCI_POSITIVE'	**Acquired Time**	10/11/2011 5:14:19 PM

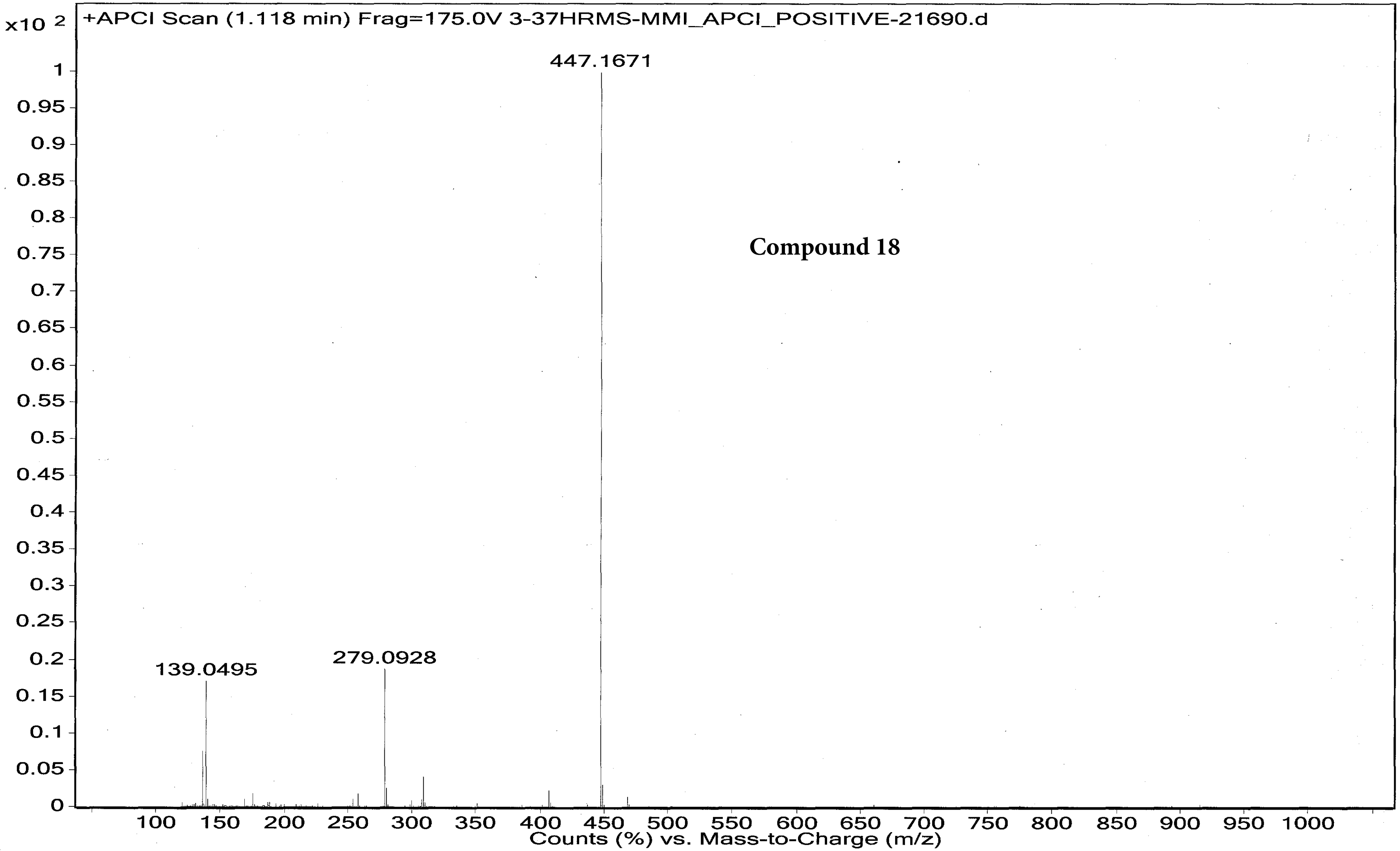

**Sample Name**	3-150hrms	**Position**	12	**Instrument Name**	Instrument 1	**User Name**	Teng Ai
**Inj Vol**	5	**InjPosition**		**SampleType**	Unknown	**IRM Calibration Status**	Success
**Data Filename**	3-150HRMS-MMI_APCI_P	**ACQ Method**	MMI_APCI_POSITIVE.M	**Comment**	Easy-Access Method: 'MMI_APCI_POSITIVE'	**Acquired Time**	4/25/2012 12:51:11 PM

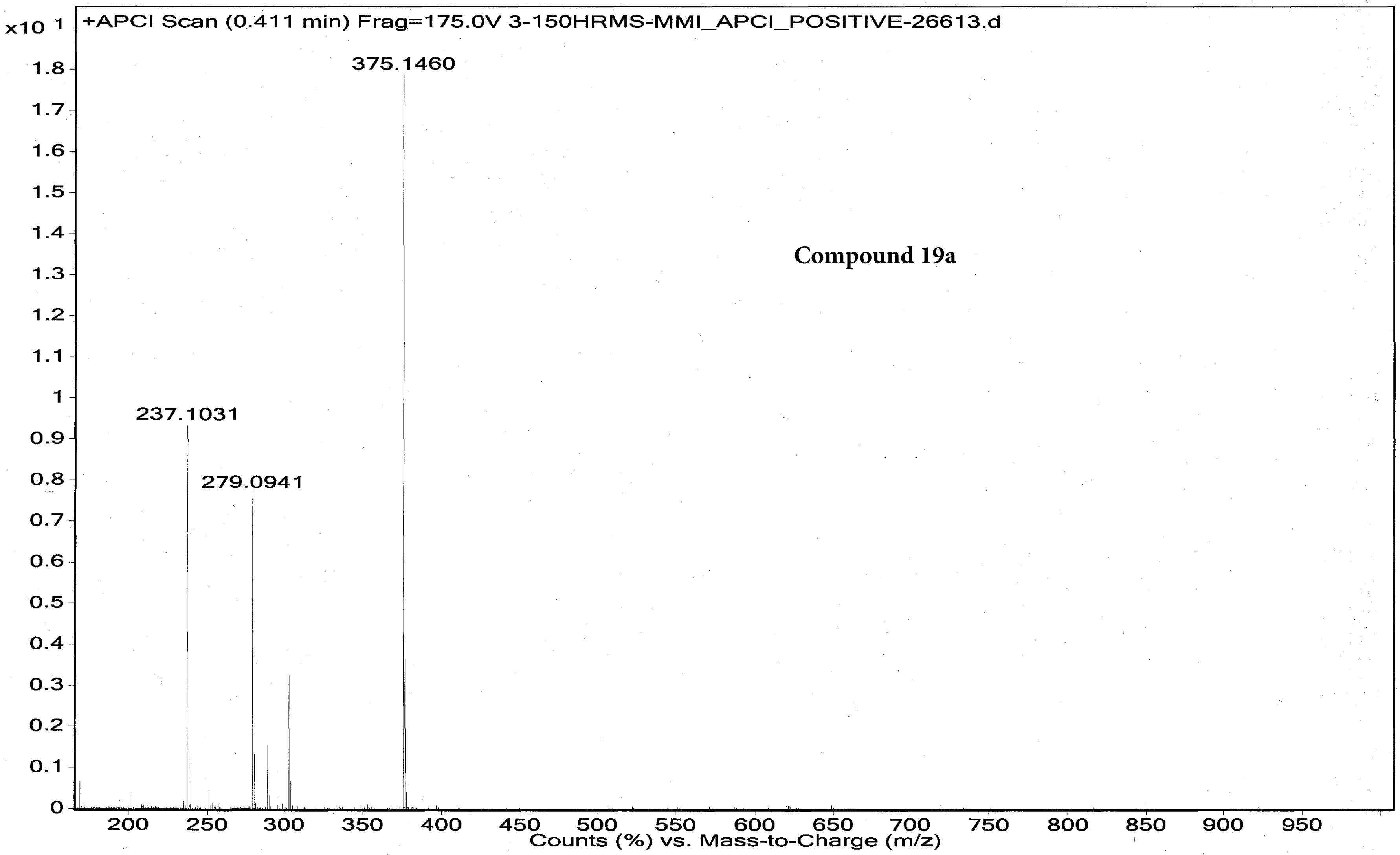

**Sample Name**	3-151hrms	**Position**	28	**Instrument Name**	Instrument 1	**User Name**	Teng Ai
**Inj Vol**	5	**InjPosition**		**SampleType**	Unknown	**IRM Calibration Status**	Success
**Data Filename**	3-151HRMS-MMI_APCI_P	**ACQ Method**	MMI_APCI_POSITIVE.M	**Comment**	Easy-Access Method: 'MMI_APCI_POSITIVE'	**Acquired Time**	5/9/2012 2:34:49 PM

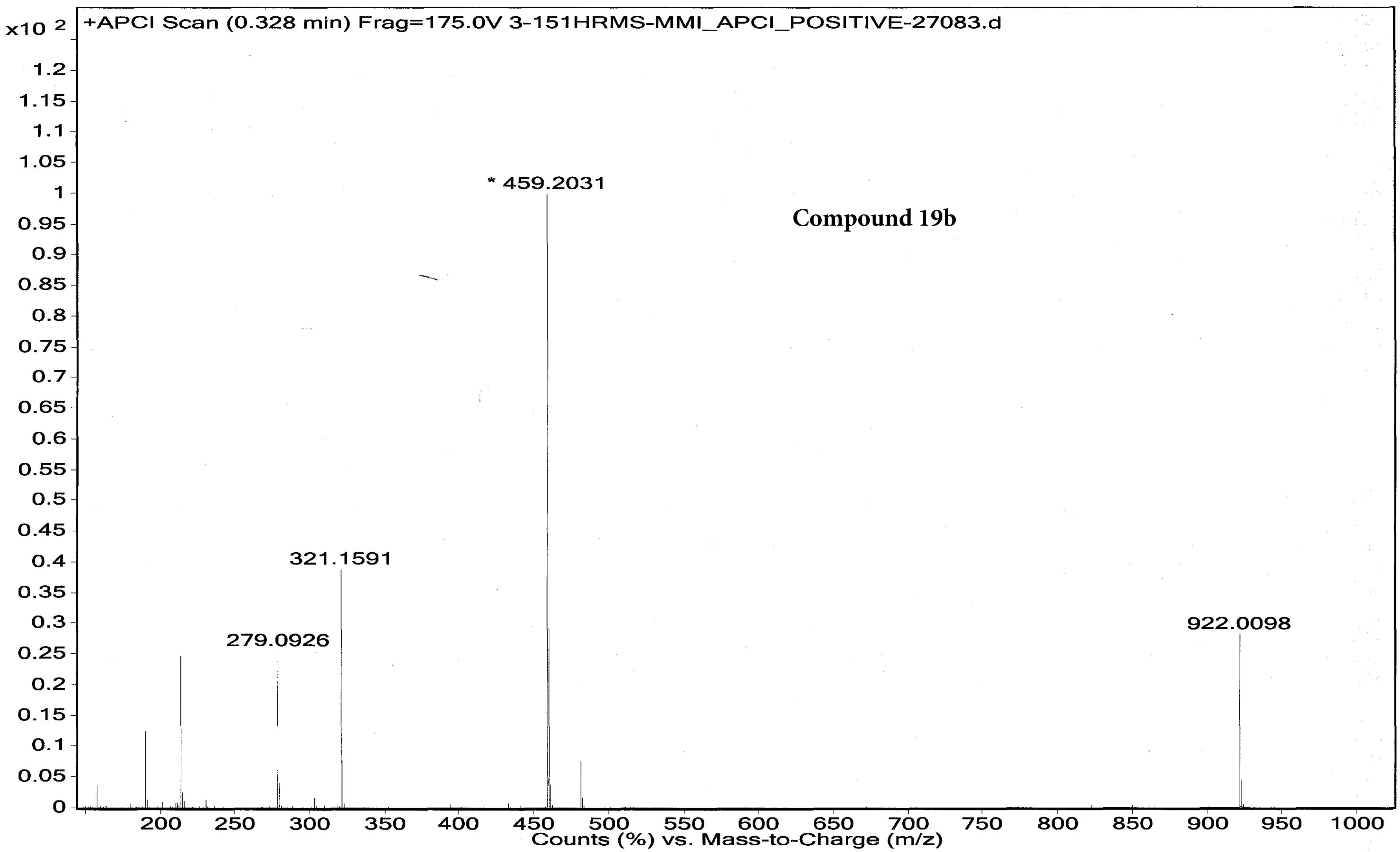

**Sample Name**	3-163hrms	**Position**	14	**Instrument Name**	Instrument 1	**User Name**	Teng Ai
**Inj Vol**	5	**InjPosition**		**SampleType**	Unknown	**IRM Calibration Status**	Success
**Data Filename**	3-163HRMS-MMI_APCI_P	**ACQ Method**	MMI_APCI_POSITIVE.M	**Comment**	Easy-Access Method: 'MMI_APCI_POSITIVE'	**Acquired Time**	4/25/2012 1:01:45 PM

Compound 19c

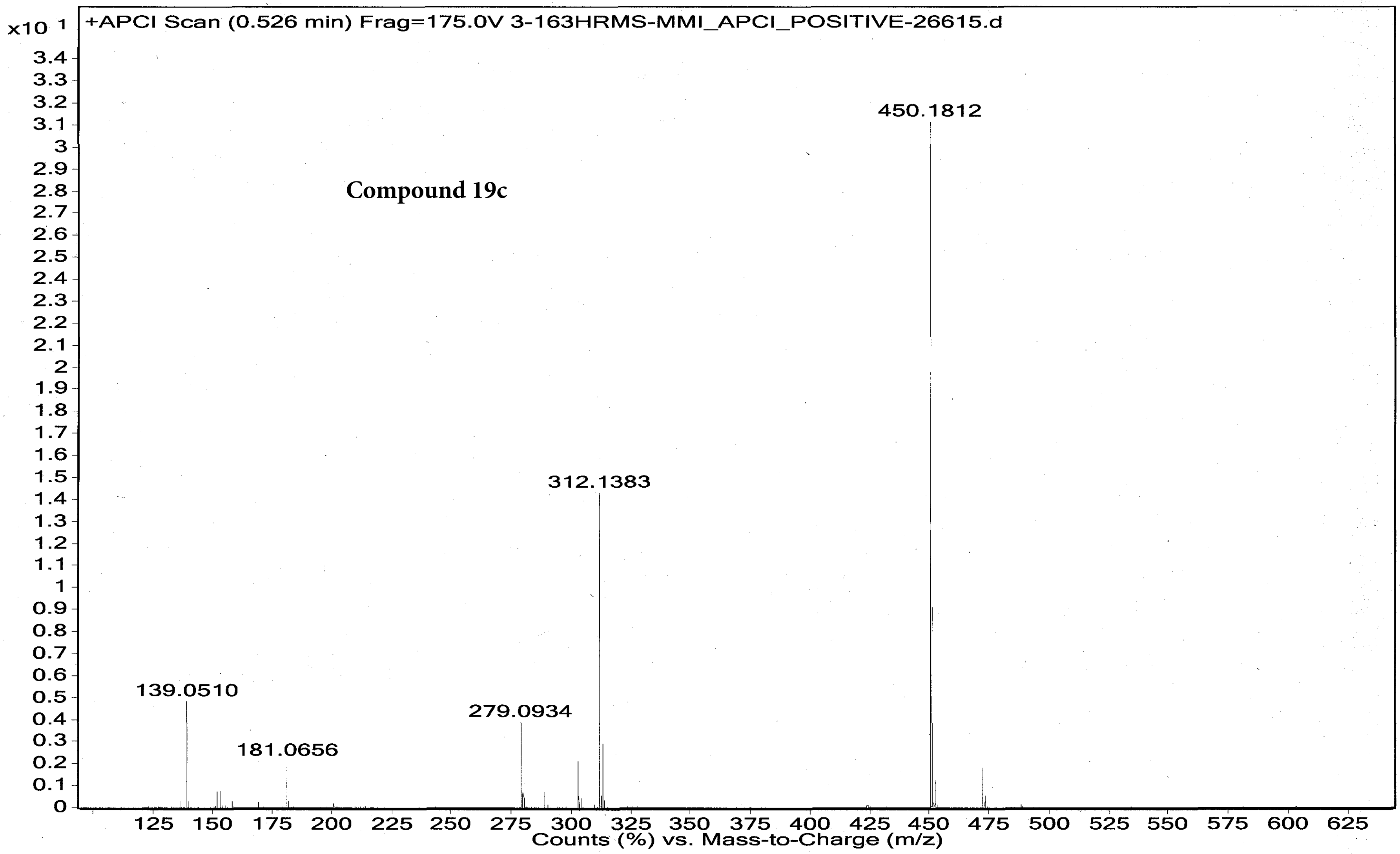

**Sample Name**	3-152hrms	**Position**	57	**Instrument Name**	Instrument 1	**User Name**	Teng Ai
**Inj Vol**	5	**InjPosition**		**SampleType**	Unknown	**IRM Calibration Status**	Success
**Data Filename**	3-152HRMS-MMI_APCI_P	**ACQ Method**	MMI_APCI_POSITIVE.M	**Comment**	Easy-Access Method: 'MMI_APCI_POSITIVE'	**Acquired Time**	5/2/2012 6:04:15 PM

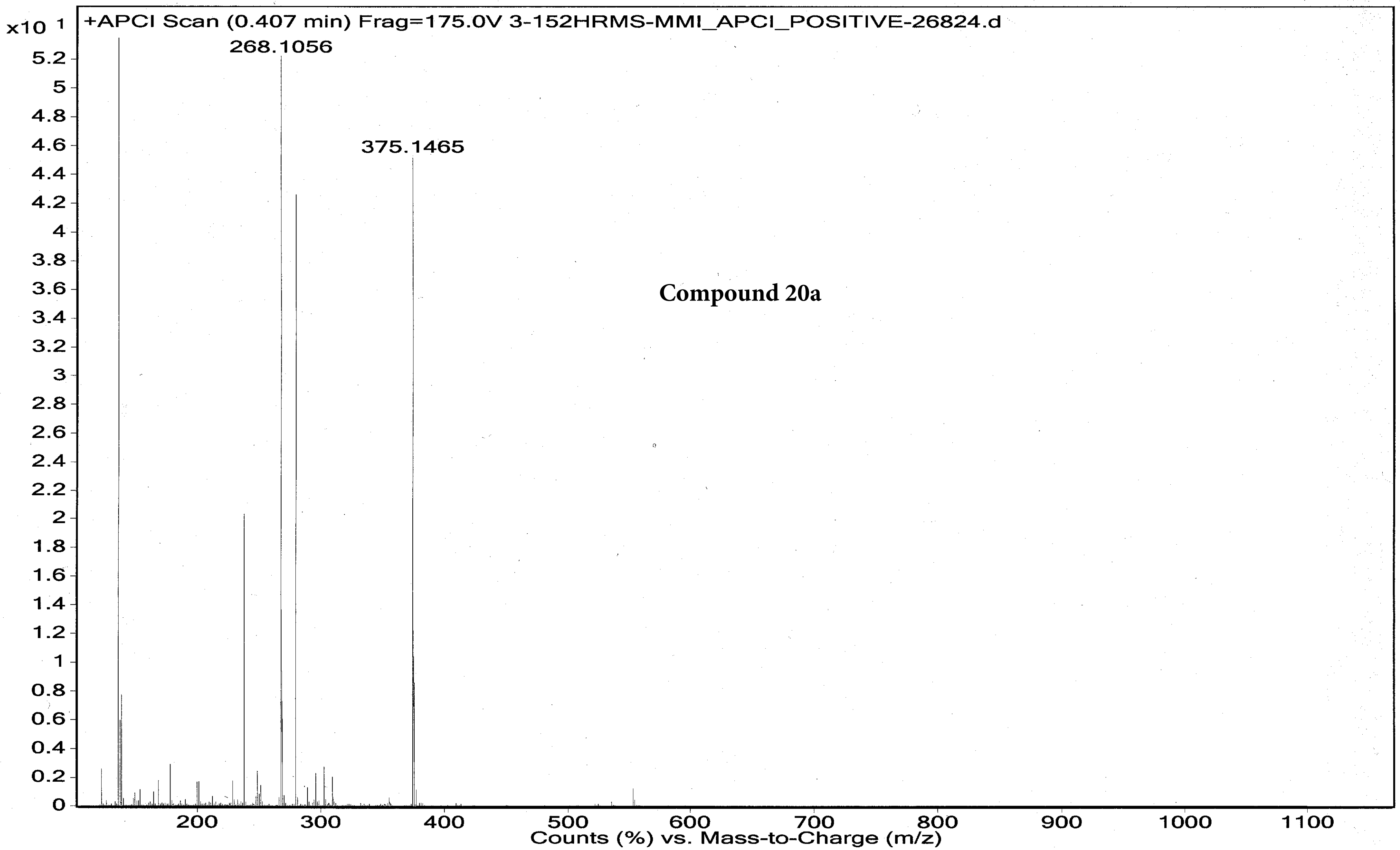

Compound 20a

**Sample Name**	3-153hrms	**Position**	52	**Instrument Name**	Instrument 1	**User Name**	Teng Ai
**Inj Vol**	5	**InjPosition**		**SampleType**	Unknown	**IRM Calibration Status**	Success
**Data Filename**	3-153HRMS-MMI_APCI_P	**ACQ Method**	MMI_APCI_POSITIVE.M	**Comment**	Easy-Access Method: 'MMI_APCI_POSITIVE'	**Acquired Time**	5/30/2012 4:14:13 PM

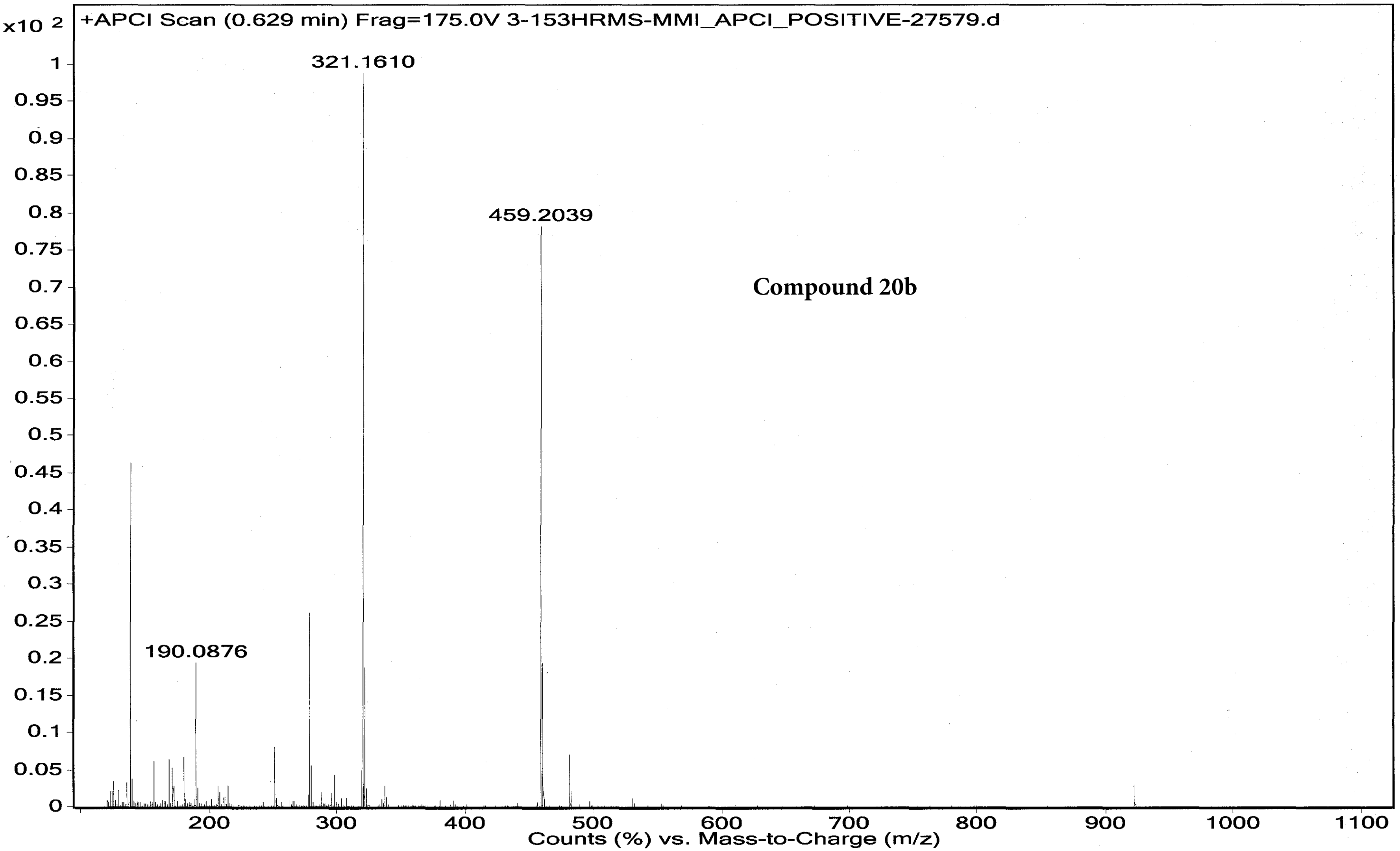

**Sample Name**	3-194	**Position**	55	**Instrument Name**	Instrument 1	**User Name**	Teng Ai
**Inj Vol**	10	**InjPosition**		**SampleType**	Unknown	**IRM Calibration Status**	Success
**Data Filename**	3-194-MMI_APCI_POSIT	**ACQ Method**	MMI_APCI_POSITIVE.M	**Comment**	Easy-Access Method: 'MMI_APCI_POSITIVE'	**Acquired Time**	9/30/2013 3:38:46 PM

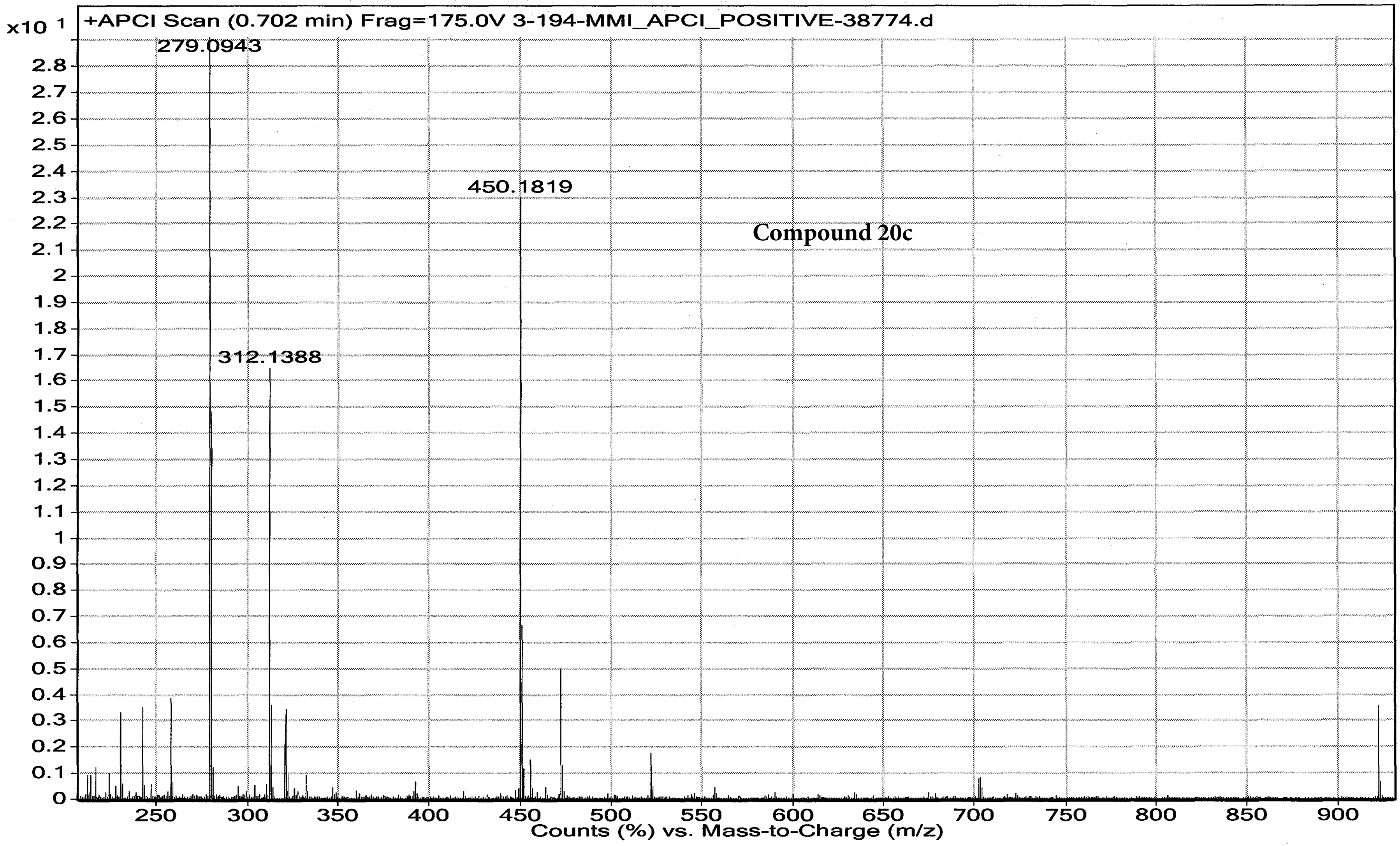

**Sample Name**	3-165hrms	**Position**	42	**Instrument Name**	Instrument 1	**User Name**	Teng Ai
**Inj Vol**	5	**InjPosition**		**SampleType**	Unknown	**IRM Calibration Status**	Success
**Data Filename**	3-165HRMS-MMI_APCI_P	**ACQ Method**	MMI_APCI_POSITIVE.M	**Comment**	Easy-Access Method: 'MMI_APCI_POSITIVE'	**Acquired Time**	5/8/2012 11:34:24 AM

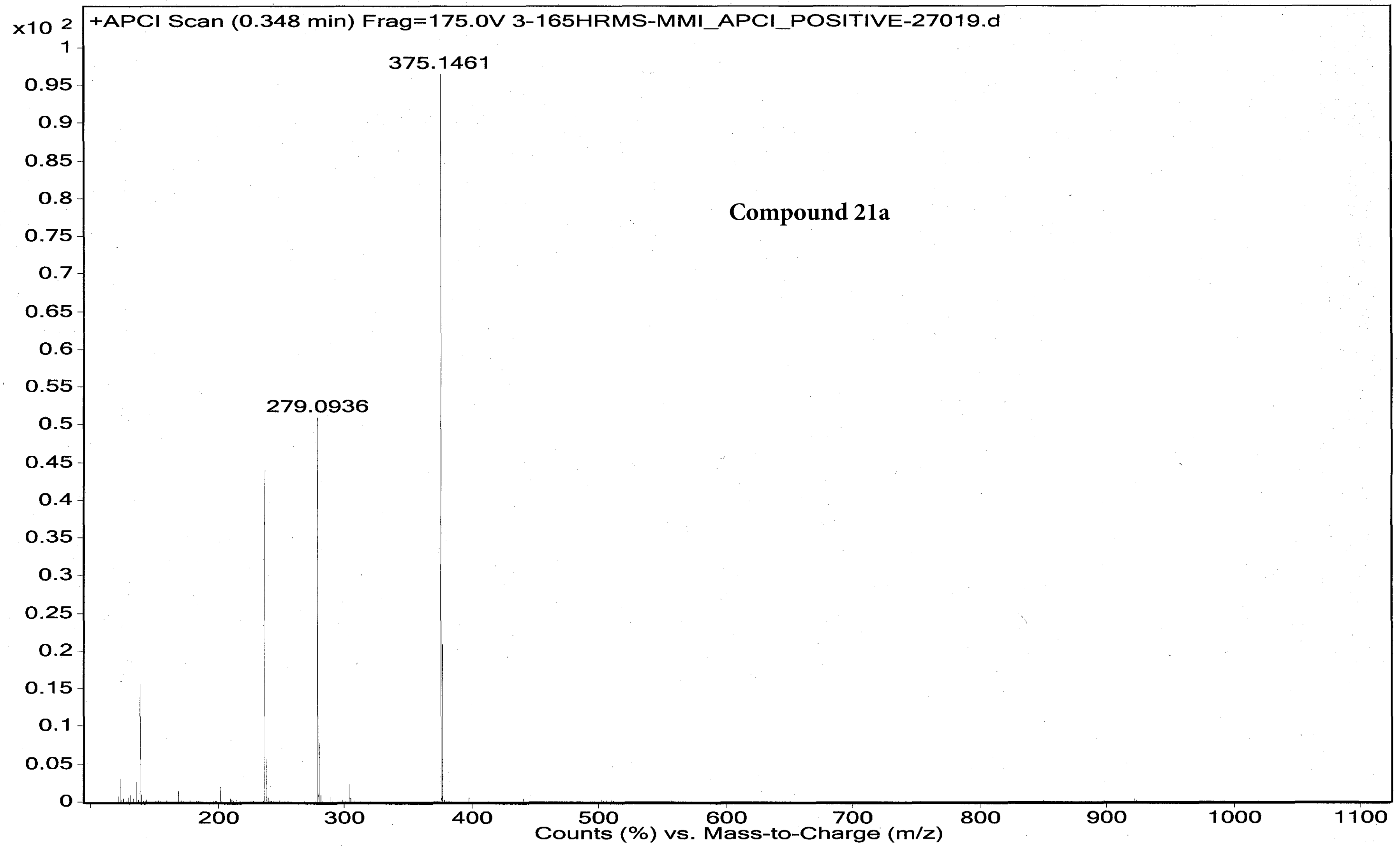

**Sample Name**	3-166hrms	**Position**	25	**Instrument Name**	Instrument 1	**User Name**	Teng Ai
**Inj Vol**	5	**InjPosition**		**SampleType**	Unknown	**IRM Calibration Status**	Success
**Data Filename**	3-166HRMS-MMI_APCI_P	**ACQ Method**	MMI_APCI_POSITIVE.M	**Comment**	Easy-Access Method: 'MMI_APCI_POSITIVE'	**Acquired Time**	5/1/2012 6:00:28 PM

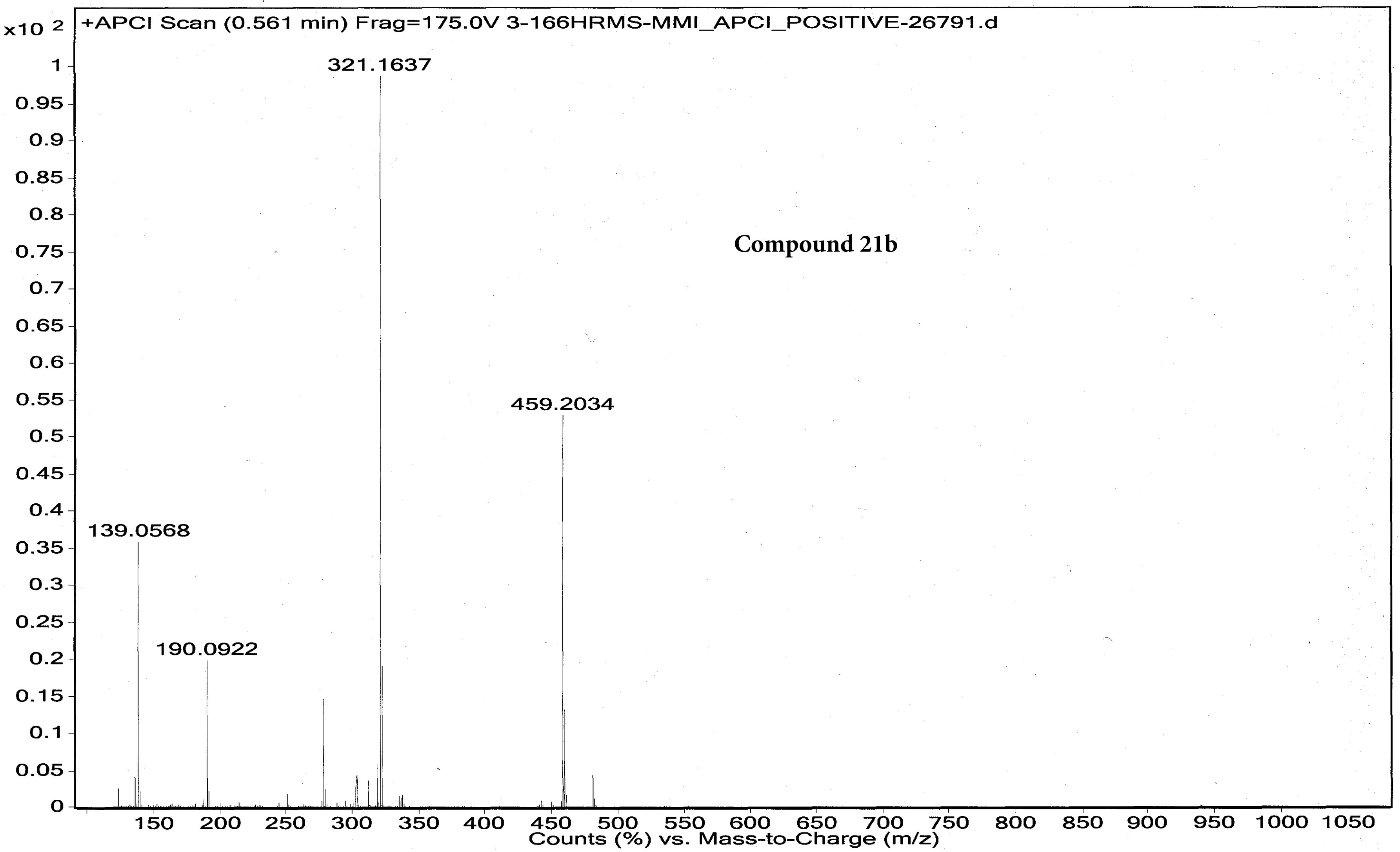

**Sample Name**	3-164hrms	**Position**	24	**Instrument Name**	Instrument 1	**User Name**	Teng Ai
**Inj Vol**	5	**InjPosition**		**SampleType**	Unknown	**IRM Calibration Status**	Success
**Data Filename**	3-164HRMS-MMI_APCI_P	**ACQ Method**	MMI_APCI_POSITIVE.M	**Comment**	Easy-Access Method: 'MMI_APCI_POSITIVE'	**Acquired Time**	5/1/2012 5:55:13 PM

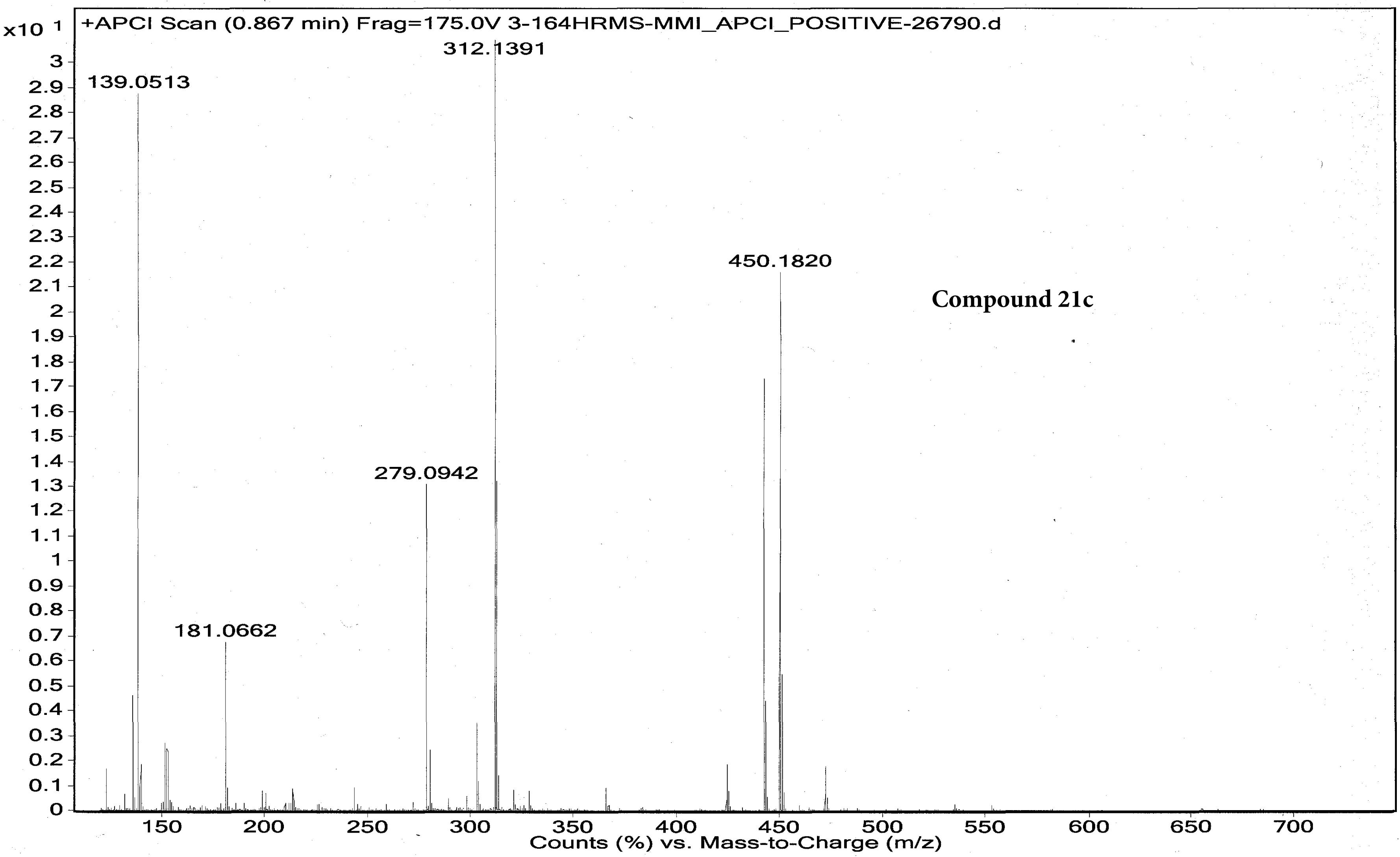

Compound 21c

**Sample Name**	3-160hrms	**Position**	53	**Instrument Name**	Instrument 1	**User Name**	Teng Ai
**Inj Vol**	5	**InjPosition**		**SampleType**	Unknown	**IRM Calibration Status**	Success
**Data Filename**	3-160HRMS-MMI_APCI_P	**ACQ Method**	MMI_APCI_POSITIVE.M	**Comment**	Easy-Access Method: 'MMI_APCI_POSITIVE'	**Acquired Time**	5/30/2012 4:19:30 PM

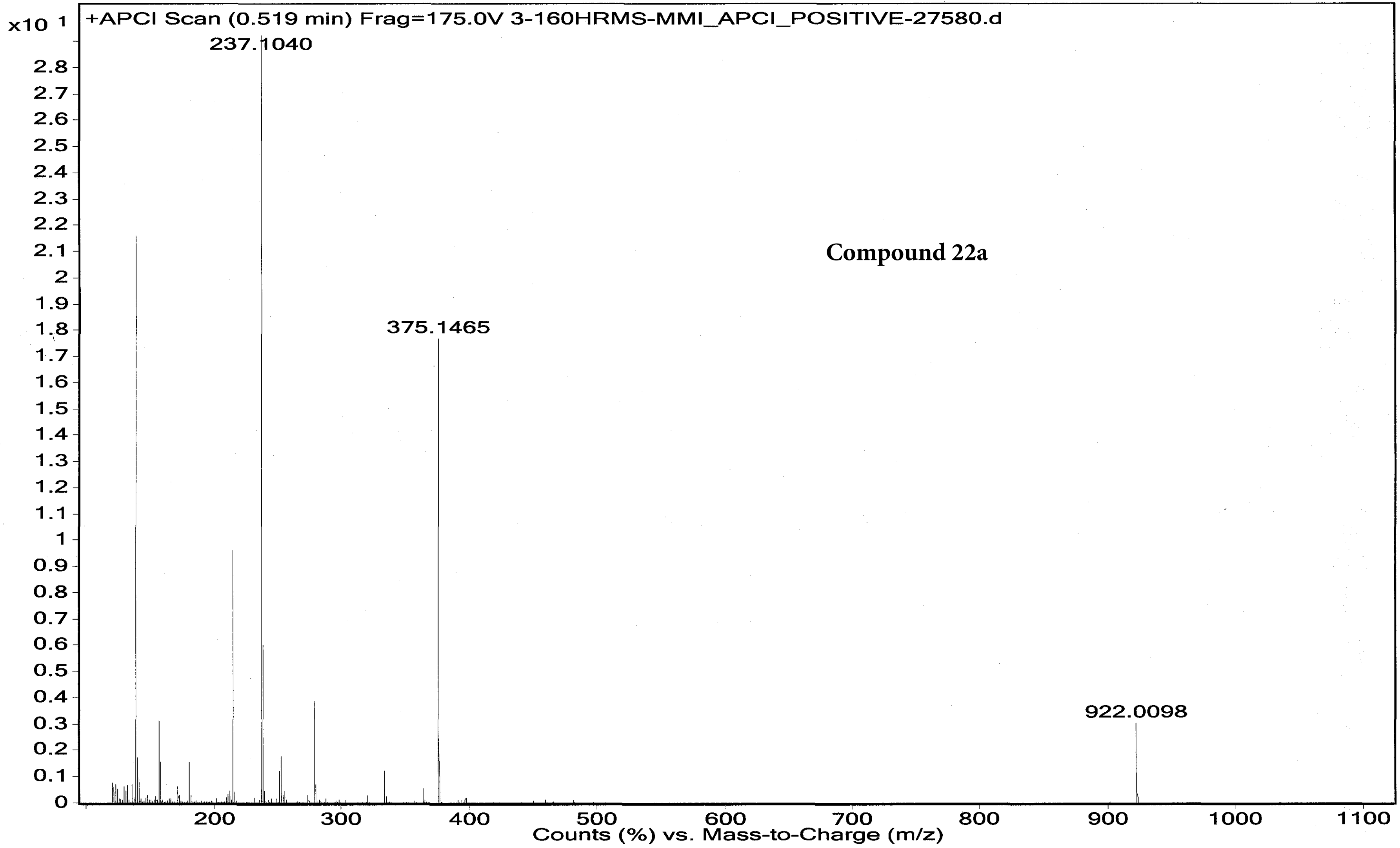

Compound 22a

**Sample Name**	3-161hrms	**Position**	23	**Instrument Name**	Instrument 1	**User Name**	Teng Ai
**Inj Vol**	5	**InjPosition**		**SampleType**	Unknown	**IRM Calibration Status**	Success
**Data Filename**	3-161HRMS-MMI_APCI_P	**ACQ Method**	MMI_APCI_POSITIVE.M	**Comment**	Easy-Access Method: 'MMI_APCI_POSITIVE'	**Acquired Time**	5/1/2012 5:49:56 PM

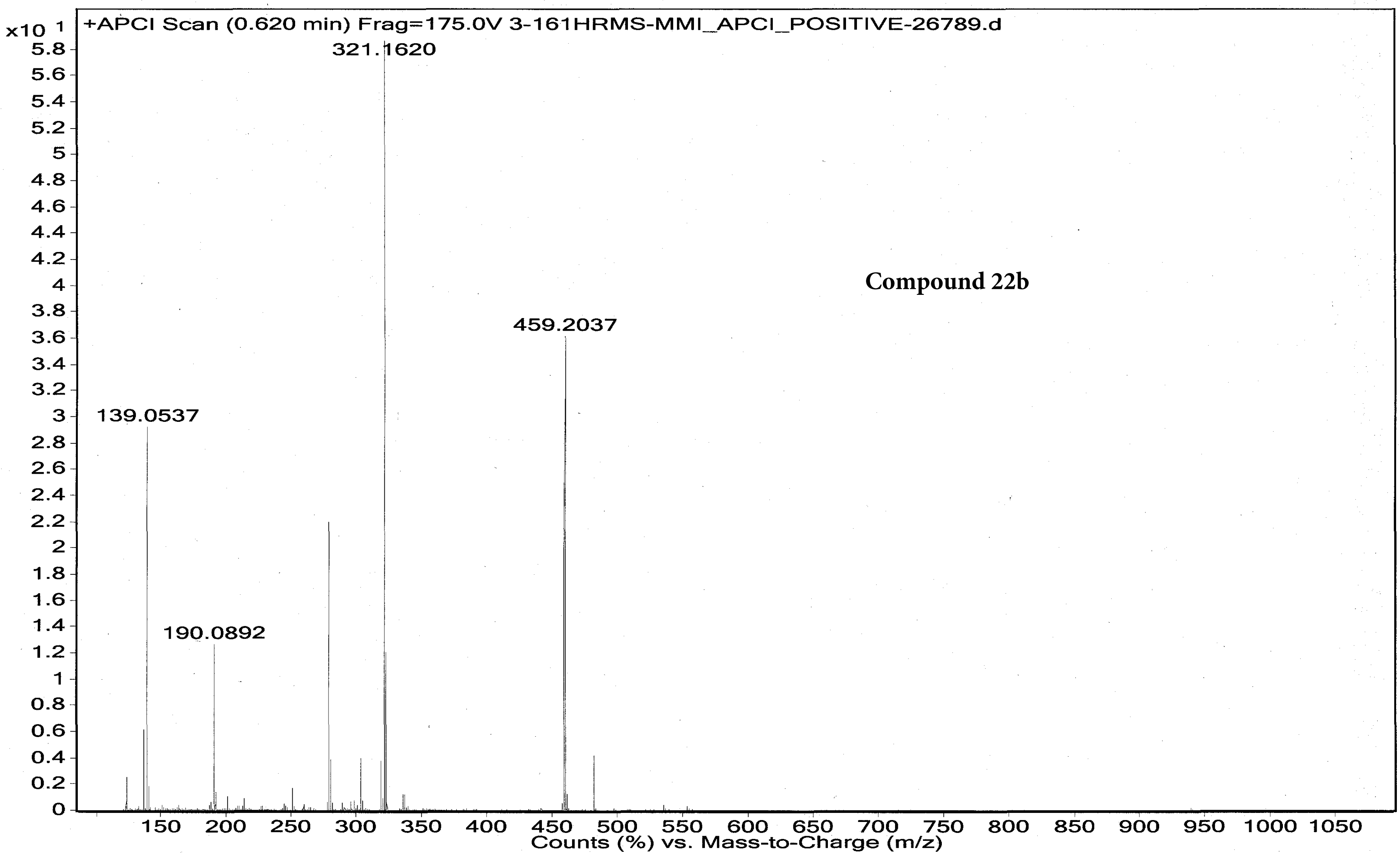

Compound 22b

**Sample Name**	3-162hrms	**Position**	13	**Instrument Name**	Instrument 1	**User Name**	Teng Ai
**Inj Vol**	5	**InjPosition**		**SampleType**	Unknown	**IRM Calibration Status**	Success
**Data Filename**	3-162HRMS-MMI_APCI_P	**ACQ Method**	MMI_APCI_POSITIVE.M	**Comment**	Easy-Access Method: 'MMI_APCI_POSITIVE'	**Acquired Time**	4/25/2012 12:56:24 PM

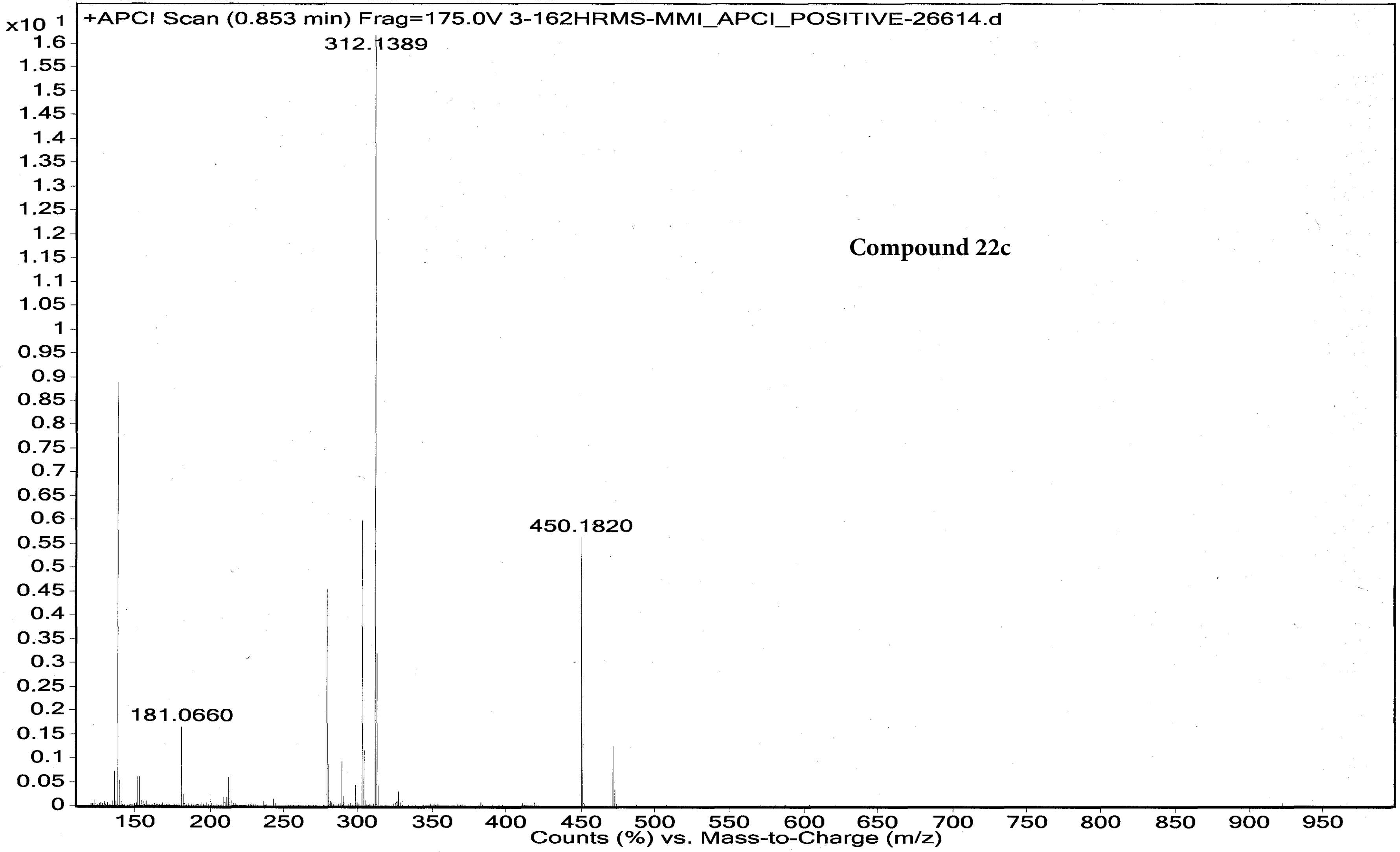

Supplement: Supplementary file 1 [file molecules-28-07655-s001.zip › molecules-2666390-supplementary.pdf]
